# Supplementary material for: Internet-delivered attentional bias modification training (iABMT) for the management of chronic musculoskeletal pain: a protocol for a randomised controlled trial
Source: BMJ Open. 2020 Feb 20;10(2):e030607. doi: 10.1136/bmjopen-2019-030607 (PMC7045192; doi:10.1136/bmjopen-2019-030607)
Supplement: Supplementary data [file bmjopen-2019-030607supp001.pdf]

ATTENTIONAL BIAS MODIFICATION PROTOCOL

Supplementary Material 1

Stimuli Development and Properties

A detailed description of the stimuli to be used in the iAMBT intervention is presented in this document. Linguistic stimulus properties and analyses on written frequency, valence, and arousal are first presented, followed by a description of pictorial stimuli properties and analyses of low-level features, valence, arousal and pain intensity. Following this, comparisons of stimuli properties between visual-probe sessions are then presented for linguistic and pictorial stimuli.

Linguistic stimuli

Properties. Linguistic and pictorial versions of the visual-probe task will be used. Each linguistic visual-probe task will include 32 threatening/neutral and 16 neutral/neutral word pairs. Threatening words stem from the following categories, with 8 words from each included in each visual-probe task: (i) sensory-pain words reflecting the sensory dimension of pain (e.g., *shooting*; *burning*; *aching*); (ii) affective-pain words reflecting the unpleasantness of pain and negative emotions associated with pain (e.g., *hopeless*, *punishing*, *tormenting*); (iii) health-threat words reflecting threat to health and well-being (e.g., *hospital, doctor*, *stress*); and (iv) general-threat words reflecting physical and emotional sources of threat, including manmade threat (e.g., *gun*), natural threat (e.g., *fire*), social threat (e.g., *humiliation*), and emotional threat (e.g., *sad*). Neutral words (including the neutral counterparts of the threatening words) include non-threatening words unrelated to pain or suffering (e.g., *version*; *rector*; *monument*), which were matched with their threatening counterparts on Kucera-Francis written frequency of use in the English language[1] and word length. Matching on written frequency minimise the chances of participants preferentially processing one stimulus due to its high frequency of use (e.g., faster recognition) when displayed simultaneously with another stimulus[2 3], while matching word length also minimise the chances of participants attending towards a stimulus due to its size.

In total, 864 words have been collected from previous studies exploring and modifying biases in pain and/or anxiety (i.e.,[4-8]), including words from the McGill Pain Questionnaire.[9]

Liossi C, *et al. BMJ Open* 2020; 10:e030607. doi: 10.1136/bmjopen-2019-030607

ATTENTIONAL BIAS MODIFICATION PROTOCOL

Matching of threatening and neutral words was achieved via the MRC Psycholinguistic database[10], an online psycholinguistic tool that generates words with specific lexical characteristics. The database includes a dictionary of 150,837 English words providing syntactic, semantic and orthographical properties of each word.[11] Valence and arousal can influence attentional allocation to external stimuli.[12 13] A computerised version of the SAM task[14] was therefore used to collect ratings on valence and arousal for each word in a sample of sixteen healthy, pain-free participants (M_{age} = 25.9, SD = 4.08, range: 20 – 33 years; 8 female). All words were randomly presented to participants for 3 seconds each. Following each word, two 9-point SAM scales were presented, one for valence (1 = low pleasure to 9 = high pleasure) and one for arousal (1 = low arousal to 9 = high arousal). As per former chronic pain attentional bias research (e.g.,[5 15 16]), stimuli categories were assessed but not matched on valence and arousal, as differences are expected to exist between categories.

Analyses and results: written frequency, valence, and arousal. Analyses comparing properties between the five word categories are presented here, while analyses comparing word properties between the nine linguistic visual-probe tasks are presented below. One-way ANOVA was conducted comparing Kucera-Francis written frequency (Table 1) across words in the five word categories, revealing no significant main effect, $F(4, 853) = 1.97$, $p = .10$.

A repeated-measures ANOVA was conducted comparing arousal ratings (Table 2) across the five word categories, revealing a significant main effect, $F(2, 25) = 18.78$, $p < .001$, $\eta p^2 = .56$. Pairwise comparisons revealed the neutral category had significantly lower arousal scores than sensory-pain (*Md* [mean difference] = 1.82, *SE* = 0.45, *p* = .001), affective-pain (*Md* = 2.36, *SE*= 0.46, *p* = .001), general-threat (*Md* = 2.20, *SE* = 0.47, *p* = .003) and health-threat (*Md* = 2.05, *SE* = 0.35, *p* < .001) categories. Statistically significant differences were not found between the arousal scores of the words included in the four threat-related categories.

Liossi C, *et al. BMJ Open* 2020; 10:e030607. doi: 10.1136/bmjopen-2019-030607

ATTENTIONAL BIAS MODIFICATION PROTOCOL

Table 1

Mean Kucera-Francis Written Frequency per Word Category

Word category	*M*	*SD*
Sensory-pain	19.07	27.01
Affective-pain	17.28	20.63
General-threat	25.00	32.10
Health-threat	21.36	36.39
All neutral words	22.60	27.97

Table 2

Mean Arousal and Valence Ratings per Word Category

	Arousal		Valence	
Word Category	*M*	*SD*	*M*	*SD*
Sensory-pain	3.80	1.85	3.95	0.83
Affective-pain	4.34	1.64	3.29	0.73
General-threat	4.19	1.78	3.35	0.79
Health-threat	4.03	1.32	3.48	0.68
Sensory-pain neutral counterparts	2.18	1.28	5.33	0.41
Affective-pain neutral counterparts	2.19	1.32	5.29	0.29
General-threat neutral counterparts	2.06	1.28	5.24	0.33
Health-threat neutral counterparts	2.05	1.19	5.19	0.36
Neutral/Neutral	1.95	1.32	5.29	0.33

A repeated-measures ANOVA was conducted comparing valence ratings (Table 2) across the five word categories, revealing a significant main effect, $F(2, 29) = 52.00$, $p < .001$, $\eta p^2 = .78$. Pairwise comparisons showed the neutral category had significantly higher pleasantness scores than sensory-pain (*Md* = 1.33, *SE* = 0.23, *p* <. 001) affective-pain (*Md* = 1.99, *SE* = 0.22, *p* < .001), general-threat (*Md* = 1.93, *SE* = 0.23, *p* <. 001) and health-threat (*Md* = 1.80, *SE* = 0.32, *p* < .001) categories. The words in the sensory-pain category were rated as more pleasant than

words included in the affective-pain (Md = 0.66, SE = 0.11, $p < .001$), general-threat (Md = 0.60, SE = 0.11, $p < .001$) and health-threat (Md = 0.47, SE = 0.11, p = .01) categories.

Pictorial Stimuli

Properties. Pictorial visual-probe tasks each include 32 threatening/neutral and 16 neutral/neutral image pairs. Threatening images stem from the following categories: (i) musculoskeletal pain images depicting people experiencing pain in muscles and/or joints, including images of people performing daily activities that can be considered painful and/or harmful for individuals with chronic musculoskeletal pain (e.g., a person running; a person lifting a heavy weight with poor posture); (ii) facial expressions of pain, featuring a single model depicting a prototypical pain expression – closing the eyes or narrowing the eyelids, lowering the eyebrows, wrinkling the nose or raising the cheek, and partly opening the mouth or extending the lips[17], including horizontal lip stretching and/or oblique lip raising[18]; (iii) health-threat images reflecting threat to health and well-being (e.g., medication, wheelchair); and (iv) general-threat images reflecting physical and emotional sources of threat, including manmade threat (e.g., gun), natural threat (e.g., flood), and social threat (e.g., social isolation). Neutral images (including the neutral pairs of the threatening images) depict non-threatening objects (e.g., a car) and people performing every-day activities (e.g., a person reading a newspaper). In total, 768 images have been collected from previous studies exploring and modifying attentional biases in chronic pain (i.e.,[5 19]), including online databases providing batteries of pictorial stimuli for research (i.e., International Affective Picture System database[20]; The Photograph Series of Daily Activities Short Version[21]; NimStim Set of Facial Expressions[22]).

Each threatening image depicting a person was paired with a neutral image featuring the same person, with the same clothes and background. Each threatening image depicting an object was matched with a neutral image depicting a similar, non-threatening object. Image pairs in the neutral/neutral condition were matched in the same manner. An equal number of images depicting male and female models were used within each image category. All images were full-

colour and the same size (i.e., 265 pixels wide x 250 pixels high). Each threatening image was matched with its neutral counterpart on complexity, luminance, contrast level, and colour saturation in blue, red and green channels via visual inspection[23]. Visual attention and gaze fixations are influenced by bottom-up variables, including the saliency of objects[24] and low-level features of visual scenes.[25 26] The additional matching rules for pictorial stimuli in the present study were therefore used to minimise the chances of participants preferentially processing one stimulus due to unnecessary variations in content. Using Adobe Photoshop CS6 Extended, the means and standard deviations for all parameters were extracted for evaluation. Luminance values range from 0 to 255, where 0 indicates no light intensity (i.e., no light emission; black) and 255 indicates the highest light intensity (i.e., absolute light emission; white). Contrast levels values range from -100 to +100, with values closer to +100 indicating more contrasted image. The intensity values for red, green and blue colours range from 0 to 255, where 0 indicates black (i.e., darkest extreme) and 255 white (i.e., lightest extreme) for each colour dimension.

Visual complexity was examined as the size of each compressed image file (.jpeg) in kilobytes under the assumption that more complex images would have higher values in kilobytes.[27] As with linguistic stimuli, a computerised version of the SAM task[14] was used to collect ratings on valence and arousal for each image. Musculoskeletal and facial expressions of pain categories were also evaluated on pain intensity using a 10 cm Visual Analogue Scale (VAS). Participants were required to rate the pain that the person in the image experienced. The scale ranged from 0 to 10, where 0 indicated ‘*no pain at all*’ and 10 indicated ‘*excruciating pain*’. This rating aimed to assess the degree to which images included in the pain-related categories were perceived as depicting pain. Sixteen healthy, pain-free participants ($M_{age} = 24.8$, $SD = 4.56$, range: 20 – 33 years; 8 female) were recruited to provide ratings on valence, arousal, and pain intensity (this was a separate sample to that recruited to provide linguistic stimuli ratings).

Analyses and results: Low-level features. Analyses comparing properties between the five image categories on low-level features (Table 3) are presented here, while analyses

ATTENTIONAL BIAS MODIFICATION PROTOCOL

comparing word properties between the eight pictorial visual-probe tasks are provided below. Considering low-level features, one-way ANOVAs revealed no significant main effects for complexity, $F(4,763) = 2.18$, $p = .70$, or luminance, $F(4,763) = 2.31$, $p = .06$. A significant main effect was found for red saturation, $F(4,763) = 5.19$, $p < .001$, $\eta p^2 = .03$. Pairwise comparisons revealed images in the musculoskeletal-pain category had significantly less red saturation than images in the facial expressions of pain (*Md* = 24.24, *SE* = 7.89, $p = .022$) and neutral categories (*Md* = 19.04, *SE* = 5.91, $p = .013$). Images in the general-threat category had significantly less red saturation than facial expressions of pain (*Md* = 23.96, *SE* = 7.89, $p = .025$) and neutral categories (*Md* = 18.76, *SE* = 5.91, $p = .016$). A significant main effect was found for blue saturation, $F(4,763) = 2.74$, $p = .03$, $\eta p^2 = .01$. Images in the general-threat categories had significantly less blue saturation than images in the neutral category (*Md* = 29.64, *SE* = 9.96, $p = .030$). A significant main effect was found for green saturation, $F(4, 763) = 4.19$, $p = .002$, $\eta p^2 = .02$. Images in the general-threat expressions category had significantly less green saturation than images in the neutral category (*Md* = 22.03, *SE* = 6.23, $p = .004$). A significant main effect was found for RMS contrast levels, $F(4,763) = 5.98$, $p < .001$, $\eta p^2 = .03$. Images in the facial expressions of pain category had significantly higher RMS contrast than the images included in musculoskeletal-pain (*Md* = 9.57, *SE* = 3.32, $p = .040$), health-threat (*Md* =13.77, *SE* = 3.32, $p < .001$) and neutral categories (*Md* = 10.89, *SE* = 2.49, $p < .001$).

Liossi C, *et al. BMJ Open* 2020; 10:e030607. doi: 10.1136/bmjopen-2019-030607

ATTENTIONAL BIAS MODIFICATION PROTOCOL

Table 3

Mean Low-Level values per Image Category

	Musculoskeletal-pain		Facial Expressions of Pain		General-threat		Health-threat		All neutral stimuli	
	M	*SD*	*M*	*SD*	*M*	*SD*	*M*	*SD*	*M*	*SD*
Complexity	20.03	7.17	15.69	3.10	20.14	6.36	19.30	10.78	18.62	10.92
Red Saturation	148.72	41.35	172.95	22.79	148.99	43.43	164.37	57.07	167.76	45.41
Green Saturation	140.39	41.39	153.81	27.56	132.80	41.82	154.92	58.08	154.84	48.60
Blue Saturation	132.53	43.55	144.86	31.64	119.90	48.00	149.09	61.87	149.54	85.49
Luminance	142.01	41.10	158.57	25.50	136.21	41.39	157.37	57.10	162.79	92.44
RMS contrast level	61.25	15.3	70.82	18.75	64.22	18.95	57.00	20.91	59.93	18.86

Analyses and results: Valence, arousal and pain intensity. A repeated-measures ANOVA was conducted comparing arousal ratings (Table 4) across the five image categories, revealing a significant main effect, $F(2,23) = 32.79$, $p < .001$, $\eta p^2 = .69$. Pairwise comparisons revealed that the neutral category had significantly lower arousal scores than facial expressions of pain (*Md* = 2.24, *SE* = 0.45, *p* = .002), musculoskeletal-pain (*Md* = 1.28, *SE* = 0.33, *p* = .014), general-threat (*Md* = 2.86, *SE* = 0.38, $p < .001$), and health-threat (*Md* = 1.91, *SE* = 0.34, *p* = .001) categories. The general-threat category was significantly more arousing than facial expressions of pain (*Md* = 0.62, *SE* = 0.15, *p* = .010), musculoskeletal-pain (*Md* = 1.59, *SE* = 0.14, $p < .001$), and health-threat categories (*Md* = 0.95, SE =0.15, $p < .001$). The musculoskeletal-pain category was also significantly less arousing than the facial expressions of pain (*Md* = 0.96, *SE* = 0.15, $p < .001$) and the health-threat (*Md* = 0.64, *SE* = 0.14, *p* = .005) categories.

ATTENTIONAL BIAS MODIFICATION PROTOCOL

A repeated-measures ANOVA conduced on valence scores (Table 4) across the five image conditions revealed a significant main effect, $F(2,29) = 64.52$, $p < .001$, $\eta p^2 = .82$. Pairwise comparisons showed the neutral category had significantly higher pleasantness scores than facial expressions of pain (*Md* = 1.56, *SE* = 0.15, *p* <. 001), musculoskeletal-pain (*Md* = 0.86, *SE* = 0.13, *p* < .001), general-threat (*Md* = 1.69, *SE* = 0.16, *p*<. 001) and health-threat (*Md* = 1.27, *SE* = 0.18, *p*< .001) categories. Images in the musculoskeletal-pain category were rated significantly more pleasant than the images included in the facial expressions of pain (*Md* = 0.69, *SE* = 0.07, *p* < .001), general-threat (*Md* = 0.83, *SE* = 0.08, *p* < .001) and health-threat (*Md* = 0.41, *SE* = 0.09, *p*= .003) categories. Images in the health-threat category had significantly higher pleasantness scores than images included in the general-threat category (*Md* = 0.42, *SE* = 0.07, *p* < .001).

An independent samples *t*-test was conducted comparing pain intensity scores (Table 4) between the images in the musculoskeletal pain and facial expressions of pain categories. There was a significant difference in the pain intensity scores of musculoskeletal-pain (*M* = 3.35, *SD* = 2.27) and facial expressions of pain (*M* = 5.15, *SD* = 2.13) categories; $t(245) = 6.520$, $p < .001$, $r = 0.38$. This result suggests images included in the facial expressions of pain category had significantly higher pain intensity ratings than those included in the musculoskeletal-pain category.

Liossi C, *et al. BMJ Open* 2020; 10:e030607. doi: 10.1136/bmjopen-2019-030607

ATTENTIONAL BIAS MODIFICATION PROTOCOL

Table 4

Mean Arousal, Valence and Pain Intensity Ratings per Image Category

	Arousal		Valence		Pain Intensity	
Image Category	*M*	*SD*	*M*	*SD*	*M*	*SD*
Musculoskeletal-pain	2.85	1.45	4.20	0.57	3.35	2.09
Facial expressions of pain	3.81	1.92	3.50	0.66	5.15	2.02
General-threat	4.43	1.69	3.37	0.62		
Health-threat	3.48	1.53	3.79	0.71		
Musculoskeletal-pain neutral counterparts	1.82	0.68	5.11	0.22		
Facial expressions of pain neutral counterparts	1.74	0.69	5.00	0.14		
General-threat neutral counterparts	1.82	0.68	5.08	0.17		
Health-threat neutral counterparts	1.75	0.54	5.04	0.24		
Neutral/Neutral	1.57	0.49	5.10	0.15		

Liossi C, *et al. BMJ Open* 2020; 10:e030607. doi: 10.1136/bmjopen-2019-030607

ATTENTIONAL BIAS MODIFICATION PROTOCOL

Comparison between Visual-Probe Sessions – Linguistic Stimuli

Differences in Arousal Scores between Threatening Word Categories across the Nine Linguistic Visual Probe Sessions

A 4 (word category; sensory-pain, affective-pain, general-threat, health-threat) x 9 (linguistic visual probe session; session 1, 3, 5, 7, 9, 11, 13, 15, 17) ANOVA was conducted to compare the arousal scores of the threatening word categories across the 9 linguistic visual probe task sessions. The results showed a significant main effect of session, $F(8, 120) = 8.22$, $p < .001$, $\eta_p^2 = .35$, and a significant word category x session interaction, $F(24, 360) = 3.05$, $p < .001$, $\eta_p^2 = .17$.

Post-hoc analyses across sessions. To explore the significant interaction, a series of one-way ANOVAs with 9 levels (Session: 1, 3, 5, 7, 9, 11, 13, 15, 17) were run for each threatening word category independently. These explored whether the arousal ratings of each threatening word category differed across the sessions. For the sensory-pain words, a main effect of session was found, $F(5,67) = 6.02$, $p < .001$, $\eta_p^2 = .29$. Pairwise comparisons revealed sensory words to be significantly more arousing in session 1 than session 9 (*Md* = 0.92, *SE* = 0.22, *p* = .03), and significantly more arousing in session 17 than session 9 (*Md* = 0.75, *SE* = 0.17, *p* = .02) and session 15 (*Md* = 0.59, *SE* = 0.11, *p* = .003).

For the affective-pain words, a main effect of session was found, $F(8,120) = 4.26$, $p < .001$, $\eta_p^2 = .22$. Pairwise comparisons revealed these words to be significantly more arousing in session 1 than in session 5 (*Md* = 0.57, *SE* = 0.12, *p* = .01) and session 13 (*Md* = 1.04, *SE* = 0.26, *p* = .05).

For the general-threat words, a main effect of session was found, $F(8, 120) = 4.91$, $p < .001$, $\eta_p^2 = .25$. Pairwise comparisons revealed these words to be significantly more arousing in session 1 than session 5 (*Md* = 0.65, *SE* = 0.13, *p* = .01), session 7 (*Md* = 0.88, *SE* = 0.17, *p* = .004) and session 15 (*Md* = 0.84, *SE* = 0.19, *p* = .02).

Liossi C, *et al. BMJ Open* 2020; 10:e030607. doi: 10.1136/bmjopen-2019-030607

ATTENTIONAL BIAS MODIFICATION PROTOCOL

For the health-threat words, a main effect of session was found, $F(8,120) = 4.08$, $p < .001$, $\eta_p^2 = .21$. Pairwise comparisons revealed these words to be significantly more arousing in session 1 than session 11 (*Md* = 1.05, *SE* = 0.18, *p* = .001).

Post-hoc analyses within sessions. To further explore the significant interaction, a series of one-way ANOVAs with 4 levels (word category; sensory-pain, affective-pain, general-threat and health-threat) were conducted for each session independently. These explored whether the arousal ratings of each threatening word category differed within each linguistic visual-probe session. No significant main effects of word category were found for session 1, $F(3,45) = 1.16$, $p = .20$, $\eta_p^2 = .10$, session 5, $F(3,45) = 2.34$, $p = .86$, $\eta_p^2 = .14$, session 13, $F(3,45) = 0.70$, $p = .06$, $\eta_p^2 = .05$, and session 17, $F(3,45) = 2.27$, $p = .09$, $\eta_p^2 = .13$.

A significant main effect of word category was found in session 3, $F(3,45) = 4.31$, $p = .01$, $\eta_p^2 = .22$. Pairwise comparisons revealed no significant differences between the four threat-related categories.

A significant main effect of word category was found in session 7, $F(3,45) = 3.75$, $p = .02$, $\eta_p^2 = .20$. Pairwise comparisons revealed no significant differences between the four threat-related categories.

A significant main effect of word category was found in session 9, $F(3,45) = 5.22$, $p = .004$, $\eta_p^2 = .26$. Pairwise comparisons revealed that sensory-pain words were significantly less arousing than affective-pain (*Md* = 0.84, *SE* = 0.21, *p* = .01), general-threat (*Md* = 0.77, *SE* = 0.23, *p* = .03) and health-threat categories (*Md* = 0.92, *SE* = 0.30, *p* = .05).

A significant main effect of word category was found in session 11, $F(3,45) = 4.45$, $p = .01$, $\eta_p^2 = .23$. Pairwise comparisons showed that the general-threat words were significantly more arousing than the health-threat words (*Md* = 0.61, *SE* = 0.19, *p* = .04).

A significant main effect of word category was found in session 15, $F(3,45) = 2.86$, $p = .05$, $\eta_p^2 = .16$. Pairwise comparisons revealed no significant differences between the four threat-related categories.

Liossi C, *et al. BMJ Open* 2020; 10:e030607. doi: 10.1136/bmjopen-2019-030607

ATTENTIONAL BIAS MODIFICATION PROTOCOL

Differences in Valence Scores between Threatening Word Categories across the nine Linguistic Visual Probe Sessions

A 4 (word category; sensory-pain, affective-pain, general-threat and health-threat) x 9 (linguistic visual probe sessions; 1, 3, 5, 7, 9, 11, 13, 15, and 17) ANOVA was conducted to compare the pleasantness scores of the threatening categories across the 9 linguistic visual probe task sessions. The results showed significant main effects of word category, $F(3,45) = 15.86$, $p < .001$, $\eta_p^2 = .51$, and session, $F(8,120) = 11.90$, $p < .001$, $\eta_p^2 = .44$, and a significant word category x session interaction, $F(24,360) = 2.57$, $p < .001$, $\eta_p^2 = .15$.

Post-hoc analyses across sessions. To explore the significant interaction, a series of one-way ANOVAS with 9 levels (Session; 1, 3, 5, 7, 9, 11, 13, 15, 17) were run for each word category independently. These explored whether the pleasantness ratings for each word category differed across the sessions. For the sensory-pain words, a main effect of session was found, $F(5,75) = 6.88$, $p< .001$, $\eta_p^2 = .31$. Pairwise comparisons revealed sensory words to be significantly less pleasant in session 1 than session 3 (*Md* = 0.49, *SE* = 0.12, $p =.04$), session 7 (*Md* = 0.59, *SE* = 0.11, $p = .003$) and session 9 (*Md* = 0.73, *SE* = 0.09, $p < .001$). Sensory-pain words were also significantly less pleasant in session 17 than session 5 (*Md* = 0.26, *SE* = 0.05, $p = .01$) and session 9 (*Md* = 0.49, *SE* = 0.09, $p = .003$).

For the affective-pain words, a main effect of session was found, $F(8,120) = 4.86$, $p< .001$, $\eta p^2 = .25$. Pairwise comparisons revealed these words to be significantly less pleasant in session 1 than session 5 (*Md* = 0.51, *SE* = 0.11, $p = .01$), session 7 (*Md* = 0.47, *SE* = 0.09, $p = .01$), session 13 (*Md* = 0.59, *SE* = 0.11, $p = .004$) and session 15 (*Md* = 0.44, *SE* = 0.11, $p = .04$).

For the general-threat words, a main effect of session was found, $F(8,120) = 9.64$, $p< .001$, $\eta p^2 = .39$. Pairwise comparisons revealed these words to be significantly less pleasant in session 1 than session 5 (*Md* = 0.63, *SE* = 0.09, $p < .001$), session 7 (*Md* = 0.65, *SE* = 0.11, $p = .001$), session 9 (*Md* = 0.44, *SE* = 0.07, $p < .001$), session 13 (*Md* = 0.33, *SE* = 0.06, $p = .001$) and session 15 (*Md* = 0.65, *SE* = 0.12, $p = .002$). Significantly less pleasant words were also found in

Liossi C, *et al. BMJ Open* 2020; 10:e030607. doi: 10.1136/bmjopen-2019-030607

session 3 than session 5 (*Md* = 0.37, *SE* = 0.09, $p = .03$) and session 7 (*Md* = 0.39, *SE* = 0.08, $p = .01$). Session 17 included significantly less pleasant words than session 7 (*Md* = 0.41, *SE* = 0.10, $p = .03$).

For the health-threat words, a main effect of session was found, $F(4,58) = 2.90$, $p = .03$, $\eta_p^2 = .16$. Pairwise comparisons revealed these words to be significantly less pleasant in session 1 than session 11 (*Md* = 0.57, *SE* = 0.14, $p = .03$).

Post-hoc analyses within sessions. To further explore the significant interaction, one-way ANOVAs with 4 levels (word category; sensory-pain, affective-pain, general-threat and health-threat) were conducted for each session independently. These explored whether pleasantness ratings for each threatening word category differed within each session. For session 1, a significant main effect was found, $F(3,45) = 4.84$, $p = .01$, $\eta_p^2 = .24$. Pairwise comparisons revealed that sensory-pain words were significantly more pleasant than affective words (*Md* = 0.63, *SE* = 0.16, $p = .01$).

For session 3, a significant main effect was found, $F(3,45) = 12.71$, $p < .001$, $\eta_p^2 = .46$. Pairwise comparisons showed that sensory-pain words were significantly more pleasant than affective-pain (*Md* = 0.73, *SE* = .15, $p < .001$), general-threat (*Md* = 0.79, *SE* = 0.13, $p < .001$) and health-threat words (*Md* = 0.56, *SE* = 0.15, $p = .01$).

For session 5, a significant main effect was found, $F(3,45) = 6.45$, $p < .001$, $\eta_p^2 = .30$. Pairwise comparisons revealed that sensory-pain words were significantly more pleasant than the affective-pain words (*Md* = 0.62, *SE* = 0.15, $p = .01$).

For session 7, a significant main effect was found, $F(3,45) = 10.19$, $p < .001$, $\eta_p^2 = .41$. Pairwise comparisons revealed that sensory-pain words were significantly more pleasant than the affective-pain words (*Md* = 0.17, *SE* = 0.13, $p < .001$), the general-threat words (*Md* = 0.51, *SE* = 0.09, $p < .001$) and the health-threat words (*Md* = 0.55, *SE* = 0.16, $p = .02$).

For session 9, a significant main effect was found, $F(2,31) = 13.67$, $p < .001$, $\eta_p^2 = .48$. Pairwise comparisons revealed that the sensory-pain words were significantly more pleasant than

Liossi C, *et al. BMJ Open* 2020; 10:e030607. doi: 10.1136/bmjopen-2019-030607

ATTENTIONAL BIAS MODIFICATION PROTOCOL

the affective-pain words (*Md* = 0.98, *SE*= 0.16, $p < .001$), the general-threat words (*Md* = 0.87, *SE* = 0.15, $p < .001$) and the health-threat words (*Md* = 0.92, *SE* = 0.21, $p < .001$).

For session 11, a significant main effect was found, $F(3,45) = 11.63$, $p < .001$, $\eta_p^2 = .44$. Pairwise comparisons revealed that the sensory-pain words were significantly more pleasant than the affective-pain (*Md* = 0.69, *SE* = 0.13, $p < .001$) and the general-threat words (*Md* = 0.70, *SE* = 0.17, $p < .001$). Also, the affective-pain words were significantly less pleasant than the health-threat words (*Md* = 9.39, *SE* = 0.13, $p = .04$).

For session 13, a significant main effect was found, $F(3,45) = 4.43$, $p = .01$, $\eta_p^2 = .23$. Pairwise comparisons revealed that the sensory-pain words were significantly more pleasant than the general-threat words (*Md* = 0.56, *SE* = 0.12, $p < .001$).

For session 15, a significant main effect was found, $F(3,45) = 8.40$, $p < .001$, $\eta_p^2 = .36$. Pairwise comparisons showed that the sensory-pain words were significantly more pleasant than the words included in the affective (*Md* = 0.61, *SE* = 0.12, $p < .001$) and the health-threat words (*Md* = 0.71, *SE* = 0.19, $p = .01$).

For session 17, a significant main effect was found, $F(3,45) = 6.78$, $p<.001$, $\eta_p^2 = 0.36$. $_p{}^2$ = 0.31. Pairwise comparisons revealed that the sensory-pain words were significantly more pleasant than affective-pain (*Md* = 0.56, *SE* = 0.15, $p = .01$) and the general-threat words (*Md* = 0.57, *SE* = 0.15, $p = .01$).

Comparison between Visual-Probe Sessions – Pictorial Stimuli

Differences in Arousal Scores between Threatening Image Categories across the eight Pictorial Visual Probe Sessions

A 4 (image category; musculoskeletal-pain, facial expressions of pain, general-threat, health-threat) x 8 (pictorial visual probe sessions; 2, 4, 6, 8, 10, 12, 14, 16) ANOVA was conducted to compare the arousal scores of the threatening image categories across the 8 pictorial visual probe task sessions. The results showed a significant main effect of image category, $F(3,45) = 33.52$, *p*

Liossi C, *et al. BMJ Open* 2020; 10:e030607. doi: 10.1136/bmjopen-2019-030607

< .001, η_p^2 = .69, and a significant image category x session interaction, $F(7,98) = 9.84$, $p < .001$, η_p^2 = .40.

Post-hoc analyses within image categories. To explore the significant interaction, a series of one-way ANOVAs with 8 levels (Session; 2, 4, 6, 8, 10, 12, 14, 16) were run for each threatening image category independently. These explored whether the arousal ratings of each threat-related image category differed across the sessions. For the images in the musculoskeletal-pain category, no main effect of session was found, $F(2,31) = 2.49$, $p = .10$, $\eta_p^2 = .14$. For the images in the facial expressions of pain category, no main effect of session was found, $F(3,46) = 0.77$, $p = .50$, $\eta_p^2 = .05$.

For the images in the general-threat category, a main effect of session was found, $F(7, 105) = 7.40$, $p < .001$, $\eta_p^2 = .33$. Pairwise comparisons revealed these images to be significantly less arousing in session 4 than session 6 (*Md* = 0.70, *SE* = 0.17, *p* = .023), session 12 (*Md* = 0.73, *SE* = 0.19, *p* = .04) and session 16 (*Md* = 0.82, *SE* = 0.21, *p* = .04). These images were also found to be significantly less arousing in session 14 than session 6 (*Md* = 0.93, *SE* = 0.19, *p* = .006), session 12 (*Md* = 0.96, *SE* = 0.17, *p* = .001) and session 16 (*Md* = 1,05, *SE* = 0.17, *p* = .001).

For the images in the health-threat category, a main effect of session was found, $F(7, 105) = 13.10$, $p < .001$, $\eta_p^2 = .47$. Pairwise comparisons revealed these images to be significantly more arousing in session 10 than session 8 (*Md* = 1.15, *SE* = 0.20, *p* = .001) and session 12 (*Md* = 0.88, *SE* = 0.20, *p* = .02). Health-threat images were also found to be significantly less arousing in session 16 than session 2 (*Md* = 0.97, *SE* = 0.17, *p* = .001), session 4 (*Md* = 1.34, *SE* = 0.26, *p* = .004), session 6 (*Md* = 1.32, *SE* = 0.29, *p* = .009), session 10 (*Md* = 1.91, *SE* = 0.28, *p* < .001), session 12 (*Md* = 1.03, *SE* = 0.26, *p* = .04) and session 14 (*Md* = 1.45, *SE* = 0.9, *p* < .001).

Post-hoc analyses within sessions. To further explore the significant interaction, a series of one-way ANOVAs with 4 levels (image category; musculoskeletal-pain, facial expressions of pain, general-threat and health-threat) were conducted for each session independently. These

explored whether the arousal ratings of each threatening image category differed within each pictorial visual-probe session.

A significant main effect of image category was found in session 2, $F(3,45) = 23.27$, $p < .001$, $\eta_p^2 = .61$. Pairwise comparisons revealed that the images in the facial expressions of pain category were more arousing than those included in the musculoskeletal-pain category (*Md* = 0.98, *SE* = 0.27, $p = .014$). The images in the general-threat category were also found to be significantly more arousing than those included in the musculoskeletal-pain (*Md* = 2.05, *SE* = 0.32, $p < .001$), facial expressions of pain (*Md* = 1.07, *SE* = 0.19, $p < .001$) and health-threat categories (*Md* = 1.34, *SE* = 0.24, $p < .001$).

A significant main effect of image category was found in session 4, $F(3,45) = 8.27$, $p < .001$, $\eta_p^2 = .36$. Pairwise comparisons revealed that the images in the musculoskeletal-pain category were significantly less arousing than the images in the facial expressions of pain (*Md* = 0.76, *SE* = 0.16, $p = .001$), health-threat (*Md* = 1.10, *SE* = 0.23, $p = .002$) and general-threat categories (*Md* = 0.70, *SE* = 0.22, $p = .04$).

A significant main effect of image category was found in session 6, $F(3,45) = 26.77$, $p < .001$, $\eta_p^2 = .64$. Pairwise comparisons revealed that the images in the musculoskeletal-pain category were significantly less arousing than the images included in the facial expressions of pain (*Md* = 1.17, *SE* = 0.25, $p = .002$), general-threat (*Md* = 2.06, *SE* = 0.23, $p < .001$) and health-threat categories (*Md* = 0.97, *SE* = 0.25, $p = .009$). The images in the general-threat category were significantly more arousing than those in the facial expressions of pain (*Md* = 0.89, *SE* = 0.21, $p = .003$) and health-threat categories (*Md* = 1.09, *SE* = 0.21, $p = .001$).

A significant main effect of image category was also found in session 8, $F(2,32) = 10.56$, $p < .001$, $\eta_p^2 = .41$. Pairwise comparisons revealed that the images in the musculoskeletal-pain category were significantly less arousing than those included in the facial expressions of pain (*Md* = 0.89, *SE* = 0.22, $p = .006$) and general-threat categories (*Md* = 1.13, *SE* = 0.25, $p = .003$).

ATTENTIONAL BIAS MODIFICATION PROTOCOL

The images in general-threat categories were significantly more arousing that those included in the health-threat category (*Md* = 1.00, *SE* = 0.20, *p* = .001).

A significant main effect of image category was found in session 10, *F*(3,45) = 17.37, $p <$ .001, η_p^2 = .54. Pairwise comparisons revealed that the images in the musculoskeletal-pain category were significantly less arousing than those included in the general-threat (*Md* = 0.92, *SE* = 0.17, $p <$ .001) and health-threat categories (*Md* = 1.23, *SE* = 0.20, $p <$ .001). The images in the facial expressions of pain category were also significantly less arousing than those included in the health-threat category (*Md* = 0.71, *SE* = 0.18, *p* = .009).

A significant main effect of image category was found in session 12, *F*(3,45) = 18.40, $p <$ .001, η_p^2 = .55. Pairwise comparisons revealed that the images in the musculoskeletal-pain category were significantly less arousing that those included in the facial expressions of pain (*Md* = 1.61, *SE* = 0.18, *p* = .022) and general-threat categories (*Md* = 1.64, *SE* = 0.19, $p <$ .001). The images included in the general-threat category were significantly more arousing than the images included in the facial expressions of pain (*Md* = 1.03, *SE* = 0.24, *p* = .001) and health-threat categories (*Md* = 1.03, *SE* = 0.26, $p <$ .001).

A significant main effect of image category was found in session 14, *F*(3,45) = 14.72, $p <$ .001, η_p^2 = .50. Pairwise comparisons revealed than the images in the musculoskeletal-pain category were significantly less arousing than those included in facial expressions of pain (*Md* = 1.39, *SE* = 0.30, *p* = .002), general-threat (*Md* = 1.41, *SE* = 0.25, $p <$ .001) and health-threat categories (*Md* = 1.35, *SE* = 0.20, $p <$ .001).

A significant main effect of image category was found in session 16, *F*(2,28) = 38.74, $p <$ .001, η_p^2 = .72. Pairwise comparisons revealed that the images in the musculoskeletal-pain category were significantly less arousing than the images included in the facial expressions of pain (*Md* = 1.38, *SE* = 0.29, *p* = .001) and general-threat categories (*Md* = 2.37, *SE* = 0.27, *p* = .002). Images included in the facial expression of pain category were also found to be significantly less arousing than those included in the general-threat category (*Md* = 0.98, *SE* =

0.21, $p = .002$). Images in the health-threat category were significantly less arousing than the images included in the facial expression of pain (*Md* = 1.57, *SE* = 0.36, $p = .004$) and general-threat categories (*Md* = 2.56, *SE* = 0.29, $p < .001$).

Differences in Valence Scores between Threatening Pictorial Categories across the eight Pictorial Visual Probe Sessions

A 4 (image category; musculoskeletal-pain, facial expressions of pain, general-threat and health-threat) x 8 (pictorial visual probe sessions; 2, 4, 6, 8, 10, 12, 14, 16) ANOVA was conducted to compare the pleasantness scores of the threatening categories across the 8 pictorial visual probe task sessions. The results showed a significant main effect of image category, $F(3,45) = 41.09$, $p < .001$, $\eta_p^2 = .73$, and a significant image category x session interaction, $F(7,102) = 9.33$, $p < .001$, $\eta_p^2 = .38$.

Post-hoc analyses within image categories. To explore the significant interaction, a series of one-way ANOVAS with 8 levels (Session; 2, 4, 6, 8, 10, 12, 14, 16) were run for each image category independently. These explored whether the pleasantness ratings for each image category differed across the sessions.

For the images included in the musculoskeletal-pain category, a main effect of session was found, $F(2,32) = 3.70$, $p = .033$, $\eta_p^2 = .20$. Pairwise comparisons revealed these images to be significantly more pleasant in session 2 than session 4 (*Md* = 0.54, *SE* = 0.11, $p = .007$).

For the images included in the facial expressions of pain category, a main effect of session was found, $F(7,105) = 3.44$, $p = .002$, $\eta_p^2 = .19$. Pairwise comparisons revealed these images to be significantly more pleasant in session 10 than session 6 (*Md* = 0.51, *SE* = 0.13, $p = .03$) and session 16 (*Md* = 0.43, *SE* = 0.09, $p = .005$).

For the images included in the general-threat category, a main effect of session was found, $F(7,105) = 6.84$, $p < .001$, $\eta_p^2 = .31$. Pairwise comparisons revealed these images to be significantly more pleasant in session 10 than session 6 (*Md* = 0.61, *SE* = 0.13, $p = .009$), session 12 (*Md* = 0.56, *SE* = 0.14, $p = .05$) and session 16 (*Md* = 0.68, *SE* = 0.14, $p = .007$). General-

threat images were significantly more pleasant in session 14 than session 6 (*Md* = 0.60, *SE* = 0.14, p = .019), session 12 (*Md* = 0.55, *SE* = 0.12, p = .013) and session 16 (*Md* = 0.67, *SE* = 0.11, p = .001). Images in this category were also significantly more pleasant in session 4 than session 6 (*Md* = 0.45, *SE* = 0.11, p = .04) and session 16 (*Md* = 0.52, *SE* = 0.11, p = .007).

For the images included in the health-threat category, a main effect of session was found, $F(7,105) = 9.87$, $p < .001$, $\eta_p^2 = .40$. Pairwise comparisons revealed these images to be significantly more pleasant in session 8 than session 10 (*Md* = 0.73, *SE* = 0.10, p = .005). Images in this category were significantly more pleasant in session 16 than session 2 (*Md* = 0.54, *SE* = 0.11, p = .004), session 4 (*Md* = 0.62, *SE* = 0.12, p = .003), session 6 (*Md* = 0.52, *SE* = 0.12, p = .02), session 10 (*Md* = 1.00, *SE* = 0.10, $p < .001$), session 12 (*Md* = 0.56, *SE* = 0.12, p = .008) and session 14 (*Md* = 0.80, *SE* = 0.10, $p < .001$).

Post-hoc analyses within sessions. To further explore the significant interaction, one-way ANOVAs with 4 levels (image category; musculoskeletal-pain, facial expressions of pain, general-threat and health-threat) were conducted for each session independently. These explored whether pleasantness ratings for each image category differed within each session.

For session 2, a significant main effect was found, $F(3,45) = 13.36$, $p < .001$, $\eta_p^2 = .47$. Pairwise comparisons revealed that images in the musculoskeletal-pain category were significantly more pleasant than images included in the facial expressions of pain (*Md* = 1.00, *SE* = 0.18, $p < .001$), general-threat (*Md* = 0.98, *SE* = 0.20, p = .001) and health-threat categories (*Md* = 0.72, *SE* = 0.21, p = .024).

For session 4, a significant main effect was found, $F(3,45) = 7.09$, $p = .001$, $\eta_p^2 = .32$. Pairwise comparisons showed that the images in the musculoskeletal-pain category were significantly more pleasant than the facial expressions of pain (*Md* = 0.52, *SE* = 0.10, p = .001) and general-threat categories (*Md* = 0.48, *SE* = 0.12, p = .005).

For session 6, a significant main effect was found, $F(3,45) = 32.12$, $p < .001$, $\eta_p^2 = .68$. Pairwise comparisons revealed that images in the musculoskeletal-pain category were

Liossi C, *et al. BMJ Open* 2020; 10:e030607. doi: 10.1136/bmjopen-2019-030607

significantly more pleasant than the images included in the facial expressions of pain (*Md* = 1.05, *SE* = 0.14, $p < .001$) and general-threat categories (*Md* = 1.27, *SE* = 0.13, $p < .001$). The images in the health-threat category were also significantly more pleasant than the images included in the facial expressions of pain (*Md* = 0.56, *SE* = 0.14, $p = .01$) and general-threat categories (*Md* = 0.77, *SE* = 0.13, $p < .001$).

For session 8, a significant main effect was found, $F(3,45) = 11.73$, $p < .001$, $\eta_p^2 = .44$. Pairwise comparisons revealed that images in the musculoskeletal-pain category were significantly more pleasant than those included in the facial expressions of pain (*Md* = 0.62, *SE* = 0.07, $p < .001$) and general-threat categories (*Md* = 0.57, *SE* = 0.14, $p = .004$). The images included in the health-threat category were also significantly more pleasant than those included in the facial expressions of pain (*Md* = 0.54, *SE* = 0.15, $p = .019$) and general-threat categories (*Md* = 0.49, *SE* = 0.14, $p = .016$).

For session 10, a significant main effect was found, $F(3,45) = 12.59$, $p < .001$, $\eta_p^2 = .46$. Pairwise comparisons revealed that images in the health-threat category were significantly less pleasant than those included in the musculoskeletal-pain (*Md* = 0.74, *SE* = 0.12, $p < .001$) and facial expressions of pain categories (*Md* = 0.44, *SE* = 0.12, $p = .017$).

For session 12, a significant main effect was found, $F(3,45) = 14.90$, $p < .001$, $\eta_p^2 = .50$. Pairwise comparisons revealed that images in the musculoskeletal-pain category were significantly more pleasant than images included in the facial expressions of pain (*Md* = 0.45, *SE* = 0.08, $p < .001$) and general-threat categories (*Md* = 0.88, *SE* = 0.16, $p < .001$). Images in the health-threat category were also significantly more pleasant than images included in the general health category (*Md* = 0.68, *SE* = 0.16, $p = .004$).

For session 14, a significant main effect was found, $F(3,45) = 13.30$, $p < .001$, $\eta_p^2 = .47$. Pairwise comparisons revealed that images in the musculoskeletal-pain category were significantly more pleasant than images included in the facial expressions of pain (*Md* = 0.71, *SE*

Liossi C, *et al. BMJ Open* 2020; 10:e030607. doi: 10.1136/bmjopen-2019-030607

= 0.16, *p* = .003), general-threat (*Md* = 0.71, *SE* = 0.15, *p* = .001) and health-threat categories (*Md* = 0.82, *SE* = 0.12, *p* < .001).

For session 16, a significant main effect was found, $F(3,45) = 83.05$, $p < .001$, $\eta_p^2 = .85$. Pairwise comparisons revealed that images in the facial expressions of pain category were significantly less pleasant than musculoskeletal-pain (*Md* = 0.97, *SE* = 0.10, *p* < .001) and health-threat categories (*Md* = 0.99, *SE* = 0.12, *p* < .001). Images in the general-threat category were also significantly less pleasant than those included in the musculoskeletal-pain (*Md* = 1.34, *SE* = 0.11, *p* < .001), facial expressions of pain (*Md* = 0.37, *SE* = 0.09, *p* = .007) and health-threat categories (*Md* = 1.36, *SE* = 0.09, *p* < .001).

Differences in Pain Intensity Scores of Threatening Pain Categories across the eight Pictorial Visual Probe Sessions

A 2 (image category; musculoskeletal-pain, facial expressions of pain) x 8 (pictorial visual probe sessions; session 2, 4, 6, 8, 10, 12, 14, 16) ANOVA was conducted to compare the pain intensity scores of the threatening pain categories across the 8 pictorial visual probe task sessions. The results showed significant main effects of image category, $F(1,15) = 45.40$, $p < .001$, $\eta_p^2 = .75$, and session, $F(2,29) = 3.81$, $p = .04$, $\eta_p^2 = .20$, and a significant image category x session interaction, $F(3,44) = 7.44$, $p < .001$, $\eta_p^2 = .33$.

Post-hoc analyses within image categories. To explore the significant interaction, one-way ANOVAs with 8 levels (Session; 2, 4, 6, 8, 10, 12, 14, 16) were run for each pain-related image category independently. These explored whether the pain intensity ratings of each pain-related image category differed across the sessions. For the musculoskeletal-pain category, a main effect of session was found, $F(2,25) = 4.70$, $p = .023$, $\eta p^2 = .24$. Pairwise comparisons revealed that the images included in session 14 had significantly less pain intensity scores than the images included in session 8 (*Md* = 1.06, *SE* = 0.24, *p* = .014), session 10 (*Md* = 0.95, *SE* = 0.19, *p* = .004) and session 12 (*Md* = 1.19, *SE* = 0.15, *p* < .001). Images in session 4 had significantly higher pain intensity scores than the images included in session 6 (*Md* = 0.82, *SE* =

0.21, p = .05). The images included in session 12 had also significantly higher pain intensity scores than the images included in session 16 (*Md* = 1.03, *SE* = 0.17, p = .001).

For the facial expression of pain category, a main effect of session was found, $F(3,39)$ = 4.65, p = .01, ηp^2 = .24. Pairwise comparisons revealed that the images included in session 10 had significantly less pain intensity scores that the images included in session 1 (*Md* = 1.07, *SE* = 0.25, p = .02) and session 6 (*Md* = 1.38, *SE* = 0.31, p = .013).

Differences in low-level features of pictorial categories across the eight visual probe sessions

A series of one-way ANOVAs were conducted independently to compare the scores of complexity, red, green and blue saturation, luminance and RMS contrast level of the threat related categories (i.e., musculoskeletal-pain, facial expression of pain, general-threat and health-threat) across the 8 pictorial visual probe task sessions (i.e., Session 2, 4, 6, 8, 10, 12, 14, 16).

For complexity, the analysis revealed a significant main effect of image category, $F(3, 224)$ = 5.15, p = .002, η_p^2 = .06. There was no significant main effect of session, $F(7, 224)$ = 1.22, p = .30, and no significant image category x session interaction, $F(21, 224)$ = 0.82, p =.70.

For red saturation, the analysis revealed a significant main effect of image category, $F(3, 224)$ = 5.05, p = .002, η_p^2 = .06. There was no significant main effect of session , $F(7,224)$ = 1.03, p = .41, and no significant image category x session interaction, $F(21,224)$ = 1.196, p =.26.

For green saturation, the analysis revealed a significant main effect of image category, $F(3, 224)$ = 4.08, p = .008, η_p^2 = .08. There was no significant main effect of session, $F(7, 224)$ = 1.65, p = .12, and no and no significant image category x session interaction, $F(21,224)$ = 1.41, p =.12.

For blue saturation, the analysis revealed a significant main effect of image category, $F(3, 224)$ = 5.288, p = .002, η_p^2 = 0.07, and a significant image category x session interaction, $F(21, 224)$ = 1.672, p = .04, η_p^2 = 0.14. There was no significant main effect of session, $F(7, 224)$ = 1.750, p = .10.

ATTENTIONAL BIAS MODIFICATION PROTOCOL

For luminance, the analysis revealed a significant main effect of image category, $F(3, 224) = 4.548$, $p = .004$, $\eta_p^2 = 0.06$. The analysis revealed no significant main effect of session, $F(7,224) = 1.504$, $p = .17$, and no significant image category x session interaction, $F(21, 224) = 1.361$, $p = .14$.

For RMS contrast level, the analysis revealed a significant main effect of image category, $F(3, 224) = 6.84$, $p < .001$, $\eta_p^2 = .08$, and a significant image category x session interaction, $F(21,224) = 1.98$, $p = .008$. There was no significant main effect of session, $F(7,224) = 1.68$, $p = .12$,

Post-hoc analyses within image categories. To explore the significant interaction for blue saturation, a series of one-way ANOVAS with 8 levels (Session; 2, 4, 6, 8, 10, 12, 14, 16) were run for each image category independently. These explored whether the blue saturation scores for each image category differed across the sessions. No significant main effect of session was found for the musculoskeletal-pain category, $F(7,56) = 0.82$, $p = .57$, or the health-threat category, $F(7,56) = 1.59$, $p = .16$.

For the facial expressions of pain category, a significant interaction for session number was found, $F(7,56) = 2.94$, $p = .01$, $\eta_p^2 = .27$. Pairwise comparisons did not reveal any significant differences however..

For the general-threat category, a significant main effect of session number was found, $F(7,56) = 2.20$, $p = .05$, $\eta_p^2 = .22$. Pairwise comparisons did not reveal any significant differences however.

To explore the significant interaction for RMS contrast level, a series of one-way ANOVAS with 8 levels (Session; 2, 4, 6, 8, 10, 12, 14, 16) were run for each image category independently. These explored whether the RMS contrast level scores for each image category differed across the sessions. No significant main effect of session was found for the musculoskeletal-pain category, $F(7,56) = 0.59$, $p = .76$, or the health-threat category, $F(7,56) = 0.16$, $p = .99$

Liossi C, *et al. BMJ Open* 2020; 10:e030607. doi: 10.1136/bmjopen-2019-030607

ATTENTIONAL BIAS MODIFICATION PROTOCOL

For the facial expressions of pain category, a significant interaction for session number was found, *F*(7,56) = 5.127, $p < .001$, $\eta_p^2 = 0.39$. Pairwise comparisons revealed that the images in session 6 had significantly less RMS contrast than those included in session 4 (*Md* = 36.02, *SE* = 7.76, *p* = .001), session 10 (*Md* = 30.81, *SE* = 7.76, *p* = .006), session 14 (*Md* = 25.96, *SE* = 7.76, *p* = .04) and session 16 (*Md* = 37.09, *SE* = 776, $p < .001$).

For the general-threat category, a significant interaction for session number was found, *F*(7,56) = 3.252, *p* = .006, $\eta_p^2 = 0.29$. Pairwise comparisons did not reveal any significant differences however.

Post-hoc analyses within sessions. To further explore the significant interaction for blue saturation, eight one-way ANOVAs with 4 levels (image category; musculoskeletal-pain, facial expressions of pain, general-threat and health-threat) were conducted for each session independently. These explored whether blue saturation scores between the five image categories differed within each session (i.e., session 2, 4, 6, 8, 10, 12, 14, 16).

No significant main effects were found for session 2, *F*(3,28) = 1.49, *p* = .24, session 4, *F*(3,28) = 0.18, *p* = .91, session 6, *F*(3,28) = 1.37, *p* = .27, session 8, *F*(3,28) = 1.127, *p* = .36, and session 14, *F*(3,28) = 0.508, *p* = .68.

For session 10, there was a significant main effect of image category, *F*(3,28) = 5.56, *p* = .004, $\eta_p^2 = .37$. Pairwise comparisons revealed that the images included in the facial expression of pain category had significantly higher blue saturation than the images included in the general-threat category (*Md* = 70.64, *SE* = 18.03, *p* = .003).

For session 12, there was a significant main effect of image category, *F*(3,28) = 3.55, *p* = .027, $\eta_p^2 = .28$. Pairwise comparisons revealed that the images included in the musculoskeletal-pain category had significantly higher blue saturation than the images included in the general-threat category (*Md* = 62.46, *SE* = 21.76, *p* = .046).

For session 16, there was a significant main effect of image category, *F*(3,28) = 6.99, *p* = .001, $\eta_p^2 = .43$. Pairwise comparisons revealed that the images included in the health-threat

categories had significantly higher blue saturation than the images included in the musculoskeletal-pain (*Md* = 74.57, *SE* = 20.09, *p* = .005) and general-threat categories (*Md* = 83.89, *SE* = 20.09, *p* = .002)

To further explore the significant interaction for RMS contrast level, eight one-way ANOVAs with 4 levels (image category; musculoskeletal-pain, facial expressions of pain, general-threat and health-threat) were conducted for each session independently. These explored whether the scores of RMS contrast level between the five image categories differed within each session (i.e., session 2, 4, 6, 8, 10, 12, 14, 16).

No significant main effects were found for session 2, *F*(3,28) = 0.720, *p* = .55, session 8, *F*(3,28) = 1.56, *p* = .22, session 12, *F*(3,28) = 0.62, *p* = .61, and session 14, *F*(3,28) = 1.76, *p* = .18.

For session 4, there was a significant main effect of image category, *F*(3,28) = 5.69, *p* = .004, η_p^2 = .38. Pairwise comparisons revealed that the images included in the facial expression of pain category had significantly higher RMS contrast than the images included in the health-threat category (*Md* = 33.44, *SE* = 8.27, *p* = .002).

For session 6, there was a significant main effect of image category, *F*(3,28) = 4.00, *p* = .02, η_p^2 = .30. Pairwise comparisons revealed that the images included in the musculoskeletal-pain category had significantly higher RMS contrast than those included in the facial expressions of pain category (*Md* = 17.69, *SE* = 5.95, *p* = .04).

For session 10, there was a significant main effect of image category, *F*(3,28) = 6.02, *p* = .003, η_p^2 = .39. Pairwise comparisons revealed that the images included in the facial expressions of pain category had significantly higher RMS contrast than those included in the general-threat (*Md* = 30.37, *SE* = 7.35, *p* = .002) and health-threat categories (*Md* = 21.42, *SE* = 7.35, *p* = .04).

For session 16, there was a significant main effect of image category, *F*(3,28) = 3.18, *p* = .04, η_p^2 = .25. Pairwise comparisons revealed that the images included in the facial expressions

of pain category had significantly higher RMS contrast level than those included in the health-threat category (*Md* = 29.32, *SE* = 10.24, *p* = .05).

ATTENTIONAL BIAS MODIFICATION PROTOCOL

References

1. Kučera H, Francis N. *Computational analysis of present-day American English*. Providence: Brown University Press, 1967.
2. AP Y. The Nature of Recollection and Familiarity: A Review of 30 Years of Research. Journal of Memory and Language 2002;**46**(3):441-517 doi: 10.1006/jmla.2002.2864.
3. Balota DA, Chumbley JI. Are lexical decisions a good measure of lexical access? The role of word frequency in the neglected decision stage. Journal of Experimental Psychology: Human perception and performance 1984;**10**(3):340-57.
4. Sharpe L, Ianiello, M., Dear, B. F, Nicholson Perry, K., Refshauge, K., Nicholas, M. K. Is there a potential role for attention bias modification in pain patients? Results of 2 randomised, controlled trials. Pain 2012;**153**(3):722-31.
5. Schoth D, Liossi C. Specificity and time-course of attentional bias in chronic headache: a visual-probe investigation. Clinical Journal of Pain 2013;**29**(7):583-90 doi: 10.1097/AJP.0b013e31826b4849.
6. Carleton R, Richter A, Asmundson G. Attention Modification in Persons with Fibromyalgia: A Double Blind, Randomized Clinical Trial. Cognitive Behaviour Therapy 2011;**40**(4):279-90.
7. Macleod C. Cognitive bias modification procedures in the management of mental disorders. Curr Opin Psychiatry 2012;**25**(2):114-20 doi: 10.1097/YCO.0b013e32834fda4a.
8. MacLeod C, Mathews A, Tata P. Attentional bias in emotional disorders. Journal of Abnormal Psychology 1986;**95**(1):15-20 doi: 10.1037/0021-843X.95.1.15.
9. Melzack R. The McGill Pain Questionnaire: major properties and scoring methods. Pain 1975;**1**(3):277-99.
10. Wilson MD. The MRC Psycholinguistic Database: Machine Readable Dictionary, Version 2. Behavioural Research Methods, Instruments and Computers 1988;**20**(1):6-11.

ATTENTIONAL BIAS MODIFICATION PROTOCOL

11. MRC Psycholinguistic Database: Machine Usable Dictionary. Version 2.00.: MRC Psycholinguistic Database, 1997.

12. Lane RD, Chua PM, Dolan RJ. Common effects of emotional valence, arousal and attention on neural activation during visual processing of pictures. Neuropsychologia 1999;**37**(9):989-97 doi: 10.1016/S0028-3932(99)00017-2.

13. Niedenthal PM, Kitayama S. *The heart's eye: Emotional influences in perception and attention*. London: Academic Press, 2013.

14. Bradley MM, Lang PJ. Measuring Emotion: The Self-Assessment Manikin and the Semantic Differential. J Behav Therap & Exp Psychiat 1994;**25**(1):49-59.

15. Liossi C, Schoth D. Attention toward interpersonal stimuli in individuals with and without chronic daily headache. Journal of Neurology and Neurosurgery 2016;**3**(3):1-7.

16. Schoth DE, Liossi C. Attentional bias toward pictorial representations of pain in individuals with chronic headache. Clin J Pain 2010;**26**(3):244-50 doi: 10.1097/AJP.0b013e3181bed0f9.

17. Craig KD. The facial expression of pain. Better than a thousand words? APS Journal 1992;**1**(3):153-62.

18. Prkachin KM, Solomon PE. The structure, reliability and validity of pain expression: evidence from patients with shoulder pain. Pain 2008;**139**(2):267-74 doi: 10.1016/j.pain.2008.04.010.

19. Schoth DE, Georgallis T, Liossi C. Attentional bias modification in people with chronic pain: a proof of concept study. Cognitive Behaviour Therapy 2013;**42**(3):233-43.

20. Bradley MM, Lang PJ. The International Affective Picture System (IAPS) in the study of emotion and attention. In: Allen CaJJB, ed. Handbook of Emotion Elicitation and Assessment: Oxford University Press, 2007.

21. Leeuw M, Goossens ME, van Breukelen GJ, et al. Measuring perceived harmfulness of physical activities in patients with chronic low back pain: the Photograph Series of Daily

Activities--short electronic version. J Pain 2007;**8**(11):840-9 doi: 10.1016/j.jpain.2007.05.013.

22. Tottenham N, Tanaka JW, Leon AC, et al. The NimStim set of facial expressions: judgments from untrained research participants. Psychiatry Res 2009;**168**(3):242-9 doi: 10.1016/j.psychres.2008.05.006.

23. Nummenmaa L, Hyönä J, Calvo MG, & Nummenmaa, L. Eye movement assessment of selective attentional capture by emotional pictures. Emotion 2006;**6**(2):257-68 doi: 10.1037/1528-3542.6.2.257.

24. Borji A, Sihite DN, Itti L. What stands out in a scene? A study of human explicit saliency judgment. Vision research 2013;**91**:62-77 doi: 10.1016/j.visres.2013.07.016.

25. Henderson JM. Human gaze control during real-world scene perception. Trends in Cognitive Sciences 2003;**7**(11):498-504 doi: 0.1016/j.tics.2003.09.006.

26. Torralba A, Oliva, A., Castelhano, M. S., & Henderson, J. M. Contextual guidance of eye movements and attention in real-world scenes: The role of global features in object search. Psychological Review 2006;**113**(4):766-86 doi: 10.1037/0033-295X.113.4.766.

27. Buodo G, Sarlo M, Palomba D. Attentional resources measured by reaction times highlight differences within pleasant and unpleasant, high arousing stimuli. Motivation and Emotion 2002;**26**(2):123-38 doi: 10.1023/A:1019886501965.
