# Supplementary material for: Internet-delivered attentional bias modification training (iABMT) for the management of chronic musculoskeletal pain: a protocol for a randomised controlled trial
Source: BMJ Open. 2020 Feb 20;10(2):e030607. doi: 10.1136/bmjopen-2019-030607 (PMC7045192; doi:10.1136/bmjopen-2019-030607)
Supplement: Supplementary data [file bmjopen-2019-030607supp002.pdf]

ATTENTIONAL BIAS MODIFICATION PROTOCOL

Supplementary Material 2 – Outcome Measures

A detailed description of each outcome measure used in this study is provided here. While the questionnaires will be administered online as part of this study, the psychometric properties reported are for paper and pencil versions of the questionnaires.

The BPI-SF[1] is a commonly used measure of pain intensity and pain interference. Participants indicate their worst, least, average and current pain on an 11-point numerical scale ranging from *no pain at all* to *pain as bad as you can imagine*. Additional questions include how much relief pain medication has provided, along with pain interference on general activity, mood, walking ability, normal work, relations with others, sleep, and enjoyment of life. A body diagram is also provided for participants to indicate where they feel pain. The psychometric properties of the BPI are well supported in patients with cancer pain and other pain populations[1 2], including internal consistency of pain intensity and interference items as assessed in patients with chronic nonmalignant pain (Cronbach's alpha .85 and .88 respectively).[3]

The HADS[4] consists of 14 items measuring the severity of anxiety and depression symptoms (seven items each) over the past week. Each item is measured on a four point scale, with possible scores ranging from 0 – 21 for each subscale. Higher scores indicate higher levels of anxiety and depression. A review of studies using the HADS (the majority of which were conducted with patients with cancer or other physical health conditions) has supported the internal consistency of anxiety (.68 - .93, mean .82) and depression (.67 - .90, mean .82) subscales.[5]

The STAI[6] is a 40-item measure of state and trait anxiety (20 items each). Each item is measuerd on a four point scale , with possible scores for both state and trait levels ranging between 20 and 80. Higher scores represent more intense or more frequent feelings of anxiety. A review of 45 articles reporting psychometric properties from clinical and non-clinical samples of state and trait subscales found high levels of internal consistency (.91 and .89 respectively) and test-retest reliability (.70 and .88 respectively).[7]

Liossi C, *et al. BMJ Open* 2020; 10:e030607. doi: 10.1136/bmjopen-2019-030607

ATTENTIONAL BIAS MODIFICATION PROTOCOL

The FOP-III[8] is a 30 item measure of pain-related fear. On a 5-point scale, respondents are asked to rate how fearful they are of pain associated with specific situations and events (e.g., being in an automobile accident). Possible scores range between 30 and 150, with higher scores representing a more intense fear of pain. In addition to a total score, the measure also provides subscales of minor, severe, and medical pain-related fear. The FOP-III is commonly used in clinical and non-clinical populations, and possesses high internal consistency as indexed in a non-clinical sample (total score = .93; severe = .88; minor = .86, medical = .88).[9]

The MOS-SS[10] is a twelve-item questionnaire developed for patients with chronic illness, measuring six dimensions of sleep over the past four weeks; sleep initiation (time to fall asleep), quantity (hours per night sleeping), maintenance, respiratory problems, perceived adequacy, and somnolence. A sleep problems index which summarises information from nine items may also be scored. Responses are recoded on a 0 – 100 scale, with higher scores indicating more of the concept being measured (e.g., greater difficulties falling asleep). Research has supported the internal consistency of the MOS-SS scales to be acceptable (.70) or higher in samples of patients with neuropathic pain.[11 12]

The SOT[13] assesses participants' attitudes towards their online administered treatment, and will be used at the study endline. Participants state how strongly they agree/disagree with each of the following items: (a) the use of a computer to access treatment, (b) level of easiness of the online intervention, (c) duration of the potential effects of the treatment, and (d) potential recommendation of online interventions to others. Responses range from "*Agree very strongly*" to "*Disagree very strongly*". An additional question asks participants how helpful they have found the online intervention, with responses ranging from "*Very helpful*" to "*Not at all helpful*". Two open questions are also included that require participants to state the aspects of the intervention they most liked and disliked.

The EOT, developed by the researchers, will be presented at the end of every online session. The EOT includes three questions. The first asks participants to indicate where they

completed the online training session (e.g., home or office). The second is related to whether or not they have been distracted during the training and, if distracted, to indicate the source of distraction (e.g., telephone ringing). The third question asks participants to rate the level of their concentration during the online task on an 11-point Visual Analogue Scale (VAS) numbered from 0 (i.e., *no concentration at all*) to 10 (i.e. *very concentrated*).

ATTENTIONAL BIAS MODIFICATION PROTOCOL

References

1. Cleeland CS, Ryan KM. Pain assessment: global use of the Brief Pain Inventory. Ann Acad Med Singapore 1994;**23**(2):129-38.

2. Jensen MP. The validity and reliability of pain measures in adults with cancer. The Journal of Pain 2003;**4**(1):2-21 doi: 10.1054/jpai.2003.1.

3. Tan G, Jensen MP, Thornby JI, et al. Validation of the Brief Pain Inventory for chronic nonmalignant pain. The Journal of Pain 2004;**5**(2):133-37 doi: 10.1016/j.jpain.2003.12.005.

4. Zigmond AS, Snaith RP. The hospital anxiety and depression scale. Acta Psychiatr Scand 1983;**67**(6):361-70.

5. Bjelland I, Dahl AA, Tangen T, et al. The validity of the Hospital Anxiety and Depression Scale: An updated literature review. Journal of Psychosomatic Research 2002;**52**:69-77 doi: 10.1016/S0022-3999(01)00296-3.

6. Spielberger CD, Gorsuch RL, Lushene RE. *State Trait Anxiety Inventory*. Palo Alto, California: Consulting Psychologists Press, 1970.

7. Barnes LLB, Harp D, Jung WS. Reliability generalization of scores on the Spielberger State-Trait Anxiety Inventory. Educational and Psychological Measurement 2002;**62**(4):603 - 18 doi: 10.1177/0013164402062004005.

8. McNeil DW, Rainwater A. Development of the Fear of Pain Questionnaire-III. Journal of Behavioral Medicine 1998;**21**(4):389-410 doi: 10.1023/A:1018782831217.

9. Roelofs J, Peters ML, Deutz J, et al. The Fear of Pain Questionnaire (FPQ): further psychometric examination in a non-clinical sample. PAIN 2005;**116**(3):339-46 doi: 10.1016/j.pain.2005.05.003.

10. Hays RD, Stewart AL. Sleep Measures. Measuring functioning and well-being. In: Steweart AL, JEJ W, eds. Measuring function and well-being; the medical outcome study approach. Duke: Duke University Press, 1992:235-59.

ATTENTIONAL BIAS MODIFICATION PROTOCOL

11. Hays RD, Martin SA, Sesti AM, et al. Psychometric properties of the medical outcomes study sleep measure. Sleep Medicine 2005;**6**(1):41-44 doi: 10.1016/j.sleep.2004.07.006.

12. Viala-Danten M, Martin S, Guillemin I, et al. Evaluation of the reliability and validity of the Medical Outcomes Study sleep scale in patients with painful diabetic peripheral neuropathy during an international clinical trial. Health and Quality of Life Outcomes 2008;**6**(113) doi: 10.1186/1477-7525-6-113.

13. Richards D, Timulak L. Satisfaction with therapist-delivered vs. self-administered online cognitive behavioural treatments for depression symptoms in college students. British Journal of Guidance & Counselling 2013;**41**(2):193-207 doi: 10.1080/03069885.2012.726347.
