# Supplementary material for: Internet-delivered attentional bias modification training (iABMT) for the management of chronic musculoskeletal pain: a protocol for a randomised controlled trial
Source: BMJ Open. 2020 Feb 20;10(2):e030607. doi: 10.1136/bmjopen-2019-030607 (PMC7045192; doi:10.1136/bmjopen-2019-030607)
Supplement: Supplementary data [file bmjopen-2019-030607supp003.pdf]

Supplementary Material 3 – Think Aloud Study

A qualitative think aloud study[1 2] was conducted following the development of the initial version of the ABMT intervention to address the question: *What are the thoughts and first impressions of individuals with chronic musculoskeletal pain towards an online ABMT programme?* Ethical approval was obtained from the University of Southampton Research Ethics Committee (ERGO ID: 26486). Thirteen participants (mean age = 37.54, SD = 16.31, range 21 – 60 years) with chronic musculoskeletal pain were recruited from the South of England from a chronic pain support group.

Each participant met with the researcher (FH) on a single occasion to take part in a think aloud interview followed by a short semi-structured interview. Participants navigated their way through the iABMT Lifeguide website and completed the first assessment visual-probe task, with the researcher seated next to them. The researcher used an interview topic guide featuring standardized instructions that were read at the beginning of the study, as well as a list of pre-determined neutral prompts used when the participant stopped talking or showed a reaction to the material through their facial expressions. On occasions where the participant deviated from the topic, the researcher attempted to steer the participant's focus back to the task at hand. The prompts used were similar to those in former research[3], such as '*what are you thinking right now?*' and '*tell me why you think that'.*

Following the think-aloud interview, a short semi-structured interview took place for the researcher to ask questions about any specific aspects of the intervention that had not yet been discussed. Questions were also asked regarding thoughts about the intervention as a whole. This method of triangulation is commonly used to complement a think-aloud interview because it can be difficult to interpret spoken thoughts alone.[4 5] Following transcription of the interviews, thematic analysis using inductive encoding was used to identify themes that clearly represent the raw data, following guidelines set out by Braun and

Clarke.[6] Three themes and 13 subthemes were identified, which are shown in a thematic map in Figure S1 and defined in Table S1 below. While it is beyond the scope of this article to present the results of this thematic analysis in full, the main points resulting in amendments to the iABMT intervention are presented here.

Regarding the iABMT website, participants liked the continuity of design between webpages, although pointed out inconsistencies in the formatting of text, which have been rectified. On some pages the volume of text was considered to be too great, and therefore where possible text has been reduced, language simplified, better use of spacing made, and longer paragraphs divided into multiple shorter paragraphs. While some participants were positive about the inclusion of images, others felt additional visual stimuli could be added throughout. It was therefore decided to only include images where these would help emphasize the points being made. Regarding the visual-probe task sessions, one important practicality raised was ability of participants to stay seated for a sufficient amount of time in order to complete the task in a single session. An additional inclusion criteria has therefore been added to the study, being that participants must be able to sit at a personal computer for forty minutes. It was also mentioned that participants needed sufficient computer skills to take part in the study. The inclusion criteria were therefore amended to state that participants need both access to and familiarity using a Window-based computer.

ATTENTIONAL BIAS MODIFICATION PROTOCOL

References

1. Yardley L, Ainsworth B, Arden-Close E, et al. The person-based approach to enhancing the acceptability and feasibility of interventions. Pilot and Feasibility Studies 2015;**1**(1):37.

2. Van den Haak MJ, De Jong MD, Schellens PJ. Evaluation of an informational web site: three variants of the think-aloud method compared. Technical Communication 2007;**54**(1):58-71.

3. Morrison L, Moss-Morris R, Michie S, et al. Optimizing engagement with internet-based health behaviour change interventions: Comparison of self-assessment with and without tailored feedback using a mixed methods approach. British Journal of Health Psychology 2014;**19**(4):839-55 doi: 10.1111/bjhp.12083.

4. Charters E. The use of think-aloud methods in qualitative research an introduction to think-aloud methods. Brock Education Journal 2003;**12**(2).

5. Anthierens S, Tonkin-Crine S, Douglas E, et al. General practitioners' views on the acceptability and applicability of a web-based intervention to reduce antibiotic prescribing for acute cough in multiple European countries: a qualitative study prior to a randomised trial. BMC Family Practice 2012;**13**(101) doi: 10.1186/1471-2296-13-101.

6. Braun V, Clarke V. Using thematic analysis in psychology. Qualitative Research in Psychology 2006;**3**(2):77-101 doi: 10.1191/1478088706qp063oa.

ATTENTIONAL BIAS MODIFICATION PROTOCOL

Table S1. Definitions of themes and subthemes identified in the think aloud study

Theme/Sub-theme	Definition
A. AMBT website	Comments relating to the ABMT website including the post-intervention questionnaires
A1. Accessibility and usability	Comments relating to the ease with which participants were able to use the website and the extent to which it was deemed to be accessible
A2. Visual design	Comments relating to the visual design and appearance of the website, including colour, orientation of elements and text
A3. Questionnaires	Comments regarding any aspect of the post-intervention questionnaires, including their purpose and presentation
A4. Specific menu pages	Comments pertaining to the six main menu pages, which are titled (i) How does ABMT work, (ii) Will ABMT cure my pain, (iii) The team behind ABMT, (iv) Evidence for ABMT, (v) Why try ABMT, and (iv) What is it like to use ABMT?
B. Visual-probe task	Comments relating to any and all aspects of the visual-probe tasks
B1. Experiences of testing the intervention	Comments provided prior to and during testing of the visual-probe task
B2. Attitudes towards task	Positive and negative attitudes expressed by participants regarding the visual-probe task
B3. Practicalities	Comments pertaining to the feasibility and practicalities of completing the visual-probe task, including commitment needed
C. Influencing factors	All personal and situational factors that may affect the participants' experiences of testing the intervention
C1. Interviewing situation	Comments relating to the style of qualitative research adopted for the think aloud study
C2. Personal barriers	Comments relating to personal factors that hindered participants' completion of the visual-probe task
C3. Personal facilitators	Comments relating to personal factors that increased participants' ability to complete the visual-probe task and improved their attitude regarding it

Liossi C, *et al. BMJ Open* 2020; 10:e030607. doi: 10.1136/bmjopen-2019-030607
