# Supplementary material for: Internet-delivered attentional bias modification training (iABMT) for the management of chronic musculoskeletal pain: a protocol for a randomised controlled trial
Source: BMJ Open. 2020 Feb 20;10(2):e030607. doi: 10.1136/bmjopen-2019-030607 (PMC7045192; doi:10.1136/bmjopen-2019-030607)
Supplement: Author's manuscript [file bmjopen-2019-030607.draft_revisions.pdf]

BMJ Open is committed to open peer review. As part of this commitment we make the peer review history of every article we publish publicly available.

When an article is published we post the peer reviewers' comments and the authors' responses online. We also post the versions of the paper that were used during peer review. These are the versions that the peer review comments apply to.

The versions of the paper that follow are the versions that were submitted during the peer review process. They are not the versions of record or the final published versions. They should not be cited or distributed as the published version of this manuscript.

BMJ Open is an open access journal and the full, final, typeset and author-corrected version of record of the manuscript is available on our site with no access controls, subscription charges or pay-per-view fees (http://bmjopen.bmj.com).

If you have any questions on BMJ Open's open peer review process please email info.bmjopen@bmj.com

BMJ Open

Internet-delivered attentional bias modification training (iABMT) for the management of chronic musculoskeletal pain: a protocol for a randomised controlled trial

Journal:	*BMJ Open*
Manuscript ID	bmjopen-2019-030607
Article Type:	Protocol
Date Submitted by the Author:	22-Mar-2019

Complete List of Authors:	Liossi, Christina; University of Southampton Faculty of Engineering and the Environment, Psychology;<br>Georgallis, Tsampikos; University of Southampton Faculty of Engineering and the Environment, School of Psychology<br>Zhang, Jin; University of Southampton Faculty of Engineering and the Environment, School of Psychology<br>Hamilton, Fiona; University of Southampton Faculty of Engineering and the Environment, School of Psychology<br>White, Paul; University of the West of England, Engineering, Design and Mathematics<br>Schoth, Daniel; University of Southampton Faculty of Engineering and the Environment, School of Psychology

Keywords:	Chronic pain, Musculoskeletal pain, Visual-probe task, Attention bias modification, Internet-delivered therapy, Randomised-controlled trial

SCHOLARONE™
Manuscripts

I, the Submitting Author has the right to grant and does grant on behalf of all authors of the Work (as defined in the below author licence), an exclusive licence and/or a non-exclusive licence for contributions from authors who are: i) UK Crown employees; ii) where BMJ has agreed a CC-BY licence shall apply, and/or iii) in accordance with the terms applicable for US Federal Government officers or employees acting as part of their official duties; on a worldwide, perpetual, irrevocable, royalty-free basis to BMJ Publishing Group Ltd ("BMJ") its licensees and where the relevant Journal is co-owned by BMJ to the co-owners of the Journal, to publish the Work in this journal and any other BMJ products and to exploit all rights, as set out in our licence.

The Submitting Author accepts and understands that any supply made under these terms is made by BMJ to the Submitting Author unless you are acting as an employee on behalf of your employer or a postgraduate student of an affiliated institution which is paying any applicable article publishing charge ("APC") for Open Access articles. Where the Submitting Author wishes to make the Work available on an Open Access basis (and intends to pay the relevant APC), the terms of reuse of such Open Access shall be governed by a Creative Commons licence – details of these licences and which Creative Commons licence will apply to this Work are set out in our licence referred to above.

Other than as permitted in any relevant BMJ Author's Self Archiving Policies, I confirm this Work has not been accepted for publication elsewhere, is not being considered for publication elsewhere and does not duplicate material already published. I confirm all authors consent to publication of this Work and authorise the granting of this licence.

For peer review only

Internet-delivered attentional bias modification training (iABMT) for the management of chronic musculoskeletal pain: a protocol for a randomised controlled trial

Christina Liossi[1*], Tsampikos Georgallis[1], Jin Zhang[1], Fiona Hamilton[1], Paul White[2], and Daniel E. Schoth[1,]

[1]Pain Research Laboratory, School of Psychology, University of Southampton, Highfield, Southampton, UK; [2] Engineering, Design and Mathematics, University of the West of England, Bristol, UK

Protocol version 2.0; 22/03/19

Corresponding author: Prof. Christina Liossi, Pain Research Laboratory, Department of Psychology, University of Southampton, Highfield, Southampton, SO17 1BJ, UK. E-mail: C.Liossi@soton.ac.uk, Tel: 0044 (0)238059 4645.

Trial registration: This Study Protocol was registered in ClinicalTrials.gov (NCT02232100) on 1 September 2014

Word count: 3625

For peer review only

Abstract

Introduction: Chronic musculoskeletal pain is a complex medical condition that can significantly impact quality of life. Patients with chronic pain demonstrate attentional biases towards pain-related information, and the therapeutic benefits of modifying attentional biases by implicitly training attention away from pain-related information towards neutral information have been supported. Limited research however has explored the efficacy of modifying pain-related biases via the internet, which enables evaluation of training in real-world settings. This protocol describes a randomised, double-blind internet-delivered attentional bias modification intervention, aimed to evaluate the efficacy of the intervention on improving pain intensity and pain interference. Secondary outcomes are state and trait anxiety, depression, pain-related fear, and sleep impairment. This study will also explore the effects of training intensity on these outcomes, along with participants' perceptions about the online therapy. **Methods and analysis**: The study is a double-blind randomised control trial with four arms exploring the efficacy of online attentional bias modification training versus placebo attentional control training theorised to offer no specific therapeutic benefit. Participants with chronic musculoskeletal pain will be randomised to one of four groups: (i) 10-session attentional modification group; (ii) 10-session attentional control group; (iii) 18-session attentional modification group; or (iv) 18-session attentional control group. In the attentional modification groups, the visual-probe task will be used to implicitly train attention away from threatening information towards neutral information. Following the intervention, participants will complete a short interview exploring their perceptions about the online training. Data will be analysed and reported separately for participants aged 16 – 24 and 25 – 60. **Ethics and dissemination**: This study has been approved by the University of Southampton Research Ethics Committee. Results will be published in peer-reviewed journals, academic conferences, and in lay reports for pain charities and patient support groups. **Registration**: The study protocol has been registered with ClinicalTrial.gov (NCT02232100).

For peer review only

Strengths and limitations of this study

- This protocol describes the first internet-delivered intervention to explore the efficacy of attentional bias modification training in patients with chronic musculoskeletal pain.
- Recruitment of a diverse sample in terms of age, socioeconomic status, and geographical region.
- Results will provide important information regarding dose effects, age effects and participants' experiences and attitudes using the online intervention.
- Limited control over the conditions under which training is completed.
- Impossibility of independently verifying participant diagnosis.

Keywords: chronic pain; musculoskeletal pain; visual-probe task; attention bias modification; internet-delivered therapy; randomised-controlled trial.

For peer review only

Introduction

Chronic musculoskeletal pain (CMSK) is pain arising from bones, muscles, ligaments, tendons and/or joints lasting for more than three months.[1] Prevalence estimates of CMSK vary considerably, which for adults is estimated to affect between 11.4 and 24% of the population[2] and for children and adolescents ranges between 4 and 40% of the population.[3] Negative effects on quality of life are commonly reported in adult[4-7] and paediatric[8-11] CMSK populations, and theory driven research[12-14] has shown that patients with chronic pain exhibit attentional biases (i.e., a selective attention) for salient pain-related words (e.g.,[15-18]) and images (e.g.,[19 20]) relative to neutral information, particularly at longer stimuli presentation times (i.e., > 1000 ms) associated with maintained attention as indicated in meta-analyses.[21 22] Preliminary research has explored the effects of re-directing attention may have on pain and pain-related distress using attentional bias modification (ABM) techniques. ABM is a computer-based intervention which implicitly trains attention away from threat-related cues toward neutral information, and is frequently achieved through a modified version of the visual-probe task[23] (Figure 1). Two small randomised controlled trials (RCTs)[24 25] and one pilot study[26] have used the modified visual-probe task to assess ABM in adults with chronic pain, which overall revealed statistically significant improvements in pain, disability and pain-related distress. One study reported clinically significant improvements in pain intensity, anxiety, depression and pain interference[26], and another in and pain intensity.[25] One small RCT in adolescents with chronic pain reported no significant differences between ABM, placebo training and waiting-list groups on pain or pain-related outcomes.[27] Given the methodological shortcomings and small sample sizes of previous studies, further research conducted in real-world settings is needed to determine the therapeutic benefits of ABM for patients with chronic pain, along with the optimal form ABM should take and the impact of age on outcomes and intervention engagement.

This article presents a study protocol for a randomised, double-blind internet-delivered ABM intervention. The results of a pilot think aloud study informing the design of the

For peer review only - http://bmjopen.bmj.com/site/about/guidelines.xhtml

intervention are also presented. The aim of this RCT is to assess the efficacy of internet-delivered ABM in improving pain and pain-related distress in people aged 16 –60 (considered separately in two age brackets [i.e., 16-24 and 25-60]) with CMSK. The primary outcome measures are pain intensity and pain interference. Secondary outcome measures are state and trait anxiety, depression, pain-related fear and sleep impairment, and engagement to and satisfaction with the online visual-probe training task. Dose effects will be explored via the inclusion of 10 versus 18 session conditions. It is hypothesised that participants in the intervention group, relative to those in the placebo group, will report significant reductions in pain intensity, pain interference, anxiety, depression, sleep impairment and pain-related fear at study endline. It is also hypothesised that dose will significantly moderate these findings, with greater effects in 18 compared to 10 sessions.

Methods and Analysis

Participants

Participants will be recruited via press announcements, social media, and patient organizations throughout the UK. Inclusion criteria will be: (a) aged between 16 to 60 years old, (b) experiencing any type of CMSK (i.e., any condition that involves pain lasting for more than three months and arises from bones, muscles and/or joints), (c) able to sit at a personal computer for forty minutes, (d) normal or corrected to normal vision, (e) access to the internet at least twice a week, (f) access to, and familiarity using, a Windows-based computer, (g) successful completion of primary school, and (h) living in the United Kingdom. Exclusion criteria will be (1) experiencing malignant CMSK (i.e., pain caused due to a tumour), (2) a diagnosis of any psychiatric disorder, either currently or within the last 5 years, and (3) currently under psychiatric therapy. There are no restrictions placed on concomitant care and participants are not required to make any changes to current treatments they may be receiving.

Patient and Public Involvement

For peer review only

Participant burden of the intervention and research measures were assessed using individual interviews and informal feedback from patients participating in two pilot studies.[26 28] Patients will not be involved in recruitment of participants or conduct of the study. Results of this study will be disseminated to participants through presentation at consumer and community forums.

Study Design and Setting

The study is a double-blind randomised control trial (RCT) with four arms exploring the efficacy of online ABM training versus a placebo attentional control training theorised to offer no specific therapeutic benefits. The choice of comparator was selected as attentional control training closely mimics attentional bias modification training, and is frequently used as a comparator in ABM studies (e.g.,[24 25 29 30]). The study will take place in a location of the participants choice (which we anticipate will typically be their home), which is accessed via personal computer connected to the internet.

The study design has a 2 x 2 x 3 experimental structure, with multiple non-commensurate dependent variables measuring pain intensity and interference, emotional functioning and pain-related disability. ABM intervention is a two-level factor (10AMG and 18AMG), placebo control is a two-level factor (10ACG and 18ACG) with measures recorded at three points in time (baseline, endline, and 6-month follow-up). The study is powered primarily to establish treatment efficacy for the primary outcome variables of pain intensity and pain interference by considering baseline and endline measures between 10AMG and 10ACG. Randomisation should ensure baseline comparability on measures between these two groups, and a difference in beneficial outcomes between these groups will be modelled as a two-way interaction in the corresponding analysis of variance. For n = 20 per group the analysis of the interaction effect for this two group comparison will proceed on 1 and 38 degrees of freedom. With an assumed medium sized effect (e.g. f-squared = 0.0625, and a 0.5 conservative correlation between the individual levels of the repeated measures factor) the test of significance for the interaction effect

For peer review only - http://bmjopen.bmj.com/site/about/guidelines.xhtml

will have in excess of 80% power (alpha = 0.05, beta = 0.13, non-centrality parameter lambda = 10). Data will be analysed and reported separately for participants aged 16 – 24 and 25 – 60.

Randomisation and Sample Size

Block randomisation of size four, performed via a randomisation command in the Lifeguide platform[31], will be used to sequentially randomise consecutive consenting participants to one of four groups: (i) 10-session attentional modification group (10AMG); (ii) 10-session attentional control group (10ACG); (iii) 18-session attentional modification group (18AMG); or (iv) 18-session attentional control group (18ACG). An allocation concealment mechanism will be used to ensure neither participants nor researchers know the study group to which the next participant will be assigned. Participants randomised to either 10-session condition will receive ten 35-minute online sessions across a four-week period, while those randomised to either 18-session condition will receive eighteen 35-minute online sessions across an eight-week period. All online sessions will be administered twice a week on a separate set of days (Monday-Thursday, Tuesday-Friday). Participants randomised to either AMG condition will receive attentional training via the modified training version of the visual probe-task, while those randomised to either ACG condition will receive placebo training via the standard version of the visual-probe task. To maintain the overall quality and legitimacy of the intervention, code breaks (i.e., unbinding) will only occur in exceptional circumstances. Adherence will be monitored electronically via Lifeguide. We anticipate that a sample of 80 participants per age bracket i.e., 16-24 and 25-60 will afford the opportunity to robustly test the hypothesis, which is also considered feasible to recruit over a 2-year period.

Procedure

Interested individuals will be assessed against the inclusion and exclusion criteria via telephone interview. If eligible, they will receive a copy of the participant information sheet (PIS) detailing the requirements and procedure of the study, along with a link to the study website which provides further details on ABM and the research team. If the individual consents to take

For peer review only

part they will be required to create an online ABM account. During registration, participants will select their preferred days for training, which will be either (i) Monday/Thursday or (ii) Tuesday/Friday.

Session 1 is the baseline session, and includes the first assessment visual-probe task assessing attentional biases prior to beginning the intervention, along with the questionnaire battery assessing the primary and secondary outcome measures. Randomisation will take place following session 1, where participants will be informed of the length of their training (i.e., four or eight weeks). Participants randomised to either of the ten session conditions will complete four pictorial and four linguistic training sessions, while those randomised to either of the 18 session conditions will complete eight pictorial and eight linguistic training sessions (baseline and endline assessment sessions comprise the remainder of the 10/18 sessions). Participants' attentional biases will be re-assessed at endline (i.e., during the final session 10/18) using the same assessment version of the visual probe task used at baseline and the same questionnaire battery. Participants' satisfaction with the online training will also be assessed. Six months after the last session, participants will be invited to again complete the online assessment visual-probe task and questionnaire battery. Within two weeks of the completion of the six-month follow-up session, participants will be invited to take part in a brief telephone semi-structured interview including 15 questions spread across five thematic sections: (a) motivation and expectations about the study, (b) experience of the online study – person, environmental and lifestyle parameters, (c) experience of the visual-probe task, (d) understanding of the process of change, and (e) the visual-probe task as a potential therapeutic tool in pain management. The study flow is presented in Table 1.

To retain as many participants as possible, reminder emails will be sent to participants 24 hours before each scheduled session becomes available, and also at 13:00 on the scheduled session date should the participant not have already logged into their iABMT account. An additional reminder email will be sent one week before their 6-month follow-up session is ready.

For peer review only

Participants will also be sent encouragement emails informing them when they have reach specific milestones (i.e., successfully completing 5 sessions for those randomised to complete 10 sessions; successfully completing 6 and 12 sessions for those randomised to 18 sessions). Participants will also have the option of receiving an SMS message on their mobile phone at 09:00 on the morning of each scheduled session.

The Intervention

The intervention will be hosted using LifeGuide and iSurvey platforms, both developed by the University of Southampton. LifeGuide is an open-source software which allows researchers to create online interventions.[31] iSurvey is a survey generation tool which allows researchers to create and disseminate questionnaires online. Participants will register for the intervention using LifeGuide, which will also host the majority of the questionnaires. iSurvey will be used to host the visual-probe tasks, along with the Engagement with Online Training questionnaire and the Brief Pain Inventory[32] that appear at the end of every visual-probe task session. The flow of sessions is provided in Figure 2, and examples of webpages are shown in Figure 3. On loading the intervention homepage, the individual is provided with a brief welcome to the website and rationale for the intervention. Individuals are able to either create an iABMT account or log into their existing account. Before any session participants are asked to indicate whether they have enough time and are ready to begin the session. Participants are only permitted to complete one session per day. Should a participant complete their missed session on a subsequently scheduled date (e.g., completing their scheduled session from Monday on Thursday instead), all remaining sessions are shifted back accordingly. This process retains the structure of the intervention.

Guidance has been informed by research showing attention to be a limited capacity resource[33 34], sleepiness to negatively influence concentration and performance[35], alcohol to impair speed of information processing and cognition[36], energy drinks to be associated with restlessness and nervousness[37], caffeine to improves alertness[38], large meals to be associated with

For peer review only - http://bmjopen.bmj.com/site/about/guidelines.xhtml

impairments in cognitive functioning[39], and hunger to influence patterns of attention.[40 41] Participants are provided with guidance to ensure concentration and comfort is maintained throughout each session: (1) allowing enough time for the session with no anticipated distractions, (2) adjusting screen brightness to match the brightness of the environment, (3) closing distracting applications such as Twitter and Facebook, (4) avoiding completing the session when tired, (5) not consuming alcohol at least 3 hours prior to the session, (6) caution in the use of energy drinks which may cause restlessness, (7) if the participant is a coffee or tea drinker, suggesting a cup 30 minutes prior to the session, and (8) avoiding completing the session when hungry or directly after a meal.

Visual-Probe Tasks. The flow of visual-probe sessions is illustrated in Figure 2. In total there will be eight linguistic and eight pictorial visual-probe training tasks used in the study, each with different stimuli. A complete description of the stimuli including ratings of valence, arousal, pain intensity, written frequency, and low-level features, is provided in Supplementary Material 1. A separate linguistic visual-probe task will be used in the three assessment sessions, featuring different stimuli to the linguistic training visual-probe tasks. The use of separate assessment and training stimuli is necessary to establish whether training effects generalise to novel stimuli not used in the actual training.

The ABM training version of the visual-probe task to be completed by AMG groups includes three main stages per trial (Figure 1). Following a 500 ms initial fixation point, a stimulus pair are presented in distinct locations (e.g., above and below the initial fixation point) for either 500 or 1250 ms. The stimulus pair may be either words or images, although critically includes one threat-related and one neutral stimulus. 500 and 1250 ms presentation times are used to explore biases in initial orienting of attention and maintained attention respectively.[21] Immediately following their presentation, the stimuli disappear and a probe appears (i.e., either *'p'* or *'q'*) in the location of the neutral stimulus. Participants indicate the location of the probe as quickly and as accurately as possible via corresponding keys on their keyboard. In the standard

For peer review only - http://bmjopen.bmj.com/site/about/guidelines.xhtml

version of this paradigm completed by ACG groups and used in the assessment sessions, the probe replaces the neutral and threat-related stimulus an equal number of times.

Outcome Measures

The primary outcome measures of pain intensity and pain interference will be assessed by the Brief Pain Inventory – Short Form (BPI-SF[32]). The secondary outcome measures of state and trait anxiety will be assessed by the State Trait Anxiety Inventory (STAI [42]), depression by the Hospital Anxiety and Depression Scale (HADS[43]), pain-related fear by the Fear of Pain Questionnaire III (FOP-III[44]), sleeping impairment by the Medical Outcome Study – Sleep Scale (MOS-SS[45]), and engagement to and satisfaction with the online visual-probe training task by the Satisfaction with the Online Training questionnaire (SOT[46]) and the Engagement with the Online Training questionnaire (EOT). A demographic questionnaire developed by the researchers will be used at baseline to collect information on participants' age, gender, ethnicity, socio-economic status, place of residence, and pain characteristics. Aside from the SOT and EOT, questionnaires will be administered to participants at baseline, endline and at six-month follow-up. Full details on each outcome measure are provided in Supplementary Material 2.

Think Aloud Study

A qualitative think aloud study[28] including a short semi-structured interview was conducted following the development of the initial version with the aim to understand participants' first impressions and attitudes towards the intervention, and to collate feedback that could be used to make improvements. Details and outcomes of this study are provided in Supplementary Material 3.

Analyses of the RCT Data

Quantitative Analyses

Statistical analyses will be performed in IBM SPSS Statistics for Windows 24. For visual-probe data, and as per former research[19 26], practice and incorrect trials will be removed prior to analyses. Box and whisker plots for overall data will be used to reveal overall outliers,

For peer review only - http://bmjopen.bmj.com/site/about/guidelines.xhtml

which will be removed. Following this, mean response times will be computed for each participant, with any response >3SD from their individual mean also removed as outliers. This process ensures extremely quick or slow responses do not unduly bias the results, which are typically removed when cleaning and screening visual-probe data in pain-related research (e.g., [18 47 48 49 50]). An attentional bias index will then be computed for each stimulus and presentation time condition using the following equation: (TuPl − TlPl)+(TlPu −TuPu))/2. Here T is the threatening stimulus, P is the probe, u is the upper position, and l is the lower position. The attention capturing quality of threatening stimuli is measured by subtracting the mean probe detection time for congruent trials from the mean probe detection time of incongruent trials.[21]

Split-half reliability will be computed via a bootstrap procedure, by randomly splitting the total number of trials in two halves, such that each half has the same number of congruent and incongruent trials. Attentional bias scores will be computed for each half and the correlation between them calculated across participants. This procedure will be repeated 100 times, and an average split-half correlation computed. In order to determine any differences between participants who complete the study and those who do not, a series of independent sample *t*-tests will be conducted on questionnaire data collected at baseline (i.e., pain intensity, pain interference, anxiety, depression, pain-related fear, and sleeping impairment) and baseline attentional bias scores.

Considering completers only, a 2 (Time; baseline, endline) x 2 (Group; AMG, ACG) x 2 (Sessions; 10, 18) ANOVA will be conducted on attentional bias scores as a means to determine any training effect on such biases. The analyses of the primary (i.e., pain intensity and pain interference) and secondary outcome measures (i.e., state and trait anxiety, depression, pain-related fear, and sleep impairment) will also be conducted via a series of 2 (Time; baseline, endline) x 2 (Group; AMG, ACG) x 2 (Sessions; 10, 18) ANOVAs. Of central interest to the study, these analyses will determine whether there are any differences between AMG and ACG groups at endline on patterns of attentional bias and outcome measures, and if so whether these

For peer review only - http://bmjopen.bmj.com/site/about/guidelines.xhtml

depends on number of sessions (due to randomisation significant differences are not anticipated between groups at baseline). Further to these analyses, a series of 2 (Group; AMG, ACG) x 2 (Sessions; 10, 18) ANOVAs will be conducted on questions from the Satisfaction with the Online Training questionnaire, which will determine whether there are any differences between groups in their attitudes towards their online administered treatment. Finally, data from the Engagement with the Online Training questionnaire will be used to compute mean level of concentration per participant throughout the intervention, with the AMG and ACG groups compared via independent *t*-test.

ANOVAs and *t*-tests will be used where appropriate in post-hoc analyses to clarify significant main effects and interactions. For all analyses, the alpha level will be set at 0.05, 2-tailed. For pairwise comparison analyses, Bonferonni correction will be applied to account for multiple comparisons. Correlations will be conducted in an exploratory manner between measures of attentional bias and the primary and secondary outcome measures, also with a Bonferonni correction applied due to the number of correlations made. In order to provide an unbiased estimate of the treatment effect[51], the intention-to-treat principle will applied in these analyses; all randomised participants will be included in the analyses in the groups they were randomised to. Multiple imputation will be used in instances of missing data[52 53]. Similar to former studies[25 26] the Reliable Change Index (RCI)[54] will be used to assess primary and secondary outcome measures for clinically significant changes. Here, a clinically significant result is a post-treatment score falling outside two standard deviations of the mean population of interest (in this study, two standard deviations outside pre-treatment means). In contrast to tests of statistical significance comparing group means, tests of clinical significance explore effects of treatment on the individual.[55]

Qualitative Analyses

Qualitative analysis will be used to detail participants' experiences completing the iABMT intervention. Specifically, thematic analysis using inductive encoding will be used to

For peer review only

identify themes present in the data obtained from the semi-structured interviews and the two open-ended questions included in the Satisfaction with the Online Training questionnaire. Guidelines provided by Braun and Clarke[56] will be followed. Following transcription of all interviews verbatim, initial codes will be generated. Potential themes and sub-themes will then be identified from the initial coding, which subsequently will be reviewed and refined. The validity of the themes in relation to the data set will also be considered. Each theme and sub-theme will then be named and defined, and a thematic map of the data finalized. Examples from the data will be illustrated for each theme and sub-theme in the final report.

Ethics and Dissemination

This study has been approved by the University of Southampton Research Ethics Committee (ERGO ID: 26486), and the study protocol registered with ClinicalTrial.gov (NCT02232100). Ethical approval will be sought before any protocol modifications are made and the study protocol will be updated at ClinicalTrial.gov. Participants will provide informed consent prior to taking part in the study. All data will remain confidential and stored on password-protected systems and databases. Lifeguide and iSurvey servers both use HTTPS connections for security purposes. All data will be anonymised prior to dissemination, and personally identifiable information not known to anyone other than the researchers. Participants may withdraw from the intervention at any point by contacting the researchers via email or telephone, or by simply opting not to log into their iABMT account for their scheduled sessions. Participants are also encouraged to contact the researchers should they experience any distress or discomfort completing the intervention. As per University policy, any harms or complaints arising from participation in the intervention will be reported to the University of Southampton Research Governance Office. Findings will be published in the most relevant, high-impact peer-reviewed journals and presented at relevant conferences. Lay reports will be written for interested pain charities and patient support groups.

For peer review only

For peer review only - http://bmjopen.bmj.com/site/about/guidelines.xhtml

List of figures and supplementary files

Table 1: Schedule of enrolment, intervention, and assessments.

Figure 1. A graphical representation of a typical visual-probe task training trial with linguistic stimuli.

Figure 2. Flow of the visual-probe task sessions for the 10-week and 18-week groups in the study

Figure 3. Examples of Lifeguide webpages included in the ABMT intervention.

Supplementary Material 1 – Stimuli development and properties

Supplementary Material 2 – Outcome Measures

Supplementary Material 3 – Think Aloud Study

For peer review only

References

1. Sambrook P, Taylor T, Schrieber L, et al. *The Musculoskeletal System: Basic Science and Clinical Conditions*. Second ed: Churchill Livingstone, Elsevier, 2010.

2. Cimmino MA, Ferrone C, Cutolo M. Epidemiology of chronic musculoskeletal pain. Best Practice & Research Clinical Rheumatology 2011;**25**(2):173-83 doi: 10.1016/j.berh.2010.01.012.

3. King S, Chambers CT, Huguet A, et al. The epidemiology of chronic pain in children and adolescents revisited: a systematic review. Pain 2011;**152**(12):2729-38 doi: 10.1016/j.pain.2011.07.016.

4. Tüzün EH. Quality of life in chronic musculoskeletal pain. Best Practice & Research Clinical Rheumatology 2007;**21**(3):567-79 doi: http://dx.doi.org/10.1016/j.berh.2007.03.001.

5. Breivik H, Collett B, Ventafridda V, et al. Survey of chronic pain in Europe: prevalence, impact on daily life, and treatment. Eur J Pain 2006;**10**(4):287-333 doi: 10.1016/j.ejpain.2005.06.009.

6. Bair MJ, Wu J, Damush TM, et al. Association of depression and anxiety alone and in combination with chronic musculoskeletal pain in primary care patients. Psychosom Med 2008;**70**(8):890-7 doi: 10.1097/PSY.0b013e318185c510.

7. Blyth FM, Noguchi N. Chronic musculoskeletal pain and its impact on older people. Best Practice & Research Clinical Rheumatology 2017;**31**(2):160-68 doi: 10.1016/j.berh.2017.10.004.

8. Harrison L, Wilson S, Munafò MR. Exploring the associations between sleep problems and chronic musculoskeletal pain in adolescents: a prospective cohort study. Pain research and management 2014;**19**(5):e139-e45 doi: 10.1155/2014/615203.

For peer review only

9. Flatø B, Aasland A, Vandvik I, et al. Outcome and predictive factors in children with chronic idiopathic musculoskeletal pain. Clinical and Experimental Rheumatology 1997;**15**(5):569-77.

10. Gold JI, Mahrer NE, Yee J, et al. Pain, fatigue and health-related quality of life in children and adolescents with chronic pain. Clinical Journal of Pain 2009;**25**(5):407 doi: 10.1097/AJP.0b013e318192bfb1.

11. Vinall J, Pavlova M, Asmundson GJ, et al. Mental health comorbidities in pediatric chronic pain: A narrative review of epidemiology, models, neurobiological mechanisms and treatment. Children 2016;**3**(4):40 doi: 10.3390/children3040040.

12. Pincus T, Morley S. Cognitive-processing bias in chronic pain: a review and integration. Psychological Bulletin 2001;**127**(5):599 - 617 doi: 10.1037/0033-2909.127.5.599.

13. Todd J, Sharpe L, Johnson A, et al. Towards a new model of attentional biases in the development, maintenance, and management of pain. PAIN 2015;**156**(9):5189-1600 doi: 10.1097/j.pain.0000000000000214.

14. Vlaeyen JWS, & Linton, S. J. Fear-avoidance and its consequences in chronic musculoskeletal pain: a state of the art. Pain 2000;**85**(3):317 - 32.

15. Liossi C, Schoth DE, Bradley BP, et al. Time-course of attentional bias for pain-related cues in chronic daily headache sufferers. European Journal of Pain 2009;**13**(9):963-69 doi: http://dx.doi.org/10.1016/j.ejpain.2008.11.007.

16. Haggman SP, Sharpe, L. A., Nicholas, M. K., & Refshauge, K. M. Attentional biases toward sensory pain words in acute and chronic pain patients. The Journal of Pain 2010;**11**(11):1136 - 45.

17. Sharpe L, Dear BF, Schrieber L. Attentional biases in chronic pain associated with rhumatoid arthritis: Hypervigilance or difficulties disengaging? The Journal of Pain 2009;**10**(3):329 - 35 doi: 10.1016/j.jpain.2008.10.005.

For peer review only - http://bmjopen.bmj.com/site/about/guidelines.xhtml

18. Schoth DE, Beaney R, Broadbent P, et al. Attentional, interpretation and memory biases for sensory-pain words in individuals with chronic headache. British Journal of Pain in press doi: 10.1177/2049463718789445.

19. Schoth D, Liossi C. Specificity and time-course of attentional bias in chronic headache: a visual-probe investigation. Clinical Journal of Pain 2013;**29**(7):583-90 doi: 10.1097/AJP.0b013e31826b4849.

20. Schoth DE, Liossi C. Attentional bias toward pictorial representations of pain in individuals with chronic headache. Clin J Pain 2010;**26**(3):244-50 doi: 10.1097/AJP.0b013e3181bed0f9.

21. Schoth D, Delgado Nunes V, Liossi C. Attentional bias towards pain-related information in chronic pain; a meta-analysis of visual-probe investigations. Clinical Psychology Review 2012;**32**(1):13 - 25 doi: 10.1016/j.cpr.2011.09.004.

22. Crombez G, Van Ryckeghem D, Eccleston C, et al. Attentional bias to pain-related information: a meta-analysis. Pain 2013;**154**(4):497-510.

23. MacLeod C, Mathews, A., & Tata, P. Attentional Bias in Emotional Disorders. Journal of Abnormal Psychology 1986;**95**(1):15 - 20.

24. Sharpe L, Ianiello, M., Dear, B. F, Nicholson Perry, K., Refshauge, K., Nicholas, M. K. Is there a potential role for attention bias modification in pain patients? Results of 2 randomised, controlled trials. Pain 2012;**153**(3):722-31.

25. Carleton R, Richter A, Asmundson G. Attention Modification in Persons with Fibromyalgia: A Double Blind, Randomized Clinical Trial. Cognitive Behaviour Therapy 2011;**40**(4):279-90.

26. Schoth DE, Georgallis T, Liossi C. Attentional bias modification in people with chronic pain: a proof of concept study. Cogn Behav Ther 2013;**42**(3):233-43 doi: 10.1080/16506073.2013.777105.

For peer review only - http://bmjopen.bmj.com/site/about/guidelines.xhtml

27. Heathcote LC, Jacobs K, Van Ryckeghem DML, et al. Attention bias modification training for adolescents with chronic pain: a randomized placebo-controlled trial. Pain 2018;**159**(2):239 - 51 doi: 10.1097/j.pain.0000000000001084.

28. Hamilton F. Perspectives of individuals with chronic musculoskeletal pain towards an online attentional bias modification training programme. University of Southampton, 2015.

29. Hakamata Y, Lissek, S., Bar-Haim, Y., Britton, J.C., Fox, N.A., Leibenluft, E., Ernst, M., & Pine, D. S. Attention bias modification treatment: A meta-analysis towards the establishment of novel treatment for anxiety. Biological Psychiatry 2010;**68**:982-90 doi: 10.1016/j.biopsych.2010.07.021.

30. Mogoaşe C, David D, Koster EH. Clinical efficacy of attentional bias modification procedures: An updated meta-analysis. Journal of Clinical Psychology 2014;**70**(12):1133-57 doi: 10.1002/jclp.22081.

31. Williams S, Yardley L, Wills GB. A qualitative case study of LifeGuide: users' experiences of software for developing Internet-based behaviour change interventions. Health Informatics J 2013;**19**(1):61-75 doi: 10.1177/1460458212458915.

32. Cleeland CS, Ryan KM. Pain assessment: global use of the Brief Pain Inventory. Ann Acad Med Singapore 1994;**23**(2):129-38.

33. Kahneman D. *Attention and effort*. New Jersey: Prentice Hall, 1973.

34. Broadbent D. *Perception and Communication*. Oxford: Pergamon, 1958.

35. Thomas M, Sing H, Belenky G, et al. Neural basis of alertness and cognitive performance impairments during sleepiness. I. Effects of 24 h of sleep deprivation on waking human regional brain activity. Journal of Sleep Research 2000;**9**(4):335-52 doi: 10.1046/j.1365-2869.2000.00225.x.

36. Tzambazis K, Stough C. Alcohol impairs speed of information processing and simple and choice reaction time and differentially impairs higher-order cognitive abilities. Alcohol and Alcoholism 2000;**35**(2):197-201 doi: 10.1093/alcalc/35.2.197.

For peer review only

37. Reissig CJ, Strain EC, Griffiths RR. Caffeinated energy drinks—a growing problem. Drug and Alcohol Dependence 2009;**99**(1):1-10 doi: 10.1093/alcalc/35.2.197.

38. Zwyghuizen-Doorenbos A, Roehrs TA, Lipschutz L, et al. Effects of caffeine on alertness. Psychopharmacology 1990;**100**(1):36-39 doi: 10.1007/BF02245786.

39. Müller K, Libuda L, Terschlüsen AM, et al. A review of the effects of lunch: On adults' short-term cognitive functioning. Canadian Journal of Dietetic Practice and Research 2013;**74**(4):181-88 doi: 10.3148/74.4.2013.181.

40. Piech RM, Pastorino MT, Zald DH. All I saw was the cake. Hunger effects on attentional capture by visual food cues. Appetite 2010;**54**(3):579-82 doi: 10.1016/j.appet.2009.11.003.

41. Al-Shawaf L. The evolutionary psychology of hunger. Appetite 2016;**105**:591-95 doi: 10.1016/j.appet.2016.06.021.

42. Spielberger CD, Gorsuch RL, Lushene RE. *State Trait Anxiety Inventory*. Palo Alto, California: Consulting Psychologists Press, 1970.

43. Zigmond AS, Snaith RP. The hospital anxiety and depression scale. Acta Psychiatr Scand 1983;**67**(6):361-70.

44. McNeil DW, Rainwater A. Development of the Fear of Pain Questionnaire-III. Journal of Behavioral Medicine 1998;**21**(4):389-410 doi: 10.1023/A:1018782831217.

45. Hays RD, Stewart AL. Sleep Measures. Measuring functioning and well-being. In: Steweart AL, JEJ W, eds. Measuring function and well-being; the medical outcome study approach. Duke: Duke University Press, 1992:235-59.

46. Richards D, Timulak L. Satisfaction with therapist-delivered vs. self-administered online cognitive behavioural treatments for depression symptoms in college students. British Journal of Guidance & Counselling 2013;**41**(2):193-207 doi: 10.1080/03069885.2012.726347.

For peer review only

For peer review only - http://bmjopen.bmj.com/site/about/guidelines.xhtml

47. Schoth DE, Parry L, Liossi C. Combined cognitive biases for sensory-pain and disability information in individuals with chronic headache. Journal of Health Psychology in press doi: 10.1177/1359105316664136.

48. Dear BF, Sharpe L, Nicholas M, et al. Pain-related attentional biases: The importance of the personal relevance and ecological validity of stimuli. The Journal of Pain 2011a;**12**(6):625-32.

49. Schoth DE, Williams S, Liossi C. Attentional bias for pain- and period-related symptom words in healthy women who experienced a recent painful period. European Journal of Pain 2015;**19**(6):745-51 doi: 10.1002/ejp.597.

50. McDermott MJ, Peck KR, Walters AB, et al. Do episodic migraineurs selectively attend to headache-related visual stimuli? Headache: The Journal of Head and Face Pain 2013;**53**(2):356-64 doi: 10.1111/head.12011.

51. Gupta SK. Intention-to-treat concept: a review. Perspectives in Clinical Research 2011;**2**(3):109 doi: 10.4103/2229-3485.83221.

52. Chan A-W, Tetzlaff JM, Gøtzsche PC, et al. SPIRIT 2013 explanation and elaboration: guidance for protocols of clinical trials. BMJ 2013;**346**:e7586 doi: 10.1136/bmj.e7586.

53. Groenwold RH, Donders ART, Roes KC, et al. Dealing with missing outcome data in randomized trials and observational studies. American Journal of Epidemiology 2012;**175**(3):210-17 doi: 10.1093/aje/kwr302.

54. Jacobson NS, Truax P. Clinical significance: A statistical approach to defining meaningful change. Psychotherapy Research 1991;**59**:12-19 doi: 10.1037/0022-006X.59.1.12.

55. Zahra D, Hedge C. The reliable change index: Why isn't it more popular in academic psychology? Psychology Postgraduate Affairs Group Quarterly 2010;**76**(76):14-19.

56. Braun V, Clarke V. Using thematic analysis in psychology. Qualitative Research in Psychology 2006;**3**(2):77-101 doi: 10.1191/1478088706qp063oa.

For peer review only

Authors' contributions: All authors have contributed to the development of the study protocol. CL, TG and DES conceived the project. Cl and DES registered the trial and are responsible for recruitment and implementation of the protocol. TG was primary responsible for the development of the online materials, including development and validation of linguistic and pictorial stimuli used in the visual-probe tasks. TG also assisted in programming the Lifeguide components. JZ was responsible for programming the visual-probe tasks to be used in the intervention, and assisted in programming the Lifeguide components. FH conducted the think aloud study. All authors contributed to the writing of this manuscript, and approve its final form. These same authors will be included on future publications presenting intervention results.

Funding: This work was supported by the Economic and Social Research Council (grant number: RES-000-22-4128). This funding source had no role in the design of this study and will not have any role during its execution, analyses, interpretation of the data, or decision to submit results. **Competing interests**: The authors declare that they have no competing interest.

For peer review only

ATTENTIONAL BIAS MODIFICATION PROTOCOL

Table 1. Schedule of enrolment, intervention, and assessments.

	STUDY PERIOD					
	Enrolment	Post-allocation				
TIMEPOINT**	$-t_1$	t_1 Baseline	t_2 Training	t_3 Evaluation	t_4 Follow-up	t_5 Study exit
**ENROLMENT:**						
**Eligibility screen**	X					
**Informed consent**	X					
**Allocation**		X				
**INTERVENTIONS:**						
Attentional bias modification training			X			
**ASSESSMENTS:**						
Demographic questionnaire		X				
EOT		X	X	X	X	
BPI-SF		X		X	X	
HADS		X		X	X	
STAI		X		X	X	
MOS-SS		X		X	X	
FOP-III		X		X	X	
SOT				X		
Interview						X

T1 = Baseline phase including standard assessment visual-probe task and questionnaires. Also includes randomization to groups.
T2 = Training phase including training visual-probe tasks.
T3 = Evaluation phase including standard assessment visual-probe task and questionnaires.
T4 = Six-month follow-up including standard assessment visual-probe task and questionnaires.
T5 = Telephone interview within two weeks of T4.

Note. EOT, Engagement with the Online Training questionnaire; BPI-SF, Brief Pain Inventory-Short Form; HADS, Hospital Anxiety and Depression Scale; STAI, State-Trait Anxiety Inventory; MOS-SS, Medical Outcome Study –Sleep Scale, FOP-III, Fear of pain questionnaire III; SOT, Satisfaction with the online treatment questionnaire

For peer review only

ATTENTIONAL BIAS MODIFICATION PROTOCOL

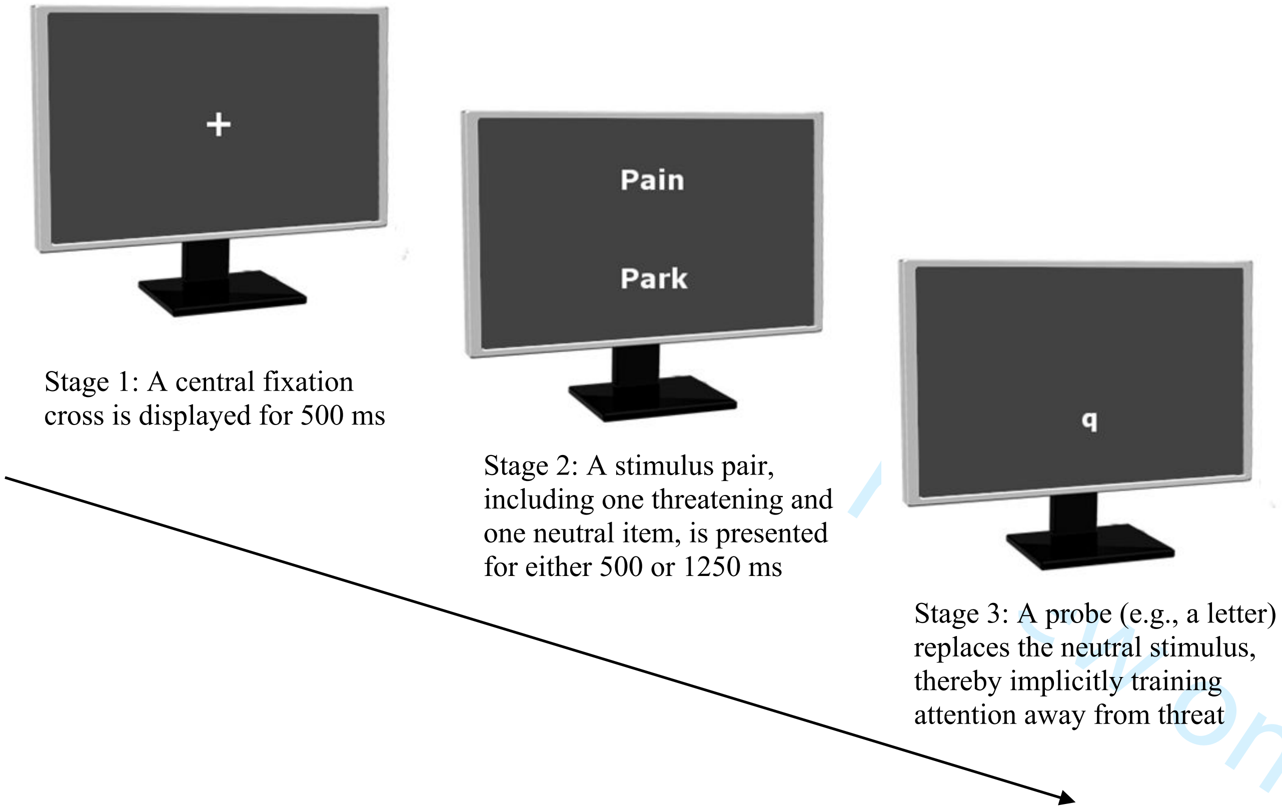

Figure 1. A graphical representation of a typical visual-probe task training trial with linguistic stimuli.

For peer review only - http://bmjopen.bmj.com/site/about/guidelines.xhtml

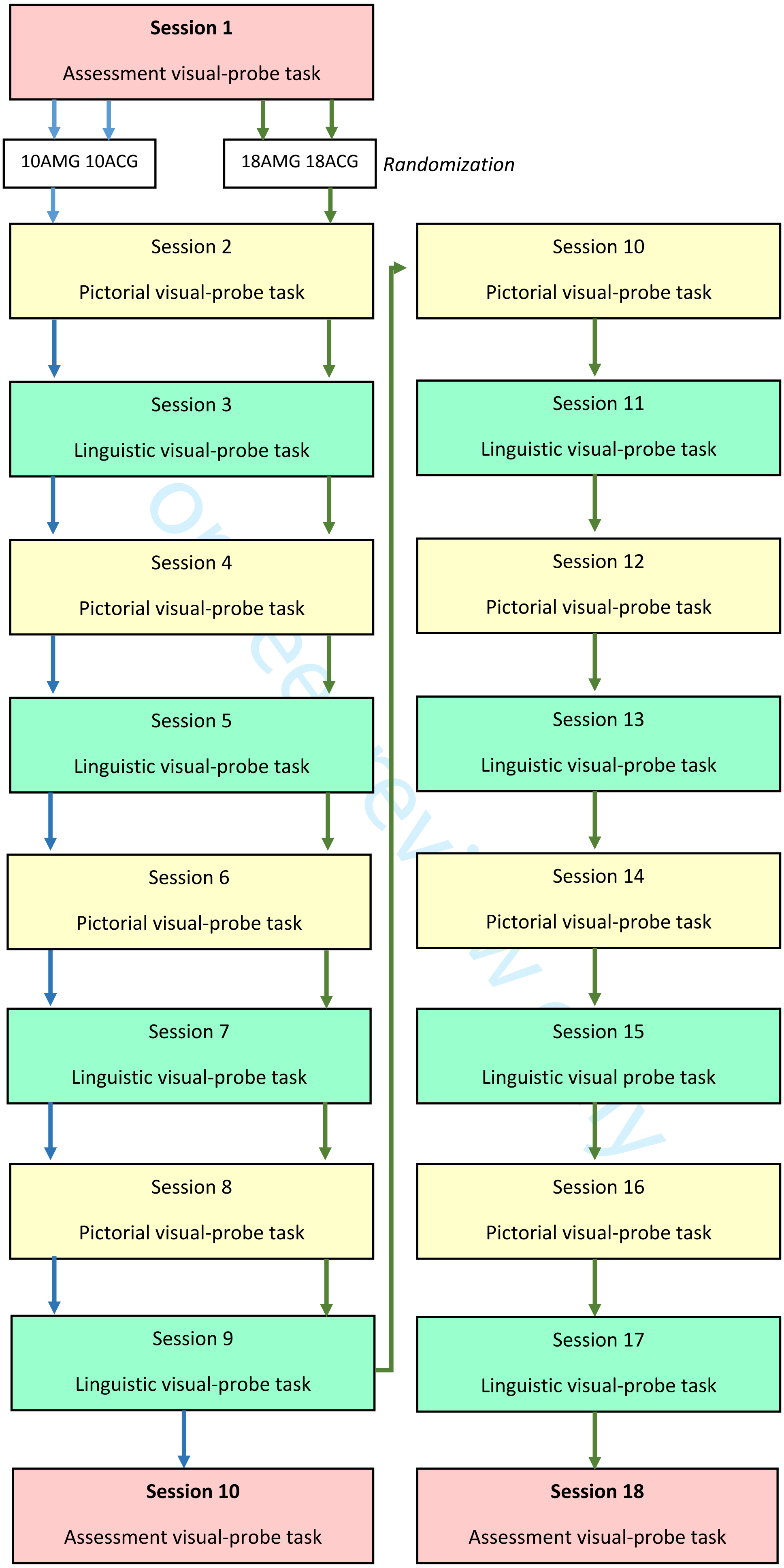

Figure 2. Flow of the visual-probe task sessions for the 10-week and 18-week groups in the study.

For peer review only - http://bmjopen.bmj.com/site/about/guidelines.xhtml

ATTENTIONAL BIAS MODIFICATION PROTOCOL

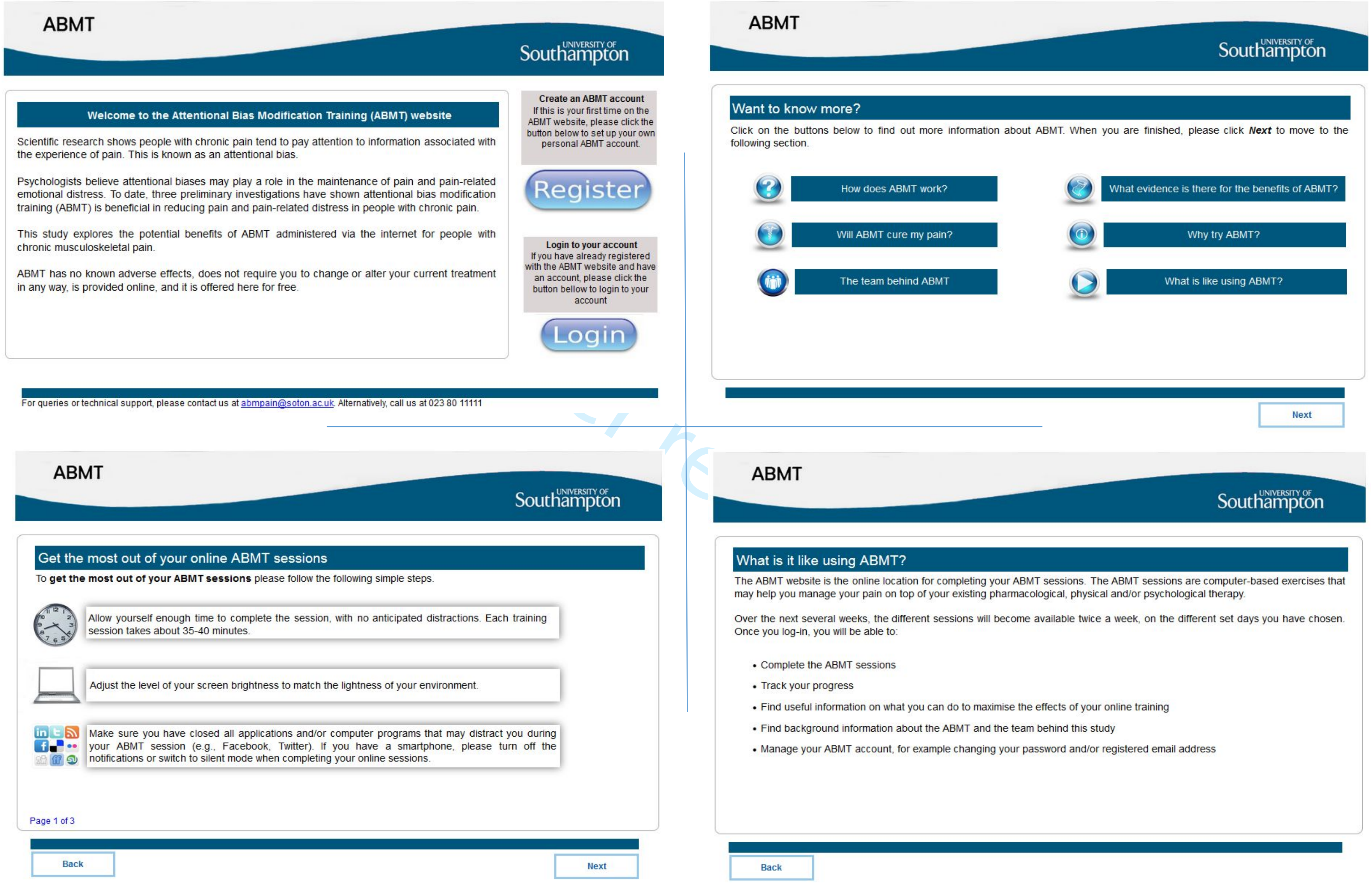

Figure 3. Examples of Lifeguide webpages included in the iABMT intervention.

Supplementary Material 1

Stimuli Development and Properties

A detailed description of the stimuli to be used in the iAMBT intervention is presented in this document. Linguistic stimulus properties and analyses on written frequency, valence, and arousal are first presented, followed by a description of pictorial stimuli properties and analyses of low-level features, valence, arousal and pain intensity. Following this, comparisons of stimuli properties between visual-probe sessions are then presented for linguistic and pictorial stimuli.

Linguistic stimuli

Properties. Linguistic and pictorial versions of the visual-probe task will be used. Each linguistic visual-probe task will include 32 threatening/neutral and 16 neutral/neutral word pairs. Threatening words stem from the following categories, with 8 words from each included in each visual-probe task: (i) sensory-pain words reflecting the sensory dimension of pain (e.g., *shooting*; *burning*; *aching*); (ii) affective-pain words reflecting the unpleasantness of pain and negative emotions associated with pain (e.g., *hopeless*, *punishing*, *tormenting*); (iii) health-threat words reflecting threat to health and well-being (e.g., *hospital, doctor*, *stress*); and (iv) general-threat words reflecting physical and emotional sources of threat, including manmade threat (e.g., *gun*), natural threat (e.g., *fire*), social threat (e.g., *humiliation*), and emotional threat (e.g., *sad*). Neutral words (including the neutral counterparts of the threatening words) include non-threatening words unrelated to pain or suffering (e.g., *version*; *rector*; *monument*), which were matched with their threatening counterparts on Kucera-Francis written frequency of use in the English language[1] and word length. Matching on written frequency minimise the chances of participants preferentially processing one stimulus due to its high frequency of use (e.g., faster recognition) when displayed simultaneously with another stimulus[2 3], while matching word length also minimise the chances of participants attending towards a stimulus due to its size.

In total, 864 words have been collected from previous studies exploring and modifying biases in pain and/or anxiety (i.e.,[4-8]), including words from the McGill Pain Questionnaire.[9]

For peer review only - http://bmjopen.bmj.com/site/about/guidelines.xhtml

ATTENTIONAL BIAS MODIFICATION PROTOCOL

Matching of threatening and neutral words was achieved via the MRC Psycholinguistic database[10], an online psycholinguistic tool that generates words with specific lexical characteristics. The database includes a dictionary of 150,837 English words providing syntactic, semantic and orthographical properties of each word.[11] Valence and arousal can influence attentional allocation to external stimuli.[12 13] A computerised version of the SAM task[14] was therefore used to collect ratings on valence and arousal for each word in a sample of sixteen healthy, pain-free participants (M_{age} = 25.9, SD = 4.08, range: 20 – 33 years; 8 female). All words were randomly presented to participants for 3 seconds each. Following each word, two 9-point SAM scales were presented, one for valence (1 = low pleasure to 9 = high pleasure) and one for arousal (1 = low arousal to 9 = high arousal). As per former chronic pain attentional bias research (e.g.,[5 15 16]), stimuli categories were assessed but not matched on valence and arousal, as differences are expected to exist between categories.

Analyses and results: written frequency, valence, and arousal. Analyses comparing properties between the five word categories are presented here, while analyses comparing word properties between the nine linguistic visual-probe tasks are presented below. One-way ANOVA was conducted comparing Kucera-Francis written frequency (Table 1) across words in the five word categories, revealing no significant main effect, $F(4, 853) = 1.97$, $p = .10$.

A repeated-measures ANOVA was conducted comparing arousal ratings (Table 2) across the five word categories, revealing a significant main effect, $F(2, 25) = 18.78$, $p < .001$, $\eta p^2 = .56$. Pairwise comparisons revealed the neutral category had significantly lower arousal scores than sensory-pain (*Md* [mean difference] = 1.82, *SE* = 0.45, *p* = .001), affective-pain (*Md* = 2.36, *SE*= 0.46, *p* = .001), general-threat (*Md* = 2.20, *SE* = 0.47, *p* = .003) and health-threat (*Md* = 2.05, *SE* = 0.35, *p* < .001) categories. Statistically significant differences were not found between the arousal scores of the words included in the four threat-related categories.

For peer review only - http://bmjopen.bmj.com/site/about/guidelines.xhtml

Table 1

Mean Kucera-Francis Written Frequency per Word Category

Word category	*M*	*SD*
Sensory-pain	19.07	27.01
Affective-pain	17.28	20.63
General-threat	25.00	32.10
Health-threat	21.36	36.39
All neutral words	22.60	27.97

Table 2

Mean Arousal and Valence Ratings per Word Category

	Arousal		Valence	
Word Category	*M*	*SD*	*M*	*SD*
Sensory-pain	3.80	1.85	3.95	0.83
Affective-pain	4.34	1.64	3.29	0.73
General-threat	4.19	1.78	3.35	0.79
Health-threat	4.03	1.32	3.48	0.68
Sensory-pain neutral counterparts	2.18	1.28	5.33	0.41
Affective-pain neutral counterparts	2.19	1.32	5.29	0.29
General-threat neutral counterparts	2.06	1.28	5.24	0.33
Health-threat neutral counterparts	2.05	1.19	5.19	0.36
Neutral/Neutral	1.95	1.32	5.29	0.33

A repeated-measures ANOVA was conducted comparing valence ratings (Table 2) across the five word categories, revealing a significant main effect, $F(2, 29) = 52.00$, $p < .001$, $\eta p^2 = .78$. Pairwise comparisons showed the neutral category had significantly higher pleasantness scores than sensory-pain (*Md* = 1.33, *SE* = 0.23, *p* <. 001) affective-pain (*Md* = 1.99, *SE* = 0.22, *p* < .001), general-threat (*Md* = 1.93, *SE* = 0.23, *p* <. 001) and health-threat (*Md* = 1.80, *SE* = 0.32, *p* < .001) categories. The words in the sensory-pain category were rated as more pleasant than

For peer review only - http://bmjopen.bmj.com/site/about/guidelines.xhtml

For peer review only

words included in the affective-pain (*Md* = 0.66, *SE* = 0.11, $p < .001$), general-threat (*Md* = 0.60, *SE* = 0.11, $p < .001$) and health-threat (*Md* = 0.47, *SE* = 0.11, $p = .01$) categories.

Pictorial Stimuli

Properties. Pictorial visual-probe tasks each include 32 threatening/neutral and 16 neutral/neutral image pairs. Threatening images stem from the following categories: (i) musculoskeletal pain images depicting people experiencing pain in muscles and/or joints, including images of people performing daily activities that can be considered painful and/or harmful for individuals with CMP (e.g., a person running; a person lifting a heavy weight with poor posture); (ii) facial expressions of pain, featuring a single model depicting a prototypical pain expression – closing the eyes or narrowing the eyelids, lowering the eyebrows, wrinkling the nose or raising the cheek, and partly opening the mouth or extending the lips[17], including horizontal lip stretching and/or oblique lip raising[18]; (iii) health-threat images reflecting threat to health and well-being (e.g., medication, wheelchair); and (iv) general-threat images reflecting physical and emotional sources of threat, including manmade threat (e.g., gun), natural threat (e.g., flood), and social threat (e.g., social isolation). Neutral images (including the neutral pairs of the threatening images) depict non-threatening objects (e.g., a car) and people performing every-day activities (e.g., a person reading a newspaper). In total, 768 images have been collected from previous studies exploring and modifying attentional biases in chronic pain (i.e.,[5] [19]), including online databases providing batteries of pictorial stimuli for research (i.e., International Affective Picture System database[20]; The Photograph Series of Daily Activities Short Version[21]; NimStim Set of Facial Expressions[22]).

Each threatening image depicting a person was paired with a neutral image featuring the same person, with the same clothes and background. Each threatening image depicting an object was matched with a neutral image depicting a similar, non-threatening object. Image pairs in the neutral/neutral condition were matched in the same manner. An equal number of images depicting male and female models were used within each image category. All images were full-

For peer review only

colour and the same size (i.e., 265 pixels wide x 250 pixels high). Each threatening image was matched with its neutral counterpart on complexity, luminance, contrast level, and colour saturation in blue, red and green channels via visual inspection[23]. Visual attention and gaze fixations are influenced by bottom-up variables, including the saliency of objects[24] and low-level features of visual scenes.[25 26] The additional matching rules for pictorial stimuli in the present study were therefore used to minimise the chances of participants preferentially processing one stimulus due to unnecessary variations in content. Using Adobe Photoshop CS6 Extended, the means and standard deviations for all parameters were extracted for evaluation. Luminance values range from 0 to 255, where 0 indicates no light intensity (i.e., no light emission; black) and 255 indicates the highest light intensity (i.e., absolute light emission; white). Contrast levels values range from -100 to +100, with values closer to +100 indicating more contrasted image. The intensity values for red, green and blue colours range from 0 to 255, where 0 indicates black (i.e., darkest extreme) and 255 white (i.e., lightest extreme) for each colour dimension.

Visual complexity was examined as the size of each compressed image file (.jpeg) in kilobytes under the assumption that more complex images would have higher values in kilobytes.[27] As with linguistic stimuli, a computerised version of the SAM task[14] was used to collect ratings on valence and arousal for each image. Musculoskeletal and facial expressions of pain categories were also evaluated on pain intensity using a 10 cm Visual Analogue Scale (VAS). Participants were required to rate the pain that the person in the image experienced. The scale ranged from 0 to 10, where 0 indicated '*no pain at all*' and 10 indicated '*excruciating pain*'. This rating aimed to assess the degree to which images included in the pain-related categories were perceived as depicting pain. Sixteen healthy, pain-free participants (M_{age} = 24.8, SD = 4.56, range: 20 – 33 years; 8 female) were recruited to provide ratings on valence, arousal, and pain intensity (this was a separate sample to that recruited to provide linguistic stimuli ratings).

Analyses and results: Low-level features. Analyses comparing properties between the five image categories on low-level features (Table 3) are presented here, while analyses

For peer review only - http://bmjopen.bmj.com/site/about/guidelines.xhtml

comparing word properties between the eight pictorial visual-probe tasks are provided below. Considering low-level features, one-way ANOVAs revealed no significant main effects for complexity, $F(4,763) = 2.18$, $p = .70$, or luminance, $F(4,763) = 2.31$, $p = .06$. A significant main effect was found for red saturation, $F(4,763) = 5.19$, $p < .001$, $\eta p^2 = .03$. Pairwise comparisons revealed images in the musculoskeletal-pain category had significantly less red saturation than images in the facial expressions of pain (*Md* = 24.24, *SE* = 7.89, $p = .022$) and neutral categories (*Md* = 19.04, *SE* = 5.91, $p = .013$). Images in the general-threat category had significantly less red saturation than facial expressions of pain (*Md* = 23.96, *SE* = 7.89, $p = .025$) and neutral categories (*Md* = 18.76, *SE* = 5.91, $p = .016$). A significant main effect was found for blue saturation, $F(4,763) = 2.74$, $p = .03$, $\eta p^2 = .01$. Images in the general-threat categories had significantly less blue saturation than images in the neutral category (*Md* = 29.64, *SE* = 9.96, $p = .030$). A significant main effect was found for green saturation, $F(4, 763) = 4.19$, $p = .002$, $\eta p^2 = .02$. Images in the general-threat expressions category had significantly less green saturation than images in the neutral category (*Md* = 22.03, *SE* = 6.23, $p = .004$). A significant main effect was found for RMS contrast levels, $F(4,763) = 5.98$, $p < .001$, $\eta p^2 = .03$. Images in the facial expressions of pain category had significantly higher RMS contrast than the images included in musculoskeletal-pain (*Md* = 9.57, *SE* = 3.32, $p = .040$), health-threat (*Md* =13.77, *SE* = 3.32, $p < .001$) and neutral categories (*Md* = 10.89, *SE* = 2.49, $p < .001$).

For peer review only

ATTENTIONAL BIAS MODIFICATION PROTOCOL

Table 3

Mean Low-Level values per Image Category

	Musculoskeletal-pain		Facial Expressions of Pain		General-threat		Health-threat		All neutral stimuli	
	M	*SD*	*M*	*SD*	*M*	*SD*	*M*	*SD*	*M*	*SD*
Complexity	20.03	7.17	15.69	3.10	20.14	6.36	19.30	10.78	18.62	10.92
Red Saturation	148.72	41.35	172.95	22.79	148.99	43.43	164.37	57.07	167.76	45.41
Green Saturation	140.39	41.39	153.81	27.56	132.80	41.82	154.92	58.08	154.84	48.60
Blue Saturation	132.53	43.55	144.86	31.64	119.90	48.00	149.09	61.87	149.54	85.49
Luminance	142.01	41.10	158.57	25.50	136.21	41.39	157.37	57.10	162.79	92.44
RMS contrast level	61.25	15.3	70.82	18.75	64.22	18.95	57.00	20.91	59.93	18.86

Analyses and results: Valence, arousal and pain intensity. A repeated-measures ANOVA was conducted comparing arousal ratings (Table 4) across the five image categories, revealing a significant main effect, $F(2,23) = 32.79$, $p < .001$, $\eta p^2 = .69$. Pairwise comparisons revealed that the neutral category had significantly lower arousal scores than facial expressions of pain (*Md* = 2.24, *SE* = 0.45, *p* = .002), musculoskeletal-pain (*Md* = 1.28, *SE* = 0.33, *p* = .014), general-threat (*Md* = 2.86, *SE* = 0.38, *p* < .001), and health-threat (*Md* = 1.91, *SE* = 0.34, *p* = .001) categories. The general-threat category was significantly more arousing than facial expressions of pain (*Md* = 0.62, *SE* = 0.15, *p* = .010), musculoskeletal-pain (*Md* = 1.59, *SE* = 0.14, *p* < .001), and health-threat categories (*Md* = 0.95, SE =0.15, *p* < .001). The musculoskeletal-pain category was also significantly less arousing than the facial expressions of pain (*Md* = 0.96, *SE* = 0.15, *p* < .001) and the health-threat (*Md* = 0.64, *SE* = 0.14, *p* = .005) categories.

For peer review only - http://bmjopen.bmj.com/site/about/guidelines.xhtml

ATTENTIONAL BIAS MODIFICATION PROTOCOL

A repeated-measures ANOVA conduced on valence scores (Table 4) across the five image conditions revealed a significant main effect, $F(2,29) = 64.52$, $p < .001$, $\eta p^2 = .82$. Pairwise comparisons showed the neutral category had significantly higher pleasantness scores than facial expressions of pain (*Md* = 1.56, *SE* = 0.15, $p <. 001$), musculoskeletal-pain (*Md* = 0.86, *SE* = 0.13, $p < .001$), general-threat (*Md* = 1.69, *SE* = 0.16, $p<. 001$) and health-threat (*Md* = 1.27, *SE* = 0.18, $p< .001$) categories. Images in the musculoskeletal-pain category were rated significantly more pleasant than the images included in the facial expressions of pain (*Md* = 0.69, *SE* = 0.07, $p < .001$), general-threat (*Md* = 0.83, *SE* = 0.08, $p < .001$) and health-threat (*Md* = 0.41, *SE* = 0.09, $p= .003$) categories. Images in the health-threat category had significantly higher pleasantness scores than images included in the general-threat category (*Md* = 0.42, *SE* = 0.07, $p < .001$).

An independent samples *t*-test was conducted comparing pain intensity scores (Table 4) between the images in the musculoskeletal pain and facial expressions of pain categories. There was a significant difference in the pain intensity scores of musculoskeletal-pain (*M* = 3.35, *SD* = 2.27) and facial expressions of pain (*M* = 5.15, *SD* = 2.13) categories; $t(245) = 6.520$, $p < .001$, $r = 0.38$. This result suggests images included in the facial expressions of pain category had significantly higher pain intensity ratings than those included in the musculoskeletal-pain category.

For peer review only - http://bmjopen.bmj.com/site/about/guidelines.xhtml

Table 4

Mean Arousal, Valence and Pain Intensity Ratings per Image Category

	Arousal		Valence		Pain Intensity	
Image Category	*M*	*SD*	*M*	*SD*	*M*	*SD*
Musculoskeletal-pain	2.85	1.45	4.20	0.57	3.35	2.09
Facial expressions of pain	3.81	1.92	3.50	0.66	5.15	2.02
General-threat	4.43	1.69	3.37	0.62		
Health-threat	3.48	1.53	3.79	0.71		
Musculoskeletal-pain neutral counterparts	1.82	0.68	5.11	0.22		
Facial expressions of pain neutral counterparts	1.74	0.69	5.00	0.14		
General-threat neutral counterparts	1.82	0.68	5.08	0.17		
Health-threat neutral counterparts	1.75	0.54	5.04	0.24		
Neutral/Neutral	1.57	0.49	5.10	0.15		

For peer review only

Comparison between Visual-Probe Sessions – Linguistic Stimuli

Differences in Arousal Scores between Threatening Word Categories across the Nine Linguistic Visual Probe Sessions

A 4 (word category; sensory-pain, affective-pain, general-threat, health-threat) x 9 (linguistic visual probe session; session 1, 3, 5, 7, 9, 11, 13, 15, 17) ANOVA was conducted to compare the arousal scores of the threatening word categories across the 9 linguistic visual probe task sessions. The results showed a significant main effect of session, $F(8, 120) = 8.22$, $p < .001$, $\eta_p^2 = .35$, and a significant word category x session interaction, $F(24, 360) = 3.05$, $p < .001$, $\eta_p^2 = .17$.

Post-hoc analyses across sessions. To explore the significant interaction, a series of one-way ANOVAs with 9 levels (Session: 1, 3, 5, 7, 9, 11, 13, 15, 17) were run for each threatening word category independently. These explored whether the arousal ratings of each threatening word category differed across the sessions. For the sensory-pain words, a main effect of session was found, $F(5,67) = 6.02$, $p < .001$, $\eta_p^2 = .29$. Pairwise comparisons revealed sensory words to be significantly more arousing in session 1 than session 9 ($Md = 0.92$, $SE = 0.22$, $p = .03$), and significantly more arousing in session 17 than session 9 ($Md = 0.75$, $SE = 0.17$, $p = .02$) and session 15 ($Md = 0.59$, $SE = 0.11$, $p = .003$).

For the affective-pain words, a main effect of session was found, $F(8,120) = 4.26$, $p < .001$, $\eta_p^2 = .22$. Pairwise comparisons revealed these words to be significantly more arousing in session 1 than in session 5 ($Md = 0.57$, $SE = 0.12$, $p = .01$) and session 13 ($Md = 1.04$, $SE = 0.26$, $p = .05$).

For the general-threat words, a main effect of session was found, $F(8, 120) = 4.91$, $p < .001$, $\eta_p^2 = .25$. Pairwise comparisons revealed these words to be significantly more arousing in session 1 than session 5 ($Md = 0.65$, $SE = 0.13$, $p = .01$), session 7 ($Md = 0.88$, $SE = 0.17$, $p = .004$) and session 15 ($Md = 0.84$, $SE = 0.19$, $p = .02$).

For peer review only - http://bmjopen.bmj.com/site/about/guidelines.xhtml

For the health-threat words, a main effect of session was found, $F(8,120) = 4.08$, $p < .001$, $\eta_p^2 = .21$. Pairwise comparisons revealed these words to be significantly more arousing in session 1 than session 11 ($Md = 1.05$, $SE = 0.18$, $p = .001$).

Post-hoc analyses within sessions. To further explore the significant interaction, a series of one-way ANOVAs with 4 levels (word category; sensory-pain, affective-pain, general-threat and health-threat) were conducted for each session independently. These explored whether the arousal ratings of each threatening word category differed within each linguistic visual-probe session. No significant main effects of word category were found for session 1, $F(3,45) = 1.16$, $p = .20$, $\eta_p^2 = .10$, session 5, $F(3,45) = 2.34$, $p = .86$, $\eta_p^2 = .14$, session 13, $F(3,45) = 0.70$, $p = .06$, $\eta_p^2 = .05$, and session 17, $F(3,45) = 2.27$, $p = .09$, $\eta_p^2 = .13$.

A significant main effect of word category was found in session 3, $F(3,45) = 4.31$, $p = .01$, $\eta_p^2 = .22$. Pairwise comparisons revealed no significant differences between the word categories of this session, however.

A significant main effect of word category was found in session 7, $F(3,45) = 3.75$, $p = .02$, $\eta_p^2 = .20$. Pairwise comparisons revealed no significant differences between the four threat-related categories.

A significant main effect of word category was found in session 9, $F(3,45) = 5.22$, $p = .004$, $\eta_p^2 = .26$. Pairwise comparisons revealed that sensory-pain words were significantly less arousing than affective-pain ($Md = 0.84$, $SE = 0.21$, $p = .01$), general-threat ($Md = 0.77$, $SE = 0.23$, $p = .03$) and health-threat categories ($Md = 0.92$, $SE = 0.30$, $p = .05$).

A significant main effect of word category was found in session 11, $F(3,45) = 4.45$, $p = .01$, $\eta_p^2 = .23$. Pairwise comparisons showed that the general-threat words were significantly more arousing than the health-threat words ($Md = 0.61$, $SE = 0.19$, $p = .04$).

A significant main effect of word category was found in session 15, $F(3,45) = 2.86$, $p = .05$, $\eta_p^2 = .16$. Pairwise comparisons revealed no significant differences between the four threat-related categories.

For peer review only - http://bmjopen.bmj.com/site/about/guidelines.xhtml

Differences in Valence Scores between Threatening Word Categories across the nine Linguistic Visual Probe Sessions

A 4 (word category; sensory-pain, affective-pain, general-threat and health-threat) x 9 (linguistic visual probe sessions; 1, 3, 5, 7, 9, 11, 13, 15, and 17) ANOVA was conducted to compare the pleasantness scores of the threatening categories across the 9 linguistic visual probe task sessions. The results showed significant main effects of word category, $F(3,45) = 15.86$, $p < .001$, $\eta_p^2 = .51$, and session, $F(8,120) = 11.90$, $p < .001$, $\eta_p^2 = .44$, and a significant word category x session interaction, $F(24,360) = 2.57$, $p < .001$, $\eta_p^2 = .15$.

Post-hoc analyses across sessions. To explore the significant interaction, a series of one-way ANOVAS with 9 levels (Session; 1, 3, 5, 7, 9, 11, 13, 15, 17) were run for each word category independently. These explored whether the pleasantness ratings for each word category differed across the sessions. For the sensory-pain words, a main effect of session was found, $F(5,75) = 6.88$, $p < .001$, $\eta_p^2 = .31$. Pairwise comparisons revealed sensory words to be significantly less pleasant in session 1 than session 3 (*Md* = 0.49, *SE* = 0.12, *p* =.04), session 7 (*Md* = 0.59, *SE* = 0.11, *p* = .003) and session 9 (*Md* = 0.73, *SE* = 0.09, *p* < .001). Sensory-pain words were also significantly less pleasant in session 17 than session 5 (*Md* = 0.26, *SE* = 0.05, *p* = .01) and session 9 (*Md* = 0.49, *SE* = 0.09, *p* = .003).

For the affective-pain words, a main effect of session was found, $F(8,120) = 4.86$, $p < .001$, $\eta p^2 = .25$. Pairwise comparisons revealed these words to be significantly less pleasant in session 1 than session 5 (*Md* = 0.51, *SE* = 0.11, *p* = .01), session 7 (*Md* = 0.47, *SE* = 0.09, *p* = .01), session 13 (*Md* = 0.59, *SE* = 0.11, *p* = .004) and session 15 (*Md* = 0.44, *SE* = 0.11, *p* = .04).

For the general-threat words, a main effect of session was found, $F(8,120) = 9.64$, $p < .001$, $\eta p^2 = .39$. Pairwise comparisons revealed these words to be significantly less pleasant in session 1 than session 5 (*Md* = 0.63, *SE* = 0.09, *p* < .001), session 7 (*Md* = 0.65, *SE* = 0.11, *p* = .001), session 9 (*Md* = 0.44, *SE* = 0.07, *p* < .001), session 13 (*Md* = 0.33, *SE* = 0.06, *p* = .001) and session 15 (*Md* = 0.65, *SE* = 0.12, *p* = .002). Significantly less pleasant words were also found in

For peer review only - http://bmjopen.bmj.com/site/about/guidelines.xhtml

session 3 than session 5 (*Md* = 0.37, *SE* = 0.09, $p = .03$) and session 7 (*Md* = 0.39, *SE* = 0.08, $p = .01$). Session 17 included significantly less pleasant words than session 7 (*Md* = 0.41, *SE* = 0.10, $p = .03$).

For the health-threat words, a main effect of session was found, $F(4,58) = 2.90$, $p = .03$, $\eta_p^2 = .16$. Pairwise comparisons revealed these words to be significantly less pleasant in session 1 than session 11 (*Md* = 0.57, *SE* = 0.14, $p = .03$).

Post-hoc analyses within sessions. To further explore the significant interaction, one-way ANOVAs with 4 levels (word category; sensory-pain, affective-pain, general-threat and health-threat) were conducted for each session independently. These explored whether pleasantness ratings for each threatening word category differed within each session. For session 1, a significant main effect was found, $F(3,45) = 4.84$, $p = .01$, $\eta_p^2 = .24$. Pairwise comparisons revealed that sensory-pain words were significantly more pleasant than affective words (*Md* = 0.63, *SE* = 0.16, $p = .01$).

For session 3, a significant main effect was found, $F(3,45) = 12.71$, $p < .001$, $\eta_p^2 = .46$. Pairwise comparisons showed that sensory-pain words were significantly more pleasant than affective-pain (*Md* = 0.73, *SE* = .15, $p < .001$), general-threat (*Md* = 0.79, *SE* = 0.13, $p < .001$) and health-threat words (*Md* = 0.56, *SE* = 0.15, $p = .01$).

For session 5, a significant main effect was found, $F(3,45) = 6.45$, $p <.001$, $\eta_p^2 = .30$. Pairwise comparisons revealed that sensory-pain words were significantly more pleasant than the affective-pain words (*Md* = 0.62, *SE* = 0.15, $p = .01$).

For session 7, a significant main effect was found, $F(3,45) = 10.19$, $p < .001$, $\eta_p^2 = .41$. Pairwise comparisons revealed that sensory-pain words were significantly more pleasant than the affective-pain words (*Md* = 0.17, *SE* = 0.13, $p < .001$), the general-threat words (*Md* = 0.51, *SE* = 0.09, $p < .001$) and the health-threat words (*Md* = 0.55, *SE* = 0.16, $p = .02$).

For session 9, a significant main effect was found, $F(2,31) = 13.67$, $p < .001$, $\eta_p^2 = .48$. Pairwise comparisons revealed that the sensory-pain words were significantly more pleasant than

For peer review only - http://bmjopen.bmj.com/site/about/guidelines.xhtml

the affective-pain words (*Md* = 0.98, *SE*= 0.16, $p < .001$), the general-threat words (*Md* = 0.87, *SE* = 0.15, $p < .001$) and the health-threat words (*Md* = 0.92, *SE* = 0.21, $p < .001$).

For session 11, a significant main effect was found, $F(3,45) = 11.63$, $p < .001$, $\eta_p^2 = .44$. Pairwise comparisons revealed that the sensory-pain words were significantly more pleasant than the affective-pain (*Md* = 0.69, *SE* = 0.13, $p < .001$) and the general-threat words (*Md* = 0.70, *SE* = 0.17, $p < .001$). Also, the affective-pain words were significantly less pleasant than the health-threat words (*Md* = 9.39, *SE* = 0.13, $p = .04$).

For peer review only

For session 13, a significant main effect was found, $F(3,45) = 4.43$, $p = .01$, $\eta_p^2 = .23$. Pairwise comparisons revealed that the sensory-pain words were significantly more pleasant than the general-threat words (*Md* = 0.56, *SE* = 0.12, $p < .001$).

For session 15, a significant main effect was found, $F(3,45) = 8.40$, $p < .001$, $\eta_p^2 = .36$. Pairwise comparisons showed that the sensory-pain words were significantly more pleasant than the words included in the affective (*Md* = 0.61, *SE* = 0.12, $p < .001$) and the health-threat words (*Md* = 0.71, *SE* = 0.19, $p = .01$).

For session 17, a significant main effect was found, $F(3,45) = 6.78$, $p<.001$, $\eta_p^2 = 0.36$. ${}_p^2 = 0.31$. Pairwise comparisons revealed that the sensory-pain words were significantly more pleasant than affective-pain (*Md* = 0.56, *SE* = 0.15, $p = .01$) and the general-threat words (*Md* = 0.57, *SE* = 0.15, $p = .01$).

Comparison between Visual-Probe Sessions – Pictorial Stimuli

Differences in Arousal Scores between Threatening Image Categories across the eight Pictorial Visual Probe Sessions

A 4 (image category; musculoskeletal-pain, facial expressions of pain, general-threat, health-threat) x 8 (pictorial visual probe sessions; 2, 4, 6, 8, 10, 12, 14, 16) ANOVA was conducted to compare the arousal scores of the threatening image categories across the 8 pictorial visual probe task sessions. The results showed a significant main effect of image category, $F(3,45) = 33.52$, *p*

< .001, η_p^2 = .69, and a significant image category x session interaction, $F(7,98) = 9.84$, $p< .001$, η_p^2 = .40.

Post-hoc analyses within image categories. To explore the significant interaction, a series of one-way ANOVAs with 8 levels (Session; 2, 4, 6, 8, 10, 12, 14, 16) were run for each threatening image category independently. These explored whether the arousal ratings of each threat-related image category differed across the sessions. For the images in the musculoskeletal-pain category, no main effect of session was found, $F(2,31) = 2.49$, $p = .10$, $\eta_p^2 = .14$. For the images in the facial expressions of pain category, no main effect of session was found, $F(3,46) = 0.77$, $p = .50$, $\eta_p^2 = .05$.

For the images in the general-threat category, a main effect of session was found, $F(7, 105) = 7.40$, $p < .001$, $\eta_p^2 = .33$. Pairwise comparisons revealed these images to be significantly less arousing in session 4 than session 6 (*Md* = 0.70, *SE* = 0.17, $p = .023$), session 12 (*Md* = 0.73, *SE* = 0.19, $p = .04$) and session 16 (*Md* = 0.82, *SE* = 0.21, $p = .04$). These images were also found to be significantly less arousing in session 14 than session 6 (*Md* = 0.93, *SE* = 0.19, $p = .006$), session 12 (*Md* = 0.96, *SE* = 0.17, $p = .001$) and session 16 (*Md* = 1,05, *SE* = 0.17, $p = .001$).

For the images in the health-threat category, a main effect of session was found, $F(7, 105) = 13.10$, $p < .001$, $\eta_p^2 = .47$. Pairwise comparisons revealed these images to be significantly more arousing in session 10 than session 8 (*Md* = 1.15, *SE* = 0.20, $p = .001$) and session 12 (*Md* = 0.88, *SE* = 0.20, $p = .02$). Health-threat images were also found to be significantly less arousing in session 16 than session 2 (*Md* = 0.97, *SE* = 0.17, $p = .001$), session 4 (*Md* = 1.34, *SE* = 0.26, $p = .004$), session 6 (*Md* = 1.32, *SE* = 0.29, $p = .009$), session 10 (*Md* = 1.91, *SE* = 0.28, $p< .001$), session 12 (*Md* = 1.03, *SE* = 0.26, $p = .04$) and session 14 (*Md* = 1.45, *SE* = 0.9, $p< .001$).

Post-hoc analyses within sessions. To further explore the significant interaction, a series of one-way ANOVAs with 4 levels (image category; musculoskeletal-pain, facial expressions of pain, general-threat and health-threat) were conducted for each session independently. These

For peer review only - http://bmjopen.bmj.com/site/about/guidelines.xhtml

explored whether the arousal ratings of each threatening image category differed within each pictorial visual-probe session.

A significant main effect of image category was found in session 2, $F(3,45) = 23.27$, $p < .001$, $\eta_p^2 = .61$. Pairwise comparisons revealed that the images in the facial expressions of pain category were more arousing than those included in the musculoskeletal-pain category ($Md = 0.98$, $SE = 0.27$, $p = .014$). The images in the general-threat category were also found to be significantly more arousing than those included in the musculoskeletal-pain ($Md = 2.05$, $SE = 0.32$, $p < .001$), facial expressions of pain ($Md = 1.07$, $SE = 0.19$, $p < .001$) and health-threat categories ($Md = 1.34$, $SE = 0.24$, $p < .001$).

A significant main effect of image category was found in session 4, $F(3,45) = 8.27$, $p < .001$, $\eta_p^2 = .36$. Pairwise comparisons revealed that the images in the musculoskeletal-pain category were significantly less arousing than the images in the facial expressions of pain ($Md = 0.76$, $SE = 0.16$, $p = .001$), health-threat ($Md = 1.10$, $SE = 0.23$, $p = .002$) and general-threat categories ($Md = 0.70$, $SE = 0.22$, $p = .04$).

A significant main effect of image category was found in session 6, $F(3,45) = 26.77$, $p < .001$, $\eta_p^2 = .64$. Pairwise comparisons revealed that the images in the musculoskeletal-pain category were significantly less arousing than the images included in the facial expressions of pain ($Md = 1.17$, $SE = 0.25$, $p = .002$), general-threat ($Md = 2.06$, $SE = 0.23$, $p < .001$) and health-threat categories ($Md = 0.97$, $SE = 0.25$, $p = .009$). The images in the general-threat category were significantly more arousing than those in the facial expressions of pain ($Md = 0.89$, $SE = 0.21$, $p = .003$) and health-threat categories ($Md = 1.09$, $SE = 0.21$, $p = .001$).

A significant main effect of image category was also found in session 8, $F(2,32) = 10.56$, $p < .001$, $\eta_p^2 = .41$. Pairwise comparisons revealed that the images in the musculoskeletal-pain category were significantly less arousing than those included in the facial expressions of pain ($Md = 0.89$, $SE = 0.22$, $p = .006$) and general-threat categories ($Md = 1.13$, $SE = 0.25$, $p = .003$).

For peer review only

For peer review only - http://bmjopen.bmj.com/site/about/guidelines.xhtml

The images in general-threat categories were significantly more arousing that those included in the health-threat category (*Md* = 1.00, *SE* = 0.20, p = .001).

A significant main effect of image category was found in session 10, $F(3,45) = 17.37$, $p < .001$, $\eta_p^2 = .54$. Pairwise comparisons revealed that the images in the musculoskeletal-pain category were significantly less arousing than those included in the general-threat (*Md* = 0.92, *SE* = 0.17, $p < .001$) and health-threat categories (*Md* = 1.23, *SE* = 0.20, $p < .001$). The images in the facial expressions of pain category were also significantly less arousing than those included in the health-threat category (*Md* = 0.71, *SE* = 0.18, p = .009).

A significant main effect of image category was found in session 12, $F(3,45) = 18.40$, $p < .001$, $\eta_p^2 = .55$. Pairwise comparisons revealed that the images in the musculoskeletal-pain category were significantly less arousing that those included in the facial expressions of pain (*Md* = 1.61, *SE* = 0.18, p = .022) and general-threat categories (*Md* = 1.64, *SE* = 0.19, $p < .001$). The images included in the general-threat category were significantly more arousing than the images included in the facial expressions of pain (*Md* = 1.03, *SE* = 0.24, p = .001) and health-threat categories (*Md* = 1.03, *SE* = 0.26, $p < .001$).

A significant main effect of image category was found in session 14, $F(3,45) = 14.72$, $p < .001$, $\eta_p^2 = .50$. Pairwise comparisons revealed than the images in the musculoskeletal-pain category were significantly less arousing than those included in facial expressions of pain (*Md* = 1.39, *SE* = 0.30, p = .002), general-threat (*Md* = 1.41, *SE* = 0.25, $p < .001$) and health-threat categories (*Md* = 1.35, *SE* = 0.20, $p < .001$).

A significant main effect of image category was found in session 16, $F(2,28) = 38.74$, $p < .001$, $\eta_p^2 = .72$. Pairwise comparisons revealed that the images in the musculoskeletal-pain category were significantly less arousing than the images included in the facial expressions of pain (*Md* = 1.38, *SE* = 0.29, p = .001) and general-threat categories (*Md* = 2.37, *SE* = 0.27, p = .002). Images included in the facial expression of pain category were also found to be significantly less arousing than those included in the general-threat category (*Md* = 0.98, *SE* =

For peer review only - http://bmjopen.bmj.com/site/about/guidelines.xhtml

0.21, p = .002). Images in the health-threat category were significantly less arousing than the images included in the facial expression of pain (Md = 1.57, SE = 0.36, p = .004) and general-threat categories (Md = 2.56, SE = 0.29, p < .001).

Differences in Valence Scores between Threatening Pictorial Categories across the eight Pictorial Visual Probe Sessions

A 4 (image category; musculoskeletal-pain, facial expressions of pain, general-threat and health-threat) x 8 (pictorial visual probe sessions; 2, 4, 6, 8, 10, 12, 14, 16) ANOVA was conducted to compare the pleasantness scores of the threatening categories across the 8 pictorial visual probe task sessions. The results showed a significant main effect of image category, $F(3,45) = 41.09, p < .001, \eta_p^2 = .73$, and a significant image category x session interaction, $F(7,102) = 9.33, p < .001, \eta_p^2 = .38$.

Post-hoc analyses within image categories. To explore the significant interaction, a series of one-way ANOVAS with 8 levels (Session; 2, 4, 6, 8, 10, 12, 14, 16) were run for each image category independently. These explored whether the pleasantness ratings for each image category differed across the sessions.

For the images included in the musculoskeletal-pain category, a main effect of session was found, $F(2,32) = 3.70, p = .033, \eta_p^2 = .20$. Pairwise comparisons revealed these images to be significantly more pleasant in session 2 than session 4 (Md = 0.54, SE = 0.11, p = .007).

For the images included in the facial expressions of pain category, a main effect of session was found, $F(7,105) = 3.44, p = .002, \eta_p^2 = .19$. Pairwise comparisons revealed these images to be significantly more pleasant in session 10 than session 6 (Md = 0.51, SE = 0.13, p = .03) and session 16 (Md = 0.43, SE = 0.09, p = .005).

For the images included in the general-threat category, a main effect of session was found, $F(7,105) = 6.84, p < .001, \eta_p^2 = .31$. Pairwise comparisons revealed these images to be significantly more pleasant in session 10 than session 6 (Md = 0.61, SE = 0.13, p = .009), session 12 (Md = 0.56, SE = 0.14, p = .05) and session 16 (Md = 0.68, SE = 0.14, p = .007). General-

For peer review only - http://bmjopen.bmj.com/site/about/guidelines.xhtml

threat images were significantly more pleasant in session 14 than session 6 (*Md* = 0.60, *SE* = 0.14, p = .019), session 12 (*Md* = 0.55, *SE* = 0.12, p = .013) and session 16 (*Md* = 0.67, *SE* = 0.11, p = .001). Images in this category were also significantly more pleasant in session 4 than session 6 (*Md* = 0.45, *SE* = 0.11, p = .04) and session 16 (*Md* = 0.52, *SE* = 0.11, p = .007).

For the images included in the health-threat category, a main effect of session was found, $F(7,105) = 9.87$, $p < .001$, $\eta_p^2 = .40$. Pairwise comparisons revealed these images to be significantly more pleasant in session 8 than session 10 (*Md* = 0.73, *SE* = 0.10, p = .005). Images in this category were significantly more pleasant in session 16 than session 2 (*Md* = 0.54, *SE* = 0.11, p = .004), session 4 (*Md* = 0.62, *SE* = 0.12, p = .003), session 6 (*Md* = 0.52, *SE* = 0.12, p = .02), session 10 (*Md* = 1.00, *SE* = 0.10, $p < .001$), session 12 (*Md* = 0.56, *SE* = 0.12, p = .008) and session 14 (*Md* = 0.80, *SE* = 0.10, $p < .001$).

Post-hoc analyses within sessions. To further explore the significant interaction, one-way ANOVAs with 4 levels (image category; musculoskeletal-pain, facial expressions of pain, general-threat and health-threat) were conducted for each session independently. These explored whether pleasantness ratings for each image category differed within each session.

For session 2, a significant main effect was found, $F(3,45) = 13.36$, $p < .001$, $\eta_p^2 = .47$. Pairwise comparisons revealed that images in the musculoskeletal-pain category were significantly more pleasant than images included in the facial expressions of pain (*Md* = 1.00, *SE* = 0.18, $p < .001$), general-threat (*Md* = 0.98, *SE* = 0.20, p = .001) and health-threat categories (*Md* = 0.72, *SE* = 0.21, p = .024).

For session 4, a significant main effect was found, $F(3,45) = 7.09$, $p = .001$, $\eta_p^2 = .32$. Pairwise comparisons showed that the images in the musculoskeletal-pain category were significantly more pleasant than the facial expressions of pain (*Md* = 0.52, *SE* = 0.10, p = .001) and general-threat categories (*Md* = 0.48, *SE* = 0.12, p = .005).

For session 6, a significant main effect was found, $F(3,45) = 32.12$, $p <.001$, $\eta_p^2 = .68$. Pairwise comparisons revealed that images in the musculoskeletal-pain category were

For peer review only - http://bmjopen.bmj.com/site/about/guidelines.xhtml

significantly more pleasant than the images included in the facial expressions of pain (*Md* = 1.05, *SE* = 0.14, $p < .001$) and general-threat categories (*Md* = 1.27, *SE* = 0.13, $p < .001$). The images in the health-threat category were also significantly more pleasant than the images included in the facial expressions of pain (*Md* = 0.56, *SE* = 0.14, *p* = .01) and general-threat categories (*Md* = 0.77, *SE* = 0.13, $p < .001$).

For session 8, a significant main effect was found, $F(3,45) = 11.73$, $p < .001$, $\eta_p^2 = .44$. Pairwise comparisons revealed that images in the musculoskeletal-pain category were significantly more pleasant than those included in the facial expressions of pain (*Md* = 0.62, *SE* = 0.07, $p < .001$) and general-threat categories (*Md* = 0.57, *SE* = 0.14, *p* = .004). The images included in the health-threat category were also significantly more pleasant than those included in the facial expressions of pain (*Md* = 0.54, *SE* = 0.15, *p* = .019) and general-threat categories (*Md* = 0.49, *SE* = 0.14, *p* = .016).

For session 10, a significant main effect was found, $F(3,45) = 12.59$, $p < .001$, $\eta_p^2 = .46$. Pairwise comparisons revealed that images in the health-threat category were significantly less pleasant than those included in the musculoskeletal-pain (*Md* = 0.74, *SE* = 0.12, $p < .001$) and facial expressions of pain categories (*Md* = 0.44, *SE* = 0.12, *p* = .017).

For session 12, a significant main effect was found, $F(3,45) = 14.90$, $p < .001$, $\eta_p^2 = .50$. Pairwise comparisons revealed that images in the musculoskeletal-pain category were significantly more pleasant than images included in the facial expressions of pain (*Md* = 0.45, *SE* = 0.08, $p < .001$) and general-threat categories (*Md* = 0.88, *SE* = 0.16, $p < .001$). Images in the health-threat category were also significantly more pleasant than images included in the general health category (*Md* = 0.68, *SE* = 0.16, *p* = .004).

For session 14, a significant main effect was found, $F(3,45) = 13.30$, $p < .001$, $\eta_p^2 = .47$. Pairwise comparisons revealed that images in the musculoskeletal-pain category were significantly more pleasant than images included in the facial expressions of pain (*Md* = 0.71, *SE*

For peer review only - http://bmjopen.bmj.com/site/about/guidelines.xhtml

= 0.16, *p* = .003), general-threat (*Md* = 0.71, *SE* = 0.15, *p* = .001) and health-threat categories (*Md* = 0.82, *SE* = 0.12, *p* < .001).

For session 16, a significant main effect was found, $F(3,45) = 83.05$, $p <.001$, $\eta_p^2 = .85$. Pairwise comparisons revealed that images in the facial expressions of pain category were significantly less pleasant than musculoskeletal-pain (*Md* = 0.97, *SE* = 0.10, *p* < .001) and health-threat categories (*Md* = 0.99, *SE* = 0.12, *p* < .001). Images in the general-threat category were also significantly less pleasant than those included in the musculoskeletal-pain (*Md* = 1.34, *SE* = 0.11, *p* < .001), facial expressions of pain (*Md* = 0.37, *SE* = 0.09, *p* = .007) and health-threat categories (*Md* = 1.36, *SE* = 0.09, *p* < .001).

Differences in Pain Intensity Scores of Threatening Pain Categories across the eight Pictorial Visual Probe Sessions

A 2 (image category; musculoskeletal-pain, facial expressions of pain) x 8 (pictorial visual probe sessions; session 2, 4, 6, 8, 10, 12, 14, 16) ANOVA was conducted to compare the pain intensity scores of the threatening pain categories across the 8 pictorial visual probe task sessions. The results showed significant main effects of image category, $F(1,15) = 45.40$, $p < .001$, $\eta_p^2 = .75$, and session, $F(2,29) = 3.81$, $p = .04$, $\eta_p^2 = .20$, and a significant image category x session interaction, $F(3,44) = 7.44$, $p < .001$, $\eta_p^2 = .33$.

Post-hoc analyses within image categories. To explore the significant interaction, two one-way ANOVAs with 8 levels (Session; 2, 4, 6, 8, 10, 12, 14, 16) were run for each pain-related image category independently. These explored whether the pain intensity ratings of each pain-related image category differed across the sessions. For the musculoskeletal-pain category, a main effect of session was found, $F(2,25) = 4.70$, $p = .023$, $\eta p^2 = .24$. Pairwise comparisons revealed that the images included in session 14 had significantly less pain intensity scores than the images included in session 8 (*Md* = 1.06, *SE* = 0.24, *p* = .014), session 10 (*Md* = 0.95, *SE* = 0.19, *p* = .004) and session 12 (*Md* = 1.19, *SE* = 0.15, *p* < .001). Images in session 4 had significantly higher pain intensity scores than the images included in session 6 (*Md* = 0.82, *SE* =

For peer review only - http://bmjopen.bmj.com/site/about/guidelines.xhtml

0.21, p = .05). The images included in session 12 had also significantly higher pain intensity scores than the images included in session 16 (Md = 1.03, SE = 0.17, p = .001).

For the facial expression of pain category, a main effect of session was found, $F(3,39)$ = 4.65, p = .01, ηp^2 = .24. Pairwise comparisons revealed that the images included in session 10 had significantly less pain intensity scores that the images included in session 1 (Md = 1.07, SE = 0.25, p = .02) and session 6 (Md = 1.38, SE = 0.31, p = .013).

Differences in low-level features of pictorial categories across the eight visual probe sessions

A series of one-way ANOVAs were conducted independently to compare the scores of complexity, red, green and blue saturation, luminance and RMS contrast level of the threat related categories (i.e., musculoskeletal-pain, facial expression of pain, general-threat and health-threat) across the 8 pictorial visual probe task sessions (i.e., Session 2, 4, 6, 8, 10, 12, 14, 16).

For complexity, the analysis revealed a significant main effect of image category, $F(3, 224)$ = 5.15, p = .002, η_p^2 = .06. There was no significant main effect of session, $F(7, 224)$ = 1.22, p = .30, and no significant image category x session interaction, $F(21, 224)$ = 0.82, p =.70.

For red saturation, the analysis revealed a significant main effect of image category, $F(3, 224)$ = 5.05, p = .002, η_p^2 = .06. There was no significant main effect of session , $F(7,224)$ = 1.03, p = .41, and no significant image category x session interaction, $F(21,224)$ = 1.196, p =.26.

For green saturation, the analysis revealed a significant main effect of image category, $F(3, 224)$ = 4.08, p = .008, η_p^2 = .08. There was no significant main effect of session, $F(7, 224)$ = 1.65, p = .12, and no and no significant image category x session interaction, $F(21,224)$ = 1.41, p =.12.

For blue saturation, the analysis revealed a significant main effect of image category, $F(3, 224)$ = 5.288, p = .002, η_p^2 = 0.07, and a significant image category x session interaction, $F(21, 224)$ = 1.672, p = .04, η_p^2 = 0.14. There was no significant main effect of session, $F(7, 224)$ = 1.750, p = .10.

For peer review only

For luminance, the analysis revealed a significant main effect of image category, $F(3, 224) = 4.548$, $p = .004$, $\eta_p^2 = 0.06$. The analysis revealed no significant main effect of session, $F(7,224) = 1.504$, $p = .17$, and no significant image category x session interaction, $F(21, 224) = 1.361$, $p = .14$.

For RMS contrast level, the analysis revealed a significant main effect of image category, $F(3, 224) = 6.84$, $p < .001$, $\eta_p^2 = .08$, and a significant image category x session interaction, $F(21,224) = 1.98$, $p = .008$. There was no significant main effect of session, $F(7,224) = 1.68$, $p = .12$,

Post-hoc analyses within image categories. To explore the significant interaction for blue saturation, a series of one-way ANOVAS with 8 levels (Session; 2, 4, 6, 8, 10, 12, 14, 16) were run for each image category independently. These explored whether the blue saturation scores for each image category differed across the sessions. No significant main effect of session was found for the musculoskeletal-pain category, $F(7,56) = 0.82$, $p = .57$, or the health-threat category, $F(7,56) = 1.59$, $p = .16$.

For the facial expressions of pain category, a significant interaction for session number was found, $F(7,56) = 2.94$, $p = .01$, $\eta_p^2 = .27$. Pairwise comparisons did not reveal any significant differences between the factors.

For the general-threat category, a significant main effect of session number, $F(7,56) = 2.20$, $p = .05$, $\eta_p^2 = .22$. Pairwise comparisons did not reveal any significant differences between the factors.

To explore the significant interaction for RMS contrast level, a series of one-way ANOVAS with 8 levels (Session; 2, 4, 6, 8, 10, 12, 14, 16) were run for each image category independently. These explored whether the RMS contrast level scores for each image category differed across the sessions. No significant main effect of session was found for the musculoskeletal-pain category, $F(7,56) = 0.59$, $p = .76$, or the health-threat category, $F(7,56) = 0.16$, $p = .99$

For peer review only - http://bmjopen.bmj.com/site/about/guidelines.xhtml

For the facial expressions of pain category, a significant interaction for session number was found, $F(7,56) = 5.127$, $p < .001$, $\eta_p^2 = 0.39$. Pairwise comparisons revealed that the images in session 6 had significantly less RMS contrast than those included in session 4 (*Md* = 36.02, *SE* = 7.76, *p* = .001), session 10 (*Md* = 30.81, *SE* = 7.76, *p* = .006), session 14 (*Md* = 25.96, *SE* = 7.76, *p* = .04) and session 16 (*Md* = 37.09, *SE* = 776, *p*< .001).

For the general-threat category, a significant interaction for session number was found, $F(7,56) = 3.252$, $p = .006$, $\eta_p^2 = 0.29$. Pairwise comparisons did not reveal any significant interaction between the sessions.

Post-hoc analyses within sessions. To further explore the significant interaction for blue saturation, eight one-way ANOVAs with 4 levels (image category; musculoskeletal-pain, facial expressions of pain, general-threat and health-threat) were conducted for each session independently. These explored whether blue saturation scores between the five image categories differed within each session (i.e., session 2, 4, 6, 8, 10, 12, 14, 16).

No significant main effects were found for session 2, $F(3,28) = 1.49$, $p = .24$, session 4, $F(3,28) = 0.18$, $p = .91$, session 6, $F(3,28) = 1.37$, $p = .27$, session 8, $F(3,28) = 1.127$, $p = .36$, and session 14, $F(3,28) = 0.508$, $p = .68$.

For session 10, there was a significant main effect of image category, $F(3,28) = 5.56$, $p = .004$, $\eta_p^2 = .37$. Pairwise comparisons revealed that the images included in the facial expression of pain category had significantly higher blue saturation than the images included in the general-threat category (*Md* = 70.64, *SE* = 18.03, *p* = .003).

For session 12, there was a significant main effect of image category, $F(3,28) = 3.55$, $p = .027$, $\eta_p^2 = .28$. Pairwise comparisons revealed that the images included in the musculoskeletal-pain category had significantly higher blue saturation than the images included in the general-threat category (*Md* = 62.46, *SE* = 21.76, *p* = .046).

For session 16, there was a significant main effect of image category, $F(3,28) = 6.99$, $p = .001$, $\eta_p^2 = .43$. Pairwise comparisons revealed that the images included in the health-threat

For peer review only - http://bmjopen.bmj.com/site/about/guidelines.xhtml

categories had significantly higher blue saturation than the images included in the musculoskeletal-pain (*Md* = 74.57, *SE* = 20.09, *p* = .005) and general-threat categories (*Md* = 83.89, *SE* = 20.09, *p* = .002)

To further explore the significant interaction for RMS contrast level, eight one-way ANOVAs with 4 levels (image category; musculoskeletal-pain, facial expressions of pain, general-threat and health-threat) were conducted for each session independently. These explored whether the scores of RMS contrast level between the five image categories differed within each session (i.e., session 2, 4, 6, 8, 10, 12, 14, 16).

No significant main effects were found for session 2, $F(3,28) = 0.720$, $p = .55$, session 8, $F(3,28) = 1.56$, $p = .22$, session 12, $F(3,28) = 0.62$, $p = .61$, and session 14, $F(3,28) = 1.76$, $p = .18$.

For session 4, there was a significant main effect of image category, $F(3,28) = 5.69$, $p = .004$, $\eta_p^2 = .38$. Pairwise comparisons revealed that the images included in the facial expression of pain category had significantly higher RMS contrast than the images included in the health-threat category (*Md* = 33.44, *SE* = 8.27, *p* = .002).

For session 6, there was a significant main effect of image category, $F(3,28) = 4.00$, $p = .02$, $\eta_p^2 = .30$. Pairwise comparisons revealed that the images included in the musculoskeletal-pain category had significantly higher RMS contrast than those included in the facial expressions of pain category (*Md* = 17.69, *SE* = 5.95, *p* = .04).

For session 10, there was a significant main effect of image category, $F(3,28) = 6.02$, $p = .003$, $\eta_p^2 = .39$. Pairwise comparisons revealed that the images included in the facial expressions of pain category had significantly higher RMS contrast than those included in the general-threat (*Md* = 30.37, *SE* = 7.35, *p* = .002) and health-threat categories (*Md* = 21.42, *SE* = 7.35, *p* = .04).

For session 16, there was a significant main effect of image category, $F(3,28) = 3.18$, $p = .04$, $\eta_p^2 = .25$. Pairwise comparisons revealed that the images included in the facial expressions

For peer review only - http://bmjopen.bmj.com/site/about/guidelines.xhtml

of pain category had significantly higher RMS contrast level than those included in the health-threat category (*Md* = 29.32, *SE* = 10.24, *p* = .05).

For peer review only

References

1. Kučera H, Francis N. *Computational analysis of present-day American English*. Providence: Brown University Press, 1967.

2. AP Y. The Nature of Recollection and Familiarity: A Review of 30 Years of Research. Journal of Memory and Language 2002;**46**(3):441-517 doi: 10.1006/jmla.2002.2864.

3. Balota DA, Chumbley JI. Are lexical decisions a good measure of lexical access? The role of word frequency in the neglected decision stage. Journal of Experimental Psychology: Human perception and performance 1984;**10**(3):340-57.

4. Sharpe L, Ianiello, M., Dear, B. F, Nicholson Perry, K., Refshauge, K., Nicholas, M. K. Is there a potential role for attention bias modification in pain patients? Results of 2 randomised, controlled trials. Pain 2012;**153**(3):722-31.

5. Schoth D, Liossi C. Specificity and time-course of attentional bias in chronic headache: a visual-probe investigation. Clinical Journal of Pain 2013;**29**(7):583-90 doi: 10.1097/AJP.0b013e31826b4849.

6. Carleton R, Richter A, Asmundson G. Attention Modification in Persons with Fibromyalgia: A Double Blind, Randomized Clinical Trial. Cognitive Behaviour Therapy 2011;**40**(4):279-90.

7. Macleod C. Cognitive bias modification procedures in the management of mental disorders. Curr Opin Psychiatry 2012;**25**(2):114-20 doi: 10.1097/YCO.0b013e32834fda4a.

8. MacLeod C, Mathews A, Tata P. Attentional bias in emotional disorders. Journal of Abnormal Psychology 1986;**95**(1):15-20 doi: 10.1037/0021-843X.95.1.15.

9. Melzack R. The McGill Pain Questionnaire: major properties and scoring methods. Pain 1975;**1**(3):277-99.

10. Wilson MD. The MRC Psycholinguistic Database: Machine Readable Dictionary, Version 2. Behavioural Research Methods, Instruments and Computers 1988;**20**(1):6-11.

For peer review only - http://bmjopen.bmj.com/site/about/guidelines.xhtml

11. MRC Psycholinguistic Database: Machine Usable Dictionary. Version 2.00.: MRC Psycholinguistic Database, 1997.

12. Lane RD, Chua PM, Dolan RJ. Common effects of emotional valence, arousal and attention on neural activation during visual processing of pictures. Neuropsychologia 1999;**37**(9):989-97 doi: 10.1016/S0028-3932(99)00017-2.

13. Niedenthal PM, Kitayama S. *The heart's eye: Emotional influences in perception and attention*. London: Academic Press, 2013.

14. Bradley MM, Lang PJ. Measuring Emotion: The Self-Assessment Manikin and the Semantic Differential. J Behav Therap & Exp Psychiat 1994;**25**(1):49-59.

15. Liossi C, Schoth D. Attention toward interpersonal stimuli in individuals with and without chronic daily headache. Journal of Neurology and Neurosurgery 2016;**3**(3):1-7.

16. Schoth DE, Liossi C. Attentional bias toward pictorial representations of pain in individuals with chronic headache. Clin J Pain 2010;**26**(3):244-50 doi: 10.1097/AJP.0b013e3181bed0f9.

17. Craig KD. The facial expression of pain. Better than a thousand words? APS Journal 1992;**1**(3):153-62.

18. Prkachin KM, Solomon PE. The structure, reliability and validity of pain expression: evidence from patients with shoulder pain. Pain 2008;**139**(2):267-74 doi: 10.1016/j.pain.2008.04.010.

19. Schoth DE, Georgallis T, Liossi C. Attentional bias modification in people with chronic pain: a proof of concept study. Cognitive Behaviour Therapy 2013;**42**(3):233-43.

20. Bradley MM, Lang PJ. The International Affective Picture System (IAPS) in the study of emotion and attention. In: Allen CaJJB, ed. Handbook of Emotion Elicitation and Assessment: Oxford University Press, 2007.

21. Leeuw M, Goossens ME, van Breukelen GJ, et al. Measuring perceived harmfulness of physical activities in patients with chronic low back pain: the Photograph Series of Daily

For peer review only

Activities--short electronic version. J Pain 2007;**8**(11):840-9 doi: 10.1016/j.jpain.2007.05.013.

22. Tottenham N, Tanaka JW, Leon AC, et al. The NimStim set of facial expressions: judgments from untrained research participants. Psychiatry Res 2009;**168**(3):242-9 doi: 10.1016/j.psychres.2008.05.006.

23. Nummenmaa L, Hyönä J, Calvo MG, & Nummenmaa, L. Eye movement assessment of selective attentional capture by emotional pictures. Emotion 2006;**6**(2):257-68 doi: 10.1037/1528-3542.6.2.257.

24. Borji A, Sihite DN, Itti L. What stands out in a scene? A study of human explicit saliency judgment. Vision research 2013;**91**:62-77 doi: 10.1016/j.visres.2013.07.016.

25. Henderson JM. Human gaze control during real-world scene perception. Trends in Cognitive Sciences 2003;**7**(11):498-504 doi: 0.1016/j.tics.2003.09.006.

26. Torralba A, Oliva, A., Castelhano, M. S., & Henderson, J. M. Contextual guidance of eye movements and attention in real-world scenes: The role of global features in object search. Psychological Review 2006;**113**(4):766-86 doi: 10.1037/0033-295X.113.4.766.

27. Buodo G, Sarlo M, Palomba D. Attentional resources measured by reaction times highlight differences within pleasant and unpleasant, high arousing stimuli. Motivation and Emotion 2002;**26**(2):123-38 doi: 10.1023/A:1019886501965.

For peer review only

Supplementary Material 2 – Outcome Measures

A detailed description of each outcome measure used in this study is provided here. While the questionnaires will be administered online as part of this study, the psychometric properties reported are for paper and pencil versions of the questionnaires.

The BPI-SF[1] is a commonly used measure of pain intensity and pain interference. Participants indicate their worst, least, average and current pain on an 11-point numerical scale ranging from *no pain at all* to *pain as bad as you can imagine*. Additional questions include how much relief pain medication has provided, along with pain interference on general activity, mood, walking ability, normal work, relations with others, sleep, and enjoyment of life. A body diagram is also provided for participants to indicate where they feel pain. The psychometric properties of the BPI are well supported in patients with cancer pain and other pain populations[1 2], including internal consistency of pain intensity and interference items as assessed in patients with chronic nonmalignant pain (Cronbach's alpha .85 and .88 respectively).[3]

The HADS[4] consists of 14 items measuring the severity of anxiety and depression symptoms (seven items each) over the past week. Each item is measured on a four point scale, with possible scores ranging from 0 – 21 for each subscale. Higher scores indicate higher levels of anxiety and depression. A review of studies using the HADS (the majority of which were conducted with patients with cancer or other physical health conditions) has supported the internal consistency of anxiety (.68 - .93, mean .82) and depression (.67 - .90, mean .82) subscales.[5]

The STAI[6] is a 40-item measure of state and trait anxiety (20 items each). Each item is measuerd on a four point scale , with possible scores for both state and trait levels ranging between 20 and 80. Higher scores represent more intense or more frequent feelings of anxiety. A review of 45 articles reporting psychometric properties from clinical and non-clinical samples of state and trait subscales found high levels of internal consistency (.91 and .89 respectively) and test-retest reliability (.70 and .88 respectively).[7]

For peer review only - http://bmjopen.bmj.com/site/about/guidelines.xhtml

The FOP-III[8] is a 30 item measure of pain-related fear. On a 5-point scale, respondents are asked to rate how fearful they are of pain associated with specific situations and events (e.g., being in an automobile accident). Possible scores range between 30 and 150, with higher scores representing a more intense fear of pain. In addition to a total score, the measure also provides subscales of minor, severe, and medical pain-related fear. The FOP-III is commonly used in clinical and non-clinical populations, and possesses high internal consistency as indexed in a non-clinical sample (total score = .93; severe = .88; minor = .86, medical = .88).[9]

The MOS-SS[10] is a twelve-item questionnaire developed for patients with chronic illness, measuring six dimensions of sleep over the past four weeks; sleep initiation (time to fall asleep), quantity (hours per night sleeping), maintenance, respiratory problems, perceived adequacy, and somnolence. A sleep problems index which summarises information from nine items may also be scored. Responses are recoded on a 0 – 100 scale, with higher scores indicating more of the concept being measured (e.g., greater difficulties falling asleep). Research has supported the internal consistency of the MOS-SS scales to be acceptable (.70) or higher in samples of patients with neuropathic pain.[11 12]

The SOT[13] assesses participants' attitudes towards their online administered treatment, and will be used at the study endline. Participants state how strongly they agree/disagree with each of the following items: (a) the use of a computer to access treatment, (b) level of easiness of the online intervention, (c) duration of the potential effects of the treatment, and (d) potential recommendation of online interventions to others. Responses range from "*Agree very strongly*" to "*Disagree very strongly*". An additional question asks participants how helpful they have found the online intervention, with responses ranging from "*Very helpful*" to "*Not at all helpful*". Two open questions are also included that require participants to state the aspects of the intervention they most liked and disliked.

The EOT, developed by the researchers, will be presented at the end of every online session. The EOT includes three questions. The first asks participants to indicate where they

For peer review only - http://bmjopen.bmj.com/site/about/guidelines.xhtml

completed the online training session (e.g., home or office). The second is related to whether or not they have been distracted during the training and, if distracted, to indicate the source of distraction (e.g., telephone ringing). The third question asks participants to rate the level of their concentration during the online task on an 11-point Visual Analogue Scale (VAS) numbered from 0 (i.e., *no concentration at all*) to 10 (i.e. *very concentrated*).

For peer review only

References

1. Cleeland CS, Ryan KM. Pain assessment: global use of the Brief Pain Inventory. Ann Acad Med Singapore 1994;**23**(2):129-38.

2. Jensen MP. The validity and reliability of pain measures in adults with cancer. The Journal of Pain 2003;**4**(1):2-21 doi: 10.1054/jpai.2003.1.

3. Tan G, Jensen MP, Thornby JI, et al. Validation of the Brief Pain Inventory for chronic nonmalignant pain. The Journal of Pain 2004;**5**(2):133-37 doi: 10.1016/j.jpain.2003.12.005.

4. Zigmond AS, Snaith RP. The hospital anxiety and depression scale. Acta Psychiatr Scand 1983;**67**(6):361-70.

5. Bjelland I, Dahl AA, Tangen T, et al. The validity of the Hospital Anxiety and Depression Scale: An updated literature review. Journal of Psychosomatic Research 2002;**52**:69-77 doi: 10.1016/S0022-3999(01)00296-3.

6. Spielberger CD, Gorsuch RL, Lushene RE. *State Trait Anxiety Inventory*. Palo Alto, California: Consulting Psychologists Press, 1970.

7. Barnes LLB, Harp D, Jung WS. Reliability generalization of scores on the Spielberger State-Trait Anxiety Inventory. Educational and Psychological Measurement 2002;**62**(4):603 - 18 doi: 10.1177/0013164402062004005.

8. McNeil DW, Rainwater A. Development of the Fear of Pain Questionnaire-III. Journal of Behavioral Medicine 1998;**21**(4):389-410 doi: 10.1023/A:1018782831217.

9. Roelofs J, Peters ML, Deutz J, et al. The Fear of Pain Questionnaire (FPQ): further psychometric examination in a non-clinical sample. PAIN 2005;**116**(3):339-46 doi: 10.1016/j.pain.2005.05.003.

10. Hays RD, Stewart AL. Sleep Measures. Measuring functioning and well-being. In: Steweart AL, JEJ W, eds. Measuring function and well-being; the medical outcome study approach. Duke: Duke University Press, 1992:235-59.

For peer review only

11. Hays RD, Martin SA, Sesti AM, et al. Psychometric properties of the medical outcomes study sleep measure. Sleep Medicine 2005;**6**(1):41-44 doi: 10.1016/j.sleep.2004.07.006.

12. Viala-Danten M, Martin S, Guillemin I, et al. Evaluation of the reliability and validity of the Medical Outcomes Study sleep scale in patients with painful diabetic peripheral neuropathy during an international clinical trial. Health and Quality of Life Outcomes 2008;**6**(113) doi: 10.1186/1477-7525-6-113.

13. Richards D, Timulak L. Satisfaction with therapist-delivered vs. self-administered online cognitive behavioural treatments for depression symptoms in college students. British Journal of Guidance & Counselling 2013;**41**(2):193-207 doi: 10.1080/03069885.2012.726347.

For peer review only

Supplementary Material 3 – Think Aloud Study

A qualitative think aloud study[1 2] was conducted following the development of the initial version of the ABMT intervention to address the question: *What are the thoughts and first impressions of individuals with chronic musculoskeletal pain towards an online ABMT programme?* Ethical approval was obtained from the University of Southampton Research Ethics Committee (ERGO ID: 26486). Thirteen participants (mean age = 37.54, *SD* = 16.31, range 21 – 60 years) with chronic musculoskeletal pain were recruited from the South of England from a chronic pain support group.

Each participant met with the researcher (FH) on a single occasion to take part in a think aloud interview followed by a short semi-structured interview. Participants navigated their way through the iABMT Lifeguide website and completed the first assessment visual-probe task, with the researcher seated next to them. The researcher used an interview topic guide featuring standardized instructions that were read at the beginning of the study, as well as a list of pre-determined neutral prompts used when the participant stopped talking or showed a reaction to the material through their facial expressions. On occasions where the participant deviated from the topic, the researcher attempted to steer the participant's focus back to the task at hand. The prompts used were similar to those in former research[3], such as '*what are you thinking right now?*' and '*tell me why you think that*'.

Following the think-aloud interview, a short semi-structured interview took place for the researcher to ask questions about any specific aspects of the intervention that had not yet been discussed. Questions were also asked regarding thoughts about the intervention as a whole. This method of triangulation is commonly used to complement a think-aloud interview because it can be difficult to interpret spoken thoughts alone.[4 5] Following transcription of the interviews, thematic analysis using inductive encoding was used to identify themes that clearly represent the raw data, following guidelines set out by Braun and

For peer review only - http://bmjopen.bmj.com/site/about/guidelines.xhtml

Clarke.[6] Three themes and 13 subthemes were identified, which are shown in a thematic map in Figure S1 and defined in Table S1 below. While it is beyond the scope of this article to present the results of this thematic analysis in full, the main points resulting in amendments to the iABMT intervention are presented here.

Regarding the iABMT website, participants liked the continuity of design between webpages, although pointed out inconsistencies in the formatting of text, which have been rectified. On some pages the volume of text was considered to be too great, and therefore where possible text has been reduced, language simplified, better use of spacing made, and longer paragraphs divided into multiple shorter paragraphs. While some participants were positive about the inclusion of images, others felt additional visual stimuli could be added throughout. It was therefore decided to only include images where these would help emphasize the points being made. Regarding the visual-probe task sessions, one important practicality raised was ability of participants to stay seated for a sufficient amount of time in order to complete the task in a single session. An additional inclusion criteria has therefore been added to the study, being that participants must be able to sit at a personal computer for forty minutes. It was also mentioned that participants needed sufficient computer skills to take part in the study. The inclusion criteria were therefore amended to state that participants need both access to and familiarity using a Window-based computer.

For peer review only - http://bmjopen.bmj.com/site/about/guidelines.xhtml

References

Theme/Sub-theme	Definition
A. AMBT website	Comments relating to the ABMT website including the post-intervention questionnaires
A1. Accessibility and usability	Comments relating to the ease with which participants were able

1. Yardley L, Ainsworth B, Arden-Close E, et al. The person-based approach to enhancing the acceptability and feasibility of interventions. Pilot and Feasibility Studies 2015;**1**(1):37.
2. Van den Haak MJ, De Jong MD, Schellens PJ. Evaluation of an informational web site: three variants of the think-aloud method compared. Technical Communication 2007;**54**(1):58-71.
3. Morrison L, Moss-Morris R, Michie S, et al. Optimizing engagement with internet-based health behaviour change interventions: Comparison of self-assessment with and without tailored feedback using a mixed methods approach. British Journal of Health Psychology 2014;**19**(4):839-55 doi: 10.1111/bjhp.12083.
4. Charters E. The use of think-aloud methods in qualitative research an introduction to think-aloud methods. Brock Education Journal 2003;**12**(2).
5. Anthierens S, Tonkin-Crine S, Douglas E, et al. General practitioners' views on the acceptability and applicability of a web-based intervention to reduce antibiotic prescribing for acute cough in multiple European countries: a qualitative study prior to a randomised trial. BMC Family Practice 2012;**13**(101) doi: 10.1186/1471-2296-13-101.
6. Braun V, Clarke V. Using thematic analysis in psychology. Qualitative Research in Psychology 2006;**3**(2):77-101 doi: 10.1191/1478088706qp063oa.

For peer review only

For peer review only - http://bmjopen.bmj.com/site/about/guidelines.xhtml

	to use the website and the extent to which it was deemed to be accessible
A2. Visual design	Comments relating to the visual design and appearance of the website, including colour, orientation of elements and text
A3. Questionnaires	Comments regarding any aspect of the post-intervention questionnaires, including their purpose and presentation
A4. Specific menu pages	Comments pertaining to the six main menu pages, which are titled (i) How does ABMT work, (ii) Will ABMT cure my pain, (iii) The team behind ABMT, (iv) Evidence for ABMT, (v) Why try ABMT, and (iv) What is it like to use ABMT?
B. Visual-probe task	Comments relating to any and all aspects of the visual-probe tasks
B1. Experiences of testing the intervention	Comments provided prior to and during testing of the visual-probe task
B2. Attitudes towards task	Positive and negative attitudes expressed by participants regarding the visual-probe task
B3. Practicalities	Comments pertaining to the feasibility and practicalities of completing the visual-probe task, including commitment needed
C. Influencing factors	All personal and situational factors that may affect the participants' experiences of testing the intervention
C1. Interviewing situation	Comments relating to the style of qualitative research adopted for the think aloud study
C2. Personal barriers	Comments relating to personal factors that hindered participants' completion of the visual-probe task
C3. Personal facilitators	Comments relating to personal factors that increased participants' ability to complete the visual-probe task and improved their attitude regarding it

Table S1. Definitions of themes and subthemes identified in the think aloud study

For peer review only

STANDARD PROTOCOL ITEMS: RECOMMENDATIONS FOR INTERVENTIONAL TRIALS

SPIRIT 2013 Checklist: Recommended items to address in a clinical trial protocol and related documents*

Section/item	Item No	Description	Addressed on page number
**Administrative information**			
Title	1	Descriptive title identifying the study design, population, interventions, and, if applicable, trial acronym	1
Trial registration	2a	Trial identifier and registry name. If not yet registered, name of intended registry	1
	2b	All items from the World Health Organization Trial Registration Data Set	Registered with clinicaltrials.gov
Protocol version	3	Date and version identifier	1
Funding	4	Sources and types of financial, material, and other support	22
Roles and responsibilities	5a	Names, affiliations, and roles of protocol contributors	1, 22
	5b	Name and contact information for the trial sponsor	22
	5c	Role of study sponsor and funders, if any, in study design; collection, management, analysis, and interpretation of data; writing of the report; and the decision to submit the report for publication, including whether they will have ultimate authority over any of these activities	22
	5d	Composition, roles, and responsibilities of the coordinating centre, steering committee, endpoint adjudication committee, data management team, and other individuals or groups overseeing the trial, if applicable (see Item 21a for data monitoring committee)	____________

For peer review only

**Introduction**			
Background and rationale	6a	Description of research question and justification for undertaking the trial, including summary of relevant studies (published and unpublished) examining benefits and harms for each intervention	4-5
	6b	Explanation for choice of comparators	4
Objectives	7	Specific objectives or hypotheses	5
Trial design	8	Description of trial design including type of trial (eg, parallel group, crossover, factorial, single group), allocation ratio, and framework (eg, superiority, equivalence, noninferiority, exploratory)	6-7
**Methods: Participants, interventions, and outcomes**			
Study setting	9	Description of study settings (eg, community clinic, academic hospital) and list of countries where data will be collected. Reference to where list of study sites can be obtained	6-7
Eligibility criteria	10	Inclusion and exclusion criteria for participants. If applicable, eligibility criteria for study centres and individuals who will perform the interventions (eg, surgeons, psychotherapists)	5
Interventions	11a	Interventions for each group with sufficient detail to allow replication, including how and when they will be administered	7-11
	11b	Criteria for discontinuing or modifying allocated interventions for a given trial participant (eg, drug dose change in response to harms, participant request, or improving/worsening disease)	14
	11c	Strategies to improve adherence to intervention protocols, and any procedures for monitoring adherence (eg, drug tablet return, laboratory tests)	7
	11d	Relevant concomitant care and interventions that are permitted or prohibited during the trial	5
Outcomes	12	Primary, secondary, and other outcomes, including the specific measurement variable (eg, systolic blood pressure), analysis metric (eg, change from baseline, final value, time to event), method of aggregation (eg, median, proportion), and time point for each outcome. Explanation of the clinical relevance of chosen efficacy and harm outcomes is strongly recommended	5, 11-14
Participant timeline	13	Time schedule of enrolment, interventions (including any run-ins and washouts), assessments, and visits for participants. A schematic diagram is highly recommended (see Figure)	Table 1

For peer review only - http://bmjopen.bmj.com/site/about/guidelines.xhtml

Sample size	14	Estimated number of participants needed to achieve study objectives and how it was determined, including clinical and statistical assumptions supporting any sample size calculations	6-7
Recruitment	15	Strategies for achieving adequate participant enrolment to reach target sample size	5

Methods: Assignment of interventions (for controlled trials)

Allocation:			
Sequence generation	16a	Method of generating the allocation sequence (eg, computer-generated random numbers), and list of any factors for stratification. To reduce predictability of a random sequence, details of any planned restriction (eg, blocking) should be provided in a separate document that is unavailable to those who enrol participants or assign interventions	7
Allocation concealment mechanism	16b	Mechanism of implementing the allocation sequence (eg, central telephone; sequentially numbered, opaque, sealed envelopes), describing any steps to conceal the sequence until interventions are assigned	7
Implementation	16c	Who will generate the allocation sequence, who will enrol participants, and who will assign participants to interventions	7
Blinding (masking)	17a	Who will be blinded after assignment to interventions (eg, trial participants, care providers, outcome assessors, data analysts), and how	7
	17b	If blinded, circumstances under which unblinding is permissible, and procedure for revealing a participant's allocated intervention during the trial	7

Methods: Data collection, management, and analysis

Data collection methods	18a	Plans for assessment and collection of outcome, baseline, and other trial data, including any related processes to promote data quality (eg, duplicate measurements, training of assessors) and a description of study instruments (eg, questionnaires, laboratory tests) along with their reliability and validity, if known. Reference to where data collection forms can be found, if not in the protocol	11, Supplementary material 2
	18b	Plans to promote participant retention and complete follow-up, including list of any outcome data to be collected for participants who discontinue or deviate from intervention protocols	8-9

Data management	19	Plans for data entry, coding, security, and storage, including any related processes to promote data quality (eg, double data entry; range checks for data values). Reference to where details of data management procedures can be found, if not in the protocol	14
Statistical methods	20a	Statistical methods for analysing primary and secondary outcomes. Reference to where other details of the statistical analysis plan can be found, if not in the protocol	11-13
	20b	Methods for any additional analyses (eg, subgroup and adjusted analyses)	N/A
	20c	Definition of analysis population relating to protocol non-adherence (eg, as randomised analysis), and any statistical methods to handle missing data (eg, multiple imputation)	13
**Methods: Monitoring**			
Data monitoring	21a	Composition of data monitoring committee (DMC); summary of its role and reporting structure; statement of whether it is independent from the sponsor and competing interests; and reference to where further details about its charter can be found, if not in the protocol. Alternatively, an explanation of why a DMC is not needed	______________
	21b	Description of any interim analyses and stopping guidelines, including who will have access to these interim results and make the final decision to terminate the trial	______________
Harms	22	Plans for collecting, assessing, reporting, and managing solicited and spontaneously reported adverse events and other unintended effects of trial interventions or trial conduct	14
Auditing	23	Frequency and procedures for auditing trial conduct, if any, and whether the process will be independent from investigators and the sponsor	______________
**Ethics and dissemination**			
Research ethics approval	24	Plans for seeking research ethics committee/institutional review board (REC/IRB) approval	14
Protocol amendments	25	Plans for communicating important protocol modifications (eg, changes to eligibility criteria, outcomes, analyses) to relevant parties (eg, investigators, REC/IRBs, trial participants, trial registries, journals, regulators)	14

Consent or assent	26a	Who will obtain informed consent or assent from potential trial participants or authorised surrogates, and how (see Item 32)	7-8, 14
	26b	Additional consent provisions for collection and use of participant data and biological specimens in ancillary studies, if applicable	____________
Confidentiality	27	How personal information about potential and enrolled participants will be collected, shared, and maintained in order to protect confidentiality before, during, and after the trial	14
Declaration of interests	28	Financial and other competing interests for principal investigators for the overall trial and each study site	22
Access to data	29	Statement of who will have access to the final trial dataset, and disclosure of contractual agreements that limit such access for investigators	31
Ancillary and post-trial care	30	Provisions, if any, for ancillary and post-trial care, and for compensation to those who suffer harm from trial participation	_____________
Dissemination policy	31a	Plans for investigators and sponsor to communicate trial results to participants, healthcare professionals, the public, and other relevant groups (eg, via publication, reporting in results databases, or other data sharing arrangements), including any publication restrictions	14
	31b	Authorship eligibility guidelines and any intended use of professional writers	22
	31c	Plans, if any, for granting public access to the full protocol, participant-level dataset, and statistical code	14
**Appendices**			
Informed consent materials	32	Model consent form and other related documentation given to participants and authorised surrogates	Supplementary materials
Biological specimens	33	Plans for collection, laboratory evaluation, and storage of biological specimens for genetic or molecular analysis in the current trial and for future use in ancillary studies, if applicable	N/A

*It is strongly recommended that this checklist be read in conjunction with the SPIRIT 2013 Explanation & Elaboration for important clarification on the items. Amendments to the protocol should be tracked and dated. The SPIRIT checklist is copyrighted by the SPIRIT Group under the Creative Commons "Attribution-NonCommercial-NoDerivs 3.0 Unported" license.

For peer review only

BMJ Open

Internet-delivered attentional bias modification training (iABMT) for the management of chronic musculoskeletal pain: a protocol for a randomised controlled trial

Journal:	*BMJ Open*
Manuscript ID	bmjopen-2019-030607.R1
Article Type:	Protocol
Date Submitted by the Author:	01-Aug-2019
Complete List of Authors:	Liossi, Christina; University of Southampton Faculty of Engineering and the Environment, Psychology;<br>Georgallis, Tsampikos; University of Southampton Faculty of Engineering and the Environment, School of Psychology<br>Zhang, Jin; University of Southampton Faculty of Engineering and the Environment, School of Psychology<br>Hamilton, Fiona; University of Southampton Faculty of Engineering and the Environment, School of Psychology<br>White, Paul; University of the West of England, Engineering, Design and Mathematics<br>Schoth, Daniel; University of Southampton Faculty of Engineering and the Environment, School of Psychology
**Primary Subject Heading**:	Neurology
Secondary Subject Heading:	Paediatrics, Qualitative research
Keywords:	Chronic pain, Musculoskeletal pain, Visual-probe task, Attention bias modification, Internet-delivered therapy, Randomised-controlled trial

SCHOLARONE™
Manuscripts

I, the Submitting Author has the right to grant and does grant on behalf of all authors of the Work (as defined in the below author licence), an exclusive licence and/or a non-exclusive licence for contributions from authors who are: i) UK Crown employees; ii) where BMJ has agreed a CC-BY licence shall apply, and/or iii) in accordance with the terms applicable for US Federal Government officers or employees acting as part of their official duties; on a worldwide, perpetual, irrevocable, royalty-free basis to BMJ Publishing Group Ltd ("BMJ") its licensees and where the relevant Journal is co-owned by BMJ to the co-owners of the Journal, to publish the Work in this journal and any other BMJ products and to exploit all rights, as set out in our licence.

The Submitting Author accepts and understands that any supply made under these terms is made by BMJ to the Submitting Author unless you are acting as an employee on behalf of your employer or a postgraduate student of an affiliated institution which is paying any applicable article publishing charge ("APC") for Open Access articles. Where the Submitting Author wishes to make the Work available on an Open Access basis (and intends to pay the relevant APC), the terms of reuse of such Open Access shall be governed by a Creative Commons licence – details of these licences and which Creative Commons licence will apply to this Work are set out in our licence referred to above.

Other than as permitted in any relevant BMJ Author's Self Archiving Policies, I confirm this Work has not been accepted for publication elsewhere, is not being considered for publication elsewhere and does not duplicate material already published. I confirm all authors consent to publication of this Work and authorise the granting of this licence.

For peer review only

Internet-delivered attentional bias modification training (ABMT) for the management of chronic musculoskeletal pain: a protocol for a randomised controlled trial

Christina Liossi[1*], Tsampikos Georgallis[1], Jin Zhang[1], Fiona Hamilton[1], Paul White[2], and Daniel E. Schoth[1,]

[1]Pain Research Laboratory, School of Psychology, University of Southampton, Highfield, Southampton, UK;

[2] Engineering, Design and Mathematics, University of the West of England, Bristol, UK

Protocol version 3.0; 25/07/19

Corresponding author: Prof. Christina Liossi, Pain Research Laboratory, Department of Psychology, University of Southampton, Highfield, Southampton, SO17 1BJ, UK. E-mail: C.Liossi@soton.ac.uk, Tel: 0044 (0)238059 4645.

Trial registration: This Study Protocol was registered in ClinicalTrials.gov (NCT02232100) on 1 September 2014

Word count: 4117

For peer review only

ATTENTIONAL BIAS MODIFICATION PROTOCOL

Abstract

Introduction: Chronic musculoskeletal pain is a complex medical condition that can significantly impact quality of life. Patients with chronic pain demonstrate attentional biases towards pain-related information, and the therapeutic benefits of modifying attentional biases by implicitly training attention away from pain-related information towards neutral information have been supported in the small number of published studies. Limited research however has explored the efficacy of modifying pain-related biases via the internet. This protocol describes a randomised, double-blind internet-delivered attentional bias modification intervention, aimed to evaluate the efficacy of the intervention on improving pain intensity and pain interference. Secondary outcomes are state and trait anxiety, depression, pain-related fear, and sleep impairment. This study will also explore the effects of training intensity on these outcomes, along with participants' perceptions about the online therapy. **Methods and analysis**: The study is a double-blind randomised controlled trial with four arms exploring the efficacy of online attentional bias modification training versus placebo training theorised to offer no specific therapeutic benefit. Participants with chronic musculoskeletal pain will be randomised to one of four groups: (i) 10-session attentional modification group; (ii) 10-session placebo training group; (iii) 18-session attentional modification group; or (iv) 18-session placebo training group. In the attentional modification groups, the visual-probe task will be used to implicitly train attention away from threatening information towards neutral information. Following the intervention, participants will complete a short interview exploring their perceptions about the online training. In addition, data will be analysed and reported separately for participants aged 16 – 24 and 25 – 60. **Ethics and dissemination**: This study has been approved by the University of Southampton Research Ethics Committee. Results will be published in peer-reviewed journals, academic conferences, and in lay reports for pain charities and patient support groups. **Registration**: The study protocol has been registered with ClinicalTrial.gov (NCT02232100).

For peer review only

Strengths and limitations of this study

- This protocol describes an internet-delivered intervention to explore the efficacy of attentional bias modification training in patients with chronic musculoskeletal pain.
- Recruitment of a diverse sample in terms of age, socioeconomic status, and geographical region.
- Results will provide important information regarding dose effects, age effects and participants' experiences and attitudes using the online intervention.
- Limited control over the conditions under which training is completed.
- Impossibility of independently verifying participant diagnosis.

Keywords: chronic pain; musculoskeletal pain; visual-probe task; attention bias modification; internet-delivered therapy; randomised-controlled trial.

For peer review only

Introduction

Chronic musculoskeletal pain (CMSK) is pain arising from bones, muscles, ligaments, tendons and/or joints lasting for more than three months.[1] Prevalence estimates of CMSK vary considerably, which for adults is estimated to affect between 11.4 and 24% of the population[2] and for children and adolescents ranges between 4 and 40% of the population.[3] Negative effects on quality of life are commonly reported in adult[4-7] and paediatric[8-11] CMSK populations, and theory driven research[12-14] has shown that patients with chronic pain exhibit attentional biases (i.e., a selective attention) for salient pain-related words (e.g.,[15-18]) and images (e.g.,[19 20]) relative to neutral information. Three meta-analyses have supported the presence of attentional biases in chronic pain patients[21-23], although evidence for the time course of such biases is mixed. Specifically, two meta-analyses reported larger effect sizes when stimuli are presented for longer than 1000 ms,[24 25] whereas a recent analysis found evidence of bias for presentation times of 500 – 1000ms but not when stimuli are presented for longer than 1000 ms.[23] Despite this inconsistency attentional biases are commonly shown in chronic pain, and preliminary research has explored the effects of re-directing attention may have on pain and pain-related distress using attentional bias modification (ABM) techniques. ABM is a computer-based intervention which implicitly trains attention away from threat-related cues toward neutral information, and is frequently achieved through a modified version of the visual-probe task[26] (Figure 1).

Two small randomised controlled trials (RCTs)[27 28] and one pilot study[29] have used the modified visual-probe task to assess ABM in adults with chronic pain. Carleton and colleagues[30] found participants receiving ABM showed significant reductions pre- to post ABM in anxiety sensitivity and fear of pain, and marginally significant reduction in current pain severity. No significant reductions were found in illness/injury sensitivity or pain anxiety. Significantly more patients receiving ABM reported clinically significant change in current pain compared with those in the placebo group (44% vs. 17%, respectively). Attentional bias scores were not recorded, and therefore the researchers could not comment on mechanisms of action. Sharpe and

For peer review only - http://bmjopen.bmj.com/site/about/guidelines.xhtml

colleagues[31] found participants receiving ABM, compared to those in a placebo group, showed significant improvements in pain-related disability after the ABM. No significant improvements were found for pain, fear of pain, fear of re-injury, depression, anxiety, stress, anxiety sensitivity or pain self-efficacy. At 6-month follow-up (and after all participants had undergone 8 sessions of CBT) the ABM group, compared to the placebo group, showed significant improvements in disability and anxiety sensitivity, and marginally significant improvements in fear of re-injury. No other significant effects were found, and the mechanism of action could not be established as ABM was not found to change patterns of attentional bias across time Schoth and colleagues[29] reported statistically and clinically significant change pre- to post-ABM in pain intensity, pain interference, anxiety, and depression. Assessed via the McGill Pain Questionnaire[32], significant changes pre- to post-ABM were shown for total pain, affective pain and miscellaneous pain, but not sensory-pain or evaluative pain. There was also no evidence that attentional biases changed across time.

One small RCT in adolescents with chronic pain randomised participants to either ABM, placebo training or waiting-list groups. No significant differences were found between the three groups in terms of pain, disability pain catastrophising, anxiety, depression, or objective measures of physical functioning (sit-to-stand task and cardio wall tasks), and no evidence was found that ABM changed attentional bias or attentional control compared to placebo and waiting-list conditions.[33] Overall, while some studies have provided support for the benefits of ABM in adults, the evidence in regards to specific outcome variables is mixed and the mechanisms of action have yet to be established. Given the methodological shortcomings and small sample sizes of previous studies however, further research is needed to determine the therapeutic benefits of ABM for patients with chronic pain, along with the optimal form ABM should take and the impact of age on outcomes and intervention engagement.

This article presents a study protocol for a randomised, double-blind internet-delivered ABM intervention. The results of a pilot think aloud study informing the design of the

For peer review only - http://bmjopen.bmj.com/site/about/guidelines.xhtml

intervention are also presented. The aim of this RCT is to assess the efficacy of internet-delivered ABM in improving pain and pain-related distress in people aged 16 –60 (additionally considered separately in two age brackets [i.e., 16-24 and 25-60]) with CMSK. A diverse sample in terms of age, socioeconomic status, and geographical region will be recruited, thus increasing the ecological validity of results to the CMSK population. The primary outcome measures are pain intensity and pain interference. Secondary outcome measures are state and trait anxiety, depression, pain-related fear and sleep impairment, and engagement to and satisfaction with the online visual-probe training task. Dose effects will be explored via the inclusion of 10 versus 18 session conditions. It is hypothesised that participants in the intervention group, relative to those in the placebo group, will report significant reductions in pain intensity, pain interference, anxiety, depression, sleep impairment and pain-related fear at study endline. It is also hypothesised that dose will significantly moderate these findings, with greater effects in 18 compared to 10 sessions.

Methods and Analysis

Participants

Participants will be recruited via press announcements, social media, and patient organizations throughout the UK. Inclusion criteria will be: (a) aged between 16 to 60 years old, (b) experiencing any type of CMSK (i.e., any condition that involves pain lasting for more than three months and arises from bones, muscles and/or joints), (c) able to sit at a personal computer for forty minutes, (d) normal or corrected to normal vision, (e) access to the internet at least twice a week, (f) access to, and familiarity using, a Windows-based computer, (g) successful completion of primary school, and (h) living in the United Kingdom. Exclusion criteria will be (1) experiencing malignant CMSK (i.e., pain caused due to a tumour), (2) a diagnosis of other forms of comorbid chronic pain (e.g., chronic headache), and (3) a diagnosis of any psychiatric disorder, either currently or within the last 5 years. There are no restrictions placed on

For peer review only

concomitant care and participants are not required to make any changes to current treatments they may be receiving.

Patient and Public Involvement

Participant burden of the intervention and research measures were assessed using individual interviews and informal feedback from patients participating in two pilot studies.[29 34] Patients will not be involved in recruitment of participants or conduct of the study. Results of this study will be disseminated to participants through presentation at client and community forums.

Study Design and Setting

The study is a double-blind randomised controlled trial (RCT) with four arms exploring the efficacy of online ABM training versus a placebo training condition theorised to offer no specific therapeutic benefits. The choice of comparator was selected as placebo training closely mimics attentional bias modification training, and is frequently used as a comparator in ABM studies (e.g.,[27 28 35 36]). The study will take place in a location of the participants choice (which we anticipate will typically be their home), which is accessed via personal computer connected to the internet.

The study design has a 2 x 2 x 3 experimental structure, with multiple non-commensurate dependent variables measuring pain intensity and interference, emotional functioning and pain-related disability. ABM intervention is a two-level factor (10AMG and 18AMG), placebo control is a two-level factor (10PCG and 18PTG) with measures recorded at three points in time (baseline, endline, and 6-month follow-up). Measures in the 18 session groups (18AMG and 18 PTG) will also be taken after session 10.

Randomisation and Sample Size

Block randomisation of size four, performed via a randomisation command in the Lifeguide platform[37], will be used to sequentially randomise consecutive consenting participants to one of four groups: (i) 10-session attentional modification group (10AMG); (ii) 10-session

For peer review only - http://bmjopen.bmj.com/site/about/guidelines.xhtml

placebo training group (10PTG); (iii) 18-session attentional modification group (18AMG); or (iv) 18-session placebo training group (18PTG). This blocked randomisation will be performed separately for those aged 16-24 and those aged 25-60. An allocation concealment mechanism will be used to ensure neither participants nor researchers know the study group to which the next participant will be assigned. This will be automatically performed within the Lifeguide platform (see below), with participants provided access to appropriate AMG or PTG sessions. Participants randomised to either 10-session condition will receive ten 35-minute online sessions across a four-week period, while those randomised to either 18-session condition will receive eighteen 35-minute online sessions across an eight-week period. All online sessions will be administered twice a week on a separate set of days (Monday-Thursday, Tuesday-Friday). Participants randomised to either AMG condition will receive attentional training via the modified training version of the visual probe-task, while those randomised to either PTG condition will receive placebo training via the standard version of the visual-probe task. To maintain the overall quality and legitimacy of the intervention, code breaks (i.e., unblinding) will only occur in exceptional circumstances. Adherence will be monitored electronically via Lifeguide.

This study is powered primarily to establish treatment efficacy for the primary outcome variables of pain intensity and pain interference by considering baseline and endline measures between 10AMG and 10PTG. Randomisation should ensure baseline comparability on measures between these two groups, and a difference in beneficial outcomes between these groups will be modelled using a linear mixed model. For an assumed correlation of 0.7 between baseline and endline a total of $n = 20$ per group would be needed to detect medium sized intervention differences with at least 80% power (alpha = 0.05, two-sided). Under the same conditions a sample size of $n = 40$ per group would have in excess of 80% power to effects (Cohen's $d = 0.25$). A 20% oversampling will be undertaken to mitigate a loss of power from loss to follow-up. Our former lab-based proof-of-concept ABM intervention found reductions in pain intensity and pain interference reduction pre- to post-ABM were associated with large effect sizes[29],

For peer review only

although differences in methodology including delivery (online vs lab-based) and number of sessions warrants a more conservative estimate.

Procedure

Interested individuals will contact the researchers on the details provided in study advertisements and will be assessed against the inclusion and exclusion criteria via telephone interview. If eligible, they will receive a copy of the participant information sheet (PIS) detailing the requirements and procedure of the study, along with a link to the study website which provides further details on ABM and the research team. If the individual consents to take part they will be required to create an online ABM account. During registration, participants will select their preferred days for training, which will be either (i) Monday/Thursday or (ii) Tuesday/Friday.

Session 1 is the baseline session, and includes the first assessment visual-probe task assessing attentional biases prior to beginning the intervention, along with the questionnaire battery assessing the primary and secondary outcome measures. Randomisation will take place following session 1, where participants will be informed of the length of their training (i.e., four or eight weeks). Participants randomised to either of the ten session conditions will complete four pictorial and four linguistic training sessions, while those randomised to either of the 18 session conditions will complete eight pictorial and eight linguistic training sessions (baseline and endline assessment sessions comprise the remainder of the 10/18 sessions). Participants' attentional biases will be re-assessed at endline (i.e., during the final session 10/18) using the same assessment version of the visual probe task (i.e., featuring the same stimuli) used at baseline and the same questionnaire battery. Participants randomised to either of the 18 session conditions will also have their attentional biases re-assessed in session 10 after their training session.

Participants' satisfaction with the online training will also be assessed. Six months after the last session, participants will be invited to again complete the online assessment visual-probe task (same as baseline and final session 10/18) and questionnaire battery. Within two weeks of

For peer review only - http://bmjopen.bmj.com/site/about/guidelines.xhtml

the completion of the six-month follow-up session, participants will be invited to take part in a brief telephone semi-structured interview including 15 questions spread across five thematic sections: (a) motivation and expectations about the study, (b) experience of the online study – person, environmental and lifestyle parameters, (c) experience of the visual-probe task, (d) understanding of the process of change, and (e) the visual-probe task as a potential therapeutic tool in pain management. The study flow is presented in Table 1.

Table 1. Schedule of enrolment, intervention, and assessments.

	STUDY PERIOD					
	Enrolment	Post-allocation				
TIMEPOINT**	$-t_1$	t_1 Baseline	t_2 Training	t_3 Evaluation	t_4 Follow-up	t_5 Study exit
**ENROLMENT:**						
**Eligibility screen**	X					
**Informed consent**	X					
**Allocation**		X				
**INTERVENTIONS:**						
Attentional bias modification training			X			
**ASSESSMENTS:**						
Demographic questionnaire		X				
EOT		X	X	X	X	
BPI-SF		X		X	X	
HADS		X		X	X	
STAI		X		X	X	
MOS-SS		X		X	X	
FOP-III		X		X	X	
SOT				X		
Interview						X

T1 = Baseline phase including standard assessment visual-probe task and questionnaires. Also includes randomization to groups.
T2 = Training phase including training visual-probe tasks.
T3 = Evaluation phase including standard assessment visual-probe task and questionnaires.

For peer review only - http://bmjopen.bmj.com/site/about/guidelines.xhtml

T4 = Six-month follow-up including standard assessment visual-probe task and questionnaires.
T5 = Telephone interview within two weeks of T4.

Note. EOT, Engagement with the Online Training questionnaire; BPI-SF, Brief Pain Inventory-Short Form; HADS, Hospital Anxiety and Depression Scale; STAI, State-Trait Anxiety Inventory; MOS-SS, Medical Outcome Study –Sleep Scale, FOP-III, Fear of pain questionnaire III; SOT, Satisfaction with the online treatment questionnaire

To retain as many participants as possible, reminder emails will be sent to participants 24 hours before each scheduled session becomes available, and also at 13:00 on the scheduled session date should the participant not have already logged into their ABMT account. An additional reminder email will be sent one week before their 6-month follow-up session is ready. Participants will also be sent encouragement emails informing them when they have reach specific milestones (i.e., successfully completing 5 sessions for those randomised to complete 10 sessions; successfully completing 6 and 12 sessions for those randomised to 18 sessions). Participants will also have the option of receiving an SMS message on their mobile phone at 09:00 on the morning of each scheduled session.

The Intervention

The intervention will be hosted using LifeGuide and iSurvey platforms, both developed by the University of Southampton. LifeGuide is an open-source software which allows researchers to create online interventions.[37] iSurvey is a survey generation tool which allows researchers to create and disseminate questionnaires online. Participants will register for the intervention using LifeGuide, which will also host the majority of the questionnaires. iSurvey will be used to host the visual-probe tasks, along with the Engagement with Online Training questionnaire and the Brief Pain Inventory[38] that appear at the end of every visual-probe task session. The flow of sessions is provided in Figure 2, and examples of webpages are shown in Figure 3. On loading the intervention homepage, the individual is provided with a brief welcome to the website and rationale for the intervention. A recent review of cognitive bias meta-analyses concluded that effect sizes were smaller when biases were modified remotely compared to laboratory-based training.[39] We therefore decided to provide information on the rationale behind

For peer review only - http://bmjopen.bmj.com/site/about/guidelines.xhtml

ABMT in order to increase motivation and reduce attrition as much as possible, which also mirrors clinical practice that involves educating patients into chronic pain and the psychological interventions used in its management. Individuals are able to either create an iABMT account or log into their existing account. Before any session participants are asked to indicate whether they have enough time and are ready to begin the session. Participants are only permitted to complete one session per day. Should a participant complete their missed session on a subsequently scheduled date (e.g., completing their scheduled session from Monday on Thursday instead), all remaining sessions are shifted back accordingly. This process retains the structure of the intervention.

Guidance has been informed by research showing attention to be a limited capacity resource[40 41], sleepiness to negatively influence concentration and performance[42], alcohol to impair speed of information processing and cognition[43], energy drinks to be associated with restlessness and nervousness[44], caffeine to improve alertness[45], large meals to be associated with impairments in cognitive functioning[46], and hunger to influence patterns of attention.[47 48] Participants are provided with guidance to ensure concentration and comfort is maintained throughout each session: (1) allowing enough time for the session with no anticipated distractions, (2) adjusting screen brightness to match the brightness of the environment, (3) closing distracting applications such as Twitter and Facebook, (4) avoiding completing the session when tired, (5) not consuming alcohol at least 3 hours prior to the session, (6) caution in the use of energy drinks which may cause restlessness, (7) if the participant is a coffee or tea drinker, suggesting a cup 30 minutes prior to the session, and (8) avoiding completing the session when hungry or directly after a meal.

Visual-Probe Tasks. The flow of visual-probe sessions is illustrated in Figure 2. In total there will be eight linguistic and eight pictorial visual-probe training tasks used in the study, each with different stimuli. A complete description of the stimuli including ratings of valence, arousal, pain intensity, written frequency, and low-level features, is provided in Supplementary Material

For peer review only - http://bmjopen.bmj.com/site/about/guidelines.xhtml

ATTENTIONAL BIAS MODIFICATION PROTOCOL

1. To briefly summarise, each linguistic visual-probe task includes 32 threatening/neutral and 16 neutral/neutral word pairs. Each task includes eight threatening words from each of the following categories: sensory-pain, affective-pain, health-threat and general-threat. Each pictorial task includes 32 threatening/neutral and 16 neutral/neutral image pairs. Each task includes eight threatening images from the following categories: musculoskeletal pain, facial expressions of pain, health-threat and general-threat. A total of 384 trials are included in each task, with each stimuli-pair presented four times for 500 ms and four times for 1250 ms. Within each exposure duration, each stimulus appears twice in the upper location of the screen, and twice in the lower location.

A separate linguistic visual-probe task will be used in the three assessment sessions, featuring different stimuli to the linguistic training visual-probe tasks. The use of separate assessment and training stimuli is necessary to establish whether training effects generalise to novel stimuli not used in the actual training. The same assessment stimuli will be used in all assessment sessions. The ABM training version of the visual-probe task to be completed by AMG groups includes three main stages per trial (Figure 1). Following a 500 ms initial fixation point, a stimulus pair are presented in distinct locations (e.g., above and below the initial fixation point) for either 500 or 1250 ms. The stimulus pair may be either words or images, although critically includes one threat-related and one neutral stimulus. 500 and 1250 ms stimuli presentation times are used as these are the most common stimuli presentation times adopted in chronic pain visual-probe studies (e.g., [18 49 50]), and will allow us to closely compare our results to those of former research. Immediately following their presentation, the stimuli disappear and a probe appears (i.e., either *'p'* or *'q'*) in the location of the neutral stimulus. Participants indicate the location of the probe as quickly and as accurately as possible via corresponding keys on their keyboard. In the standard version of this paradigm completed by PTG groups and used in the assessment sessions, the probe replaces the neutral and threat-related stimulus an equal number of times.

For peer review only - http://bmjopen.bmj.com/site/about/guidelines.xhtml

Outcome Measures

The primary outcome measures of pain intensity and pain interference will be assessed by the Brief Pain Inventory – Short Form (BPI-SF[38]). The secondary outcome measures of state and trait anxiety will be assessed by the State Trait Anxiety Inventory (STAI [51]), depression by the Hospital Anxiety and Depression Scale (HADS[52]), pain-related fear by the Fear of Pain Questionnaire III (FOP-III[53]), sleeping impairment by the Medical Outcome Study – Sleep Scale (MOS-SS[54]), and engagement to and satisfaction with the online visual-probe training task by the Satisfaction with the Online Training questionnaire (SOT[55]) and the Engagement with the Online Training questionnaire (EOT). A demographic questionnaire developed by the researchers will be used at baseline to collect information on participants' age, gender, ethnicity, socio-economic status, place of residence, and pain characteristics. Aside from the SOT and EOT, questionnaires in the 10 session groups (10AMG and 10PTG) will be administered to participants at baseline, after 10 sessions, and at six-month follow-up. In the 18 session groups (18AMG and 18PTG) questionnaires will be administered to participants at baseline, after 10 sessions, after 18 sessions, and at six-month follow-up. At endline for each participant we will also assess whether the participant and researchers became aware at any point prior to endline which group they had been allocated to. Full details on each outcome measure are provided in Supplementary Material 2.

Think Aloud Study

A qualitative think aloud study[34] including a short semi-structured interview was conducted following the development of the initial version with the aim to understand participants' first impressions and attitudes towards the intervention, and to collate feedback that could be used to make improvements. Details and outcomes of this study are provided in Supplementary Material 3.

Analyses of the RCT Data

Quantitative Analyses

For peer review only - http://bmjopen.bmj.com/site/about/guidelines.xhtml

ATTENTIONAL BIAS MODIFICATION PROTOCOL

A full and detailed Statistical Analysis Plan will be written prior to trial closure. In brief, the data analysis will be performed on an intention-to-treat (ITT) basis to provide an unbiased estimate of the treatment effect.[56] Per protocol analyses will also be performed (comprising those correctly randomized, without protocol violations, and providing complete data on the primary outcome measure). A linear mixed model with random intercepts will be used to analyze the data. This approach avoids listwise deletion which would apply in a repeated measures ANOVA approach. The linear mixed modelling approach uses all of the available data, and adheres to ITT. As a sensitivity analysis we will used multiple imputation chained equations (MICE) to impute outcome data[57 58] and uses these imputed data sets in the linear mixed model. Pairwise comparisons will follow the same procedure. The analysis plan would apply to the primary (i.e., pain intensity and pain interference) and secondary outcome measures. Similar to former studies[28 29] the Reliable Change Index (RCI)[59] will be used to assess primary and secondary outcome measures for changes over and above changes which could otherwise be attributed to measurement unreliability. Here, the minimum clinically significant result occurs if the post-treatment score differs from the pre-treatment score by the minimum detectable difference at the 95% level (i.e. by 2 standard deviations). In contrast to tests of statistical significance comparing group means, an assessment of the RCI is an exploration of effects of the treatment on the individual.[60]

For visual-probe data, and as per former research[19 29], practice and incorrect trials will be removed prior to analyses. Box and whisker plots for overall data will be used to reveal overall outliers, which will be removed. Following this, mean response times will be computed for each participant, with any response >3SD from their individual mean also removed as outliers. This process ensures extremely quick or slow responses do not unduly bias the results, which are typically removed when cleaning and screening visual-probe data in pain-related research (e.g., [18 61 62 63 64]). An attentional bias index will then be computed for each stimulus and presentation time condition using the following equation: $(TuPl - TlPl)+(TlPu - TuPu))/2$. Here T is the

For peer review only - http://bmjopen.bmj.com/site/about/guidelines.xhtml

threatening stimulus, P is the probe, u is the upper position, and l is the lower position. The attention capturing quality of threatening stimuli is measured by subtracting the mean probe detection time for congruent trials from the mean probe detection time of incongruent trials.[21]

Split-half reliability will be computed via a bootstrap procedure, by randomly splitting the total number of trials in two halves, such that each half has the same number of congruent and incongruent trials. Attentional bias scores will be computed for each half and the correlation between them calculated across participants. This procedure will be repeated 100 times, and an average split-half correlation computed

Qualitative Analyses

Qualitative analysis will be used to detail participants' experiences completing the ABMT intervention. Specifically, thematic analysis using inductive coding will be used to identify themes present in the data obtained from the semi-structured interviews and the two open-ended questions included in the Satisfaction with the Online Training questionnaire. Guidelines provided by Braun and Clarke[65] will be followed. Following transcription of all interviews verbatim, initial codes will be generated. Potential themes and sub-themes will then be identified from the initial coding, which subsequently will be reviewed and refined. The validity of the themes in relation to the data set will also be considered. Each theme and sub-theme will then be named and defined, and a thematic map of the data finalized. Examples from the data will be illustrated for each theme and sub-theme in the final report.

Ethics and Dissemination

This study has been approved by the University of Southampton Research Ethics Committee (ERGO ID: 26486), and the study protocol registered with ClinicalTrial.gov (NCT02232100). Ethical approval will be sought before any protocol modifications are made and the study protocol will be updated at ClinicalTrial.gov. Participants will provide informed consent prior to taking part in the study. All data will remain confidential and stored on password-protected systems and databases. Lifeguide and iSurvey servers both use HTTPS

For peer review only - http://bmjopen.bmj.com/site/about/guidelines.xhtml

connections for security purposes. All data will be anonymised prior to dissemination, and personally identifiable information not known to anyone other than the researchers. Participants may withdraw from the intervention at any point by contacting the researchers via email or telephone, or by simply opting not to log into their iABMT account for their scheduled sessions. Participants are also encouraged to contact the researchers should they experience any distress or discomfort completing the intervention. As per University policy, any adverse events, harms or complaints arising from participation in the intervention will be reported to the University of Southampton Research Governance Office. Findings will be published in the most relevant, high-impact peer-reviewed journals and presented at relevant conferences. Lay reports will be written for interested pain charities and patient support groups.

List of figures and supplementary files

Table 1: Schedule of enrolment, intervention, and assessments.

Figure 1. A graphical representation of a typical visual-probe task training trial with linguistic stimuli.

Figure 2. Flow of the visual-probe task sessions for the 10-week and 18-week groups in the study

Figure 3. Examples of Lifeguide webpages included in the iABMT intervention.

Supplementary Material 1 – Stimuli development and properties

Supplementary Material 2 – Outcome Measures

Supplementary Material 3 – Think Aloud Study

For peer review only

References

1. Sambrook P, Taylor T, Schrieber L, et al. *The Musculoskeletal System: Basic Science and Clinical Conditions*. Second ed: Churchill Livingstone, Elsevier, 2010.

2. Cimmino MA, Ferrone C, Cutolo M. Epidemiology of chronic musculoskeletal pain. Best Practice & Research Clinical Rheumatology 2011;**25**(2):173-83 doi: 10.1016/j.berh.2010.01.012.

3. King S, Chambers CT, Huguet A, et al. The epidemiology of chronic pain in children and adolescents revisited: a systematic review. Pain 2011;**152**(12):2729-38 doi: 10.1016/j.pain.2011.07.016.

4. Tüzün EH. Quality of life in chronic musculoskeletal pain. Best Practice & Research Clinical Rheumatology 2007;**21**(3):567-79 doi: http://dx.doi.org/10.1016/j.berh.2007.03.001.

5. Breivik H, Collett B, Ventafridda V, et al. Survey of chronic pain in Europe: prevalence, impact on daily life, and treatment. Eur J Pain 2006;**10**(4):287-333 doi: 10.1016/j.ejpain.2005.06.009.

6. Bair MJ, Wu J, Damush TM, et al. Association of depression and anxiety alone and in combination with chronic musculoskeletal pain in primary care patients. Psychosom Med 2008;**70**(8):890-7 doi: 10.1097/PSY.0b013e318185c510.

7. Blyth FM, Noguchi N. Chronic musculoskeletal pain and its impact on older people. Best Practice & Research Clinical Rheumatology 2017;**31**(2):160-68 doi: 10.1016/j.berh.2017.10.004.

8. Harrison L, Wilson S, Munafò MR. Exploring the associations between sleep problems and chronic musculoskeletal pain in adolescents: a prospective cohort study. Pain research and management 2014;**19**(5):e139-e45 doi: 10.1155/2014/615203.

For peer review only

For peer review only - http://bmjopen.bmj.com/site/about/guidelines.xhtml

9. Flatø B, Aasland A, Vandvik I, et al. Outcome and predictive factors in children with chronic idiopathic musculoskeletal pain. Clinical and Experimental Rheumatology 1997;**15**(5):569-77.

10. Gold JI, Mahrer NE, Yee J, et al. Pain, fatigue and health-related quality of life in children and adolescents with chronic pain. Clinical Journal of Pain 2009;**25**(5):407 doi: 10.1097/AJP.0b013e318192bfb1.

11. Vinall J, Pavlova M, Asmundson GJ, et al. Mental health comorbidities in pediatric chronic pain: A narrative review of epidemiology, models, neurobiological mechanisms and treatment. Children 2016;**3**(4):40 doi: 10.3390/children3040040.

12. Pincus T, Morley S. Cognitive-processing bias in chronic pain: a review and integration. Psychol Bull 2001;**127**(5):599 - 617 doi: 10.1037/0033-2909.127.5.599.

13. Todd J, Sharpe L, Johnson A, et al. Towards a new model of attentional biases in the development, maintenance, and management of pain. Pain 2015;**156**(9):5189-1600 doi: 10.1097/j.pain.0000000000000214.

14. Vlaeyen JWS, & Linton, S. J. Fear-avoidance and its consequences in chronic musculoskeletal pain: a state of the art. Pain 2000;**85**(3):317 - 32.

15. Liossi C, Schoth DE, Bradley BP, et al. Time-course of attentional bias for pain-related cues in chronic daily headache sufferers. European Journal of Pain 2009;**13**(9):963-69 doi: http://dx.doi.org/10.1016/j.ejpain.2008.11.007.

16. Haggman SP, Sharpe, L. A., Nicholas, M. K., & Refshauge, K. M. Attentional biases toward sensory pain words in acute and chronic pain patients. The Journal of Pain 2010;**11**(11):1136 - 45.

17. Sharpe L, Dear BF, Schrieber L. Attentional biases in chronic pain associated with rhumatoid arthritis: Hypervigilance or difficulties disengaging? J Pain 2009;**10**(3):329 - 35 doi: 10.1016/j.jpain.2008.10.005.

For peer review only

18. Schoth DE, Beaney R, Broadbent P, et al. Attentional, interpretation and memory biases for sensory-pain words in individuals with chronic headache. British Journal of Pain 2019;**13**(1):22 - 31 doi: 10.1177/2049463718789445.

19. Schoth D, Liossi C. Specificity and time-course of attentional bias in chronic headache: a visual-probe investigation. Clinical Journal of Pain 2013;**29**(7):583-90 doi: 10.1097/AJP.0b013e31826b4849.

20. Schoth DE, Liossi C. Attentional bias toward pictorial representations of pain in individuals with chronic headache. Clin J Pain 2010;**26**(3):244-50 doi: 10.1097/AJP.0b013e3181bed0f9.

21. Schoth D, Delgado Nunes V, Liossi C. Attentional bias towards pain-related information in chronic pain; a meta-analysis of visual-probe investigations. Clinical Psychology Review 2012;**32**(1):13 - 25 doi: 10.1016/j.cpr.2011.09.004.

22. Crombez G, Van Ryckeghem D, Eccleston C, et al. Attentional bias to pain-related information: a meta-analysis. Pain 2013;**154**(4):497-510.

23. Todd J, van Ryckeghem DM, Sharpe L, et al. Attentional bias to pain-related information: A meta-analysis of dot-probe studies. Health Psychol Rev 2018;**12**(4):419-36.

24. Crombez G, Van Ryckeghem D, Eccleston C, et al. Attentional bias to pain-related information: a meta-analysis. Pain 2013;**154**(4):497-510 doi: 10.1016/j.pain.2012.11.013.

25. Schoth DE, Delgado Nunes V, Liossi C. Attentional bias towards pain-related information in chronic pain; a meta-analysis of visual-probe investigations. Clin Psychol Rev 2012;**32**(1):13 - 25 doi: 10.1016/j.cpr.2011.09.004.

26. MacLeod C, Mathews, A., & Tata, P. Attentional Bias in Emotional Disorders. Journal of Abnormal Psychology 1986;**95**(1):15 - 20.

27. Sharpe L, Ianiello, M., Dear, B. F, Nicholson Perry, K., Refshauge, K., Nicholas, M. K. Is there a potential role for attention bias modification in pain patients? Results of 2 randomised, controlled trials. Pain 2012;**153**(3):722-31.

For peer review only - http://bmjopen.bmj.com/site/about/guidelines.xhtml

28. Carleton R, Richter A, Asmundson G. Attention Modification in Persons with Fibromyalgia: A Double Blind, Randomized Clinical Trial. Cognitive Behaviour Therapy 2011;**40**(4):279-90.

29. Schoth DE, Georgallis T, Liossi C. Attentional bias modification in people with chronic pain: a proof of concept study. Cognitive Behaviour Therapy 2013;**42**(3):233-43.

30. Carleton RN, Richter AA, Asmundson GJG. Attention Modification in Persons with Fibromyalgia: A Double Blind, Randomized Clinical Trial. Cogn Behav Ther 2011;**40**(4):279-90 doi: 10.1080/16506073.2011.616218.

31. Sharpe L, Ianiello M, Dear BF, et al. Is there a potential role for attention bias modification in pain patients? Results of 2 randomised, controlled trials. Pain 2012;**153**(3):722-31 doi: 10.1016/j.pain.2011.12.014.

32. Curriculum for a CCT in Anaesthetics (2010). Secondary Curriculum for a CCT in Anaesthetics (2010). https://www.rcoa.ac.uk/document-store/curriculum-cct-anaesthetics-2010.

33. Heathcote LC, Jacobs K, Van Ryckeghem DML, et al. Attention bias modification training for adolescents with chronic pain: a randomized placebo-controlled trial. Pain 2018;**159**(2):239 - 51 doi: 10.1097/j.pain.0000000000001084.

34. Hamilton F. Perspectives of individuals with chronic musculoskeletal pain towards an online attentional bias modification training programme. University of Southampton, 2015.

35. Hakamata Y, Lissek, S., Bar-Haim, Y., Britton, J.C., Fox, N.A., Leibenluft, E., Ernst, M., & Pine, D. S. Attention bias modification treatment: A meta-analysis towards the establishment of novel treatment for anxiety. Biol Psychiatry 2010;**68**:982-90 doi: 10.1016/j.biopsych.2010.07.021.

36. Mogoaşe C, David D, Koster EH. Clinical efficacy of attentional bias modification procedures: An updated meta-analysis. J Clin Psychol 2014;**70**(12):1133-57 doi: 10.1002/jclp.2208.

For peer review only - http://bmjopen.bmj.com/site/about/guidelines.xhtml

37. Williams S, Yardley L, Wills GB. A qualitative case study of LifeGuide: users' experiences of software for developing Internet-based behaviour change interventions. Health Informatics J 2013;**19**(1):61-75 doi: 10.1177/1460458212458915.

38. Cleeland CS, Ryan KM. Pain assessment: global use of the Brief Pain Inventory. Ann Acad Med Singapore 1994;**23**(2):129-38.

39. Jones EB, Sharpe L. Cognitive bias modification: A review of meta-analyses. J Affect Disord 2017;**223**:175-83 doi: 10.1016/j.jad.2017.07.034.

40. Kahneman D. *Attention and effort*. New Jersey: Prentice Hall, 1973.

41. Broadbent D. *Perception and communication*. Oxford: Pergamon, 1958.

42. Thomas M, Sing H, Belenky G, et al. Neural basis of alertness and cognitive performance impairments during sleepiness. I. Effects of 24 h of sleep deprivation on waking human regional brain activity. Journal of Sleep Research 2000;**9**(4):335-52 doi: 10.1046/j.1365-2869.2000.00225.x.

43. Tzambazis K, Stough C. Alcohol impairs speed of information processing and simple and choice reaction time and differentially impairs higher-order cognitive abilities. Alcohol and Alcoholism 2000;**35**(2):197-201 doi: 10.1093/alcalc/35.2.197.

44. Reissig CJ, Strain EC, Griffiths RR. Caffeinated energy drinks—a growing problem. Drug and Alcohol Dependence 2009;**99**(1):1-10 doi: 10.1093/alcalc/35.2.197.

45. Zwyghuizen-Doorenbos A, Roehrs TA, Lipschutz L, et al. Effects of caffeine on alertness. Psychopharmacology 1990;**100**(1):36-39 doi: 10.1007/BF02245786.

46. Müller K, Libuda L, Terschlüsen AM, et al. A review of the effects of lunch: On adults' short-term cognitive functioning. Canadian Journal of Dietetic Practice and Research 2013;**74**(4):181-88 doi: 10.3148/74.4.2013.181.

47. Piech RM, Pastorino MT, Zald DH. All I saw was the cake. Hunger effects on attentional capture by visual food cues. Appetite 2010;**54**(3):579-82 doi: 10.1016/j.appet.2009.11.003.

For peer review only

For peer review only - http://bmjopen.bmj.com/site/about/guidelines.xhtml

48. Al-Shawaf L. The evolutionary psychology of hunger. Appetite 2016;**105**:591-95 doi: 10.1016/j.appet.2016.06.021.

49. Schoth DE, Liossi C. Attentional bias towards pictorial representations of pain in individuals with chronic headache. Clin J Pain 2010;**26**(3):244 - 50 doi: 10.1097/AJP.0b013e3181bed0f9.

50. Schoth DE, Liossi C. Specificity and time-course of attentional bias in chronic headache: a visual-probe investigation. Clin J Pain 2013;**29**(7):583-90 doi: 10.1097/AJP.0b013e31826b4849.

51. Spielberger CD, Gorsuch RL, Lushene RE. *State Trait Anxiety Inventory*. Palo Alto, California: Consulting Psychologists Press, 1970.

52. Zigmond AS, Snaith RP. The hospital anxiety and depression scale. Acta Psychiatr Scand 1983;**67**(6):361-70.

53. McNeil DW, Rainwater A. Development of the Fear of Pain Questionnaire-III. Journal of Behavioral Medicine 1998;**21**(4):389-410 doi: 10.1023/A:1018782831217.

54. Hays RD, Stewart AL. Sleep Measures. Measuring functioning and well-being. In: Steweart AL, JEJ W, eds. Measuring function and well-being; the medical outcome study approach. Duke: Duke University Press, 1992:235-59.

55. Richards D, Timulak L. Satisfaction with therapist-delivered vs. self-administered online cognitive behavioural treatments for depression symptoms in college students. British Journal of Guidance & Counselling 2013;**41**(2):193-207 doi: 10.1080/03069885.2012.726347.

56. Gupta SK. Intention-to-treat concept: a review. Perspectives in Clinical Research 2011;**2**(3):109 doi: 10.4103/2229-3485.83221.

57. Chan A-W, Tetzlaff JM, Gøtzsche PC, et al. SPIRIT 2013 explanation and elaboration: guidance for protocols of clinical trials. BMJ 2013;**346**:e7586 doi: 10.1136/bmj.e7586.

For peer review only

58. Groenwold RH, Donders ART, Roes KC, et al. Dealing with missing outcome data in randomized trials and observational studies. American Journal of Epidemiology 2012;**175**(3):210-17 doi: 10.1093/aje/kwr302.

59. Jacobson NS, Truax P. Clinical significance: A statistical approach to defining meaningful change. Psychotherapy Research 1991;**59**:12-19 doi: 10.1037/0022-006X.59.1.12.

60. Zahra D, Hedge C. The reliable change index: Why isn't it more popular in academic psychology? Psychology Postgraduate Affairs Group Quarterly 2010;**76**(76):14-19.

61. Schoth DE, Parry L, Liossi C. Combined cognitive biases for sensory-pain and disability information in individuals with chronic headache. J Health Psychol 2018;**23**(12):1610-21 doi: 10.1177/1359105316664136.

62. Dear BF, Sharpe L, Nicholas M, et al. Pain-related attentional biases: The importance of the personal relevance and ecological validity of stimuli. J Pain 2011a;**12**(6):625-32.

63. Schoth DE, Williams S, Liossi C. Attentional bias for pain- and period-related symptom words in healthy women who experienced a recent painful period. Eur J Pain 2015;**19**(6):745-51 doi: 10.1002/ejp.597.

64. McDermott MJ, Peck KR, Walters AB, et al. Do episodic migraineurs selectively attend to headache-related visual stimuli? Headache: The Journal of Head and Face Pain 2013;**53**(2):356-64 doi: 10.1111/head.12011.

65. Braun V, Clarke V. Using thematic analysis in psychology. Qualitative Research in Psychology 2006;**3**(2):77-101 doi: 10.1191/1478088706qp063oa.

For peer review only

For peer review only - http://bmjopen.bmj.com/site/about/guidelines.xhtml

Authors' contributions: All authors have contributed to the development of the study protocol. CL, TG and DES conceived the project. CL and DES registered the trial and are responsible for recruitment and implementation of the protocol. TG was primary responsible for the development of the online materials, including development and validation of linguistic and pictorial stimuli used in the visual-probe tasks. TG also assisted in programming the Lifeguide components. JZ was responsible for programming the visual-probe tasks to be used in the intervention, and assisted in programming the Lifeguide components. FH conducted the think aloud study. PW was responsible for development of the statistical analysis plan. All authors contributed to the writing of this manuscript, and approve its final form. These same authors will be included on future publications presenting intervention results, and will have access to the cleaned study datasets. **Funding**: This work was supported by the Economic and Social Research Council (grant number: RES-000-22-4128). This funding source had no role in the design of this study and will not have any role during its execution, analyses, interpretation of the data, or decision to submit results. **Competing interests**: The authors declare that they have no competing interest.

For peer review only

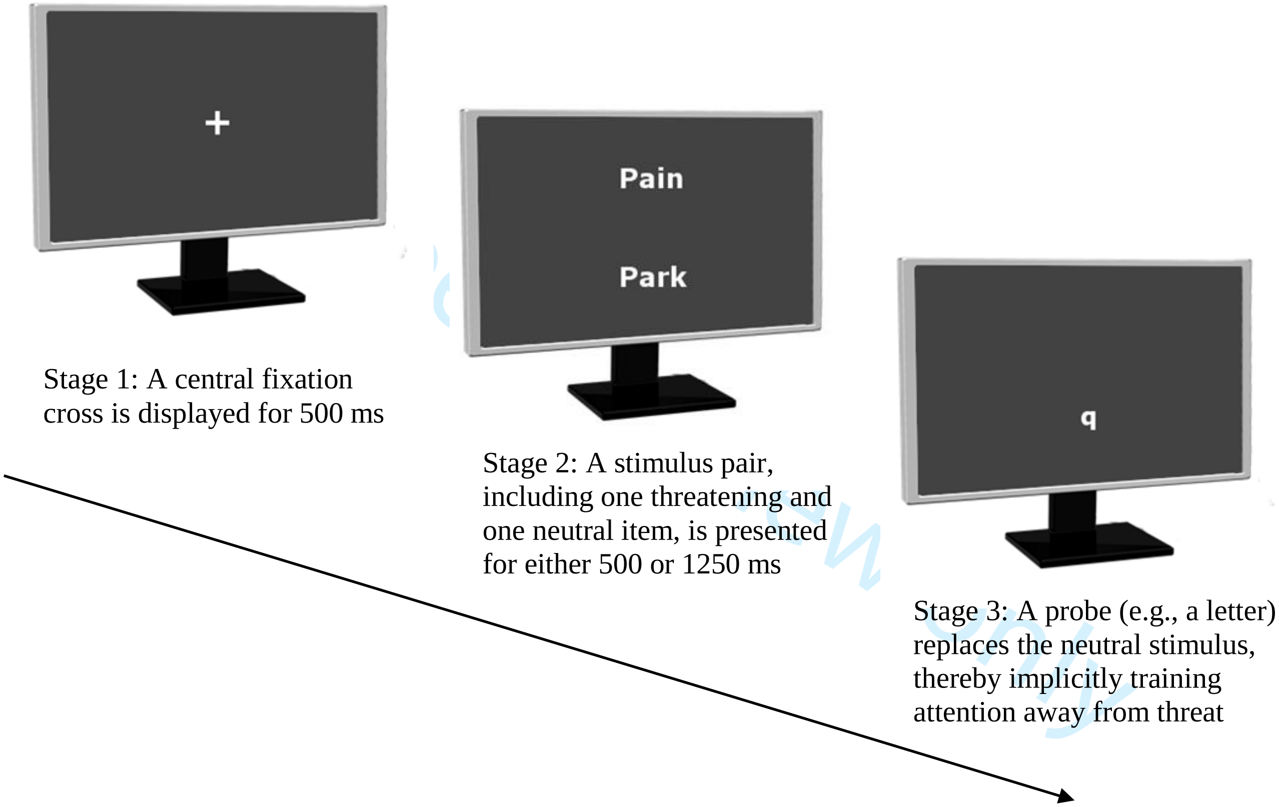

Figure 1. A graphical representation of a typical visual-probe task training trial with linguistic stimuli.

For peer review only - http://bmjopen.bmj.com/site/about/guidelines.xhtml

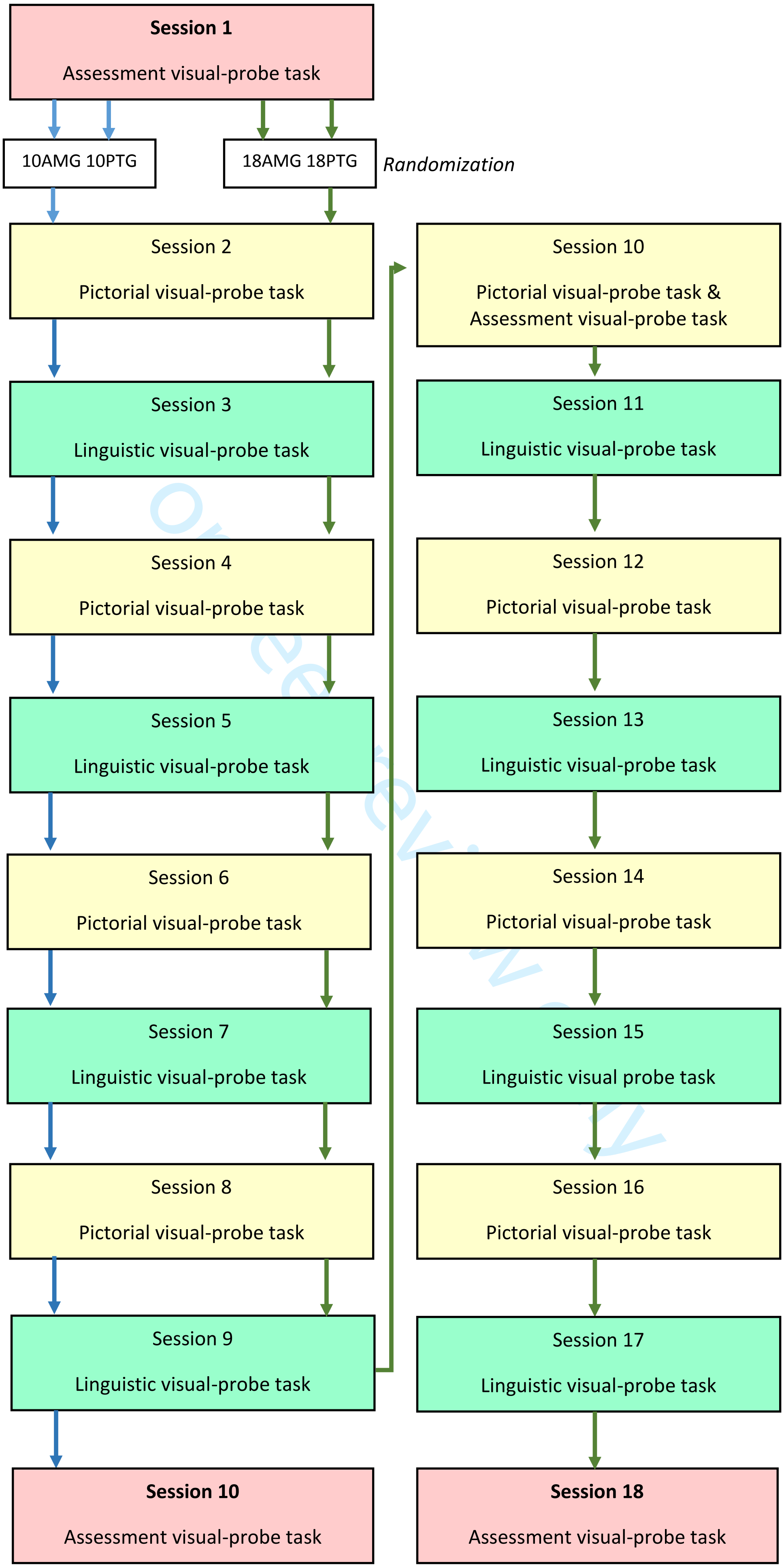

Figure 2. Flow of the visual-probe task sessions for the 10-week and 18-week groups in the study.

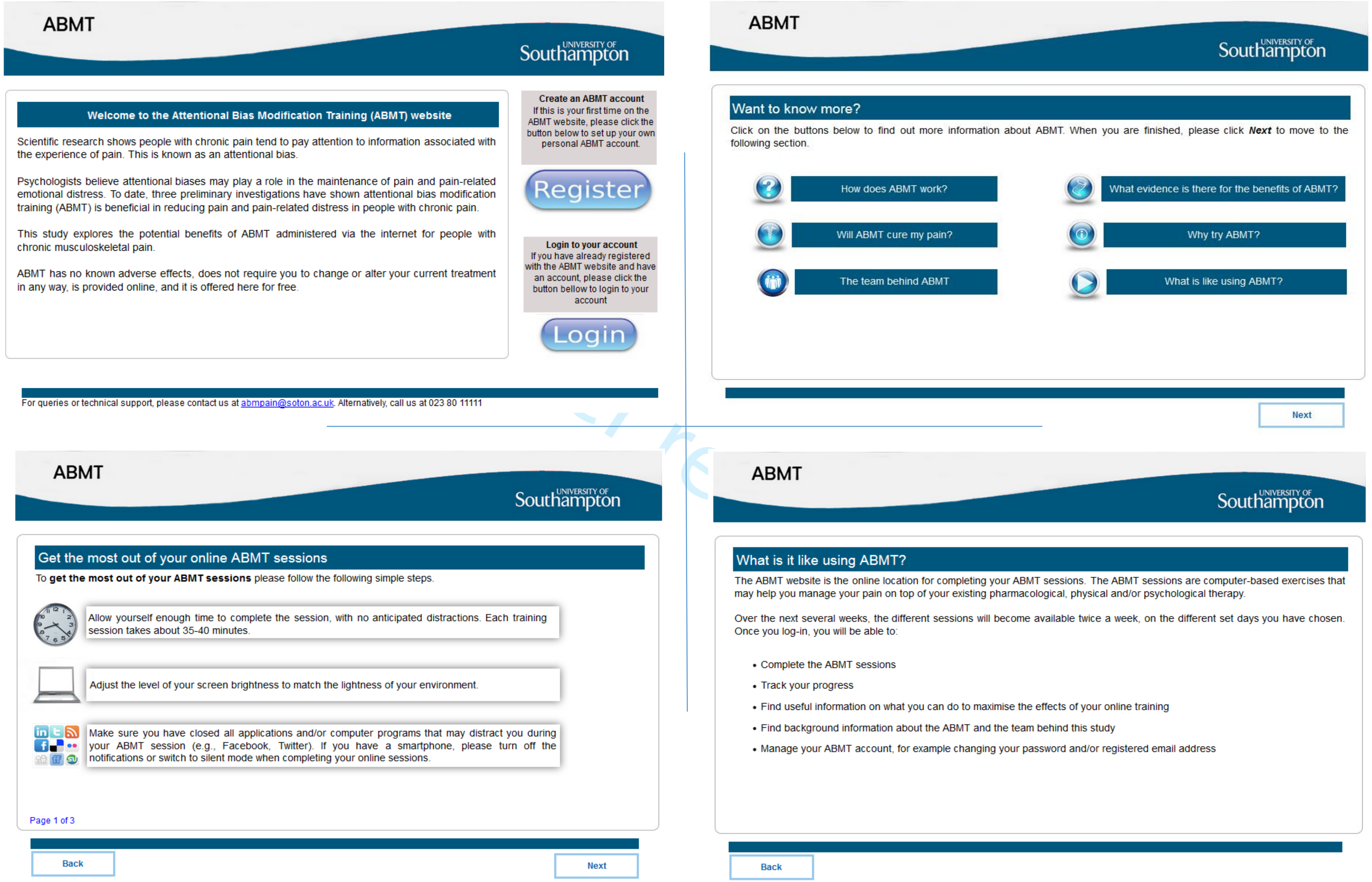

Figure 3. Examples of Lifeguide webpages included in the iABMT intervention.

Supplementary Material 1

Stimuli Development and Properties

A detailed description of the stimuli to be used in the iAMBT intervention is presented in this document. Linguistic stimulus properties and analyses on written frequency, valence, and arousal are first presented, followed by a description of pictorial stimuli properties and analyses of low-level features, valence, arousal and pain intensity. Following this, comparisons of stimuli properties between visual-probe sessions are then presented for linguistic and pictorial stimuli.

Linguistic stimuli

Properties. Linguistic and pictorial versions of the visual-probe task will be used. Each linguistic visual-probe task will include 32 threatening/neutral and 16 neutral/neutral word pairs. Threatening words stem from the following categories, with 8 words from each included in each visual-probe task: (i) sensory-pain words reflecting the sensory dimension of pain (e.g., *shooting*; *burning*; *aching*); (ii) affective-pain words reflecting the unpleasantness of pain and negative emotions associated with pain (e.g., *hopeless*, *punishing*, *tormenting*); (iii) health-threat words reflecting threat to health and well-being (e.g., *hospital*, *doctor*, *stress*); and (iv) general-threat words reflecting physical and emotional sources of threat, including manmade threat (e.g., *gun*), natural threat (e.g., *fire*), social threat (e.g., *humiliation*), and emotional threat (e.g., *sad*). Neutral words (including the neutral counterparts of the threatening words) include non-threatening words unrelated to pain or suffering (e.g., *version*; *rector*; *monument*), which were matched with their threatening counterparts on Kucera-Francis written frequency of use in the English language[1] and word length. Matching on written frequency minimise the chances of participants preferentially processing one stimulus due to its high frequency of use (e.g., faster recognition) when displayed simultaneously with another stimulus[2 3], while matching word length also minimise the chances of participants attending towards a stimulus due to its size.

In total, 864 words have been collected from previous studies exploring and modifying biases in pain and/or anxiety (i.e.,[4-8]), including words from the McGill Pain Questionnaire.[9]

For peer review only - http://bmjopen.bmj.com/site/about/guidelines.xhtml

ATTENTIONAL BIAS MODIFICATION PROTOCOL

Matching of threatening and neutral words was achieved via the MRC Psycholinguistic database[10], an online psycholinguistic tool that generates words with specific lexical characteristics. The database includes a dictionary of 150,837 English words providing syntactic, semantic and orthographical properties of each word.[11] Valence and arousal can influence attentional allocation to external stimuli.[12 13] A computerised version of the SAM task[14] was therefore used to collect ratings on valence and arousal for each word in a sample of sixteen healthy, pain-free participants (M_{age} = 25.9, SD = 4.08, range: 20 – 33 years; 8 female). All words were randomly presented to participants for 3 seconds each. Following each word, two 9-point SAM scales were presented, one for valence (1 = low pleasure to 9 = high pleasure) and one for arousal (1 = low arousal to 9 = high arousal). As per former chronic pain attentional bias research (e.g.,[5 15 16]), stimuli categories were assessed but not matched on valence and arousal, as differences are expected to exist between categories.

Analyses and results: written frequency, valence, and arousal. Analyses comparing properties between the five word categories are presented here, while analyses comparing word properties between the nine linguistic visual-probe tasks are presented below. One-way ANOVA was conducted comparing Kucera-Francis written frequency (Table 1) across words in the five word categories, revealing no significant main effect, $F(4, 853) = 1.97$, $p = .10$.

A repeated-measures ANOVA was conducted comparing arousal ratings (Table 2) across the five word categories, revealing a significant main effect, $F(2, 25) = 18.78$, $p < .001$, $\eta p^2 = .56$. Pairwise comparisons revealed the neutral category had significantly lower arousal scores than sensory-pain (*Md* [mean difference] = 1.82, *SE* = 0.45, *p* = .001), affective-pain (*Md* = 2.36, *SE*= 0.46, *p* = .001), general-threat (*Md* = 2.20, *SE* = 0.47, *p* = .003) and health-threat (*Md* = 2.05, *SE* = 0.35, *p* < .001) categories. Statistically significant differences were not found between the arousal scores of the words included in the four threat-related categories.

For peer review only - http://bmjopen.bmj.com/site/about/guidelines.xhtml

Table 1

Mean Kucera-Francis Written Frequency per Word Category

Word category	*M*	*SD*
Sensory-pain	19.07	27.01
Affective-pain	17.28	20.63
General-threat	25.00	32.10
Health-threat	21.36	36.39
All neutral words	22.60	27.97

Table 2

Mean Arousal and Valence Ratings per Word Category

	Arousal		Valence	
Word Category	*M*	*SD*	*M*	*SD*
Sensory-pain	3.80	1.85	3.95	0.83
Affective-pain	4.34	1.64	3.29	0.73
General-threat	4.19	1.78	3.35	0.79
Health-threat	4.03	1.32	3.48	0.68
Sensory-pain neutral counterparts	2.18	1.28	5.33	0.41
Affective-pain neutral counterparts	2.19	1.32	5.29	0.29
General-threat neutral counterparts	2.06	1.28	5.24	0.33
Health-threat neutral counterparts	2.05	1.19	5.19	0.36
Neutral/Neutral	1.95	1.32	5.29	0.33

A repeated-measures ANOVA was conducted comparing valence ratings (Table 2) across the five word categories, revealing a significant main effect, $F(2, 29) = 52.00$, $p < .001$, $\eta p^2 = .78$. Pairwise comparisons showed the neutral category had significantly higher pleasantness scores than sensory-pain (*Md* = 1.33, *SE* = 0.23, *p* <. 001) affective-pain (*Md* = 1.99, *SE* = 0.22, *p* < .001), general-threat (*Md* = 1.93, *SE* = 0.23, *p* <. 001) and health-threat (*Md* = 1.80, *SE* = 0.32, *p* < .001) categories. The words in the sensory-pain category were rated as more pleasant than

For peer review only - http://bmjopen.bmj.com/site/about/guidelines.xhtml

words included in the affective-pain (Md = 0.66, SE = 0.11, $p < .001$), general-threat (Md = 0.60, SE = 0.11, $p < .001$) and health-threat (Md = 0.47, SE = 0.11, $p = .01$) categories.

Pictorial Stimuli

Properties. Pictorial visual-probe tasks each include 32 threatening/neutral and 16 neutral/neutral image pairs. Threatening images stem from the following categories: (i) musculoskeletal pain images depicting people experiencing pain in muscles and/or joints, including images of people performing daily activities that can be considered painful and/or harmful for individuals with CMP (e.g., a person running; a person lifting a heavy weight with poor posture); (ii) facial expressions of pain, featuring a single model depicting a prototypical pain expression – closing the eyes or narrowing the eyelids, lowering the eyebrows, wrinkling the nose or raising the cheek, and partly opening the mouth or extending the lips[17], including horizontal lip stretching and/or oblique lip raising[18]; (iii) health-threat images reflecting threat to health and well-being (e.g., medication, wheelchair); and (iv) general-threat images reflecting physical and emotional sources of threat, including manmade threat (e.g., gun), natural threat (e.g., flood), and social threat (e.g., social isolation). Neutral images (including the neutral pairs of the threatening images) depict non-threatening objects (e.g., a car) and people performing every-day activities (e.g., a person reading a newspaper). In total, 768 images have been collected from previous studies exploring and modifying attentional biases in chronic pain (i.e.,[5] [19]), including online databases providing batteries of pictorial stimuli for research (i.e., International Affective Picture System database[20]; The Photograph Series of Daily Activities Short Version[21]; NimStim Set of Facial Expressions[22]).

Each threatening image depicting a person was paired with a neutral image featuring the same person, with the same clothes and background. Each threatening image depicting an object was matched with a neutral image depicting a similar, non-threatening object. Image pairs in the neutral/neutral condition were matched in the same manner. An equal number of images depicting male and female models were used within each image category. All images were full-

For peer review only - http://bmjopen.bmj.com/site/about/guidelines.xhtml

colour and the same size (i.e., 265 pixels wide x 250 pixels high). Each threatening image was matched with its neutral counterpart on complexity, luminance, contrast level, and colour saturation in blue, red and green channels via visual inspection[23]. Visual attention and gaze fixations are influenced by bottom-up variables, including the saliency of objects[24] and low-level features of visual scenes.[25 26] The additional matching rules for pictorial stimuli in the present study were therefore used to minimise the chances of participants preferentially processing one stimulus due to unnecessary variations in content. Using Adobe Photoshop CS6 Extended, the means and standard deviations for all parameters were extracted for evaluation. Luminance values range from 0 to 255, where 0 indicates no light intensity (i.e., no light emission; black) and 255 indicates the highest light intensity (i.e., absolute light emission; white). Contrast levels values range from -100 to +100, with values closer to +100 indicating more contrasted image. The intensity values for red, green and blue colours range from 0 to 255, where 0 indicates black (i.e., darkest extreme) and 255 white (i.e., lightest extreme) for each colour dimension.

Visual complexity was examined as the size of each compressed image file (.jpeg) in kilobytes under the assumption that more complex images would have higher values in kilobytes.[27] As with linguistic stimuli, a computerised version of the SAM task[14] was used to collect ratings on valence and arousal for each image. Musculoskeletal and facial expressions of pain categories were also evaluated on pain intensity using a 10 cm Visual Analogue Scale (VAS). Participants were required to rate the pain that the person in the image experienced. The scale ranged from 0 to 10, where 0 indicated '*no pain at all*' and 10 indicated '*excruciating pain*'. This rating aimed to assess the degree to which images included in the pain-related categories were perceived as depicting pain. Sixteen healthy, pain-free participants (M_{age} = 24.8, *SD* = 4.56, range: 20 – 33 years; 8 female) were recruited to provide ratings on valence, arousal, and pain intensity (this was a separate sample to that recruited to provide linguistic stimuli ratings).

Analyses and results: Low-level features. Analyses comparing properties between the five image categories on low-level features (Table 3) are presented here, while analyses

For peer review only - http://bmjopen.bmj.com/site/about/guidelines.xhtml

comparing word properties between the eight pictorial visual-probe tasks are provided below. Considering low-level features, one-way ANOVAs revealed no significant main effects for complexity, $F(4,763) = 2.18$, $p = .70$, or luminance, $F(4,763) = 2.31$, $p = .06$. A significant main effect was found for red saturation, $F(4,763) = 5.19$, $p < .001$, $\eta p^2 = .03$. Pairwise comparisons revealed images in the musculoskeletal-pain category had significantly less red saturation than images in the facial expressions of pain (*Md* = 24.24, *SE* = 7.89, $p = .022$) and neutral categories (*Md* = 19.04, *SE* = 5.91, $p = .013$). Images in the general-threat category had significantly less red saturation than facial expressions of pain (*Md* = 23.96, *SE* = 7.89, $p = .025$) and neutral categories (*Md* = 18.76, *SE* = 5.91, $p = .016$). A significant main effect was found for blue saturation, $F(4,763) = 2.74$, $p = .03$, $\eta p^2 = .01$. Images in the general-threat categories had significantly less blue saturation than images in the neutral category (*Md* = 29.64, *SE* = 9.96, $p = .030$). A significant main effect was found for green saturation, $F(4, 763) = 4.19$, $p = .002$, $\eta p^2 = .02$. Images in the general-threat expressions category had significantly less green saturation than images in the neutral category (*Md* = 22.03, *SE* = 6.23, $p = .004$). A significant main effect was found for RMS contrast levels, $F(4,763) = 5.98$, $p < .001$, $\eta p^2 = .03$. Images in the facial expressions of pain category had significantly higher RMS contrast than the images included in musculoskeletal-pain (*Md* = 9.57, *SE* = 3.32, $p = .040$), health-threat (*Md* =13.77, *SE* = 3.32, $p < .001$) and neutral categories (*Md* = 10.89, *SE* = 2.49, $p < .001$).

For peer review only

Table 3

Mean Low-Level values per Image Category

	Musculoskeletal-pain		Facial Expressions of Pain		General-threat		Health-threat		All neutral stimuli	
	M	*SD*	*M*	*SD*	*M*	*SD*	*M*	*SD*	*M*	*SD*
Complexity	20.03	7.17	15.69	3.10	20.14	6.36	19.30	10.78	18.62	10.92
Red Saturation	148.72	41.35	172.95	22.79	148.99	43.43	164.37	57.07	167.76	45.41
Green Saturation	140.39	41.39	153.81	27.56	132.80	41.82	154.92	58.08	154.84	48.60
Blue Saturation	132.53	43.55	144.86	31.64	119.90	48.00	149.09	61.87	149.54	85.49
Luminance	142.01	41.10	158.57	25.50	136.21	41.39	157.37	57.10	162.79	92.44
RMS contrast level	61.25	15.3	70.82	18.75	64.22	18.95	57.00	20.91	59.93	18.86

Analyses and results: Valence, arousal and pain intensity. A repeated-measures ANOVA was conducted comparing arousal ratings (Table 4) across the five image categories, revealing a significant main effect, $F(2,23) = 32.79$, $p < .001$, $\eta p^2 = .69$. Pairwise comparisons revealed that the neutral category had significantly lower arousal scores than facial expressions of pain (*Md* = 2.24, *SE* = 0.45, *p* = .002), musculoskeletal-pain (*Md* = 1.28, *SE* = 0.33, *p* = .014), general-threat (*Md* = 2.86, *SE* = 0.38, *p* < .001), and health-threat (*Md* = 1.91, *SE* = 0.34, *p* = .001) categories. The general-threat category was significantly more arousing than facial expressions of pain (*Md* = 0.62, *SE* = 0.15, *p* = .010), musculoskeletal-pain (*Md* = 1.59, *SE* = 0.14, *p* < .001), and health-threat categories (*Md* = 0.95, SE =0.15, *p* < .001). The musculoskeletal-pain category was also significantly less arousing than the facial expressions of pain (*Md* = 0.96, *SE* = 0.15, *p* < .001) and the health-threat (*Md* = 0.64, *SE* = 0.14, *p* = .005) categories.

For peer review only - http://bmjopen.bmj.com/site/about/guidelines.xhtml

A repeated-measures ANOVA conduced on valence scores (Table 4) across the five image conditions revealed a significant main effect, $F(2,29) = 64.52$, $p < .001$, $\eta p^2 = .82$. Pairwise comparisons showed the neutral category had significantly higher pleasantness scores than facial expressions of pain (*Md* = 1.56, *SE* = 0.15, *p* <. 001), musculoskeletal-pain (*Md* = 0.86, *SE* = 0.13, *p* < .001), general-threat (*Md* = 1.69, *SE* = 0.16, *p*<. 001) and health-threat (*Md* = 1.27, *SE* = 0.18, *p*< .001) categories. Images in the musculoskeletal-pain category were rated significantly more pleasant than the images included in the facial expressions of pain (*Md* = 0.69, *SE* = 0.07, *p* < .001), general-threat (*Md* = 0.83, *SE* = 0.08, *p* < .001) and health-threat (*Md* = 0.41, *SE* = 0.09, *p*= .003) categories. Images in the health-threat category had significantly higher pleasantness scores than images included in the general-threat category (*Md* = 0.42, *SE* = 0.07, *p* < .001).

An independent samples *t*-test was conducted comparing pain intensity scores (Table 4) between the images in the musculoskeletal pain and facial expressions of pain categories. There was a significant difference in the pain intensity scores of musculoskeletal-pain (*M* = 3.35, *SD* = 2.27) and facial expressions of pain (*M* = 5.15, *SD* = 2.13) categories; $t(245) = 6.520$, $p < .001$, $r = 0.38$. This result suggests images included in the facial expressions of pain category had significantly higher pain intensity ratings than those included in the musculoskeletal-pain category.

For peer review only - http://bmjopen.bmj.com/site/about/guidelines.xhtml

Table 4

Mean Arousal, Valence and Pain Intensity Ratings per Image Category

	Arousal		Valence		Pain Intensity	
Image Category	*M*	*SD*	*M*	*SD*	*M*	*SD*
Musculoskeletal-pain	2.85	1.45	4.20	0.57	3.35	2.09
Facial expressions of pain	3.81	1.92	3.50	0.66	5.15	2.02
General-threat	4.43	1.69	3.37	0.62		
Health-threat	3.48	1.53	3.79	0.71		
Musculoskeletal-pain neutral counterparts	1.82	0.68	5.11	0.22		
Facial expressions of pain neutral counterparts	1.74	0.69	5.00	0.14		
General-threat neutral counterparts	1.82	0.68	5.08	0.17		
Health-threat neutral counterparts	1.75	0.54	5.04	0.24		
Neutral/Neutral	1.57	0.49	5.10	0.15		

For peer review only

For peer review only - http://bmjopen.bmj.com/site/about/guidelines.xhtml

Comparison between Visual-Probe Sessions – Linguistic Stimuli

Differences in Arousal Scores between Threatening Word Categories across the Nine Linguistic Visual Probe Sessions

A 4 (word category; sensory-pain, affective-pain, general-threat, health-threat) x 9 (linguistic visual probe session; session 1, 3, 5, 7, 9, 11, 13, 15, 17) ANOVA was conducted to compare the arousal scores of the threatening word categories across the 9 linguistic visual probe task sessions. The results showed a significant main effect of session, $F(8, 120) = 8.22$, $p < .001$, $\eta_p^2 = .35$, and a significant word category x session interaction, $F(24, 360) = 3.05$, $p < .001$, $\eta_p^2 = .17$.

Post-hoc analyses across sessions. To explore the significant interaction, a series of one-way ANOVAs with 9 levels (Session: 1, 3, 5, 7, 9, 11, 13, 15, 17) were run for each threatening word category independently. These explored whether the arousal ratings of each threatening word category differed across the sessions. For the sensory-pain words, a main effect of session was found, $F(5,67) = 6.02$, $p < .001$, $\eta_p^2 = .29$. Pairwise comparisons revealed sensory words to be significantly more arousing in session 1 than session 9 ($Md = 0.92$, $SE = 0.22$, $p = .03$), and significantly more arousing in session 17 than session 9 ($Md = 0.75$, $SE = 0.17$, $p = .02$) and session 15 ($Md = 0.59$, $SE = 0.11$, $p = .003$).

For the affective-pain words, a main effect of session was found, $F(8,120) = 4.26$, $p < .001$, $\eta_p^2 = .22$. Pairwise comparisons revealed these words to be significantly more arousing in session 1 than in session 5 ($Md = 0.57$, $SE = 0.12$, $p = .01$) and session 13 ($Md = 1.04$, $SE = 0.26$, $p = .05$).

For the general-threat words, a main effect of session was found, $F(8, 120) = 4.91$, $p < .001$, $\eta_p^2 = .25$. Pairwise comparisons revealed these words to be significantly more arousing in session 1 than session 5 ($Md = 0.65$, $SE = 0.13$, $p = .01$), session 7 ($Md = 0.88$, $SE = 0.17$, $p = .004$) and session 15 ($Md = 0.84$, $SE = 0.19$, $p = .02$).

For peer review only - http://bmjopen.bmj.com/site/about/guidelines.xhtml

For the health-threat words, a main effect of session was found, $F(8,120) = 4.08$, $p < .001$, $\eta_p^2 = .21$. Pairwise comparisons revealed these words to be significantly more arousing in session 1 than session 11 ($Md = 1.05$, $SE = 0.18$, $p = .001$).

Post-hoc analyses within sessions. To further explore the significant interaction, a series of one-way ANOVAs with 4 levels (word category; sensory-pain, affective-pain, general-threat and health-threat) were conducted for each session independently. These explored whether the arousal ratings of each threatening word category differed within each linguistic visual-probe session. No significant main effects of word category were found for session 1, $F(3,45) = 1.16$, $p = .20$, $\eta_p^2 = .10$, session 5, $F(3,45) = 2.34$, $p = .86$, $\eta_p^2 = .14$, session 13, $F(3,45) = 0.70$, $p = .06$, $\eta_p^2 = .05$, and session 17, $F(3,45) = 2.27$, $p = .09$, $\eta_p^2 = .13$.

A significant main effect of word category was found in session 3, $F(3,45) = 4.31$, $p = .01$, $\eta_p^2 = .22$. Pairwise comparisons revealed no significant differences between the word categories of this session, however.

A significant main effect of word category was found in session 7, $F(3,45) = 3.75$, $p = .02$, $\eta_p^2 = .20$. Pairwise comparisons revealed no significant differences between the four threat-related categories.

A significant main effect of word category was found in session 9, $F(3,45) = 5.22$, $p = .004$, $\eta_p^2 = .26$. Pairwise comparisons revealed that sensory-pain words were significantly less arousing than affective-pain ($Md = 0.84$, $SE = 0.21$, $p = .01$), general-threat ($Md = 0.77$, $SE = 0.23$, $p = .03$) and health-threat categories ($Md = 0.92$, $SE = 0.30$, $p = .05$).

A significant main effect of word category was found in session 11, $F(3,45) = 4.45$, $p = .01$, $\eta_p^2 = .23$. Pairwise comparisons showed that the general-threat words were significantly more arousing than the health-threat words ($Md = 0.61$, $SE = 0.19$, $p = .04$).

A significant main effect of word category was found in session 15, $F(3,45) = 2.86$, $p = .05$, $\eta_p^2 = .16$. Pairwise comparisons revealed no significant differences between the four threat-related categories.

For peer review only - http://bmjopen.bmj.com/site/about/guidelines.xhtml

Differences in Valence Scores between Threatening Word Categories across the nine Linguistic Visual Probe Sessions

A 4 (word category; sensory-pain, affective-pain, general-threat and health-threat) x 9 (linguistic visual probe sessions; 1, 3, 5, 7, 9, 11, 13, 15, and 17) ANOVA was conducted to compare the pleasantness scores of the threatening categories across the 9 linguistic visual probe task sessions. The results showed significant main effects of word category, $F(3,45) = 15.86$, $p < .001$, $\eta_p^2 = .51$, and session, $F(8,120) = 11.90$, $p < .001$, $\eta_p^2 = .44$, and a significant word category x session interaction, $F(24,360) = 2.57$, $p < .001$, $\eta_p^2 = .15$.

Post-hoc analyses across sessions. To explore the significant interaction, a series of one-way ANOVAS with 9 levels (Session; 1, 3, 5, 7, 9, 11, 13, 15, 17) were run for each word category independently. These explored whether the pleasantness ratings for each word category differed across the sessions. For the sensory-pain words, a main effect of session was found, $F(5,75) = 6.88$, $p< .001$, $\eta_p^2 = .31$. Pairwise comparisons revealed sensory words to be significantly less pleasant in session 1 than session 3 (*Md* = 0.49, *SE* = 0.12, *p* =.04), session 7 (*Md* = 0.59, *SE* = 0.11, *p* = .003) and session 9 (*Md* = 0.73, *SE* = 0.09, *p* < .001). Sensory-pain words were also significantly less pleasant in session 17 than session 5 (*Md* = 0.26, *SE* = 0.05, *p* = .01) and session 9 (*Md* = 0.49, *SE* = 0.09, *p* = .003).

For the affective-pain words, a main effect of session was found, $F(8,120) = 4.86$, $p< .001$, $\eta p^2 = .25$. Pairwise comparisons revealed these words to be significantly less pleasant in session 1 than session 5 (*Md* = 0.51, *SE* = 0.11, *p* = .01), session 7 (*Md* = 0.47, *SE* = 0.09, *p* = .01), session 13 (*Md* = 0.59, *SE* = 0.11, *p* = .004) and session 15 (*Md* = 0.44, *SE* = 0.11, *p* = .04).

For the general-threat words, a main effect of session was found, $F(8,120) = 9.64$, $p< .001$, $\eta p^2 = .39$. Pairwise comparisons revealed these words to be significantly less pleasant in session 1 than session 5 (*Md* = 0.63, *SE* = 0.09, *p* < .001), session 7 (*Md* = 0.65, *SE* = 0.11, *p* = .001), session 9 (*Md* = 0.44, *SE* = 0.07, *p* < .001), session 13 (*Md* = 0.33, *SE* = 0.06, *p* = .001) and session 15 (*Md* = 0.65, *SE* = 0.12, *p* = .002). Significantly less pleasant words were also found in

For peer review only - http://bmjopen.bmj.com/site/about/guidelines.xhtml

session 3 than session 5 (*Md* = 0.37, *SE* = 0.09, *p* = .03) and session 7 (*Md* = 0.39, *SE* = 0.08, *p* = .01). Session 17 included significantly less pleasant words than session 7 (*Md* = 0.41, *SE* = 0.10, *p* = .03).

For the health-threat words, a main effect of session was found, $F(4,58)= 2.90$, $p= .03$, $\eta_p^2 = .16$. Pairwise comparisons revealed these words to be significantly less pleasant in session 1 than session 11 (*Md* = 0.57, *SE* = 0.14, *p* = .03).

Post-hoc analyses within sessions. To further explore the significant interaction, one-way ANOVAs with 4 levels (word category; sensory-pain, affective-pain, general-threat and health-threat) were conducted for each session independently. These explored whether pleasantness ratings for each threatening word category differed within each session. For session 1, a significant main effect was found, $F(3,45) = 4.84$, $p = .01$, $\eta_p^2 = .24$. Pairwise comparisons revealed that sensory-pain words were significantly more pleasant than affective words (*Md* = 0.63, *SE* = 0.16, *p* = .01).

For session 3, a significant main effect was found, $F(3,45) = 12.71$, $p < .001$, $\eta_p^2 = .46$. Pairwise comparisons showed that sensory-pain words were significantly more pleasant than affective-pain (*Md* = 0.73, *SE* = .15, *p* < .001), general-threat (*Md* = 0.79, *SE* = 0.13, *p* < .001) and health-threat words (*Md* = 0.56, *SE* = 0.15, *p* = .01).

For session 5, a significant main effect was found, $F(3,45) = 6.45$, $p <.001$, $\eta_p^2 = .30$. Pairwise comparisons revealed that sensory-pain words were significantly more pleasant than the affective-pain words (*Md* = 0.62, *SE* = 0.15, *p* = .01).

For session 7, a significant main effect was found, $F(3,45) = 10.19$, $p < .001$, $\eta_p^2 = .41$. Pairwise comparisons revealed that sensory-pain words were significantly more pleasant than the affective-pain words (*Md* = 0.17, *SE* = 0.13, *p* < .001), the general-threat words (*Md* = 0.51, *SE* = 0.09, *p* < .001) and the health-threat words (*Md* = 0.55, *SE* = 0.16, *p* = .02).

For session 9, a significant main effect was found, $F(2,31) = 13.67$, $p < .001$, $\eta_p^2 = .48$. Pairwise comparisons revealed that the sensory-pain words were significantly more pleasant than

For peer review only - http://bmjopen.bmj.com/site/about/guidelines.xhtml

the affective-pain words (*Md* = 0.98, *SE*= 0.16, $p < .001$), the general-threat words (*Md* = 0.87, *SE* = 0.15, $p < .001$) and the health-threat words (*Md* = 0.92, *SE* = 0.21, $p < .001$).

For session 11, a significant main effect was found, $F(3,45) = 11.63$, $p < .001$, $\eta_p^2 = .44$. Pairwise comparisons revealed that the sensory-pain words were significantly more pleasant than the affective-pain (*Md* = 0.69, *SE* = 0.13, $p < .001$) and the general-threat words (*Md* = 0.70, *SE* = 0.17, $p < .001$). Also, the affective-pain words were significantly less pleasant than the health-threat words (*Md* = 9.39, *SE* = 0.13, $p = .04$).

For session 13, a significant main effect was found, $F(3,45) = 4.43$, $p = .01$, $\eta_p^2 = .23$. Pairwise comparisons revealed that the sensory-pain words were significantly more pleasant than the general-threat words (*Md* = 0.56, *SE* = 0.12, $p < .001$).

For session 15, a significant main effect was found, $F(3,45) = 8.40$, $p < .001$, $\eta_p^2 = .36$. Pairwise comparisons showed that the sensory-pain words were significantly more pleasant than the words included in the affective (*Md* = 0.61, *SE* = 0.12, $p < .001$) and the health-threat words (*Md* = 0.71, *SE* = 0.19, $p = .01$).

For session 17, a significant main effect was found, $F(3,45) = 6.78$, $p<.001$, $\eta_p^2 = 0.36$. $_p^2$ = 0.31. Pairwise comparisons revealed that the sensory-pain words were significantly more pleasant than affective-pain (*Md* = 0.56, *SE* = 0.15, $p = .01$) and the general-threat words (*Md* = 0.57, *SE* = 0.15, $p = .01$).

Comparison between Visual-Probe Sessions – Pictorial Stimuli

Differences in Arousal Scores between Threatening Image Categories across the eight Pictorial Visual Probe Sessions

A 4 (image category; musculoskeletal-pain, facial expressions of pain, general-threat, health-threat) x 8 (pictorial visual probe sessions; 2, 4, 6, 8, 10, 12, 14, 16) ANOVA was conducted to compare the arousal scores of the threatening image categories across the 8 pictorial visual probe task sessions. The results showed a significant main effect of image category, $F(3,45) = 33.52$, *p*

For peer review only - http://bmjopen.bmj.com/site/about/guidelines.xhtml

$< .001$, $\eta_p^2 = .69$, and a significant image category x session interaction, $F(7,98) = 9.84$, $p < .001$, $\eta_p^2 = .40$.

Post-hoc analyses within image categories. To explore the significant interaction, a series of one-way ANOVAs with 8 levels (Session; 2, 4, 6, 8, 10, 12, 14, 16) were run for each threatening image category independently. These explored whether the arousal ratings of each threat-related image category differed across the sessions. For the images in the musculoskeletal-pain category, no main effect of session was found, $F(2,31) = 2.49$, $p = .10$, $\eta_p^2 = .14$. For the images in the facial expressions of pain category, no main effect of session was found, $F(3,46) = 0.77$, $p = .50$, $\eta_p^2 = .05$.

For the images in the general-threat category, a main effect of session was found, $F(7, 105) = 7.40$, $p < .001$, $\eta_p^2 = .33$. Pairwise comparisons revealed these images to be significantly less arousing in session 4 than session 6 (*Md* = 0.70, *SE* = 0.17, *p* = .023), session 12 (*Md* = 0.73, *SE* = 0.19, *p* = .04) and session 16 (*Md* = 0.82, *SE* = 0.21, *p* = .04). These images were also found to be significantly less arousing in session 14 than session 6 (*Md* = 0.93, *SE* = 0.19, *p* = .006), session 12 (*Md* = 0.96, *SE* = 0.17, *p* = .001) and session 16 (*Md* = 1,05, *SE* = 0.17, *p* = .001).

For the images in the health-threat category, a main effect of session was found, $F(7, 105) = 13.10$, $p < .001$, $\eta_p^2 = .47$. Pairwise comparisons revealed these images to be significantly more arousing in session 10 than session 8 (*Md* = 1.15, *SE* = 0.20, *p* = .001) and session 12 (*Md* = 0.88, *SE* = 0.20, *p* = .02). Health-threat images were also found to be significantly less arousing in session 16 than session 2 (*Md* = 0.97, *SE* = 0.17, *p* = .001), session 4 (*Md* = 1.34, *SE* = 0.26, *p* = .004), session 6 (*Md* = 1.32, *SE* = 0.29, *p* = .009), session 10 (*Md* = 1.91, *SE* = 0.28, *p*< .001), session 12 (*Md* = 1.03, *SE* = 0.26, *p* = .04) and session 14 (*Md* = 1.45, *SE* = 0.9, *p*< .001).

Post-hoc analyses within sessions. To further explore the significant interaction, a series of one-way ANOVAs with 4 levels (image category; musculoskeletal-pain, facial expressions of pain, general-threat and health-threat) were conducted for each session independently. These

For peer review only - http://bmjopen.bmj.com/site/about/guidelines.xhtml

explored whether the arousal ratings of each threatening image category differed within each pictorial visual-probe session.

A significant main effect of image category was found in session 2, $F(3,45) = 23.27$, $p < .001$, $\eta_p^2 = .61$. Pairwise comparisons revealed that the images in the facial expressions of pain category were more arousing than those included in the musculoskeletal-pain category (Md = 0.98, SE = 0.27, $p = .014$). The images in the general-threat category were also found to be significantly more arousing than those included in the musculoskeletal-pain (Md = 2.05, SE = 0.32, $p < .001$), facial expressions of pain (Md = 1.07, SE = 0.19, $p < .001$) and health-threat categories (Md = 1.34, SE = 0.24, $p < .001$).

A significant main effect of image category was found in session 4, $F(3,45) = 8.27$, $p < .001$, $\eta_p^2 = .36$. Pairwise comparisons revealed that the images in the musculoskeletal-pain category were significantly less arousing than the images in the facial expressions of pain (Md = 0.76, SE = 0.16, $p = .001$), health-threat (Md = 1.10, SE = 0.23, $p = .002$) and general-threat categories (Md = 0.70, SE = 0.22, $p = .04$).

A significant main effect of image category was found in session 6, $F(3,45) = 26.77$, $p < .001$, $\eta_p^2 = .64$. Pairwise comparisons revealed that the images in the musculoskeletal-pain category were significantly less arousing than the images included in the facial expressions of pain (Md = 1.17, SE = 0.25, $p = .002$), general-threat (Md = 2.06, SE = 0.23, $p < .001$) and health-threat categories (Md = 0.97, SE = 0.25, $p = .009$). The images in the general-threat category were significantly more arousing than those in the facial expressions of pain (Md = 0.89, SE = 0.21, $p = .003$) and health-threat categories (Md = 1.09, SE = 0.21, $p = .001$).

A significant main effect of image category was also found in session 8, $F(2,32) = 10.56$, $p < .001$, $\eta_p^2 = .41$. Pairwise comparisons revealed that the images in the musculoskeletal-pain category were significantly less arousing than those included in the facial expressions of pain (Md = 0.89, SE = 0.22, $p = .006$) and general-threat categories (Md = 1.13, SE = 0.25, $p = .003$).

For peer review only - http://bmjopen.bmj.com/site/about/guidelines.xhtml

The images in general-threat categories were significantly more arousing that those included in the health-threat category (*Md* = 1.00, *SE* = 0.20, *p* = .001).

A significant main effect of image category was found in session 10, $F(3,45) = 17.37$, $p < .001$, $\eta_p^2 = .54$. Pairwise comparisons revealed that the images in the musculoskeletal-pain category were significantly less arousing than those included in the general-threat (*Md* = 0.92, *SE* = 0.17, *p*< .001) and health-threat categories (*Md* = 1.23, *SE* = 0.20, *p* < .001). The images in the facial expressions of pain category were also significantly less arousing than those included in the health-threat category (*Md* = 0.71, *SE* = 0.18, *p* = .009).

A significant main effect of image category was found in session 12, $F(3,45) = 18.40$, $p < .001$, $\eta_p^2 = .55$. Pairwise comparisons revealed that the images in the musculoskeletal-pain category were significantly less arousing that those included in the facial expressions of pain (*Md* = 1.61, *SE* = 0.18, *p* = .022) and general-threat categories (*Md* = 1.64, *SE* = 0.19, *p* < .001). The images included in the general-threat category were significantly more arousing than the images included in the facial expressions of pain (*Md* = 1.03, *SE* = 0.24, *p* = .001) and health-threat categories (*Md* = 1.03, *SE* = 0.26, *p* < .001).

A significant main effect of image category was found in session 14, $F(3,45) = 14.72$, $p < .001$, $\eta_p^2 = .50$. Pairwise comparisons revealed than the images in the musculoskeletal-pain category were significantly less arousing than those included in facial expressions of pain (*Md* = 1.39, *SE* = 0.30, *p* = .002), general-threat (*Md* = 1.41, *SE* = 0.25, *p* < .001) and health-threat categories (*Md* = 1.35, *SE* = 0.20, *p* < .001).

A significant main effect of image category was found in session 16, $F(2,28) = 38.74$, $p < .001$, $\eta_p^2 = .72$. Pairwise comparisons revealed that the images in the musculoskeletal-pain category were significantly less arousing than the images included in the facial expressions of pain (*Md* = 1.38, *SE* = 0.29, *p* = .001) and general-threat categories (*Md* = 2.37, *SE* = 0.27, *p* = .002). Images included in the facial expression of pain category were also found to be significantly less arousing than those included in the general-threat category (*Md* = 0.98, *SE* =

For peer review only - http://bmjopen.bmj.com/site/about/guidelines.xhtml

0.21, p = .002). Images in the health-threat category were significantly less arousing than the images included in the facial expression of pain (Md = 1.57, SE = 0.36, p = .004) and general-threat categories (Md = 2.56, SE = 0.29, p < .001).

Differences in Valence Scores between Threatening Pictorial Categories across the eight Pictorial Visual Probe Sessions

A 4 (image category; musculoskeletal-pain, facial expressions of pain, general-threat and health-threat) x 8 (pictorial visual probe sessions; 2, 4, 6, 8, 10, 12, 14, 16) ANOVA was conducted to compare the pleasantness scores of the threatening categories across the 8 pictorial visual probe task sessions. The results showed a significant main effect of image category, $F(3,45) = 41.09$, $p < .001$, $\eta_p^2 = .73$, and a significant image category x session interaction, $F(7,102) = 9.33$, $p < .001$, $\eta_p^2 = .38$.

Post-hoc analyses within image categories. To explore the significant interaction, a series of one-way ANOVAS with 8 levels (Session; 2, 4, 6, 8, 10, 12, 14, 16) were run for each image category independently. These explored whether the pleasantness ratings for each image category differed across the sessions.

For the images included in the musculoskeletal-pain category, a main effect of session was found, $F(2,32) = 3.70$, $p = .033$, $\eta_p^2 = .20$. Pairwise comparisons revealed these images to be significantly more pleasant in session 2 than session 4 (Md = 0.54, SE = 0.11, p = .007).

For the images included in the facial expressions of pain category, a main effect of session was found, $F(7,105) = 3.44$, $p = .002$, $\eta_p^2 = .19$. Pairwise comparisons revealed these images to be significantly more pleasant in session 10 than session 6 (Md = 0.51, SE = 0.13, p = .03) and session 16 (Md = 0.43, SE = 0.09, p = .005).

For the images included in the general-threat category, a main effect of session was found, $F(7,105) = 6.84$, $p < .001$, $\eta_p^2 = .31$. Pairwise comparisons revealed these images to be significantly more pleasant in session 10 than session 6 (Md = 0.61, SE = 0.13, p = .009), session 12 (Md = 0.56, SE = 0.14, p = .05) and session 16 (Md = 0.68, SE = 0.14, p = .007). General-

For peer review only - http://bmjopen.bmj.com/site/about/guidelines.xhtml

threat images were significantly more pleasant in session 14 than session 6 (*Md* = 0.60, *SE* = 0.14, *p* = .019), session 12 (*Md* = 0.55, *SE* = 0.12, *p* = .013) and session 16 (*Md* = 0.67, *SE* = 0.11, *p* = .001). Images in this category were also significantly more pleasant in session 4 than session 6 (*Md* = 0.45, *SE* = 0.11, *p* = .04) and session 16 (*Md* = 0.52, *SE* = 0.11, *p* = .007).

For the images included in the health-threat category, a main effect of session was found, $F(7,105) = 9.87$, $p < .001$, $\eta_p^2 = .40$. Pairwise comparisons revealed these images to be significantly more pleasant in session 8 than session 10 (*Md* = 0.73, *SE* = 0.10, *p* = .005). Images in this category were significantly more pleasant in session 16 than session 2 (*Md* = 0.54, *SE* = 0.11, *p* = .004), session 4 (*Md* = 0.62, *SE* = 0.12, *p* = .003), session 6 (*Md* = 0.52, *SE* = 0.12, *p* = .02), session 10 (*Md* = 1.00, *SE* = 0.10, $p < .001$), session 12 (*Md* = 0.56, *SE* = 0.12, *p* = .008) and session 14 (*Md* = 0.80, *SE* = 0.10, $p < .001$).

Post-hoc analyses within sessions. To further explore the significant interaction, one-way ANOVAs with 4 levels (image category; musculoskeletal-pain, facial expressions of pain, general-threat and health-threat) were conducted for each session independently. These explored whether pleasantness ratings for each image category differed within each session.

For session 2, a significant main effect was found, $F(3,45) = 13.36$, $p < .001$, $\eta_p^2 = .47$. Pairwise comparisons revealed that images in the musculoskeletal-pain category were significantly more pleasant than images included in the facial expressions of pain (*Md* = 1.00, *SE* = 0.18, $p < .001$), general-threat (*Md* = 0.98, *SE* = 0.20, *p* = .001) and health-threat categories (*Md* = 0.72, *SE* = 0.21, *p* = .024).

For session 4, a significant main effect was found, $F(3,45) = 7.09$, $p = .001$, $\eta_p^2 = .32$. Pairwise comparisons showed that the images in the musculoskeletal-pain category were significantly more pleasant than the facial expressions of pain (*Md* = 0.52, *SE* = 0.10, *p* = .001) and general-threat categories (*Md* = 0.48, *SE* = 0.12, *p* = .005).

For session 6, a significant main effect was found, $F(3,45) = 32.12$, $p <.001$, $\eta_p^2 = .68$. Pairwise comparisons revealed that images in the musculoskeletal-pain category were

For peer review only - http://bmjopen.bmj.com/site/about/guidelines.xhtml

significantly more pleasant than the images included in the facial expressions of pain (*Md* = 1.05, *SE* = 0.14, $p < .001$) and general-threat categories (*Md* = 1.27, *SE* = 0.13, $p < .001$). The images in the health-threat category were also significantly more pleasant than the images included in the facial expressions of pain (*Md* = 0.56, *SE* = 0.14, $p = .01$) and general-threat categories (*Md* = 0.77, *SE* = 0.13, $p < .001$).

For session 8, a significant main effect was found, $F(3,45) = 11.73$, $p < .001$, $\eta_p^2 = .44$. Pairwise comparisons revealed that images in the musculoskeletal-pain category were significantly more pleasant than those included in the facial expressions of pain (*Md* = 0.62, *SE* = 0.07, $p < .001$) and general-threat categories (*Md* = 0.57, *SE* = 0.14, $p = .004$). The images included in the health-threat category were also significantly more pleasant than those included in the facial expressions of pain (*Md* = 0.54, *SE* = 0.15, $p = .019$) and general-threat categories (*Md* = 0.49, *SE* = 0.14, $p = .016$).

For session 10, a significant main effect was found, $F(3,45) = 12.59$, $p < .001$, $\eta_p^2 = .46$. Pairwise comparisons revealed that images in the health-threat category were significantly less pleasant than those included in the musculoskeletal-pain (*Md* = 0.74, *SE* = 0.12, $p < .001$) and facial expressions of pain categories (*Md* = 0.44, *SE* = 0.12, $p = .017$).

For session 12, a significant main effect was found, $F(3,45) = 14.90$, $p < .001$, $\eta_p^2 = .50$. Pairwise comparisons revealed that images in the musculoskeletal-pain category were significantly more pleasant than images included in the facial expressions of pain (*Md* = 0.45, *SE* = 0.08, $p < .001$) and general-threat categories (*Md* = 0.88, *SE* = 0.16, $p < .001$). Images in the health-threat category were also significantly more pleasant than images included in the general health category (*Md* = 0.68, *SE* = 0.16, $p = .004$).

For session 14, a significant main effect was found, $F(3,45) = 13.30$, $p < .001$, $\eta_p^2 = .47$. Pairwise comparisons revealed that images in the musculoskeletal-pain category were significantly more pleasant than images included in the facial expressions of pain (*Md* = 0.71, *SE*

For peer review only

= 0.16, p = .003), general-threat (Md = 0.71, SE = 0.15, p = .001) and health-threat categories (Md = 0.82, SE = 0.12, p < .001).

For session 16, a significant main effect was found, $F(3,45) = 83.05$, p <.001, $\eta_p^2 = .85$. Pairwise comparisons revealed that images in the facial expressions of pain category were significantly less pleasant than musculoskeletal-pain (Md = 0.97, SE = 0.10, p < .001) and health-threat categories (Md = 0.99, SE = 0.12, p < .001). Images in the general-threat category were also significantly less pleasant than those included in the musculoskeletal-pain (Md = 1.34, SE = 0.11, p < .001), facial expressions of pain (Md = 0.37, SE = 0.09, p = .007) and health-threat categories (Md = 1.36, SE = 0.09, p < .001).

Differences in Pain Intensity Scores of Threatening Pain Categories across the eight Pictorial Visual Probe Sessions

A 2 (image category; musculoskeletal-pain, facial expressions of pain) x 8 (pictorial visual probe sessions; session 2, 4, 6, 8, 10, 12, 14, 16) ANOVA was conducted to compare the pain intensity scores of the threatening pain categories across the 8 pictorial visual probe task sessions. The results showed significant main effects of image category, $F(1,15) = 45.40$, p < .001, $\eta_p^2 = .75$, and session, $F(2,29) = 3.81$, p = .04, $\eta_p^2 = .20$, and a significant image category x session interaction, $F(3,44) = 7.44$, p < .001, $\eta_p^2 = .33$.

Post-hoc analyses within image categories. To explore the significant interaction, two one-way ANOVAs with 8 levels (Session; 2, 4, 6, 8, 10, 12, 14, 16) were run for each pain-related image category independently. These explored whether the pain intensity ratings of each pain-related image category differed across the sessions. For the musculoskeletal-pain category, a main effect of session was found, $F(2,25) = 4.70$, p = .023, $\eta p^2 = .24$. Pairwise comparisons revealed that the images included in session 14 had significantly less pain intensity scores than the images included in session 8 (Md = 1.06, SE = 0.24, p = .014), session 10 (Md = 0.95, SE = 0.19, p = .004) and session 12 (Md = 1.19, SE = 0.15, p < .001). Images in session 4 had significantly higher pain intensity scores than the images included in session 6 (Md = 0.82, SE =

For peer review only - http://bmjopen.bmj.com/site/about/guidelines.xhtml

0.21, p = .05). The images included in session 12 had also significantly higher pain intensity scores than the images included in session 16 (*Md* = 1.03, *SE* = 0.17, p = .001).

For the facial expression of pain category, a main effect of session was found, $F(3,39)$ = 4.65, p = .01, ηp^2 = .24. Pairwise comparisons revealed that the images included in session 10 had significantly less pain intensity scores that the images included in session 1 (*Md* = 1.07, *SE* = 0.25, p = .02) and session 6 (*Md* = 1.38, *SE* = 0.31, p = .013).

Differences in low-level features of pictorial categories across the eight visual probe sessions

A series of one-way ANOVAs were conducted independently to compare the scores of complexity, red, green and blue saturation, luminance and RMS contrast level of the threat related categories (i.e., musculoskeletal-pain, facial expression of pain, general-threat and health-threat) across the 8 pictorial visual probe task sessions (i.e., Session 2, 4, 6, 8, 10, 12, 14, 16).

For complexity, the analysis revealed a significant main effect of image category, $F(3, 224)$ = 5.15, p = .002, η_p^2 = .06. There was no significant main effect of session, $F(7, 224)$ = 1.22, p = .30, and no significant image category x session interaction, $F(21, 224)$ = 0.82, p =.70.

For red saturation, the analysis revealed a significant main effect of image category, $F(3, 224)$ = 5.05, p = .002, η_p^2 = .06. There was no significant main effect of session , $F(7,224)$ = 1.03, p = .41, and no significant image category x session interaction, $F(21,224)$ = 1.196, p =.26.

For green saturation, the analysis revealed a significant main effect of image category, $F(3, 224)$ = 4.08, p = .008, η_p^2 = .08. There was no significant main effect of session, $F(7, 224)$ = 1.65, p = .12, and no and no significant image category x session interaction, $F(21,224)$ = 1.41, p =.12.

For blue saturation, the analysis revealed a significant main effect of image category, $F(3, 224)$ = 5.288, p = .002, η_p^2 = 0.07, and a significant image category x session interaction, $F(21, 224)$ = 1.672, p = .04, η_p^2 = 0.14. There was no significant main effect of session, $F(7, 224)$ = 1.750, p = .10.

For peer review only - http://bmjopen.bmj.com/site/about/guidelines.xhtml

For luminance, the analysis revealed a significant main effect of image category, $F(3, 224) = 4.548$, $p = .004$, $\eta_p^2 = 0.06$. The analysis revealed no significant main effect of session, $F(7,224) = 1.504$, $p = .17$, and no significant image category x session interaction, $F(21, 224) = 1.361$, $p = .14$.

For RMS contrast level, the analysis revealed a significant main effect of image category, $F(3, 224) = 6.84$, $p < .001$, $\eta_p^2 = .08$, and a significant image category x session interaction, $F(21,224) = 1.98$, $p = .008$. There was no significant main effect of session, $F(7,224) = 1.68$, $p = .12$,

Post-hoc analyses within image categories. To explore the significant interaction for blue saturation, a series of one-way ANOVAS with 8 levels (Session; 2, 4, 6, 8, 10, 12, 14, 16) were run for each image category independently. These explored whether the blue saturation scores for each image category differed across the sessions. No significant main effect of session was found for the musculoskeletal-pain category, $F(7,56) = 0.82$, $p = .57$, or the health-threat category, $F(7,56) = 1.59$, $p = .16$.

For the facial expressions of pain category, a significant interaction for session number was found, $F(7,56) = 2.94$, $p = .01$, $\eta_p^2 = .27$. Pairwise comparisons did not reveal any significant differences between the factors.

For the general-threat category, a significant main effect of session number, $F(7,56) = 2.20$, $p = .05$, $\eta_p^2 = .22$. Pairwise comparisons did not reveal any significant differences between the factors.

To explore the significant interaction for RMS contrast level, a series of one-way ANOVAS with 8 levels (Session; 2, 4, 6, 8, 10, 12, 14, 16) were run for each image category independently. These explored whether the RMS contrast level scores for each image category differed across the sessions. No significant main effect of session was found for the musculoskeletal-pain category, $F(7,56) = 0.59$, $p = .76$, or the health-threat category, $F(7,56) = 0.16$, $p = .99$

For peer review only - http://bmjopen.bmj.com/site/about/guidelines.xhtml

For the facial expressions of pain category, a significant interaction for session number was found, $F(7,56) = 5.127$, $p < .001$, $\eta_p^2 = 0.39$. Pairwise comparisons revealed that the images in session 6 had significantly less RMS contrast than those included in session 4 (*Md* = 36.02, *SE* = 7.76, *p* = .001), session 10 (*Md* = 30.81, *SE* = 7.76, *p* = .006), session 14 (*Md* = 25.96, *SE* = 7.76, *p* = .04) and session 16 (*Md* = 37.09, *SE* = 776, *p*< .001).

For the general-threat category, a significant interaction for session number was found, $F(7,56) = 3.252$, $p = .006$, $\eta_p^2 = 0.29$. Pairwise comparisons did not reveal any significant interaction between the sessions.

Post-hoc analyses within sessions. To further explore the significant interaction for blue saturation, eight one-way ANOVAs with 4 levels (image category; musculoskeletal-pain, facial expressions of pain, general-threat and health-threat) were conducted for each session independently. These explored whether blue saturation scores between the five image categories differed within each session (i.e., session 2, 4, 6, 8, 10, 12, 14, 16).

No significant main effects were found for session 2, $F(3,28) = 1.49$, $p = .24$, session 4, $F(3,28) = 0.18$, $p = .91$, session 6, $F(3,28) = 1.37$, $p = .27$, session 8, $F(3,28) = 1.127$, $p = .36$, and session 14, $F(3,28) = 0.508$, $p = .68$.

For session 10, there was a significant main effect of image category, $F(3,28) = 5.56$, $p = .004$, $\eta_p^2 = .37$. Pairwise comparisons revealed that the images included in the facial expression of pain category had significantly higher blue saturation than the images included in the general-threat category (*Md* = 70.64, *SE* = 18.03, *p* = .003).

For session 12, there was a significant main effect of image category, $F(3,28) = 3.55$, $p = .027$, $\eta_p^2 = .28$. Pairwise comparisons revealed that the images included in the musculoskeletal-pain category had significantly higher blue saturation than the images included in the general-threat category (*Md* = 62.46, *SE* = 21.76, *p* = .046).

For session 16, there was a significant main effect of image category, $F(3,28) = 6.99$, $p = .001$, $\eta_p^2 = .43$. Pairwise comparisons revealed that the images included in the health-threat

For peer review only - http://bmjopen.bmj.com/site/about/guidelines.xhtml

categories had significantly higher blue saturation than the images included in the musculoskeletal-pain (*Md* = 74.57, *SE* = 20.09, p = .005) and general-threat categories (*Md* = 83.89, *SE* = 20.09, p = .002)

To further explore the significant interaction for RMS contrast level, eight one-way ANOVAs with 4 levels (image category; musculoskeletal-pain, facial expressions of pain, general-threat and health-threat) were conducted for each session independently. These explored whether the scores of RMS contrast level between the five image categories differed within each session (i.e., session 2, 4, 6, 8, 10, 12, 14, 16).

No significant main effects were found for session 2, $F(3,28) = 0.720$, p = .55, session 8, $F(3,28) = 1.56$, p = .22, session 12, $F(3,28) = 0.62$, p = .61, and session 14, $F(3,28) = 1.76$, p = .18.

For session 4, there was a significant main effect of image category, $F(3,28) = 5.69$, p = .004, $\eta_p^2 = .38$. Pairwise comparisons revealed that the images included in the facial expression of pain category had significantly higher RMS contrast than the images included in the health-threat category (*Md* = 33.44, *SE* = 8.27, p = .002).

For session 6, there was a significant main effect of image category, $F(3,28) = 4.00$, p = .02, $\eta_p^2 = .30$. Pairwise comparisons revealed that the images included in the musculoskeletal-pain category had significantly higher RMS contrast than those included in the facial expressions of pain category (*Md* = 17.69, *SE* = 5.95, p = .04).

For session 10, there was a significant main effect of image category, $F(3,28) = 6.02$, p = .003, $\eta_p^2 = .39$. Pairwise comparisons revealed that the images included in the facial expressions of pain category had significantly higher RMS contrast than those included in the general-threat (*Md* = 30.37, *SE* = 7.35, p = .002) and health-threat categories (*Md* = 21.42, *SE* = 7.35, p = .04).

For session 16, there was a significant main effect of image category, $F(3,28) = 3.18$, p = .04, $\eta_p^2 = .25$. Pairwise comparisons revealed that the images included in the facial expressions

For peer review only - http://bmjopen.bmj.com/site/about/guidelines.xhtml

of pain category had significantly higher RMS contrast level than those included in the health-threat category (*Md* = 29.32, *SE* = 10.24, *p* = .05).

For peer review only

References

1. Kučera H, Francis N. *Computational analysis of present-day American English*. Providence: Brown University Press, 1967.

2. AP Y. The Nature of Recollection and Familiarity: A Review of 30 Years of Research. Journal of Memory and Language 2002;**46**(3):441-517 doi: 10.1006/jmla.2002.2864.

3. Balota DA, Chumbley JI. Are lexical decisions a good measure of lexical access? The role of word frequency in the neglected decision stage. Journal of Experimental Psychology: Human perception and performance 1984;**10**(3):340-57.

4. Sharpe L, Ianiello, M., Dear, B. F, Nicholson Perry, K., Refshauge, K., Nicholas, M. K. Is there a potential role for attention bias modification in pain patients? Results of 2 randomised, controlled trials. Pain 2012;**153**(3):722-31.

5. Schoth D, Liossi C. Specificity and time-course of attentional bias in chronic headache: a visual-probe investigation. Clinical Journal of Pain 2013;**29**(7):583-90 doi: 10.1097/AJP.0b013e31826b4849.

6. Carleton R, Richter A, Asmundson G. Attention Modification in Persons with Fibromyalgia: A Double Blind, Randomized Clinical Trial. Cognitive Behaviour Therapy 2011;**40**(4):279-90.

7. Macleod C. Cognitive bias modification procedures in the management of mental disorders. Curr Opin Psychiatry 2012;**25**(2):114-20 doi: 10.1097/YCO.0b013e32834fda4a.

8. MacLeod C, Mathews A, Tata P. Attentional bias in emotional disorders. Journal of Abnormal Psychology 1986;**95**(1):15-20 doi: 10.1037/0021-843X.95.1.15.

9. Melzack R. The McGill Pain Questionnaire: major properties and scoring methods. Pain 1975;**1**(3):277-99.

10. Wilson MD. The MRC Psycholinguistic Database: Machine Readable Dictionary, Version 2. Behavioural Research Methods, Instruments and Computers 1988;**20**(1):6-11.

For peer review only

11. MRC Psycholinguistic Database: Machine Usable Dictionary. Version 2.00.: MRC Psycholinguistic Database, 1997.

12. Lane RD, Chua PM, Dolan RJ. Common effects of emotional valence, arousal and attention on neural activation during visual processing of pictures. Neuropsychologia 1999;**37**(9):989-97 doi: 10.1016/S0028-3932(99)00017-2.

13. Niedenthal PM, Kitayama S. *The heart's eye: Emotional influences in perception and attention*. London: Academic Press, 2013.

14. Bradley MM, Lang PJ. Measuring Emotion: The Self-Assessment Manikin and the Semantic Differential. J Behav Therap & Exp Psychiat 1994;**25**(1):49-59.

15. Liossi C, Schoth D. Attention toward interpersonal stimuli in individuals with and without chronic daily headache. Journal of Neurology and Neurosurgery 2016;**3**(3):1-7.

16. Schoth DE, Liossi C. Attentional bias toward pictorial representations of pain in individuals with chronic headache. Clin J Pain 2010;**26**(3):244-50 doi: 10.1097/AJP.0b013e3181bed0f9.

17. Craig KD. The facial expression of pain. Better than a thousand words? APS Journal 1992;**1**(3):153-62.

18. Prkachin KM, Solomon PE. The structure, reliability and validity of pain expression: evidence from patients with shoulder pain. Pain 2008;**139**(2):267-74 doi: 10.1016/j.pain.2008.04.010.

19. Schoth DE, Georgallis T, Liossi C. Attentional bias modification in people with chronic pain: a proof of concept study. Cognitive Behaviour Therapy 2013;**42**(3):233-43.

20. Bradley MM, Lang PJ. The International Affective Picture System (IAPS) in the study of emotion and attention. In: Allen CaJJB, ed. Handbook of Emotion Elicitation and Assessment: Oxford University Press, 2007.

21. Leeuw M, Goossens ME, van Breukelen GJ, et al. Measuring perceived harmfulness of physical activities in patients with chronic low back pain: the Photograph Series of Daily

For peer review only - http://bmjopen.bmj.com/site/about/guidelines.xhtml

Activities--short electronic version. J Pain 2007;**8**(11):840-9 doi: 10.1016/j.jpain.2007.05.013.

22. Tottenham N, Tanaka JW, Leon AC, et al. The NimStim set of facial expressions: judgments from untrained research participants. Psychiatry Res 2009;**168**(3):242-9 doi: 10.1016/j.psychres.2008.05.006.

23. Nummenmaa L, Hyönä J, Calvo MG, & Nummenmaa, L. Eye movement assessment of selective attentional capture by emotional pictures. Emotion 2006;**6**(2):257-68 doi: 10.1037/1528-3542.6.2.257.

24. Borji A, Sihite DN, Itti L. What stands out in a scene? A study of human explicit saliency judgment. Vision research 2013;**91**:62-77 doi: 10.1016/j.visres.2013.07.016.

25. Henderson JM. Human gaze control during real-world scene perception. Trends in Cognitive Sciences 2003;**7**(11):498-504 doi: 0.1016/j.tics.2003.09.006.

26. Torralba A, Oliva, A., Castelhano, M. S., & Henderson, J. M. Contextual guidance of eye movements and attention in real-world scenes: The role of global features in object search. Psychological Review 2006;**113**(4):766-86 doi: 10.1037/0033-295X.113.4.766.

27. Buodo G, Sarlo M, Palomba D. Attentional resources measured by reaction times highlight differences within pleasant and unpleasant, high arousing stimuli. Motivation and Emotion 2002;**26**(2):123-38 doi: 10.1023/A:1019886501965.

For peer review only

Supplementary Material 2 – Outcome Measures

A detailed description of each outcome measure used in this study is provided here. While the questionnaires will be administered online as part of this study, the psychometric properties reported are for paper and pencil versions of the questionnaires.

The BPI-SF[1] is a commonly used measure of pain intensity and pain interference. Participants indicate their worst, least, average and current pain on an 11-point numerical scale ranging from *no pain at all* to *pain as bad as you can imagine*. Additional questions include how much relief pain medication has provided, along with pain interference on general activity, mood, walking ability, normal work, relations with others, sleep, and enjoyment of life. A body diagram is also provided for participants to indicate where they feel pain. The psychometric properties of the BPI are well supported in patients with cancer pain and other pain populations[1] [2], including internal consistency of pain intensity and interference items as assessed in patients with chronic nonmalignant pain (Cronbach's alpha .85 and .88 respectively).[3]

The HADS[4] consists of 14 items measuring the severity of anxiety and depression symptoms (seven items each) over the past week. Each item is measured on a four point scale, with possible scores ranging from 0 – 21 for each subscale. Higher scores indicate higher levels of anxiety and depression. A review of studies using the HADS (the majority of which were conducted with patients with cancer or other physical health conditions) has supported the internal consistency of anxiety (.68 - .93, mean .82) and depression (.67 - .90, mean .82) subscales.[5]

The STAI[6] is a 40-item measure of state and trait anxiety (20 items each). Each item is measuerd on a four point scale , with possible scores for both state and trait levels ranging between 20 and 80. Higher scores represent more intense or more frequent feelings of anxiety. A review of 45 articles reporting psychometric properties from clinical and non-clinical samples of state and trait subscales found high levels of internal consistency (.91 and .89 respectively) and test-retest reliability (.70 and .88 respectively).[7]

For peer review only - http://bmjopen.bmj.com/site/about/guidelines.xhtml

The FOP-III[8] is a 30 item measure of pain-related fear. On a 5-point scale, respondents are asked to rate how fearful they are of pain associated with specific situations and events (e.g., being in an automobile accident). Possible scores range between 30 and 150, with higher scores representing a more intense fear of pain. In addition to a total score, the measure also provides subscales of minor, severe, and medical pain-related fear. The FOP-III is commonly used in clinical and non-clinical populations, and possesses high internal consistency as indexed in a non-clinical sample (total score = .93; severe = .88; minor = .86, medical = .88).[9]

The MOS-SS[10] is a twelve-item questionnaire developed for patients with chronic illness, measuring six dimensions of sleep over the past four weeks; sleep initiation (time to fall asleep), quantity (hours per night sleeping), maintenance, respiratory problems, perceived adequacy, and somnolence. A sleep problems index which summarises information from nine items may also be scored. Responses are recoded on a 0 – 100 scale, with higher scores indicating more of the concept being measured (e.g., greater difficulties falling asleep). Research has supported the internal consistency of the MOS-SS scales to be acceptable (.70) or higher in samples of patients with neuropathic pain.[11 12]

The SOT[13] assesses participants' attitudes towards their online administered treatment, and will be used at the study endline. Participants state how strongly they agree/disagree with each of the following items: (a) the use of a computer to access treatment, (b) level of easiness of the online intervention, (c) duration of the potential effects of the treatment, and (d) potential recommendation of online interventions to others. Responses range from "*Agree very strongly*" to "*Disagree very strongly*". An additional question asks participants how helpful they have found the online intervention, with responses ranging from "*Very helpful*" to "*Not at all helpful*". Two open questions are also included that require participants to state the aspects of the intervention they most liked and disliked.

The EOT, developed by the researchers, will be presented at the end of every online session. The EOT includes three questions. The first asks participants to indicate where they

For peer review only - http://bmjopen.bmj.com/site/about/guidelines.xhtml

completed the online training session (e.g., home or office). The second is related to whether or not they have been distracted during the training and, if distracted, to indicate the source of distraction (e.g., telephone ringing). The third question asks participants to rate the level of their concentration during the online task on an 11-point Visual Analogue Scale (VAS) numbered from 0 (i.e., *no concentration at all*) to 10 (i.e. *very concentrated*).

For peer review only

For peer review only - http://bmjopen.bmj.com/site/about/guidelines.xhtml

References

1. Cleeland CS, Ryan KM. Pain assessment: global use of the Brief Pain Inventory. Ann Acad Med Singapore 1994;**23**(2):129-38.

2. Jensen MP. The validity and reliability of pain measures in adults with cancer. The Journal of Pain 2003;**4**(1):2-21 doi: 10.1054/jpai.2003.1.

3. Tan G, Jensen MP, Thornby JI, et al. Validation of the Brief Pain Inventory for chronic nonmalignant pain. The Journal of Pain 2004;**5**(2):133-37 doi: 10.1016/j.jpain.2003.12.005.

4. Zigmond AS, Snaith RP. The hospital anxiety and depression scale. Acta Psychiatr Scand 1983;**67**(6):361-70.

5. Bjelland I, Dahl AA, Tangen T, et al. The validity of the Hospital Anxiety and Depression Scale: An updated literature review. Journal of Psychosomatic Research 2002;**52**:69-77 doi: 10.1016/S0022-3999(01)00296-3.

6. Spielberger CD, Gorsuch RL, Lushene RE. *State Trait Anxiety Inventory*. Palo Alto, California: Consulting Psychologists Press, 1970.

7. Barnes LLB, Harp D, Jung WS. Reliability generalization of scores on the Spielberger State-Trait Anxiety Inventory. Educational and Psychological Measurement 2002;**62**(4):603 - 18 doi: 10.1177/0013164402062004005.

8. McNeil DW, Rainwater A. Development of the Fear of Pain Questionnaire-III. Journal of Behavioral Medicine 1998;**21**(4):389-410 doi: 10.1023/A:1018782831217.

9. Roelofs J, Peters ML, Deutz J, et al. The Fear of Pain Questionnaire (FPQ): further psychometric examination in a non-clinical sample. PAIN 2005;**116**(3):339-46 doi: 10.1016/j.pain.2005.05.003.

10. Hays RD, Stewart AL. Sleep Measures. Measuring functioning and well-being. In: Steweart AL, JEJ W, eds. Measuring function and well-being; the medical outcome study approach. Duke: Duke University Press, 1992:235-59.

For peer review only

11. Hays RD, Martin SA, Sesti AM, et al. Psychometric properties of the medical outcomes study sleep measure. Sleep Medicine 2005;**6**(1):41-44 doi: 10.1016/j.sleep.2004.07.006.

12. Viala-Danten M, Martin S, Guillemin I, et al. Evaluation of the reliability and validity of the Medical Outcomes Study sleep scale in patients with painful diabetic peripheral neuropathy during an international clinical trial. Health and Quality of Life Outcomes 2008;**6**(113) doi: 10.1186/1477-7525-6-113.

13. Richards D, Timulak L. Satisfaction with therapist-delivered vs. self-administered online cognitive behavioural treatments for depression symptoms in college students. British Journal of Guidance & Counselling 2013;**41**(2):193-207 doi: 10.1080/03069885.2012.726347.

For peer review only

Supplementary Material 3 – Think Aloud Study

A qualitative think aloud study[1 2] was conducted following the development of the initial version of the ABMT intervention to address the question: *What are the thoughts and first impressions of individuals with chronic musculoskeletal pain towards an online ABMT programme?* Ethical approval was obtained from the University of Southampton Research Ethics Committee (ERGO ID: 26486). Thirteen participants (mean age = 37.54, *SD* = 16.31, range 21 – 60 years) with chronic musculoskeletal pain were recruited from the South of England from a chronic pain support group.

Each participant met with the researcher (FH) on a single occasion to take part in a think aloud interview followed by a short semi-structured interview. Participants navigated their way through the iABMT Lifeguide website and completed the first assessment visual-probe task, with the researcher seated next to them. The researcher used an interview topic guide featuring standardized instructions that were read at the beginning of the study, as well as a list of pre-determined neutral prompts used when the participant stopped talking or showed a reaction to the material through their facial expressions. On occasions where the participant deviated from the topic, the researcher attempted to steer the participant's focus back to the task at hand. The prompts used were similar to those in former research[3], such as '*what are you thinking right now?*' and '*tell me why you think that*'.

Following the think-aloud interview, a short semi-structured interview took place for the researcher to ask questions about any specific aspects of the intervention that had not yet been discussed. Questions were also asked regarding thoughts about the intervention as a whole. This method of triangulation is commonly used to complement a think-aloud interview because it can be difficult to interpret spoken thoughts alone.[4 5] Following transcription of the interviews, thematic analysis using inductive encoding was used to identify themes that clearly represent the raw data, following guidelines set out by Braun and

For peer review only - http://bmjopen.bmj.com/site/about/guidelines.xhtml

Clarke.[6] Three themes and 13 subthemes were identified, which are shown in a thematic map in Figure S1 and defined in Table S1 below. While it is beyond the scope of this article to present the results of this thematic analysis in full, the main points resulting in amendments to the iABMT intervention are presented here.

Regarding the iABMT website, participants liked the continuity of design between webpages, although pointed out inconsistencies in the formatting of text, which have been rectified. On some pages the volume of text was considered to be too great, and therefore where possible text has been reduced, language simplified, better use of spacing made, and longer paragraphs divided into multiple shorter paragraphs. While some participants were positive about the inclusion of images, others felt additional visual stimuli could be added throughout. It was therefore decided to only include images where these would help emphasize the points being made. Regarding the visual-probe task sessions, one important practicality raised was ability of participants to stay seated for a sufficient amount of time in order to complete the task in a single session. An additional inclusion criteria has therefore been added to the study, being that participants must be able to sit at a personal computer for forty minutes. It was also mentioned that participants needed sufficient computer skills to take part in the study. The inclusion criteria were therefore amended to state that participants need both access to and familiarity using a Window-based computer.

For peer review only - http://bmjopen.bmj.com/site/about/guidelines.xhtml

References

1. Yardley L, Ainsworth B, Arden-Close E, et al. The person-based approach to enhancing the acceptability and feasibility of interventions. Pilot and Feasibility Studies 2015;**1**(1):37.

2. Van den Haak MJ, De Jong MD, Schellens PJ. Evaluation of an informational web site: three variants of the think-aloud method compared. Technical Communication 2007;**54**(1):58-71.

3. Morrison L, Moss-Morris R, Michie S, et al. Optimizing engagement with internet-based health behaviour change interventions: Comparison of self-assessment with and without tailored feedback using a mixed methods approach. British Journal of Health Psychology 2014;**19**(4):839-55 doi: 10.1111/bjhp.12083.

4. Charters E. The use of think-aloud methods in qualitative research an introduction to think-aloud methods. Brock Education Journal 2003;**12**(2).

5. Anthierens S, Tonkin-Crine S, Douglas E, et al. General practitioners' views on the acceptability and applicability of a web-based intervention to reduce antibiotic prescribing for acute cough in multiple European countries: a qualitative study prior to a randomised trial. BMC Family Practice 2012;**13**(101) doi: 10.1186/1471-2296-13-101.

6. Braun V, Clarke V. Using thematic analysis in psychology. Qualitative Research in Psychology 2006;**3**(2):77-101 doi: 10.1191/1478088706qp063oa.

For peer review only - http://bmjopen.bmj.com/site/about/guidelines.xhtml

ATTENTIONAL BIAS MODIFICATION PROTOCOL

Table S1. Definitions of themes and subthemes identified in the think aloud study

Theme/Sub-theme	Definition
A. AMBT website	Comments relating to the ABMT website including the post-intervention questionnaires
A1. Accessibility and usability	Comments relating to the ease with which participants were able to use the website and the extent to which it was deemed to be accessible
A2. Visual design	Comments relating to the visual design and appearance of the website, including colour, orientation of elements and text
A3. Questionnaires	Comments regarding any aspect of the post-intervention questionnaires, including their purpose and presentation
A4. Specific menu pages	Comments pertaining to the six main menu pages, which are titled (i) How does ABMT work, (ii) Will ABMT cure my pain, (iii) The team behind ABMT, (iv) Evidence for ABMT, (v) Why try ABMT, and (iv) What is it like to use ABMT?
B. Visual-probe task	Comments relating to any and all aspects of the visual-probe tasks
B1. Experiences of testing the intervention	Comments provided prior to and during testing of the visual-probe task
B2. Attitudes towards task	Positive and negative attitudes expressed by participants regarding the visual-probe task
B3. Practicalities	Comments pertaining to the feasibility and practicalities of completing the visual-probe task, including commitment needed
C. Influencing factors	All personal and situational factors that may affect the participants' experiences of testing the intervention
C1. Interviewing situation	Comments relating to the style of qualitative research adopted for the think aloud study
C2. Personal barriers	Comments relating to personal factors that hindered participants' completion of the visual-probe task
C3. Personal facilitators	Comments relating to personal factors that increased participants' ability to complete the visual-probe task and improved their attitude regarding it

For peer review only - http://bmjopen.bmj.com/site/about/guidelines.xhtml

For peer review only

STANDARD PROTOCOL ITEMS: RECOMMENDATIONS FOR INTERVENTIONAL TRIALS

SPIRIT 2013 Checklist: Recommended items to address in a clinical trial protocol and related documents*

Section/item	Item No	Description	Addressed on page number
**Administrative information**			
Title	1	Descriptive title identifying the study design, population, interventions, and, if applicable, trial acronym	1
Trial registration	2a	Trial identifier and registry name. If not yet registered, name of intended registry	1
	2b	All items from the World Health Organization Trial Registration Data Set	Registered with clinicaltrials.gov
Protocol version	3	Date and version identifier	1
Funding	4	Sources and types of financial, material, and other support	25
Roles and responsibilities	5a	Names, affiliations, and roles of protocol contributors	1, 25
	5b	Name and contact information for the trial sponsor	25
	5c	Role of study sponsor and funders, if any, in study design; collection, management, analysis, and interpretation of data; writing of the report; and the decision to submit the report for publication, including whether they will have ultimate authority over any of these activities	25
	5d	Composition, roles, and responsibilities of the coordinating centre, steering committee, endpoint adjudication committee, data management team, and other individuals or groups overseeing the trial, if applicable (see Item 21a for data monitoring committee)	________

For peer review only

For peer review only - http://bmjopen.bmj.com/site/about/guidelines.xhtml

**Introduction**			
Background and rationale	6a	Description of research question and justification for undertaking the trial, including summary of relevant studies (published and unpublished) examining benefits and harms for each intervention	4-6
	6b	Explanation for choice of comparators	4
Objectives	7	Specific objectives or hypotheses	6
Trial design	8	Description of trial design including type of trial (eg, parallel group, crossover, factorial, single group), allocation ratio, and framework (eg, superiority, equivalence, noninferiority, exploratory)	7-8
**Methods: Participants, interventions, and outcomes**			
Study setting	9	Description of study settings (eg, community clinic, academic hospital) and list of countries where data will be collected. Reference to where list of study sites can be obtained	6-8
Eligibility criteria	10	Inclusion and exclusion criteria for participants. If applicable, eligibility criteria for study centres and individuals who will perform the interventions (eg, surgeons, psychotherapists)	6
Interventions	11a	Interventions for each group with sufficient detail to allow replication, including how and when they will be administered	7-13
	11b	Criteria for discontinuing or modifying allocated interventions for a given trial participant (eg, drug dose change in response to harms, participant request, or improving/worsening disease)	17
	11c	Strategies to improve adherence to intervention protocols, and any procedures for monitoring adherence (eg, drug tablet return, laboratory tests)	12
	11d	Relevant concomitant care and interventions that are permitted or prohibited during the trial	6-7
Outcomes	12	Primary, secondary, and other outcomes, including the specific measurement variable (eg, systolic blood pressure), analysis metric (eg, change from baseline, final value, time to event), method of aggregation (eg, median, proportion), and time point for each outcome. Explanation of the clinical relevance of chosen efficacy and harm outcomes is strongly recommended	6, 14
Participant timeline	13	Time schedule of enrolment, interventions (including any run-ins and washouts), assessments, and visits for participants. A schematic diagram is highly recommended (see Figure)	Table 1

For peer review only - http://bmjopen.bmj.com/site/about/guidelines.xhtml

Sample size	14	Estimated number of participants needed to achieve study objectives and how it was determined, including clinical and statistical assumptions supporting any sample size calculations	6-7
Recruitment	15	Strategies for achieving adequate participant enrolment to reach target sample size	6-7
**Methods: Assignment of interventions (for controlled trials)**			
Allocation:			
Sequence generation	16a	Method of generating the allocation sequence (eg, computer-generated random numbers), and list of any factors for stratification. To reduce predictability of a random sequence, details of any planned restriction (eg, blocking) should be provided in a separate document that is unavailable to those who enrol participants or assign interventions	7-8
Allocation concealment mechanism	16b	Mechanism of implementing the allocation sequence (eg, central telephone; sequentially numbered, opaque, sealed envelopes), describing any steps to conceal the sequence until interventions are assigned	7-8
Implementation	16c	Who will generate the allocation sequence, who will enrol participants, and who will assign participants to interventions	7-8
Blinding (masking)	17a	Who will be blinded after assignment to interventions (eg, trial participants, care providers, outcome assessors, data analysts), and how	7-8
	17b	If blinded, circumstances under which unblinding is permissible, and procedure for revealing a participant's allocated intervention during the trial	7-8
**Methods: Data collection, management, and analysis**			
Data collection methods	18a	Plans for assessment and collection of outcome, baseline, and other trial data, including any related processes to promote data quality (eg, duplicate measurements, training of assessors) and a description of study instruments (eg, questionnaires, laboratory tests) along with their reliability and validity, if known. Reference to where data collection forms can be found, if not in the protocol	14, Supplementary material 2
	18b	Plans to promote participant retention and complete follow-up, including list of any outcome data to be collected for participants who discontinue or deviate from intervention protocols	11

Data management	19	Plans for data entry, coding, security, and storage, including any related processes to promote data quality (eg, double data entry; range checks for data values). Reference to where details of data management procedures can be found, if not in the protocol	16
Statistical methods	20a	Statistical methods for analysing primary and secondary outcomes. Reference to where other details of the statistical analysis plan can be found, if not in the protocol	15-16
	20b	Methods for any additional analyses (eg, subgroup and adjusted analyses)	N/A
	20c	Definition of analysis population relating to protocol non-adherence (eg, as randomised analysis), and any statistical methods to handle missing data (eg, multiple imputation)	15
**Methods: Monitoring**			
Data monitoring	21a	Composition of data monitoring committee (DMC); summary of its role and reporting structure; statement of whether it is independent from the sponsor and competing interests; and reference to where further details about its charter can be found, if not in the protocol. Alternatively, an explanation of why a DMC is not needed	______________
	21b	Description of any interim analyses and stopping guidelines, including who will have access to these interim results and make the final decision to terminate the trial	______________
Harms	22	Plans for collecting, assessing, reporting, and managing solicited and spontaneously reported adverse events and other unintended effects of trial interventions or trial conduct	17
Auditing	23	Frequency and procedures for auditing trial conduct, if any, and whether the process will be independent from investigators and the sponsor	______________
**Ethics and dissemination**			
Research ethics approval	24	Plans for seeking research ethics committee/institutional review board (REC/IRB) approval	16-17
Protocol amendments	25	Plans for communicating important protocol modifications (eg, changes to eligibility criteria, outcomes, analyses) to relevant parties (eg, investigators, REC/IRBs, trial participants, trial registries, journals, regulators)	16

For peer review only - http://bmjopen.bmj.com/site/about/guidelines.xhtml

Consent or assent	26a	Who will obtain informed consent or assent from potential trial participants or authorised surrogates, and how (see Item 32)	9, 16
	26b	Additional consent provisions for collection and use of participant data and biological specimens in ancillary studies, if applicable	____________
Confidentiality	27	How personal information about potential and enrolled participants will be collected, shared, and maintained in order to protect confidentiality before, during, and after the trial	16-17
Declaration of interests	28	Financial and other competing interests for principal investigators for the overall trial and each study site	25
Access to data	29	Statement of who will have access to the final trial dataset, and disclosure of contractual agreements that limit such access for investigators	25
Ancillary and post-trial care	30	Provisions, if any, for ancillary and post-trial care, and for compensation to those who suffer harm from trial participation	____________
Dissemination policy	31a	Plans for investigators and sponsor to communicate trial results to participants, healthcare professionals, the public, and other relevant groups (eg, via publication, reporting in results databases, or other data sharing arrangements), including any publication restrictions	17
	31b	Authorship eligibility guidelines and any intended use of professional writers	25
	31c	Plans, if any, for granting public access to the full protocol, participant-level dataset, and statistical code	17
**Appendices**			
Informed consent materials	32	Model consent form and other related documentation given to participants and authorised surrogates	Supplementary materials
Biological specimens	33	Plans for collection, laboratory evaluation, and storage of biological specimens for genetic or molecular analysis in the current trial and for future use in ancillary studies, if applicable	N/A

*It is strongly recommended that this checklist be read in conjunction with the SPIRIT 2013 Explanation & Elaboration for important clarification on the items. Amendments to the protocol should be tracked and dated. The SPIRIT checklist is copyrighted by the SPIRIT Group under the Creative Commons "Attribution-NonCommercial-NoDerivs 3.0 Unported" license.

For peer review only

BMJ Open

Internet-delivered attentional bias modification training (iABMT) for the management of chronic musculoskeletal pain: a protocol for a randomised controlled trial

Journal:	*BMJ Open*
Manuscript ID	bmjopen-2019-030607.R2
Article Type:	Protocol
Date Submitted by the Author:	02-Dec-2019
Complete List of Authors:	Liossi, Christina; University of Southampton Faculty of Engineering and the Environment, Psychology;<br>Georgallis, Tsampikos; University of Southampton Faculty of Engineering and the Environment, School of Psychology<br>Zhang, Jin; University of Southampton Faculty of Engineering and the Environment, School of Psychology<br>Hamilton, Fiona; University of Southampton Faculty of Engineering and the Environment, School of Psychology<br>White, Paul; University of the West of England, Engineering, Design and Mathematics<br>Schoth, Daniel; University of Southampton Faculty of Engineering and the Environment, School of Psychology
**Primary Subject Heading**:	Neurology
Secondary Subject Heading:	Paediatrics, Qualitative research
Keywords:	Chronic pain, Musculoskeletal pain, Visual-probe task, Attention bias modification, Internet-delivered therapy, Randomised-controlled trial

SCHOLARONE™
Manuscripts

I, the Submitting Author has the right to grant and does grant on behalf of all authors of the Work (as defined in the below author licence), an exclusive licence and/or a non-exclusive licence for contributions from authors who are: i) UK Crown employees; ii) where BMJ has agreed a CC-BY licence shall apply, and/or iii) in accordance with the terms applicable for US Federal Government officers or employees acting as part of their official duties; on a worldwide, perpetual, irrevocable, royalty-free basis to BMJ Publishing Group Ltd ("BMJ") its licensees and where the relevant Journal is co-owned by BMJ to the co-owners of the Journal, to publish the Work in this journal and any other BMJ products and to exploit all rights, as set out in our licence.

The Submitting Author accepts and understands that any supply made under these terms is made by BMJ to the Submitting Author unless you are acting as an employee on behalf of your employer or a postgraduate student of an affiliated institution which is paying any applicable article publishing charge ("APC") for Open Access articles. Where the Submitting Author wishes to make the Work available on an Open Access basis (and intends to pay the relevant APC), the terms of reuse of such Open Access shall be governed by a Creative Commons licence – details of these licences and which Creative Commons licence will apply to this Work are set out in our licence referred to above.

Other than as permitted in any relevant BMJ Author's Self Archiving Policies, I confirm this Work has not been accepted for publication elsewhere, is not being considered for publication elsewhere and does not duplicate material already published. I confirm all authors consent to publication of this Work and authorise the granting of this licence.

For peer review only

Internet-delivered attentional bias modification training (iABMT) for the management of chronic musculoskeletal pain: a protocol for a randomised controlled trial

Christina Liossi[1*], Tsampikos Georgallis[1], Jin Zhang[1], Fiona Hamilton[1], Paul White[2], and Daniel E. Schoth[1,]

[1]Pain Research Laboratory, School of Psychology, University of Southampton, Highfield, Southampton, UK;

[2] Engineering Design and Mathematics, University of the West of England, Bristol, UK

Protocol version 4.0; 30/11/19

Corresponding author: Prof. Christina Liossi, Pain Research Laboratory, Department of Psychology, University of Southampton, Highfield, Southampton, SO17 1BJ, UK. E-mail: Cliossi@soton.ac.uk, Tel: 0044 (0)238059 4645.

Trial registration: This Study Protocol was registered in ClinicalTrials.gov (NCT02232100) on 1 September 2014

Word count: 4117

For peer review only

ATTENTIONAL BIAS MODIFICATION PROTOCOL

Abstract

Introduction: Chronic musculoskeletal pain is a complex medical condition that can significantly impact quality of life. Patients with chronic pain demonstrate attentional biases towards pain-related information, and the therapeutic benefits of modifying attentional biases by implicitly training attention away from pain-related information towards neutral information have been supported in the small number of published studies. Limited research however has explored the efficacy of modifying pain-related biases via the internet. This protocol describes a randomised, double-blind internet-delivered attentional bias modification intervention, aimed to evaluate the efficacy of the intervention on reducing pain interference. Secondary outcomes are pain intensity, state and trait anxiety, depression, pain-related fear, and sleep impairment. This study will also explore the effects of training intensity on these outcomes, along with participants' perceptions about the online therapy. **Methods and analysis**: The study is a double-blind randomised controlled trial with four arms exploring the efficacy of online attentional bias modification training versus placebo training theorised to offer no specific therapeutic benefit. Participants with chronic musculoskeletal pain will be randomised to one of four groups: (i) 10-session attentional modification group; (ii) 10-session placebo training group; (iii) 18-session attentional modification group; or (iv) 18-session placebo training group. In the attentional modification groups, the visual-probe task will be used to implicitly train attention away from threatening information towards neutral information. Following the intervention, participants will complete a short interview exploring their perceptions about the online training. In addition, a subgroup analysis for by participant age 16 – 24 and 25 – 60 will be undertaken. **Ethics and dissemination**: This study has been approved by the University of Southampton Research Ethics Committee. Results will be published in peer-reviewed journals, academic conferences, and in lay reports for pain charities and patient support groups. **Registration**: The study protocol has been registered with ClinicalTrial.gov (NCT02232100).

For peer review only

Strengths and limitations of this study

- This protocol describes an internet-delivered intervention to explore the efficacy of attentional bias modification training in patients with chronic musculoskeletal pain.
- Recruitment of a diverse sample in terms of age, socioeconomic status, and geographical region.
- Results will provide important information regarding dose effects, age effects and participants' experiences and attitudes using the online intervention.
- Limited control over the conditions under which training is completed.
- Impossibility of independently verifying participant diagnosis.

Keywords: chronic pain; musculoskeletal pain; visual-probe task; attention bias modification; internet-delivered therapy; randomised-controlled trial.

For peer review only

Introduction

Chronic musculoskeletal pain (CMSK) is pain arising from bones, muscles, ligaments, tendons and/or joints lasting for more than three months.[1] Prevalence estimates of CMSK vary considerably, which for adults is estimated to affect between 11.4 and 24% of the population[2] and for children and adolescents ranges between 4 and 40% of the population.[3] Negative effects on quality of life are commonly reported in adult[4-7] and paediatric[8-11] CMSK populations, and theory driven research[12-14] has shown that patients with chronic pain exhibit attentional biases (i.e., a selective attention) for salient pain-related words (e.g.,[15-18]) and images (e.g.,[19 20]) relative to neutral information. Three meta-analyses have supported the presence of attentional biases in chronic pain patients[21-23], although evidence for the time-course of such biases is mixed. Specifically, two meta-analyses reported larger effect sizes when stimuli are presented for longer than 1000 ms,[21 23] whereas a recent analysis found evidence of bias for presentation times of 500 – 1000ms but not when stimuli are presented for longer than 1000 ms.[22] Despite this inconsistency, attentional biases are commonly shown in chronic pain, and preliminary research has explored the effects of re-directing attention may have on pain and pain-related distress using attentional bias modification (ABM) techniques. ABM is a computer-based intervention which implicitly trains attention away from threat-related cues toward neutral information, and is frequently achieved through a modified version of the visual-probe task[24] (Figure 1).

Two small randomised controlled trials (RCTs)[25 26] and one pilot study[27] have used the modified visual-probe task to assess ABM in adults with chronic pain. Carleton and colleagues[25] found participants receiving ABM showed significant reductions pre- to post ABM in anxiety sensitivity and fear of pain, and marginally significant reduction in current pain severity. No significant reductions were found in illness/injury sensitivity or pain anxiety. Significantly more patients receiving ABM reported clinically significant change in current pain compared with those in the placebo group (44% vs. 17%, respectively). Attentional bias scores were not recorded, and therefore the researchers could not comment on mechanisms of action. Sharpe and

For peer review only - http://bmjopen.bmj.com/site/about/guidelines.xhtml

colleagues[26] found participants receiving ABM, compared to those in a placebo group, showed significant improvements in pain-related disability after the ABM. No significant improvements were found for pain, fear of pain, fear of re-injury, depression, anxiety, stress, anxiety sensitivity or pain self-efficacy. At 6-month follow-up (and after all participants had undergone 8 sessions of CBT) the ABM group, compared to the placebo group, showed significant improvements in disability and anxiety sensitivity, and marginally significant improvements in fear of re-injury. No other significant effects were found, and the mechanism of action could not be established as ABM was not found to change patterns of attentional bias across time. Schoth and colleagues[27] reported statistically and clinically significant change pre- to post-ABM in pain intensity, pain interference, anxiety, and depression. Assessed via the McGill Pain Questionnaire[28], significant changes pre- to post-ABM were shown for total pain, affective pain and miscellaneous pain, but not sensory-pain or evaluative pain. There was also no evidence that attentional biases changed across time.

One small RCT in adolescents with chronic pain randomised participants to either ABM, placebo training or waiting-list groups. No significant differences were found between the three groups in terms of pain, disability, pain catastrophising, anxiety, depression, or objective measures of physical functioning (sit-to-stand task and cardio wall tasks), and no evidence was found that ABM changed attentional bias or attentional control compared to placebo and waiting-list conditions.[29] Overall, while some studies have provided support for the benefits of ABM in adults, the evidence in regards to specific outcome variables is mixed and the mechanisms of action have yet to be established. Given the methodological shortcomings and small sample sizes of previous studies however, further research is needed to determine the therapeutic benefits of ABM for patients with chronic pain, along with the optimal form ABM should take and the impact of age on outcomes and intervention engagement. Considering the optimal form of ABM, a review of the broader cognitive bias modification (CBM) literature identified 12 published meta-analyses, 11 examining ABM specifically.[30] Three out of six analyses found the number of

For peer review only - http://bmjopen.bmj.com/site/about/guidelines.xhtml

sessions to be a significant moderator of CBM, and three out of nine analyses found the number of sessions to be a significant moderator of symptom outcomes. Although evidence is therefore mixed, it is nevertheless worth exploring whether number of sessions impacts patterns of attentional bias and pain-related outcomes in individuals with chronic pain.

This article presents a study protocol for a randomised, double-blind internet-delivered ABM intervention. The results of a pilot think aloud study informing the design of the intervention are also presented. The aim of this RCT is to assess the efficacy of internet-delivered ABM in improving pain and pain-related distress in people aged 16 –60 with CMSK, and will report a subgroup by treatment effects using the two age brackets of 16-24 and 25-60. While only limited evidence has been found for age as a moderator of CBM on bias change and symptoms,[30] it has been argued that the age of adolescence should be extended from 19 to 24 as the latter corresponds more closely to adolescent growth and popular understandings of this life phase.[31] Furthermore, although use of the internet has been growing among all age groups, there are nevertheless still some differences in internet use and activity according to age (e.g., in the UK 1% of those aged 16 to 24 do not use the internet, compared to 7% aged 45-54),[32] thus warranting a separate consideration of adolescent and adult participants. Overall, a diverse sample in terms of age, socioeconomic status, and geographical region will be recruited, thus increasing generalisability of results to the CMSK population. The primary outcome measure is pain interference. Secondary outcome measures are pain intensity, state and trait anxiety, depression, pain-related fear and sleep impairment, and engagement to and satisfaction with the online visual-probe training task. Dose effects will be explored via the inclusion of 10 versus 18 session conditions. It is hypothesised that participants in the intervention group, relative to those in the placebo group, will report significant reductions in pain interference, pain intensity, anxiety, depression, sleep impairment and pain-related fear at study endline. It is also hypothesised that dose will significantly moderate these findings, with greater effects in 18 compared to 10 sessions.

For peer review only

Methods and Analysis

Participants

Participants will be recruited via press announcements, social media, and patient organizations throughout the UK. Inclusion criteria will be: (a) aged between 16 to 60 years old, (b) experiencing any type of CMSK (i.e., any condition that involves pain lasting for more than three months and arises from bones, muscles and/or joints), (c) able to sit at a personal computer for forty minutes, (d) normal or corrected to normal vision, (e) access to the internet at least twice a week, (f) access to, and familiarity using, a Windows-based computer, (g) successful completion of primary school, and (h) living in the United Kingdom. Exclusion criteria will be (1) experiencing malignant CMSK (i.e., pain caused due to a tumour), (2) a diagnosis of other forms of comorbid chronic pain (e.g., chronic headache), and (3) a diagnosis of any psychiatric disorder, either currently or within the last 5 years. There are no restrictions placed on concomitant care and participants are not required to make any changes to current treatments they may be receiving.

Patient and Public Involvement

Participant burden of the intervention and outcome measures were assessed using individual interviews and informal feedback from patients participating in two pilot studies.[27 33] Patients will not be involved in recruitment of participants or conduct of the study. Results of this study will be disseminated to participants through presentation at client and community forums.

Study Design and Setting

The study is a double-blind randomised controlled trial (RCT) with four arms exploring the efficacy of online ABM training versus a placebo training condition theorised to offer no specific therapeutic benefits. The choice of comparator was selected as placebo training closely mimics attentional bias modification training, and is frequently used as a comparator in ABM studies (e.g.,[25 26 34 35]). The study will take place in a location of the participant’s choice (which

For peer review only - http://bmjopen.bmj.com/site/about/guidelines.xhtml

we anticipate will typically be their home), which is accessed via personal computer connected to the internet.

The study design has a 2 x 2 x 3 experimental structure, with multiple non-commensurate dependent variables measuring pain intensity and interference, emotional functioning and pain-related disability. Treatment is a two-level factor (ABM, Placebo), number of Sessions is a two-level factor (10 sessions, 18 sessions), with measures recorded at three points in time (baseline, endline, and 6-month follow-up). Measures in the 18 session groups (18AMG and 18 PTG) will also be taken after session 10.

Randomisation and Sample Size

Block randomisation of size four, performed via a randomisation command in the Lifeguide platform[36], will be used to sequentially randomise eligible consecutive consenting participants to one of four groups: (i) 10-session attentional modification group (10AMG); (ii) 10-session placebo training group (10PTG); (iii) 18-session attentional modification group (18AMG); or (iv) 18-session placebo training group (18PTG). This blocked randomisation will be performed separately for those aged 16-24 and those aged 25-60. An allocation concealment mechanism will be used to ensure neither participants nor researchers know the study group to which the next participant will be assigned. This will be automatically performed within the Lifeguide platform (see below), with participants provided access to appropriate AMG or PTG sessions. Participants randomised to either 10-session condition will receive ten 35-minute online sessions across a four-week period, while those randomised to either 18-session condition will receive eighteen 35-minute online sessions across an eight-week period. All online sessions will be administered twice a week on a separate set of days (Monday-Thursday, Tuesday-Friday). Participants randomised to either AMG condition will receive attentional training via the modified training version of the visual probe-task, while those randomised to either PTG condition will receive placebo training via the standard version of the visual-probe task. To maintain the overall quality and legitimacy of the intervention, code breaks (i.e., unblinding) will

For peer review only

only occur in exceptional circumstances. Adherence will be monitored electronically via Lifeguide.

Our former lab-based proof-of-concept ABM intervention found reductions in pain intensity and pain interference pre- to post-ABM were associated with large effect sizes[27], although differences in methodology including delivery (online vs lab-based) and number of sessions warrants a more conservative estimate. On this basis, the study will recruit a minimum of N = 100 with 1:1:1:1 randomisation. This study is powered primarily to establish treatment efficacy for the primary outcome variable of pain interference by considering baseline and measures after session 10 between those randomised to an attentional modification arm and those randomised to a placebo arm. Randomisation should ensure baseline comparability on measures between these two groups, and a difference in beneficial outcomes between these groups will be modelled using a linear mixed model. For an assumed correlation of 0.7 between baseline and end of session 10 measures a total of $n = 40$ per group (i.e. 20 per randomized arm) would be needed to detect small to moderate standardized intervention differences (Cohen's $d = 0.25$) with at least 80% power (alpha = 0.05, two-sided). The recruitment strategy includes a 20% oversampling to mitigate a loss of power from loss to follow-up

Procedure

Interested individuals will contact the researchers on the details provided in study advertisements and will be assessed against the inclusion and exclusion criteria via telephone interview. If eligible, they will receive a copy of the participant information sheet (PIS) detailing the requirements and procedure of the study, along with a link to the study website which provides further details on ABM and the research team. If the individual consents to take part they will be required to create an online ABM account. During registration, participants will select their preferred days for training, which will be either (i) Monday/Thursday or (ii) Tuesday/Friday.

For peer review only - http://bmjopen.bmj.com/site/about/guidelines.xhtml

ATTENTIONAL BIAS MODIFICATION PROTOCOL

Session 1 is the baseline session, and includes the first assessment visual-probe task assessing attentional biases prior to beginning the intervention, along with the questionnaire battery assessing the primary and secondary outcome measures. Randomisation will take place following session 1, where participants will be informed of the length of their training (i.e., four or eight weeks). Participants randomised to either of the ten session conditions will complete four pictorial and four linguistic training sessions, while those randomised to either of the 18 session conditions will complete eight pictorial and eight linguistic training sessions (baseline and endline assessment sessions comprise the remainder of the 10/18 sessions). Participants' attentional biases will be re-assessed at endline (i.e., during the final session 10/18) using the same assessment version of the visual probe task (i.e., featuring the same stimuli) used at baseline and the same questionnaire battery. Participants randomised to either of the 18 session conditions will also have their attentional biases re-assessed in session 10 after their training session.

Participants' satisfaction with the online training will also be assessed. Six months after the last session, participants will be invited to again complete the online assessment visual-probe task (same as baseline and final session 10/18) and questionnaire battery. Within two weeks of the completion of the six-month follow-up session, participants will be invited to take part in a brief telephone semi-structured interview including 15 questions spread across five thematic sections: (a) motivation and expectations about the study, (b) experience of the online study – person, environmental and lifestyle parameters, (c) experience of the visual-probe task, (d) understanding of the process of change, and (e) the visual-probe task as a potential therapeutic tool in pain management. The study flow is presented in Table 1.

For peer review only - http://bmjopen.bmj.com/site/about/guidelines.xhtml

ATTENTIONAL BIAS MODIFICATION PROTOCOL

Table 1. Schedule of enrolment, intervention, and assessments.

	STUDY PERIOD					
	Enrolment	Post-allocation				
**TIMEPOINT****	$-t_1$	t_1 Baseline	t_2 Training	t_3 Evaluation	t_4 Follow-up	t_5 Study exit
**ENROLMENT:**						
**Eligibility screen**	X					
**Informed consent**	X					
**Allocation**		X				
**INTERVENTIONS:**						
Attentional bias modification training			X			
**ASSESSMENTS:**						
Demographic questionnaire		X				
EOT		X	X	X	X	
BPI-SF		X		X	X	
HADS		X		X	X	
STAI		X		X	X	
MOS-SS		X		X	X	
FOP-III		X		X	X	
SOT				X		
Interview						X

T1 = Baseline phase including standard assessment visual-probe task and questionnaires. Also includes randomization to groups.
T2 = Training phase including training visual-probe tasks.
T3 = Evaluation phase including standard assessment visual-probe task and questionnaires. Participants randomized to 18 session conditions also complete evaluation phase during session 10.
T4 = Six-month follow-up including standard assessment visual-probe task and questionnaires.
T5 = Telephone interview within two weeks of T4.

Note. EOT, Engagement with the Online Training questionnaire; BPI-SF, Brief Pain Inventory-Short Form; HADS, Hospital Anxiety and Depression Scale; STAI, State-Trait Anxiety Inventory; MOS-SS, Medical Outcome Study –Sleep Scale, FOP-III, Fear of pain questionnaire III; SOT, Satisfaction with the online treatment questionnaire

To retain as many participants as possible, reminder emails will be sent to participants 24 hours before each scheduled session becomes available, and also at 13:00 on the scheduled session date should the participant not have already logged into their iABMT account. An

For peer review only - http://bmjopen.bmj.com/site/about/guidelines.xhtml

additional reminder email will be sent one week before their 6-month follow-up session is ready. Participants will also be sent encouragement emails informing them when they have reach specific milestones (i.e., successfully completing 5 sessions for those randomised to complete 10 sessions; successfully completing 6 and 12 sessions for those randomised to 18 sessions). Participants will also have the option of receiving an SMS message on their mobile phone at 09:00 on the morning of each scheduled session.

The Intervention

The intervention will be hosted using LifeGuide and iSurvey platforms, both developed by the University of Southampton. LifeGuide is an open-source software which allows researchers to create online interventions.[36] iSurvey is a survey generation tool which allows researchers to create and disseminate questionnaires online. Participants will register for the intervention using LifeGuide, which will also host the majority of the questionnaires. iSurvey will be used to host the visual-probe tasks, along with the Engagement with Online Training questionnaire and the Brief Pain Inventory[37] that appear at the end of every visual-probe task session. The flow of sessions is provided in Figure 2, and examples of webpages are shown in Figure 3. On loading the intervention homepage, the individual is provided with a brief welcome to the website and rationale for the intervention. A recent review of cognitive bias meta-analyses concluded that effect sizes were smaller when biases were modified remotely compared to laboratory-based training.[30] We therefore decided to provide information on the rationale behind ABMT in order to increase motivation and reduce attrition as much as possible, which also mirrors clinical practice that involves educating patients into chronic pain and the psychological interventions used in its management. Individuals are able to either create an iABMT account or log into their existing account. Before any session participants are asked to indicate whether they have enough time and are ready to begin the session. Participants are only permitted to complete one session per day. Should a participant complete their missed session on a subsequently scheduled date (e.g., completing their scheduled session from Monday on Thursday instead), all

For peer review only - http://bmjopen.bmj.com/site/about/guidelines.xhtml

remaining sessions are shifted back accordingly. This process retains the structure of the intervention.

Guidance has been informed by research showing attention to be a limited capacity resource[38 39], sleepiness to negatively influence concentration and performance[40], alcohol to impair speed of information processing and cognition[41], energy drinks to be associated with restlessness and nervousness[42], caffeine to improve alertness[43], large meals to be associated with impairments in cognitive functioning[44], and hunger to influence patterns of attention.[45 46] Participants are provided with guidance to ensure concentration and comfort is maintained throughout each session: (1) allowing enough time for the session with no anticipated distractions, (2) adjusting screen brightness to match the brightness of the environment, (3) closing distracting applications such as Twitter and Facebook, (4) avoiding completing the session when tired, (5) not consuming alcohol at least 3 hours prior to the session, (6) caution in the use of energy drinks which may cause restlessness, (7) if the participant is a coffee or tea drinker, suggesting a cup 30 minutes prior to the session, and (8) avoiding completing the session when hungry or directly after a meal.

Visual-Probe Tasks. The flow of visual-probe sessions is illustrated in Figure 2. In total there will be eight linguistic and eight pictorial visual-probe training tasks used in the study, each with different stimuli. A complete description of the stimuli including ratings of valence, arousal, pain intensity, written frequency, and low-level features, is provided in Supplementary Material 1. To briefly summarise, each linguistic visual-probe task includes 32 threatening/neutral and 16 neutral/neutral word pairs. Each task includes eight threatening words from each of the following categories: sensory-pain, affective-pain, health-threat and general-threat. Each pictorial task includes 32 threatening/neutral and 16 neutral/neutral image pairs. Each task includes eight threatening images from the following categories: musculoskeletal pain, facial expressions of pain, health-threat and general-threat. A total of 384 trials are included in each task, with each

For peer review only - http://bmjopen.bmj.com/site/about/guidelines.xhtml

stimuli-pair presented four times for 500 ms and four times for 1250 ms. Within each exposure duration, each stimulus appears twice in the upper location of the screen, and twice in the lower location.

A separate linguistic visual-probe task will be used in the three assessment sessions, featuring different stimuli to the linguistic training visual-probe tasks. The use of separate assessment and training stimuli is necessary to establish whether training effects generalise to novel stimuli not used in the actual training. The same assessment stimuli will be used in all assessment sessions. The ABM training version of the visual-probe task to be completed by AMG groups includes three main stages per trial (Figure 1). Following a 500 ms initial fixation point, a stimulus pair are presented in distinct locations (e.g., above and below the initial fixation point) for either 500 or 1250 ms. The stimulus pair may be either words or images, although critically includes one threat-related and one neutral stimulus. 500 and 1250 ms stimuli presentation times are used as these are the most common stimuli presentation times adopted in chronic pain visual-probe studies (e.g., [18 20 47]), and will allow us to closely compare our results to those of former research. Immediately following their presentation, the stimuli disappear and a probe appears (i.e., either *'p'* or *'q'*) in the location of the neutral stimulus. Participants indicate the location of the probe as quickly and as accurately as possible via corresponding keys on their keyboard. In the standard version of this paradigm completed by PTG groups and used in the assessment sessions, the probe replaces the neutral and threat-related stimulus an equal number of times.

Outcome Measures

The primary outcome measure of pain interference will be assessed by the Brief Pain Inventory – Short Form (BPI-SF[37]). Pain interference will be scored as the mean of seven interfernce items (i.e., general activity, walking, work, mood, enjoyment of life, relations with others, and sleep). Following guidance in the Brief Pain Inventory User Guide[48] and also IMMPACT recommendations for assessing pain in clinical trials,[49] we will include individual

For peer review only - http://bmjopen.bmj.com/site/about/guidelines.xhtml

assessments of ‘worst’, ‘least’, ‘average’ and ‘now’ (i.e., current) pain intensity as secondary outcome measures. Additional secondary outcome measures of state and trait anxiety will be assessed by the State Trait Anxiety Inventory (STAI [50]), depression by the Hospital Anxiety and Depression Scale (HADS[51]), pain-related fear by the Fear of Pain Questionnaire III (FOP-III[52]), sleeping impairment by the Medical Outcome Study – Sleep Scale (MOS-SS[53]), and engagement to and satisfaction with the online visual-probe training task by the Satisfaction with the Online Training questionnaire (SOT[54]) and the Engagement with the Online Training questionnaire (EOT). A demographic questionnaire developed by the researchers will be used at baseline to collect information on participants’ age, gender, ethnicity, socio-economic status, place of residence, and pain characteristics. Aside from the SOT and EOT, questionnaires in the 10 session groups (10AMG and 10PTG) will be administered to participants at baseline, after 10 sessions, and at six-month follow-up. In the 18 session groups (18AMG and 18PTG) questionnaires will be administered to participants at baseline, after 10 sessions, after 18 sessions, and at six-month follow-up. At endline for each participant we will also assess whether the participant and researchers became aware at any point prior to endline which group they had been allocated to. Full details on each outcome measure are provided in Supplementary Material 2.

Think Aloud Study

A qualitative think aloud study[33] including a short semi-structured interview was conducted following the development of the initial version of the intervention with the aim to understand participants’ first impressions and attitudes towards the intervention, and to collate feedback that could be used to make improvements. Details and outcomes of this study are provided in Supplementary Material 3.

Analyses of the RCT Data

Quantitative Analyses

A full and detailed Statistical Analysis Plan will be written prior to trial closure. In brief, the data analysis will be performed on an intention-to-treat (ITT) basis to provide an unbiased

For peer review only - http://bmjopen.bmj.com/site/about/guidelines.xhtml

estimate of the treatment effect.[55] Per protocol analyses will also be performed (comprising those correctly randomized, without protocol violations, and providing complete data on the primary outcome measure). Sweetman and Doig[56] have identified five types of protocol violations, including violations in (i) enrollment (i.e., failure to correctly apply eligibility criteria resulting in the enrolment of an inappropriate participant), (ii) randomization (i.e., violation of intended randomization sequence), (iii) study intervention (i.e., dosing, timing or delivery errors attributable to members of the research team), (iv) patient compliance (i.e., participants failing to comply with the trial protocol or other requirements of participation in the trial, such as skipping scheduled appointments or sessions), and (v) data collection (i.e., a failure by the research team to comply with pre-specific trial guidelines for data collection and/or outcome evaluation due to avoidable reasons). Any such violations will be clearly reported.

Analyses undertaken will include:

(a) The primary analysis on pain interference will utilize the initial 2 by 2 by 2 structure comprising Stage (baseline, 10 weeks), randomized Treatment (AMBT, Placebo), and randomized number of Sessions (10, 18). This comparison will be extended to compare 10AMG and 10PTG after 10 sessions, and to compare 18AMG and 18PTG after 10 sessions.

(b) An analysis of pain interference will utilize the 2 by 2 by 3 structure comprising randomised Treatment (AMBT, Placebo), randomized number of Sessions (10, 18), and Stage (baseline, endline, follow-up).

(c) An assessment of change in pain interference, both within and between groups, for 18AMG and 18PTG, between the end of session 10 and endline.

(d) The four randomized arms compared against one another on pain interference at each of baseline, after session 10, endline, and follow-up.

For peer review only - http://bmjopen.bmj.com/site/about/guidelines.xhtml

(e) An age (16 – 24; 25 – 60) subgroup analysis using an age subgroup by treatment effect interaction. These analyses will be an extension of (a), (b), and (c) to incorporate age as a subgroup factor.

The same analyses would apply to the secondary outcome measures. The data will be represented descriptively and graphically by arm, and by treatment and age, and standardized effect sizes reported.

Linear mixed model with random intercepts will be used to analyze the data with, as appropriate, Treatment (AMBT, Placebo), number of Sessions (10, 18) and assessment stage as fixed factors. This approach avoids listwise deletion which would apply in a repeated measures ANOVA approach. The linear mixed modelling approach uses all of the available data and adheres to ITT. As a secondary analysis we will used multiple imputation chained equations (MICE) to impute outcome data[57 58] and use these imputed data sets in the linear mixed model. Pairwise comparisons will follow the same procedure. An assessment of the sensitivity of findings to missing data mechanisms will be undertaken.[59 60] Specifically, we will use the strategy discussed by Morris and colleagues[60] whereby multiply imputed values are altered by a randomised group dependent value prior to analysis. We will run this sensitivity analysis over a factorial arrangement of group dependent values constraining any altered imputed values to be in the valid response range. The instance of the group dependent values both being equal to zero will correspond to multiple imputation under MAR and non-zero values would reflect deviations away from this assumption. The analysis plan would apply to the primary outcome measure and secondary outcome measures.

Similar to former studies[25 27] the Reliable Change Index (RCI)[61] will be used to assess primary and secondary outcome measures for clinically significant changes. Here, a clinically significant result is a post-treatment score falling outside two standard deviations of the mean population of interest (in this study, two standard deviations outside pre-treatment means). In

For peer review only - http://bmjopen.bmj.com/site/about/guidelines.xhtml

contrast to tests of statistical significance comparing group means, tests of clinical significance explore effects of treatment on the individual.[62]

For visual-probe data, and as per former research[19 27], practice and incorrect trials will be removed prior to analyses. Box and whisker plots for overall data will be used to reveal overall outliers, which will be removed. Following this, mean response times will be computed for each participant, with any response >3SD from their individual mean also removed as outliers. This process ensures extremely quick or slow responses do not unduly bias the results, which are typically removed when cleaning and screening visual-probe data in pain-related research (e.g., [18 63 64 65 66]). An attentional bias index will then be computed for each stimulus and presentation time condition using the following equation: (TuPl − TlPl)+(TlPu −TuPu))/2. Here T is the threatening stimulus, P is the probe, u is the upper position, and l is the lower position. The attention capturing quality of threatening stimuli is measured by subtracting the mean probe detection time for congruent trials from the mean probe detection time of incongruent trials.[21]

Split-half reliability will be computed via a bootstrap procedure, by randomly splitting the total number of trials in two halves, such that each half has the same number of congruent and incongruent trials. Attentional bias scores will be computed for each half and the correlation between them calculated across participants. This procedure will be repeated 100 times, and an average split-half correlation computed.

Qualitative Analyses

Qualitative analysis will be used to detail participants' experiences completing the iABMT intervention. Specifically, thematic analysis using inductive coding will be used to identify themes present in the data obtained from the semi-structured interviews and the two open-ended questions included in the Satisfaction with the Online Training questionnaire. Guidelines provided by Braun and Clarke[67] will be followed. Following transcription of all interviews verbatim, initial codes will be generated. Potential themes and sub-themes will then be identified from the initial coding, which subsequently will be reviewed and refined. The

For peer review only - http://bmjopen.bmj.com/site/about/guidelines.xhtml

validity of the themes in relation to the data set will also be considered. Each theme and sub-theme will then be named and defined, and a thematic map of the data finalized. Examples from the data will be illustrated for each theme and sub-theme in the final report.

Ethics and Dissemination

This study has been approved by the University of Southampton Research Ethics Committee (ERGO ID: 26486), and the study protocol registered with ClinicalTrial.gov (NCT02232100). Ethical approval will be sought before any protocol modifications are made and the study protocol will be updated at ClinicalTrial.gov. Participants will provide informed consent prior to taking part in the study. All data will remain confidential and stored on password-protected systems and databases. Lifeguide and iSurvey servers both use HTTPS connections for security purposes. All data will be anonymised prior to dissemination, and personally identifiable information not known to anyone other than the researchers. Participants may withdraw from the intervention at any point by contacting the researchers via email or telephone, or by simply opting not to log into their iABMT account for their scheduled sessions. Participants are also encouraged to contact the researchers should they experience any distress or discomfort completing the intervention. As per University policy, any adverse events, harms or complaints arising from participation in the intervention will be reported to the University of Southampton Research Governance Office. Findings will be published in the most relevant, high-impact peer-reviewed journals and presented at relevant conferences. Lay reports will be written for interested pain charities and patient support groups.

For peer review only

List of figures and supplementary files

Table 1: Schedule of enrolment, intervention, and assessments.

Figure 1. A graphical representation of a typical visual-probe task training trial with linguistic stimuli.

Figure 2. Flow of the visual-probe task sessions for the 10-week and 18-week groups in the study

Figure 3. Examples of Lifeguide webpages included in the iABMT intervention.

Supplementary Material 1 – Stimuli development and properties

Supplementary Material 2 – Outcome Measures

Supplementary Material 3 – Think Aloud Study

For peer review only

For peer review only - http://bmjopen.bmj.com/site/about/guidelines.xhtml

References

1. Sambrook P, Taylor T, Schrieber L, et al. *The Musculoskeletal System: Basic Science and Clinical Conditions*. Second ed: Churchill Livingstone, Elsevier, 2010.

2. Cimmino MA, Ferrone C, Cutolo M. Epidemiology of chronic musculoskeletal pain. Best Practice & Research Clinical Rheumatology 2011;**25**(2):173-83 doi: 10.1016/j.berh.2010.01.012.

3. King S, Chambers CT, Huguet A, et al. The epidemiology of chronic pain in children and adolescents revisited: a systematic review. Pain 2011;**152**(12):2729-38 doi: 10.1016/j.pain.2011.07.016.

4. Tüzün EH. Quality of life in chronic musculoskeletal pain. Best Practice & Research Clinical Rheumatology 2007;**21**(3):567-79 doi: http://dx.doi.org/10.1016/j.berh.2007.03.001.

5. Breivik H, Collett B, Ventafridda V, et al. Survey of chronic pain in Europe: prevalence, impact on daily life, and treatment. Eur J Pain 2006;**10**(4):287-333 doi: 10.1016/j.ejpain.2005.06.009.

6. Bair MJ, Wu J, Damush TM, et al. Association of depression and anxiety alone and in combination with chronic musculoskeletal pain in primary care patients. Psychosom Med 2008;**70**(8):890-7 doi: 10.1097/PSY.0b013e318185c510.

7. Blyth FM, Noguchi N. Chronic musculoskeletal pain and its impact on older people. Best Practice & Research Clinical Rheumatology 2017;**31**(2):160-68 doi: 10.1016/j.berh.2017.10.004.

8. Harrison L, Wilson S, Munafò MR. Exploring the associations between sleep problems and chronic musculoskeletal pain in adolescents: a prospective cohort study. Pain research and management 2014;**19**(5):e139-e45 doi: 10.1155/2014/615203.

9. Flatø B, Aasland A, Vandvik I, et al. Outcome and predictive factors in children with chronic idiopathic musculoskeletal pain. Clinical and Experimental Rheumatology 1997;**15**(5):569-77.

For peer review only

10. Gold JI, Mahrer NE, Yee J, et al. Pain, fatigue and health-related quality of life in children and adolescents with chronic pain. Clinical Journal of Pain 2009;**25**(5):407 doi: 10.1097/AJP.0b013e318192bfb1.

11. Vinall J, Pavlova M, Asmundson GJ, et al. Mental health comorbidities in pediatric chronic pain: A narrative review of epidemiology, models, neurobiological mechanisms and treatment. Children 2016;**3**(4):40 doi: 10.3390/children3040040.

12. Pincus T, Morley S. Cognitive-processing bias in chronic pain: a review and integration. Psychol Bull 2001;**127**(5):599 - 617 doi: 10.1037/0033-2909.127.5.599.

13. Todd J, Sharpe L, Johnson A, et al. Towards a new model of attentional biases in the development, maintenance, and management of pain. Pain 2015;**156**(9):5189-1600 doi: 10.1097/j.pain.0000000000000214.

14. Vlaeyen JWS, & Linton, S. J. Fear-avoidance and its consequences in chronic musculoskeletal pain: a state of the art. Pain 2000;**85**(3):317 - 32.

15. Liossi C, Schoth DE, Bradley BP, et al. Time-course of attentional bias for pain-related cues in chronic daily headache sufferers. European Journal of Pain 2009;**13**(9):963-69 doi: http://dx.doi.org/10.1016/j.ejpain.2008.11.007.

16. Haggman SP, Sharpe, L. A., Nicholas, M. K., & Refshauge, K. M. Attentional biases toward sensory pain words in acute and chronic pain patients. The Journal of Pain 2010;**11**(11):1136 - 45.

17. Sharpe L, Dear BF, Schrieber L. Attentional biases in chronic pain associated with rhumatoid arthritis: Hypervigilance or difficulties disengaging? J Pain 2009;**10**(3):329 - 35 doi: 10.1016/j.jpain.2008.10.005.

18. Schoth DE, Beaney R, Broadbent P, et al. Attentional, interpretation and memory biases for sensory-pain words in individuals with chronic headache. British Journal of Pain 2019;**13**(1):22 - 31 doi: 10.1177/2049463718789445.

For peer review only - http://bmjopen.bmj.com/site/about/guidelines.xhtml

19. Schoth D, Liossi C. Specificity and time-course of attentional bias in chronic headache: a visual-probe investigation. Clinical Journal of Pain 2013;**29**(7):583-90 doi: 10.1097/AJP.0b013e31826b4849.

20. Schoth DE, Liossi C. Attentional bias towards pictorial representations of pain in individuals with chronic headache. Clin J Pain 2010;**26**(3):244 - 50 doi: 10.1097/AJP.0b013e3181bed0f9.

21. Schoth DE, Delgado Nunes V, Liossi C. Attentional bias towards pain-related information in chronic pain; a meta-analysis of visual-probe investigations. Clin Psychol Rev 2012;**32**(1):13 - 25 doi: 10.1016/j.cpr.2011.09.004.

22. Todd J, van Ryckeghem DM, Sharpe L, et al. Attentional bias to pain-related information: A meta-analysis of dot-probe studies. Health Psychol Rev 2018;**12**(4):419-36.

23. Crombez G, Van Ryckeghem D, Eccleston C, et al. Attentional bias to pain-related information: a meta-analysis. Pain 2013;**154**(4):497-510 doi: 10.1016/j.pain.2012.11.013.

24. MacLeod C, Mathews, A., & Tata, P. Attentional Bias in Emotional Disorders. Journal of Abnormal Psychology 1986;**95**(1):15 - 20.

25. Carleton RN, Richter AA, Asmundson GJG. Attention Modification in Persons with Fibromyalgia: A Double Blind, Randomized Clinical Trial. Cogn Behav Ther 2011;**40**(4):279-90 doi: 10.1080/16506073.2011.616218.

26. Sharpe L, Ianiello M, Dear BF, et al. Is there a potential role for attention bias modification in pain patients? Results of 2 randomised, controlled trials. Pain 2012;**153**(3):722-31 doi: 10.1016/j.pain.2011.12.014.

27. Schoth DE, Georgallis T, Liossi C. Attentional bias modification in people with chronic pain: a proof of concept study. Cognitive Behaviour Therapy 2013;**42**(3):233-43.

28. Curriculum for a CCT in Anaesthetics (2010). Secondary Curriculum for a CCT in Anaesthetics (2010). https://www.rcoa.ac.uk/document-store/curriculum-cct-anaesthetics-2010.

For peer review only - http://bmjopen.bmj.com/site/about/guidelines.xhtml

29. Heathcote LC, Jacobs K, Van Ryckeghem DML, et al. Attention bias modification training for adolescents with chronic pain: a randomized placebo-controlled trial. Pain 2018;**159**(2):239 - 51 doi: 10.1097/j.pain.0000000000001084.

30. Jones EB, Sharpe L. Cognitive bias modification: A review of meta-analyses. J Affect Disord 2017;**223**:175-83 doi: 10.1016/j.jad.2017.07.034.

31. Sawyer SM, Azzopardi PS, Wickremarathne D, et al. The age of adolescence. The Lancet Child & Adolescent Health 2018;**2**(3):223-28 doi: 10.1016/s2352-4642(18)30022-1.

32. Ofcom. Adults: Media use and attitudes report 2019, 2019.

33. Hamilton F. Perspectives of individuals with chronic musculoskeletal pain towards an online attentional bias modification training programme. University of Southampton, 2015.

34. Hakamata Y, Lissek, S., Bar-Haim, Y., Britton, J.C., Fox, N.A., Leibenluft, E., Ernst, M., & Pine, D. S. Attention bias modification treatment: A meta-analysis towards the establishment of novel treatment for anxiety. Biol Psychiatry 2010;**68**:982-90 doi: 10.1016/j.biopsych.2010.07.021.

35. Mogoaşe C, David D, Koster EH. Clinical efficacy of attentional bias modification procedures: An updated meta-analysis. J Clin Psychol 2014;**70**(12):1133-57 doi: 10.1002/jclp.2208.

36. Williams S, Yardley L, Wills GB. A qualitative case study of LifeGuide: users' experiences of software for developing Internet-based behaviour change interventions. Health Informatics J 2013;**19**(1):61-75 doi: 10.1177/1460458212458915.

37. Cleeland CS, Ryan KM. Pain assessment: global use of the Brief Pain Inventory. Ann Acad Med Singapore 1994;**23**(2):129-38.

38. Kahneman D. *Attention and effort*. New Jersey: Prentice Hall, 1973.

39. Broadbent D. *Perception and communication*. Oxford: Pergamon, 1958.

40. Thomas M, Sing H, Belenky G, et al. Neural basis of alertness and cognitive performance impairments during sleepiness. I. Effects of 24 h of sleep deprivation on waking human

For peer review only

regional brain activity. Journal of Sleep Research 2000;**9**(4):335-52 doi: 10.1046/j.1365-2869.2000.00225.x.

41. Tzambazis K, Stough C. Alcohol impairs speed of information processing and simple and choice reaction time and differentially impairs higher-order cognitive abilities. Alcohol and Alcoholism 2000;**35**(2):197-201 doi: 10.1093/alcalc/35.2.197.

42. Reissig CJ, Strain EC, Griffiths RR. Caffeinated energy drinks—a growing problem. Drug and Alcohol Dependence 2009;**99**(1):1-10 doi: 10.1093/alcalc/35.2.197.

43. Zwyghuizen-Doorenbos A, Roehrs TA, Lipschutz L, et al. Effects of caffeine on alertness. Psychopharmacology 1990;**100**(1):36-39 doi: 10.1007/BF02245786.

44. Müller K, Libuda L, Terschlüsen AM, et al. A review of the effects of lunch: On adults' short-term cognitive functioning. Canadian Journal of Dietetic Practice and Research 2013;**74**(4):181-88 doi: 10.3148/74.4.2013.181.

45. Piech RM, Pastorino MT, Zald DH. All I saw was the cake. Hunger effects on attentional capture by visual food cues. Appetite 2010;**54**(3):579-82 doi: 10.1016/j.appet.2009.11.003.

46. Al-Shawaf L. The evolutionary psychology of hunger. Appetite 2016;**105**:591-95 doi: 10.1016/j.appet.2016.06.021.

47. Schoth DE, Liossi C. Specificity and time-course of attentional bias in chronic headache: a visual-probe investigation. Clin J Pain 2013;**29**(7):583-90 doi: 10.1097/AJP.0b013e31826b4849.

48. Cleeland CS. The brief pain inventory: user guide. Houston, TX: The University of Texas MD Anderson Cancer Center 2009:1-11.

49. Dworkin RH, Turk DC, Wyrwich KW, et al. Interpreting the clinical importance of treatment outcomes in chronic pain clinical trials: IMMPACT recommendations. J Pain 2008;**9**(2):105-21.

For peer review only

50. Spielberger CD, Gorsuch RL, Lushene RE. *State Trait Anxiety Inventory*. Palo Alto, California: Consulting Psychologists Press, 1970.

51. Zigmond AS, Snaith RP. The hospital anxiety and depression scale. Acta Psychiatr Scand 1983;**67**(6):361-70.

52. McNeil DW, Rainwater A. Development of the Fear of Pain Questionnaire-III. Journal of Behavioral Medicine 1998;**21**(4):389-410 doi: 10.1023/A:1018782831217.

53. Hays RD, Stewart AL. Sleep Measures. Measuring functioning and well-being. In: Steweart AL, JEJ W, eds. Measuring function and well-being; the medical outcome study approach. Duke: Duke University Press, 1992:235-59.

54. Richards D, Timulak L. Satisfaction with therapist-delivered vs. self-administered online cognitive behavioural treatments for depression symptoms in college students. British Journal of Guidance & Counselling 2013;**41**(2):193-207 doi: 10.1080/03069885.2012.726347.

55. Gupta SK. Intention-to-treat concept: a review. Perspectives in Clinical Research 2011;**2**(3):109 doi: 10.4103/2229-3485.83221.

56. Sweetman EA, Doig GS. Failure to report protocol violations in clinical trials: a threat to internal validity? Trials 2011;**12**(1):214.

57. Chan A-W, Tetzlaff JM, Gøtzsche PC, et al. SPIRIT 2013 explanation and elaboration: guidance for protocols of clinical trials. BMJ 2013;**346**:e7586 doi: 10.1136/bmj.e7586.

58. Groenwold RH, Donders ART, Roes KC, et al. Dealing with missing outcome data in randomized trials and observational studies. American Journal of Epidemiology 2012;**175**(3):210-17 doi: 10.1093/aje/kwr302.

59. White IR, Horton NJ, Carpenter J, et al. Strategy for intention to treat analysis in randomised trials with missing outcome data. BMJ 2011;**342**:d40.

60. Morris TP, Kahan BC, White IR. Choosing sensitivity analyses for randomised trials: principles. BMC Med Res Methodol 2014;**14**(1):11.

For peer review only

61. Jacobson NS, Truax P. Clinical significance: A statistical approach to defining meaningful change. Psychotherapy Research 1991;**59**:12-19 doi: 10.1037/0022-006X.59.1.12.

62. Zahra D, Hedge C. The reliable change index: Why isn't it more popular in academic psychology? Psychology Postgraduate Affairs Group Quarterly 2010;**76**(76):14-19.

63. Schoth DE, Parry L, Liossi C. Combined cognitive biases for sensory-pain and disability information in individuals with chronic headache. J Health Psychol 2018;**23**(12):1610-21 doi: 10.1177/1359105316664136.

64. Dear BF, Sharpe L, Nicholas M, et al. Pain-related attentional biases: The importance of the personal relevance and ecological validity of stimuli. J Pain 2011a;**12**(6):625-32.

65. Schoth DE, Williams S, Liossi C. Attentional bias for pain- and period-related symptom words in healthy women who experienced a recent painful period. Eur J Pain 2015;**19**(6):745-51 doi: 10.1002/ejp.597.

66. McDermott MJ, Peck KR, Walters AB, et al. Do episodic migraineurs selectively attend to headache-related visual stimuli? Headache: The Journal of Head and Face Pain 2013;**53**(2):356-64 doi: 10.1111/head.12011.

67. Braun V, Clarke V. Using thematic analysis in psychology. Qualitative Research in Psychology 2006;**3**(2):77-101 doi: 10.1191/1478088706qp063oa.

For peer review only

Authors' contributions: All authors have contributed to the development of the study protocol. CL, TG and DES conceived the project. CL and DES registered the trial and are responsible for recruitment and implementation of the protocol. TG was primary responsible for the development of the online materials, including development and validation of linguistic and pictorial stimuli used in the visual-probe tasks. TG also assisted in programming the Lifeguide components. JZ was responsible for programming the visual-probe tasks to be used in the intervention, and assisted in programming the Lifeguide components. FH conducted the think aloud study. PW was responsible for development of the statistical analysis plan. All authors contributed to the writing of this manuscript, and approve its final form. These same authors will be included on future publications presenting intervention results, and will have access to the cleaned study datasets. **Funding**: This work was supported by the Economic and Social Research Council (grant number: RES-000-22-4128). This funding source had no role in the design of this study and will not have any role during its execution, analyses, interpretation of the data, or decision to submit results. **Competing interests**: The authors declare that they have no competing interest.

For peer review only

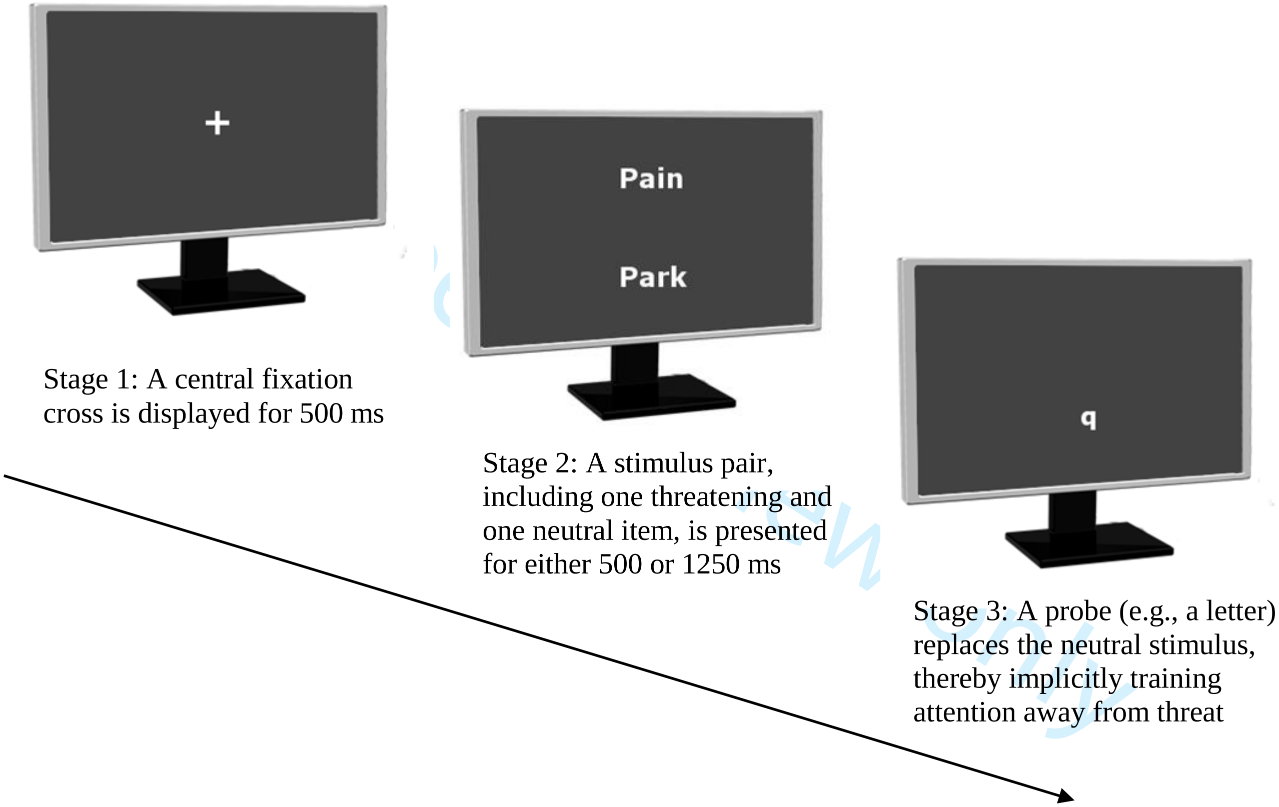

Figure 1. A graphical representation of a typical visual-probe task training trial with linguistic stimuli.

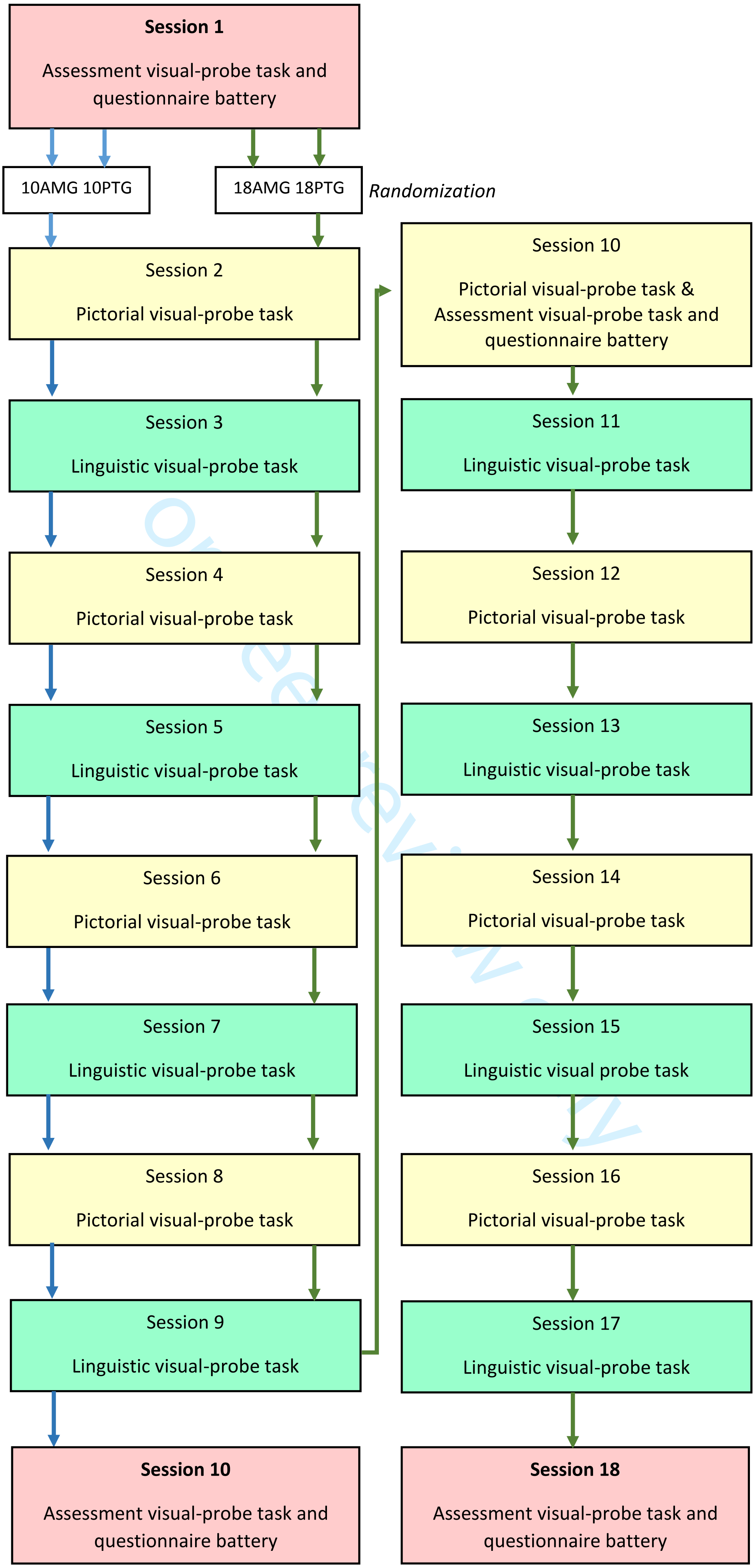

Figure 2. Flow of the visual-probe task sessions for the 10-week and 18-week groups in the study.

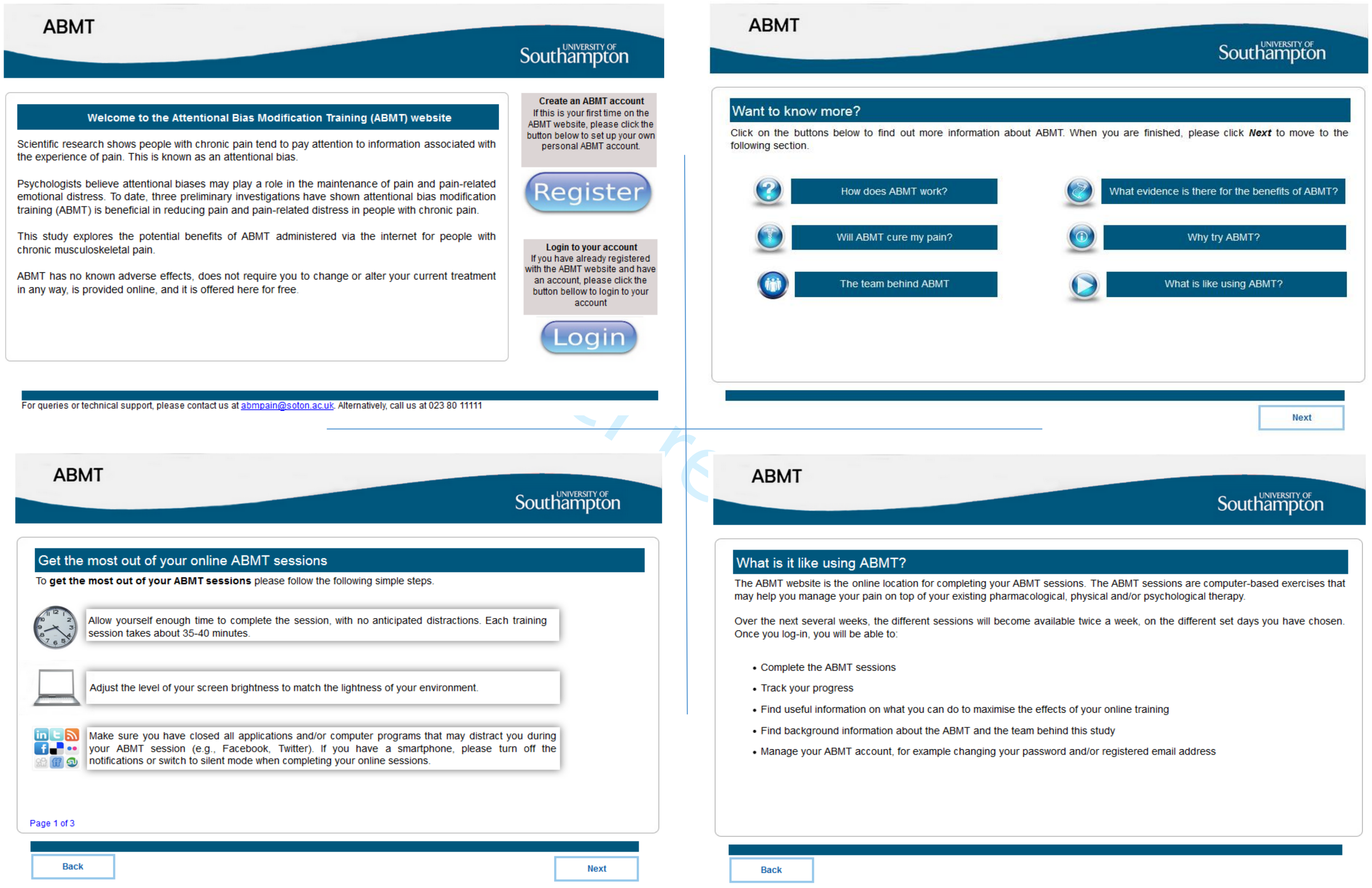

Figure 3. Examples of Lifeguide webpages included in the iABMT intervention.

Supplementary Material 1

Stimuli Development and Properties

A detailed description of the stimuli to be used in the iAMBT intervention is presented in this document. Linguistic stimulus properties and analyses on written frequency, valence, and arousal are first presented, followed by a description of pictorial stimuli properties and analyses of low-level features, valence, arousal and pain intensity. Following this, comparisons of stimuli properties between visual-probe sessions are then presented for linguistic and pictorial stimuli.

Linguistic stimuli

Properties. Linguistic and pictorial versions of the visual-probe task will be used. Each linguistic visual-probe task will include 32 threatening/neutral and 16 neutral/neutral word pairs. Threatening words stem from the following categories, with 8 words from each included in each visual-probe task: (i) sensory-pain words reflecting the sensory dimension of pain (e.g., *shooting*; *burning*; *aching*); (ii) affective-pain words reflecting the unpleasantness of pain and negative emotions associated with pain (e.g., *hopeless*, *punishing*, *tormenting*); (iii) health-threat words reflecting threat to health and well-being (e.g., *hospital*, *doctor*, *stress*); and (iv) general-threat words reflecting physical and emotional sources of threat, including manmade threat (e.g., *gun*), natural threat (e.g., *fire*), social threat (e.g., *humiliation*), and emotional threat (e.g., *sad*). Neutral words (including the neutral counterparts of the threatening words) include non-threatening words unrelated to pain or suffering (e.g., *version*; *rector*; *monument*), which were matched with their threatening counterparts on Kucera-Francis written frequency of use in the English language[1] and word length. Matching on written frequency minimise the chances of participants preferentially processing one stimulus due to its high frequency of use (e.g., faster recognition) when displayed simultaneously with another stimulus[2 3], while matching word length also minimise the chances of participants attending towards a stimulus due to its size.

In total, 864 words have been collected from previous studies exploring and modifying biases in pain and/or anxiety (i.e.,[4-8]), including words from the McGill Pain Questionnaire.[9]

For peer review only - http://bmjopen.bmj.com/site/about/guidelines.xhtml

Matching of threatening and neutral words was achieved via the MRC Psycholinguistic database[10], an online psycholinguistic tool that generates words with specific lexical characteristics. The database includes a dictionary of 150,837 English words providing syntactic, semantic and orthographical properties of each word.[11] Valence and arousal can influence attentional allocation to external stimuli.[12 13] A computerised version of the SAM task[14] was therefore used to collect ratings on valence and arousal for each word in a sample of sixteen healthy, pain-free participants (M_{age} = 25.9, SD = 4.08, range: 20 – 33 years; 8 female). All words were randomly presented to participants for 3 seconds each. Following each word, two 9-point SAM scales were presented, one for valence (1 = low pleasure to 9 = high pleasure) and one for arousal (1 = low arousal to 9 = high arousal). As per former chronic pain attentional bias research (e.g.,[5 15 16]), stimuli categories were assessed but not matched on valence and arousal, as differences are expected to exist between categories.

Analyses and results: written frequency, valence, and arousal. Analyses comparing properties between the five word categories are presented here, while analyses comparing word properties between the nine linguistic visual-probe tasks are presented below. One-way ANOVA was conducted comparing Kucera-Francis written frequency (Table 1) across words in the five word categories, revealing no significant main effect, $F(4, 853) = 1.97$, $p = .10$.

A repeated-measures ANOVA was conducted comparing arousal ratings (Table 2) across the five word categories, revealing a significant main effect, $F(2, 25) = 18.78$, $p < .001$, $\eta p^2 = .56$. Pairwise comparisons revealed the neutral category had significantly lower arousal scores than sensory-pain (*Md* [mean difference] = 1.82, *SE* = 0.45, *p* = .001), affective-pain (*Md* = 2.36, *SE*= 0.46, *p* = .001), general-threat (*Md* = 2.20, *SE* = 0.47, *p* = .003) and health-threat (*Md* = 2.05, *SE* = 0.35, *p* < .001) categories. Statistically significant differences were not found between the arousal scores of the words included in the four threat-related categories.

For peer review only - http://bmjopen.bmj.com/site/about/guidelines.xhtml

Table 1

Mean Kucera-Francis Written Frequency per Word Category

Word category	*M*	*SD*
Sensory-pain	19.07	27.01
Affective-pain	17.28	20.63
General-threat	25.00	32.10
Health-threat	21.36	36.39
All neutral words	22.60	27.97

Table 2

Mean Arousal and Valence Ratings per Word Category

	Arousal		Valence	
Word Category	*M*	*SD*	*M*	*SD*
Sensory-pain	3.80	1.85	3.95	0.83
Affective-pain	4.34	1.64	3.29	0.73
General-threat	4.19	1.78	3.35	0.79
Health-threat	4.03	1.32	3.48	0.68
Sensory-pain neutral counterparts	2.18	1.28	5.33	0.41
Affective-pain neutral counterparts	2.19	1.32	5.29	0.29
General-threat neutral counterparts	2.06	1.28	5.24	0.33
Health-threat neutral counterparts	2.05	1.19	5.19	0.36
Neutral/Neutral	1.95	1.32	5.29	0.33

A repeated-measures ANOVA was conducted comparing valence ratings (Table 2) across the five word categories, revealing a significant main effect, $F(2, 29) = 52.00$, $p < .001$, $\eta p^2 = .78$. Pairwise comparisons showed the neutral category had significantly higher pleasantness scores than sensory-pain (*Md* = 1.33, *SE* = 0.23, *p* <. 001) affective-pain (*Md* = 1.99, *SE* = 0.22, *p* < .001), general-threat (*Md* = 1.93, *SE* = 0.23, *p* <. 001) and health-threat (*Md* = 1.80, *SE* = 0.32, *p* < .001) categories. The words in the sensory-pain category were rated as more pleasant than

For peer review only - http://bmjopen.bmj.com/site/about/guidelines.xhtml

words included in the affective-pain (Md = 0.66, SE = 0.11, p < .001), general-threat (Md = 0.60, SE = 0.11, p < .001) and health-threat (Md = 0.47, SE = 0.11, p = .01) categories.

Pictorial Stimuli

Properties. Pictorial visual-probe tasks each include 32 threatening/neutral and 16 neutral/neutral image pairs. Threatening images stem from the following categories: (i) musculoskeletal pain images depicting people experiencing pain in muscles and/or joints, including images of people performing daily activities that can be considered painful and/or harmful for individuals with CMP (e.g., a person running; a person lifting a heavy weight with poor posture); (ii) facial expressions of pain, featuring a single model depicting a prototypical pain expression – closing the eyes or narrowing the eyelids, lowering the eyebrows, wrinkling the nose or raising the cheek, and partly opening the mouth or extending the lips[17], including horizontal lip stretching and/or oblique lip raising[18]; (iii) health-threat images reflecting threat to health and well-being (e.g., medication, wheelchair); and (iv) general-threat images reflecting physical and emotional sources of threat, including manmade threat (e.g., gun), natural threat (e.g., flood), and social threat (e.g., social isolation). Neutral images (including the neutral pairs of the threatening images) depict non-threatening objects (e.g., a car) and people performing every-day activities (e.g., a person reading a newspaper). In total, 768 images have been collected from previous studies exploring and modifying attentional biases in chronic pain (i.e.,[5] [19]), including online databases providing batteries of pictorial stimuli for research (i.e., International Affective Picture System database[20]; The Photograph Series of Daily Activities Short Version[21]; NimStim Set of Facial Expressions[22]).

Each threatening image depicting a person was paired with a neutral image featuring the same person, with the same clothes and background. Each threatening image depicting an object was matched with a neutral image depicting a similar, non-threatening object. Image pairs in the neutral/neutral condition were matched in the same manner. An equal number of images depicting male and female models were used within each image category. All images were full-

For peer review only - http://bmjopen.bmj.com/site/about/guidelines.xhtml

colour and the same size (i.e., 265 pixels wide x 250 pixels high). Each threatening image was matched with its neutral counterpart on complexity, luminance, contrast level, and colour saturation in blue, red and green channels via visual inspection[23]. Visual attention and gaze fixations are influenced by bottom-up variables, including the saliency of objects[24] and low-level features of visual scenes.[25 26] The additional matching rules for pictorial stimuli in the present study were therefore used to minimise the chances of participants preferentially processing one stimulus due to unnecessary variations in content. Using Adobe Photoshop CS6 Extended, the means and standard deviations for all parameters were extracted for evaluation. Luminance values range from 0 to 255, where 0 indicates no light intensity (i.e., no light emission; black) and 255 indicates the highest light intensity (i.e., absolute light emission; white). Contrast levels values range from -100 to +100, with values closer to +100 indicating more contrasted image. The intensity values for red, green and blue colours range from 0 to 255, where 0 indicates black (i.e., darkest extreme) and 255 white (i.e., lightest extreme) for each colour dimension.

Visual complexity was examined as the size of each compressed image file (.jpeg) in kilobytes under the assumption that more complex images would have higher values in kilobytes.[27] As with linguistic stimuli, a computerised version of the SAM task[14] was used to collect ratings on valence and arousal for each image. Musculoskeletal and facial expressions of pain categories were also evaluated on pain intensity using a 10 cm Visual Analogue Scale (VAS). Participants were required to rate the pain that the person in the image experienced. The scale ranged from 0 to 10, where 0 indicated '*no pain at all*' and 10 indicated '*excruciating pain*'. This rating aimed to assess the degree to which images included in the pain-related categories were perceived as depicting pain. Sixteen healthy, pain-free participants (M_{age} = 24.8, SD = 4.56, range: 20 – 33 years; 8 female) were recruited to provide ratings on valence, arousal, and pain intensity (this was a separate sample to that recruited to provide linguistic stimuli ratings).

Analyses and results: Low-level features. Analyses comparing properties between the five image categories on low-level features (Table 3) are presented here, while analyses

For peer review only - http://bmjopen.bmj.com/site/about/guidelines.xhtml

comparing word properties between the eight pictorial visual-probe tasks are provided below. Considering low-level features, one-way ANOVAs revealed no significant main effects for complexity, $F(4,763) = 2.18$, $p = .70$, or luminance, $F(4,763) = 2.31$, $p = .06$. A significant main effect was found for red saturation, $F(4,763) = 5.19$, $p < .001$, $\eta p^2 = .03$. Pairwise comparisons revealed images in the musculoskeletal-pain category had significantly less red saturation than images in the facial expressions of pain (*Md* = 24.24, *SE* = 7.89, $p = .022$) and neutral categories (*Md* = 19.04, *SE* = 5.91, $p = .013$). Images in the general-threat category had significantly less red saturation than facial expressions of pain (*Md* = 23.96, *SE* = 7.89, $p = .025$) and neutral categories (*Md* = 18.76, *SE* = 5.91, $p = .016$). A significant main effect was found for blue saturation, $F(4,763) = 2.74$, $p = .03$, $\eta p^2 = .01$. Images in the general-threat categories had significantly less blue saturation than images in the neutral category (*Md* = 29.64, *SE* = 9.96, $p = .030$). A significant main effect was found for green saturation, $F(4, 763) = 4.19$, $p = .002$, $\eta p^2 = .02$. Images in the general-threat expressions category had significantly less green saturation than images in the neutral category (*Md* = 22.03, *SE* = 6.23, $p = .004$). A significant main effect was found for RMS contrast levels, $F(4,763) = 5.98$, $p < .001$, $\eta p^2 = .03$. Images in the facial expressions of pain category had significantly higher RMS contrast than the images included in musculoskeletal-pain (*Md* = 9.57, *SE* = 3.32, $p = .040$), health-threat (*Md* =13.77, *SE* = 3.32, $p < .001$) and neutral categories (*Md* = 10.89, *SE* = 2.49, $p < .001$).

For peer review only

Table 3

Mean Low-Level values per Image Category

	Musculoskeletal-pain		Facial Expressions of Pain		General-threat		Health-threat		All neutral stimuli	
	M	*SD*	*M*	*SD*	*M*	*SD*	*M*	*SD*	*M*	*SD*
Complexity	20.03	7.17	15.69	3.10	20.14	6.36	19.30	10.78	18.62	10.92
Red Saturation	148.72	41.35	172.95	22.79	148.99	43.43	164.37	57.07	167.76	45.41
Green Saturation	140.39	41.39	153.81	27.56	132.80	41.82	154.92	58.08	154.84	48.60
Blue Saturation	132.53	43.55	144.86	31.64	119.90	48.00	149.09	61.87	149.54	85.49
Luminance	142.01	41.10	158.57	25.50	136.21	41.39	157.37	57.10	162.79	92.44
RMS contrast level	61.25	15.3	70.82	18.75	64.22	18.95	57.00	20.91	59.93	18.86

Analyses and results: Valence, arousal and pain intensity. A repeated-measures ANOVA was conducted comparing arousal ratings (Table 4) across the five image categories, revealing a significant main effect, $F(2,23) = 32.79$, $p < .001$, $\eta p^2 = .69$. Pairwise comparisons revealed that the neutral category had significantly lower arousal scores than facial expressions of pain (*Md* = 2.24, *SE* = 0.45, *p* = .002), musculoskeletal-pain (*Md* = 1.28, *SE* = 0.33, *p* = .014), general-threat (*Md* = 2.86, *SE* = 0.38, *p* < .001), and health-threat (*Md* = 1.91, *SE* = 0.34, *p* = .001) categories. The general-threat category was significantly more arousing than facial expressions of pain (*Md* = 0.62, *SE* = 0.15, *p* = .010), musculoskeletal-pain (*Md* = 1.59, *SE* = 0.14, *p* < .001), and health-threat categories (*Md* = 0.95, SE =0.15, *p* < .001). The musculoskeletal-pain category was also significantly less arousing than the facial expressions of pain (*Md* = 0.96, *SE* = 0.15, *p* < .001) and the health-threat (*Md* = 0.64, *SE* = 0.14, *p* = .005) categories.

For peer review only - http://bmjopen.bmj.com/site/about/guidelines.xhtml

A repeated-measures ANOVA conduced on valence scores (Table 4) across the five image conditions revealed a significant main effect, $F(2,29) = 64.52$, $p < .001$, $\eta p^2 = .82$. Pairwise comparisons showed the neutral category had significantly higher pleasantness scores than facial expressions of pain (*Md* = 1.56, *SE* = 0.15, *p* <. 001), musculoskeletal-pain (*Md* = 0.86, *SE* = 0.13, *p* < .001), general-threat (*Md* = 1.69, *SE* = 0.16, *p*<. 001) and health-threat (*Md* = 1.27, *SE* = 0.18, *p*< .001) categories. Images in the musculoskeletal-pain category were rated significantly more pleasant than the images included in the facial expressions of pain (*Md* = 0.69, *SE* = 0.07, *p* < .001), general-threat (*Md* = 0.83, *SE* = 0.08, *p* < .001) and health-threat (*Md* = 0.41, *SE* = 0.09, *p*= .003) categories. Images in the health-threat category had significantly higher pleasantness scores than images included in the general-threat category (*Md* = 0.42, *SE* = 0.07, *p* < .001).

An independent samples *t*-test was conducted comparing pain intensity scores (Table 4) between the images in the musculoskeletal pain and facial expressions of pain categories. There was a significant difference in the pain intensity scores of musculoskeletal-pain (*M* = 3.35, *SD* = 2.27) and facial expressions of pain (*M* = 5.15, *SD* = 2.13) categories; $t(245) = 6.520$, $p < .001$, $r = 0.38$. This result suggests images included in the facial expressions of pain category had significantly higher pain intensity ratings than those included in the musculoskeletal-pain category.

For peer review only - http://bmjopen.bmj.com/site/about/guidelines.xhtml

Table 4

Mean Arousal, Valence and Pain Intensity Ratings per Image Category

	Arousal		Valence		Pain Intensity	
Image Category	*M*	*SD*	*M*	*SD*	*M*	*SD*
Musculoskeletal-pain	2.85	1.45	4.20	0.57	3.35	2.09
Facial expressions of pain	3.81	1.92	3.50	0.66	5.15	2.02
General-threat	4.43	1.69	3.37	0.62		
Health-threat	3.48	1.53	3.79	0.71		
Musculoskeletal-pain neutral counterparts	1.82	0.68	5.11	0.22		
Facial expressions of pain neutral counterparts	1.74	0.69	5.00	0.14		
General-threat neutral counterparts	1.82	0.68	5.08	0.17		
Health-threat neutral counterparts	1.75	0.54	5.04	0.24		
Neutral/Neutral	1.57	0.49	5.10	0.15		

For peer review only

Comparison between Visual-Probe Sessions – Linguistic Stimuli

Differences in Arousal Scores between Threatening Word Categories across the Nine Linguistic Visual Probe Sessions

A 4 (word category; sensory-pain, affective-pain, general-threat, health-threat) x 9 (linguistic visual probe session; session 1, 3, 5, 7, 9, 11, 13, 15, 17) ANOVA was conducted to compare the arousal scores of the threatening word categories across the 9 linguistic visual probe task sessions. The results showed a significant main effect of session, $F(8, 120) = 8.22$, $p < .001$, $\eta_p^2 = .35$, and a significant word category x session interaction, $F(24, 360) = 3.05$, $p < .001$, $\eta_p^2 = .17$.

Post-hoc analyses across sessions. To explore the significant interaction, a series of one-way ANOVAs with 9 levels (Session: 1, 3, 5, 7, 9, 11, 13, 15, 17) were run for each threatening word category independently. These explored whether the arousal ratings of each threatening word category differed across the sessions. For the sensory-pain words, a main effect of session was found, $F(5,67) = 6.02$, $p < .001$, $\eta_p^2 = .29$. Pairwise comparisons revealed sensory words to be significantly more arousing in session 1 than session 9 (*Md* = 0.92, *SE* = 0.22, *p* = .03), and significantly more arousing in session 17 than session 9 (*Md* = 0.75, *SE* = 0.17, *p* = .02) and session 15 (*Md* = 0.59, *SE* = 0.11, *p* = .003).

For the affective-pain words, a main effect of session was found, $F(8,120) = 4.26$, $p < .001$, $\eta_p^2 = .22$. Pairwise comparisons revealed these words to be significantly more arousing in session 1 than in session 5 (*Md* = 0.57, *SE* = 0.12, *p* = .01) and session 13 (*Md* = 1.04, *SE* = 0.26, *p* = .05).

For the general-threat words, a main effect of session was found, $F(8, 120) = 4.91$, $p < .001$, $\eta_p^2 = .25$. Pairwise comparisons revealed these words to be significantly more arousing in session 1 than session 5 (*Md* = 0.65, *SE* = 0.13, *p* = .01), session 7 (*Md* = 0.88, *SE* = 0.17, *p* = .004) and session 15 (*Md* = 0.84, *SE* = 0.19, *p* = .02).

For peer review only - http://bmjopen.bmj.com/site/about/guidelines.xhtml

For the health-threat words, a main effect of session was found, $F(8,120) = 4.08$, $p < .001$, $\eta_p^2 = .21$. Pairwise comparisons revealed these words to be significantly more arousing in session 1 than session 11 ($Md = 1.05$, $SE = 0.18$, $p = .001$).

Post-hoc analyses within sessions. To further explore the significant interaction, a series of one-way ANOVAs with 4 levels (word category; sensory-pain, affective-pain, general-threat and health-threat) were conducted for each session independently. These explored whether the arousal ratings of each threatening word category differed within each linguistic visual-probe session. No significant main effects of word category were found for session 1, $F(3,45) = 1.16$, $p =.20$, $\eta_p^2 = .10$, session 5, $F(3,45) = 2.34$, $p = .86$, $\eta_p^2 = .14$, session 13, $F(3,45) = 0.70$, $p = .06$, $\eta_p^2 = .05$, and session 17, $F(3,45) = 2.27$, $p = .09$, $\eta_p^2 = .13$.

A significant main effect of word category was found in session 3, $F(3,45) = 4.31$, $p = .01$, $\eta_p^2 = .22$. Pairwise comparisons revealed no significant differences between the word categories of this session, however.

A significant main effect of word category was found in session 7, $F(3,45) = 3.75$, $p = .02$, $\eta_p^2 = .20$. Pairwise comparisons revealed no significant differences between the four threat-related categories.

A significant main effect of word category was found in session 9, $F(3,45) = 5.22$, $p = .004$, $\eta_p^2 = .26$. Pairwise comparisons revealed that sensory-pain words were significantly less arousing than affective-pain ($Md = 0.84$, $SE = 0.21$, $p = .01$), general-threat ($Md = 0.77$, $SE = 0.23$, $p = .03$) and health-threat categories ($Md = 0.92$, $SE = 0.30$, $p = .05$).

A significant main effect of word category was found in session 11, $F(3,45) = 4.45$, $p = .01$, $\eta_p^2 = .23$. Pairwise comparisons showed that the general-threat words were significantly more arousing than the health-threat words ($Md = 0.61$, $SE = 0.19$, $p = .04$).

A significant main effect of word category was found in session 15, $F(3,45) = 2.86$, $p =.05$, $\eta_p^2 = .16$. Pairwise comparisons revealed no significant differences between the four threat-related categories.

For peer review only - http://bmjopen.bmj.com/site/about/guidelines.xhtml

For peer review only

Differences in Valence Scores between Threatening Word Categories across the nine Linguistic Visual Probe Sessions

A 4 (word category; sensory-pain, affective-pain, general-threat and health-threat) x 9 (linguistic visual probe sessions; 1, 3, 5, 7, 9, 11, 13, 15, and 17) ANOVA was conducted to compare the pleasantness scores of the threatening categories across the 9 linguistic visual probe task sessions. The results showed significant main effects of word category, $F(3,45) = 15.86$, $p < .001$, $\eta_p^2 = .51$, and session, $F(8,120) = 11.90$, $p < .001$, $\eta_p^2 = .44$, and a significant word category x session interaction, $F(24,360) = 2.57$, $p < .001$, $\eta_p^2 = .15$.

Post-hoc analyses across sessions. To explore the significant interaction, a series of one-way ANOVAS with 9 levels (Session; 1, 3, 5, 7, 9, 11, 13, 15, 17) were run for each word category independently. These explored whether the pleasantness ratings for each word category differed across the sessions. For the sensory-pain words, a main effect of session was found, $F(5,75) = 6.88$, $p< .001$, $\eta_p^2 = .31$. Pairwise comparisons revealed sensory words to be significantly less pleasant in session 1 than session 3 (*Md* = 0.49, *SE* = 0.12, $p =.04$), session 7 (*Md* = 0.59, *SE* = 0.11, $p = .003$) and session 9 (*Md* = 0.73, *SE* = 0.09, $p < .001$). Sensory-pain words were also significantly less pleasant in session 17 than session 5 (*Md* = 0.26, *SE* = 0.05, $p = .01$) and session 9 (*Md* = 0.49, *SE* = 0.09, $p = .003$).

For the affective-pain words, a main effect of session was found, $F(8,120) = 4.86$, $p< .001$, $\eta p^2 = .25$. Pairwise comparisons revealed these words to be significantly less pleasant in session 1 than session 5 (*Md* = 0.51, *SE* = 0.11, $p = .01$), session 7 (*Md* = 0.47, *SE* = 0.09, $p = .01$), session 13 (*Md* = 0.59, *SE* = 0.11, $p = .004$) and session 15 (*Md* = 0.44, *SE* = 0.11, $p = .04$).

For the general-threat words, a main effect of session was found, $F(8,120) = 9.64$, $p< .001$, $\eta p^2 = .39$. Pairwise comparisons revealed these words to be significantly less pleasant in session 1 than session 5 (*Md* = 0.63, *SE* = 0.09, $p < .001$), session 7 (*Md* = 0.65, *SE* = 0.11, $p = .001$), session 9 (*Md* = 0.44, *SE* = 0.07, $p < .001$), session 13 (*Md* = 0.33, *SE* = 0.06, $p = .001$) and session 15 (*Md* = 0.65, *SE* = 0.12, $p = .002$). Significantly less pleasant words were also found in

For peer review only - http://bmjopen.bmj.com/site/about/guidelines.xhtml

session 3 than session 5 (*Md* = 0.37, *SE* = 0.09, $p = .03$) and session 7 (*Md* = 0.39, *SE* = 0.08, $p = .01$). Session 17 included significantly less pleasant words than session 7 (*Md* = 0.41, *SE* = 0.10, $p = .03$).

For the health-threat words, a main effect of session was found, $F(4,58) = 2.90$, $p = .03$, $\eta_p^2 = .16$. Pairwise comparisons revealed these words to be significantly less pleasant in session 1 than session 11 (*Md* = 0.57, *SE* = 0.14, $p = .03$).

Post-hoc analyses within sessions. To further explore the significant interaction, one-way ANOVAs with 4 levels (word category; sensory-pain, affective-pain, general-threat and health-threat) were conducted for each session independently. These explored whether pleasantness ratings for each threatening word category differed within each session. For session 1, a significant main effect was found, $F(3,45) = 4.84$, $p = .01$, $\eta_p^2 = .24$. Pairwise comparisons revealed that sensory-pain words were significantly more pleasant than affective words (*Md* = 0.63, *SE* = 0.16, $p = .01$).

For session 3, a significant main effect was found, $F(3,45) = 12.71$, $p < .001$, $\eta_p^2 = .46$. Pairwise comparisons showed that sensory-pain words were significantly more pleasant than affective-pain (*Md* = 0.73, *SE* = .15, $p < .001$), general-threat (*Md* = 0.79, *SE* = 0.13, $p < .001$) and health-threat words (*Md* = 0.56, *SE* = 0.15, $p = .01$).

For session 5, a significant main effect was found, $F(3,45) = 6.45$, $p < .001$, $\eta_p^2 = .30$. Pairwise comparisons revealed that sensory-pain words were significantly more pleasant than the affective-pain words (*Md* = 0.62, *SE* = 0.15, $p = .01$).

For session 7, a significant main effect was found, $F(3,45) = 10.19$, $p < .001$, $\eta_p^2 = .41$. Pairwise comparisons revealed that sensory-pain words were significantly more pleasant than the affective-pain words (*Md* = 0.17, *SE* = 0.13, $p < .001$), the general-threat words (*Md* = 0.51, *SE* = 0.09, $p < .001$) and the health-threat words (*Md* = 0.55, *SE* = 0.16, $p = .02$).

For session 9, a significant main effect was found, $F(2,31) = 13.67$, $p < .001$, $\eta_p^2 = .48$. Pairwise comparisons revealed that the sensory-pain words were significantly more pleasant than

For peer review only - http://bmjopen.bmj.com/site/about/guidelines.xhtml

the affective-pain words (*Md* = 0.98, *SE*= 0.16, $p < .001$), the general-threat words (*Md* = 0.87, *SE* = 0.15, $p < .001$) and the health-threat words (*Md* = 0.92, *SE* = 0.21, $p < .001$).

For session 11, a significant main effect was found, $F(3,45) = 11.63$, $p < .001$, $\eta_p^2 = .44$. Pairwise comparisons revealed that the sensory-pain words were significantly more pleasant than the affective-pain (*Md* = 0.69, *SE* = 0.13, $p < .001$) and the general-threat words (*Md* = 0.70, *SE* = 0.17, $p < .001$). Also, the affective-pain words were significantly less pleasant than the health-threat words (*Md* = 9.39, *SE* = 0.13, $p = .04$).

For session 13, a significant main effect was found, $F(3,45) = 4.43$, $p = .01$, $\eta_p^2 = .23$. Pairwise comparisons revealed that the sensory-pain words were significantly more pleasant than the general-threat words (*Md* = 0.56, *SE* = 0.12, $p < .001$).

For session 15, a significant main effect was found, $F(3,45) = 8.40$, $p < .001$, $\eta_p^2 = .36$. Pairwise comparisons showed that the sensory-pain words were significantly more pleasant than the words included in the affective (*Md* = 0.61, *SE* = 0.12, $p < .001$) and the health-threat words (*Md* = 0.71, *SE* = 0.19, $p = .01$).

For session 17, a significant main effect was found, $F(3,45) = 6.78$, $p<.001$, $\eta_p^2 = 0.36$. $_p^2$ = 0.31. Pairwise comparisons revealed that the sensory-pain words were significantly more pleasant than affective-pain (*Md* = 0.56, *SE* = 0.15, $p = .01$) and the general-threat words (*Md* = 0.57, *SE* = 0.15, $p = .01$).

Comparison between Visual-Probe Sessions – Pictorial Stimuli

Differences in Arousal Scores between Threatening Image Categories across the eight Pictorial Visual Probe Sessions

A 4 (image category; musculoskeletal-pain, facial expressions of pain, general-threat, health-threat) x 8 (pictorial visual probe sessions; 2, 4, 6, 8, 10, 12, 14, 16) ANOVA was conducted to compare the arousal scores of the threatening image categories across the 8 pictorial visual probe task sessions. The results showed a significant main effect of image category, $F(3,45) = 33.52$, *p*

For peer review only

$< .001$, $\eta_p^2 = .69$, and a significant image category x session interaction, $F(7,98) = 9.84$, $p < .001$, $\eta_p^2 = .40$.

Post-hoc analyses within image categories. To explore the significant interaction, a series of one-way ANOVAs with 8 levels (Session; 2, 4, 6, 8, 10, 12, 14, 16) were run for each threatening image category independently. These explored whether the arousal ratings of each threat-related image category differed across the sessions. For the images in the musculoskeletal-pain category, no main effect of session was found, $F(2,31) = 2.49$, $p = .10$, $\eta_p^2 = .14$. For the images in the facial expressions of pain category, no main effect of session was found, $F(3,46) = 0.77$, $p = .50$, $\eta_p^2 = .05$.

For the images in the general-threat category, a main effect of session was found, $F(7, 105) = 7.40$, $p < .001$, $\eta_p^2 = .33$. Pairwise comparisons revealed these images to be significantly less arousing in session 4 than session 6 (*Md* = 0.70, *SE* = 0.17, *p* = .023), session 12 (*Md* = 0.73, *SE* = 0.19, *p* = .04) and session 16 (*Md* = 0.82, *SE* = 0.21, *p* = .04). These images were also found to be significantly less arousing in session 14 than session 6 (*Md* = 0.93, *SE* = 0.19, *p* = .006), session 12 (*Md* = 0.96, *SE* = 0.17, *p* = .001) and session 16 (*Md* = 1,05, *SE* = 0.17, *p* = .001).

For the images in the health-threat category, a main effect of session was found, $F(7, 105) = 13.10$, $p < .001$, $\eta_p^2 = .47$. Pairwise comparisons revealed these images to be significantly more arousing in session 10 than session 8 (*Md* = 1.15, *SE* = 0.20, *p* = .001) and session 12 (*Md* = 0.88, *SE* = 0.20, *p* = .02). Health-threat images were also found to be significantly less arousing in session 16 than session 2 (*Md* = 0.97, *SE* = 0.17, *p* = .001), session 4 (*Md* = 1.34, *SE* = 0.26, *p* = .004), session 6 (*Md* = 1.32, *SE* = 0.29, *p* = .009), session 10 (*Md* = 1.91, *SE* = 0.28, *p*< .001), session 12 (*Md* = 1.03, *SE* = 0.26, *p* = .04) and session 14 (*Md* = 1.45, *SE* = 0.9, *p*< .001).

Post-hoc analyses within sessions. To further explore the significant interaction, a series of one-way ANOVAs with 4 levels (image category; musculoskeletal-pain, facial expressions of pain, general-threat and health-threat) were conducted for each session independently. These

For peer review only - http://bmjopen.bmj.com/site/about/guidelines.xhtml

explored whether the arousal ratings of each threatening image category differed within each pictorial visual-probe session.

A significant main effect of image category was found in session 2, $F(3,45) = 23.27$, $p < .001$, $\eta_p^2 = .61$. Pairwise comparisons revealed that the images in the facial expressions of pain category were more arousing than those included in the musculoskeletal-pain category (Md = 0.98, SE = 0.27, $p = .014$). The images in the general-threat category were also found to be significantly more arousing than those included in the musculoskeletal-pain (Md = 2.05, SE = 0.32, $p < .001$), facial expressions of pain (Md = 1.07, SE = 0.19, $p < .001$) and health-threat categories (Md = 1.34, SE = 0.24, $p < .001$).

A significant main effect of image category was found in session 4, $F(3,45) = 8.27$, $p < .001$, $\eta_p^2 = .36$. Pairwise comparisons revealed that the images in the musculoskeletal-pain category were significantly less arousing than the images in the facial expressions of pain (Md = 0.76, SE = 0.16, $p = .001$), health-threat (Md = 1.10, SE = 0.23, $p = .002$) and general-threat categories (Md = 0.70, SE = 0.22, $p = .04$).

A significant main effect of image category was found in session 6, $F(3,45) = 26.77$, $p < .001$, $\eta_p^2 = .64$. Pairwise comparisons revealed that the images in the musculoskeletal-pain category were significantly less arousing than the images included in the facial expressions of pain (Md = 1.17, SE = 0.25, $p = .002$), general-threat (Md = 2.06, SE = 0.23, $p < .001$) and health-threat categories (Md = 0.97, SE = 0.25, $p = .009$). The images in the general-threat category were significantly more arousing than those in the facial expressions of pain (Md = 0.89, SE = 0.21, $p = .003$) and health-threat categories (Md = 1.09, SE = 0.21, $p = .001$).

A significant main effect of image category was also found in session 8, $F(2,32) = 10.56$, $p < .001$, $\eta_p^2 = .41$. Pairwise comparisons revealed that the images in the musculoskeletal-pain category were significantly less arousing than those included in the facial expressions of pain (Md = 0.89, SE = 0.22, $p = .006$) and general-threat categories (Md = 1.13, SE = 0.25, $p = .003$).

For peer review only - http://bmjopen.bmj.com/site/about/guidelines.xhtml

The images in general-threat categories were significantly more arousing that those included in the health-threat category (*Md* = 1.00, *SE* = 0.20, p = .001).

A significant main effect of image category was found in session 10, $F(3,45) = 17.37$, $p < .001$, $\eta_p^2 = .54$. Pairwise comparisons revealed that the images in the musculoskeletal-pain category were significantly less arousing than those included in the general-threat (*Md* = 0.92, *SE* = 0.17, $p < .001$) and health-threat categories (*Md* = 1.23, *SE* = 0.20, $p < .001$). The images in the facial expressions of pain category were also significantly less arousing than those included in the health-threat category (*Md* = 0.71, *SE* = 0.18, $p = .009$).

A significant main effect of image category was found in session 12, $F(3,45) = 18.40$, $p < .001$, $\eta_p^2 = .55$. Pairwise comparisons revealed that the images in the musculoskeletal-pain category were significantly less arousing that those included in the facial expressions of pain (*Md* = 1.61, *SE* = 0.18, $p = .022$) and general-threat categories (*Md* = 1.64, *SE* = 0.19, $p < .001$). The images included in the general-threat category were significantly more arousing than the images included in the facial expressions of pain (*Md* = 1.03, *SE* = 0.24, $p = .001$) and health-threat categories (*Md* = 1.03, *SE* = 0.26, $p < .001$).

A significant main effect of image category was found in session 14, $F(3,45) = 14.72$, $p < .001$, $\eta_p^2 = .50$. Pairwise comparisons revealed than the images in the musculoskeletal-pain category were significantly less arousing than those included in facial expressions of pain (*Md* = 1.39, *SE* = 0.30, $p = .002$), general-threat (*Md* = 1.41, *SE* = 0.25, $p < .001$) and health-threat categories (*Md* = 1.35, *SE* = 0.20, $p < .001$).

A significant main effect of image category was found in session 16, $F(2,28) = 38.74$, $p < .001$, $\eta_p^2 = .72$. Pairwise comparisons revealed that the images in the musculoskeletal-pain category were significantly less arousing than the images included in the facial expressions of pain (*Md* = 1.38, *SE* = 0.29, $p = .001$) and general-threat categories (*Md* = 2.37, *SE* = 0.27, $p = .002$). Images included in the facial expression of pain category were also found to be significantly less arousing than those included in the general-threat category (*Md* = 0.98, *SE* =

For peer review only - http://bmjopen.bmj.com/site/about/guidelines.xhtml

0.21, p = .002). Images in the health-threat category were significantly less arousing than the images included in the facial expression of pain (Md = 1.57, SE = 0.36, p = .004) and general-threat categories (Md = 2.56, SE = 0.29, p < .001).

Differences in Valence Scores between Threatening Pictorial Categories across the eight Pictorial Visual Probe Sessions

A 4 (image category; musculoskeletal-pain, facial expressions of pain, general-threat and health-threat) x 8 (pictorial visual probe sessions; 2, 4, 6, 8, 10, 12, 14, 16) ANOVA was conducted to compare the pleasantness scores of the threatening categories across the 8 pictorial visual probe task sessions. The results showed a significant main effect of image category, $F(3,45) = 41.09$, $p < .001$, $\eta_p^2 = .73$, and a significant image category x session interaction, $F(7,102) = 9.33$, $p < .001$, $\eta_p^2 = .38$.

Post-hoc analyses within image categories. To explore the significant interaction, a series of one-way ANOVAS with 8 levels (Session; 2, 4, 6, 8, 10, 12, 14, 16) were run for each image category independently. These explored whether the pleasantness ratings for each image category differed across the sessions.

For the images included in the musculoskeletal-pain category, a main effect of session was found, $F(2,32) = 3.70$, $p = .033$, $\eta_p^2 = .20$. Pairwise comparisons revealed these images to be significantly more pleasant in session 2 than session 4 (Md = 0.54, SE = 0.11, p = .007).

For the images included in the facial expressions of pain category, a main effect of session was found, $F(7,105) = 3.44$, $p = .002$, $\eta_p^2 = .19$. Pairwise comparisons revealed these images to be significantly more pleasant in session 10 than session 6 (Md = 0.51, SE = 0.13, p = .03) and session 16 (Md = 0.43, SE = 0.09, p = .005).

For the images included in the general-threat category, a main effect of session was found, $F(7,105) = 6.84$, $p < .001$, $\eta_p^2 = .31$. Pairwise comparisons revealed these images to be significantly more pleasant in session 10 than session 6 (Md = 0.61, SE = 0.13, p = .009), session 12 (Md = 0.56, SE = 0.14, p = .05) and session 16 (Md = 0.68, SE = 0.14, p = .007). General-

For peer review only - http://bmjopen.bmj.com/site/about/guidelines.xhtml

threat images were significantly more pleasant in session 14 than session 6 (*Md* = 0.60, *SE* = 0.14, *p* = .019), session 12 (*Md* = 0.55, *SE* = 0.12, *p* = .013) and session 16 (*Md* = 0.67, *SE* = 0.11, *p* = .001). Images in this category were also significantly more pleasant in session 4 than session 6 (*Md* = 0.45, *SE* = 0.11, *p* = .04) and session 16 (*Md* = 0.52, *SE* = 0.11, *p* = .007).

For the images included in the health-threat category, a main effect of session was found, $F(7,105) = 9.87$, $p < .001$, $\eta_p^2 = .40$. Pairwise comparisons revealed these images to be significantly more pleasant in session 8 than session 10 (*Md* = 0.73, *SE* = 0.10, *p* = .005). Images in this category were significantly more pleasant in session 16 than session 2 (*Md* = 0.54, *SE* = 0.11, *p* = .004), session 4 (*Md* = 0.62, *SE* = 0.12, *p* = .003), session 6 (*Md* = 0.52, *SE* = 0.12, *p* = .02), session 10 (*Md* = 1.00, *SE* = 0.10, *p* < .001), session 12 (*Md* = 0.56, *SE* = 0.12, *p* = .008) and session 14 (*Md* = 0.80, *SE* = 0.10, *p* < .001).

Post-hoc analyses within sessions. To further explore the significant interaction, one-way ANOVAs with 4 levels (image category; musculoskeletal-pain, facial expressions of pain, general-threat and health-threat) were conducted for each session independently. These explored whether pleasantness ratings for each image category differed within each session.

For session 2, a significant main effect was found, $F(3,45) = 13.36$, $p < .001$, $\eta_p^2 = .47$. Pairwise comparisons revealed that images in the musculoskeletal-pain category were significantly more pleasant than images included in the facial expressions of pain (*Md* = 1.00, *SE* = 0.18, *p* < .001), general-threat (*Md* = 0.98, *SE* = 0.20, *p* = .001) and health-threat categories (*Md* = 0.72, *SE* = 0.21, *p* = .024).

For session 4, a significant main effect was found, $F(3,45) = 7.09$, $p = .001$, $\eta_p^2 = .32$. Pairwise comparisons showed that the images in the musculoskeletal-pain category were significantly more pleasant than the facial expressions of pain (*Md* = 0.52, *SE* = 0.10, *p* = .001) and general-threat categories (*Md* = 0.48, *SE* = 0.12, *p* = .005).

For session 6, a significant main effect was found, $F(3,45) = 32.12$, $p < .001$, $\eta_p^2 = .68$. Pairwise comparisons revealed that images in the musculoskeletal-pain category were

For peer review only - http://bmjopen.bmj.com/site/about/guidelines.xhtml

significantly more pleasant than the images included in the facial expressions of pain (*Md* = 1.05, *SE* = 0.14, $p < .001$) and general-threat categories (*Md* = 1.27, *SE* = 0.13, $p < .001$). The images in the health-threat category were also significantly more pleasant than the images included in the facial expressions of pain (*Md* = 0.56, *SE* = 0.14, $p = .01$) and general-threat categories (*Md* = 0.77, *SE* = 0.13, $p < .001$).

For session 8, a significant main effect was found, $F(3,45) = 11.73$, $p < .001$, $\eta_p^2 = .44$. Pairwise comparisons revealed that images in the musculoskeletal-pain category were significantly more pleasant than those included in the facial expressions of pain (*Md* = 0.62, *SE* = 0.07, $p < .001$) and general-threat categories (*Md* = 0.57, *SE* = 0.14, $p = .004$). The images included in the health-threat category were also significantly more pleasant than those included in the facial expressions of pain (*Md* = 0.54, *SE* = 0.15, $p = .019$) and general-threat categories (*Md* = 0.49, *SE* = 0.14, $p = .016$).

For session 10, a significant main effect was found, $F(3,45) = 12.59$, $p < .001$, $\eta_p^2 = .46$. Pairwise comparisons revealed that images in the health-threat category were significantly less pleasant than those included in the musculoskeletal-pain (*Md* = 0.74, *SE* = 0.12, $p < .001$) and facial expressions of pain categories (*Md* = 0.44, *SE* = 0.12, $p = .017$).

For session 12, a significant main effect was found, $F(3,45) = 14.90$, $p < .001$, $\eta_p^2 = .50$. Pairwise comparisons revealed that images in the musculoskeletal-pain category were significantly more pleasant than images included in the facial expressions of pain (*Md* = 0.45, *SE* = 0.08, $p < .001$) and general-threat categories (*Md* = 0.88, *SE* = 0.16, $p < .001$). Images in the health-threat category were also significantly more pleasant than images included in the general health category (*Md* = 0.68, *SE* = 0.16, $p = .004$).

For session 14, a significant main effect was found, $F(3,45) = 13.30$, $p < .001$, $\eta_p^2 = .47$. Pairwise comparisons revealed that images in the musculoskeletal-pain category were significantly more pleasant than images included in the facial expressions of pain (*Md* = 0.71, *SE*

For peer review only - http://bmjopen.bmj.com/site/about/guidelines.xhtml

= 0.16, p = .003), general-threat (Md = 0.71, SE = 0.15, p = .001) and health-threat categories (Md = 0.82, SE = 0.12, p < .001).

For session 16, a significant main effect was found, $F(3,45) = 83.05$, p <.001, $\eta_p^2 = .85$. Pairwise comparisons revealed that images in the facial expressions of pain category were significantly less pleasant than musculoskeletal-pain (Md = 0.97, SE = 0.10, p < .001) and health-threat categories (Md = 0.99, SE = 0.12, p < .001). Images in the general-threat category were also significantly less pleasant than those included in the musculoskeletal-pain (Md = 1.34, SE = 0.11, p < .001), facial expressions of pain (Md = 0.37, SE = 0.09, p = .007) and health-threat categories (Md = 1.36, SE = 0.09, p < .001).

Differences in Pain Intensity Scores of Threatening Pain Categories across the eight Pictorial Visual Probe Sessions

A 2 (image category; musculoskeletal-pain, facial expressions of pain) x 8 (pictorial visual probe sessions; session 2, 4, 6, 8, 10, 12, 14, 16) ANOVA was conducted to compare the pain intensity scores of the threatening pain categories across the 8 pictorial visual probe task sessions. The results showed significant main effects of image category, $F(1,15) = 45.40$, p < .001, $\eta_p^2 = .75$, and session, $F(2,29) = 3.81$, p = .04, $\eta_p^2 = .20$, and a significant image category x session interaction, $F(3,44) = 7.44$, p < .001, $\eta_p^2 = .33$.

Post-hoc analyses within image categories. To explore the significant interaction, two one-way ANOVAs with 8 levels (Session; 2, 4, 6, 8, 10, 12, 14, 16) were run for each pain-related image category independently. These explored whether the pain intensity ratings of each pain-related image category differed across the sessions. For the musculoskeletal-pain category, a main effect of session was found, $F(2,25) = 4.70$, p = .023, $\eta p^2 = .24$. Pairwise comparisons revealed that the images included in session 14 had significantly less pain intensity scores than the images included in session 8 (Md = 1.06, SE = 0.24, p = .014), session 10 (Md = 0.95, SE = 0.19, p = .004) and session 12 (Md = 1.19, SE = 0.15, p < .001). Images in session 4 had significantly higher pain intensity scores than the images included in session 6 (Md = 0.82, SE =

For peer review only - http://bmjopen.bmj.com/site/about/guidelines.xhtml

0.21, p = .05). The images included in session 12 had also significantly higher pain intensity scores than the images included in session 16 (*Md* = 1.03, *SE* = 0.17, p = .001).

For the facial expression of pain category, a main effect of session was found, F(3,39) = 4.65, p = .01, ηp^2 = .24. Pairwise comparisons revealed that the images included in session 10 had significantly less pain intensity scores that the images included in session 1 (*Md* = 1.07, *SE* = 0.25, p = .02) and session 6 (*Md* = 1.38, *SE* = 0.31, p = .013).

Differences in low-level features of pictorial categories across the eight visual probe sessions

A series of one-way ANOVAs were conducted independently to compare the scores of complexity, red, green and blue saturation, luminance and RMS contrast level of the threat related categories (i.e., musculoskeletal-pain, facial expression of pain, general-threat and health-threat) across the 8 pictorial visual probe task sessions (i.e., Session 2, 4, 6, 8, 10, 12, 14, 16).

For complexity, the analysis revealed a significant main effect of image category, F(3, 224) = 5.15, p = .002, η_p^2 = .06. There was no significant main effect of session, F(7, 224) = 1.22, p = .30, and no significant image category x session interaction, F(21, 224) = 0.82, p =.70.

For red saturation, the analysis revealed a significant main effect of image category, F(3, 224) = 5.05, p = .002, η_p^2 = .06. There was no significant main effect of session , F(7,224) = 1.03, p = .41, and no significant image category x session interaction, F(21,224) = 1.196, p =.26.

For green saturation, the analysis revealed a significant main effect of image category, F(3, 224) = 4.08, p = .008, η_p^2 = .08. There was no significant main effect of session, F(7, 224) = 1.65, p = .12, and no and no significant image category x session interaction, F(21,224) = 1.41, p =.12.

For blue saturation, the analysis revealed a significant main effect of image category, F(3, 224) = 5.288, p = .002, η_p^2 = 0.07, and a significant image category x session interaction, F(21, 224) = 1.672, p = .04, η_p^2 = 0.14. There was no significant main effect of session, F(7, 224) = 1.750, p = .10.

For peer review only - http://bmjopen.bmj.com/site/about/guidelines.xhtml

For luminance, the analysis revealed a significant main effect of image category, $F(3, 224) = 4.548$, $p = .004$, $\eta_p^2 = 0.06$. The analysis revealed no significant main effect of session, $F(7,224) = 1.504$, $p = .17$, and no significant image category x session interaction, $F(21, 224) = 1.361$, $p = .14$.

For RMS contrast level, the analysis revealed a significant main effect of image category, $F(3, 224) = 6.84$, $p < .001$, $\eta_p^2 = .08$, and a significant image category x session interaction, $F(21,224) = 1.98$, $p = .008$. There was no significant main effect of session, $F(7,224) = 1.68$, $p = .12$,

Post-hoc analyses within image categories. To explore the significant interaction for blue saturation, a series of one-way ANOVAS with 8 levels (Session; 2, 4, 6, 8, 10, 12, 14, 16) were run for each image category independently. These explored whether the blue saturation scores for each image category differed across the sessions. No significant main effect of session was found for the musculoskeletal-pain category, $F(7,56) = 0.82$, $p = .57$, or the health-threat category, $F(7,56) = 1.59$, $p = .16$.

For the facial expressions of pain category, a significant interaction for session number was found, $F(7,56) = 2.94$, $p = .01$, $\eta_p^2 = .27$. Pairwise comparisons did not reveal any significant differences between the factors.

For the general-threat category, a significant main effect of session number, $F(7,56) = 2.20$, $p = .05$, $\eta_p^2 = .22$. Pairwise comparisons did not reveal any significant differences between the factors.

To explore the significant interaction for RMS contrast level, a series of one-way ANOVAS with 8 levels (Session; 2, 4, 6, 8, 10, 12, 14, 16) were run for each image category independently. These explored whether the RMS contrast level scores for each image category differed across the sessions. No significant main effect of session was found for the musculoskeletal-pain category, $F(7,56) = 0.59$, $p = .76$, or the health-threat category, $F(7,56) = 0.16$, $p = .99$

For peer review only

For the facial expressions of pain category, a significant interaction for session number was found, $F(7,56) = 5.127$, $p < .001$, $\eta_p^2 = 0.39$. Pairwise comparisons revealed that the images in session 6 had significantly less RMS contrast than those included in session 4 (*Md* = 36.02, *SE* = 7.76, *p* = .001), session 10 (*Md* = 30.81, *SE* = 7.76, *p* = .006), session 14 (*Md* = 25.96, *SE* = 7.76, *p* = .04) and session 16 (*Md* = 37.09, *SE* = 776, *p*< .001).

For the general-threat category, a significant interaction for session number was found, $F(7,56) = 3.252$, $p = .006$, $\eta_p^2 = 0.29$. Pairwise comparisons did not reveal any significant interaction between the sessions.

Post-hoc analyses within sessions. To further explore the significant interaction for blue saturation, eight one-way ANOVAs with 4 levels (image category; musculoskeletal-pain, facial expressions of pain, general-threat and health-threat) were conducted for each session independently. These explored whether blue saturation scores between the five image categories differed within each session (i.e., session 2, 4, 6, 8, 10, 12, 14, 16).

No significant main effects were found for session 2, $F(3,28) = 1.49$, $p = .24$, session 4, $F(3,28) = 0.18$, $p = .91$, session 6, $F(3,28) = 1.37$, $p = .27$, session 8, $F(3,28) = 1.127$, $p = .36$, and session 14, $F(3,28) = 0.508$, $p = .68$.

For session 10, there was a significant main effect of image category, $F(3,28) = 5.56$, $p = .004$, $\eta_p^2 = .37$. Pairwise comparisons revealed that the images included in the facial expression of pain category had significantly higher blue saturation than the images included in the general-threat category (*Md* = 70.64, *SE* = 18.03, *p* = .003).

For session 12, there was a significant main effect of image category, $F(3,28) = 3.55$, $p = .027$, $\eta_p^2 = .28$. Pairwise comparisons revealed that the images included in the musculoskeletal-pain category had significantly higher blue saturation than the images included in the general-threat category (*Md* = 62.46, *SE* = 21.76, *p* = .046).

For session 16, there was a significant main effect of image category, $F(3,28) = 6.99$, $p = .001$, $\eta_p^2 = .43$. Pairwise comparisons revealed that the images included in the health-threat

For peer review only - http://bmjopen.bmj.com/site/about/guidelines.xhtml

categories had significantly higher blue saturation than the images included in the musculoskeletal-pain (*Md* = 74.57, *SE* = 20.09, *p* = .005) and general-threat categories (*Md* = 83.89, *SE* = 20.09, *p* = .002)

To further explore the significant interaction for RMS contrast level, eight one-way ANOVAs with 4 levels (image category; musculoskeletal-pain, facial expressions of pain, general-threat and health-threat) were conducted for each session independently. These explored whether the scores of RMS contrast level between the five image categories differed within each session (i.e., session 2, 4, 6, 8, 10, 12, 14, 16).

No significant main effects were found for session 2, $F(3,28) = 0.720$, $p = .55$, session 8, $F(3,28) = 1.56$, $p = .22$, session 12, $F(3,28) = 0.62$, $p = .61$, and session 14, $F(3,28) = 1.76$, $p = .18$.

For session 4, there was a significant main effect of image category, $F(3,28) = 5.69$, $p = .004$, $\eta_p^2 = .38$. Pairwise comparisons revealed that the images included in the facial expression of pain category had significantly higher RMS contrast than the images included in the health-threat category (*Md* = 33.44, *SE* = 8.27, *p* = .002).

For session 6, there was a significant main effect of image category, $F(3,28) = 4.00$, $p = .02$, $\eta_p^2 = .30$. Pairwise comparisons revealed that the images included in the musculoskeletal-pain category had significantly higher RMS contrast than those included in the facial expressions of pain category (*Md* = 17.69, *SE* = 5.95, *p* = .04).

For session 10, there was a significant main effect of image category, $F(3,28) = 6.02$, $p = .003$, $\eta_p^2 = .39$. Pairwise comparisons revealed that the images included in the facial expressions of pain category had significantly higher RMS contrast than those included in the general-threat (*Md* = 30.37, *SE* = 7.35, *p* = .002) and health-threat categories (*Md* = 21.42, *SE* = 7.35, *p* = .04).

For session 16, there was a significant main effect of image category, $F(3,28) = 3.18$, $p = .04$, $\eta_p^2 = .25$. Pairwise comparisons revealed that the images included in the facial expressions

For peer review only - http://bmjopen.bmj.com/site/about/guidelines.xhtml

of pain category had significantly higher RMS contrast level than those included in the health-threat category (*Md* = 29.32, *SE* = 10.24, *p* = .05).

For peer review only

For peer review only - http://bmjopen.bmj.com/site/about/guidelines.xhtml

References

1. Kučera H, Francis N. *Computational analysis of present-day American English*. Providence: Brown University Press, 1967.

2. AP Y. The Nature of Recollection and Familiarity: A Review of 30 Years of Research. Journal of Memory and Language 2002;**46**(3):441-517 doi: 10.1006/jmla.2002.2864.

3. Balota DA, Chumbley JI. Are lexical decisions a good measure of lexical access? The role of word frequency in the neglected decision stage. Journal of Experimental Psychology: Human perception and performance 1984;**10**(3):340-57.

4. Sharpe L, Ianiello, M., Dear, B. F, Nicholson Perry, K., Refshauge, K., Nicholas, M. K. Is there a potential role for attention bias modification in pain patients? Results of 2 randomised, controlled trials. Pain 2012;**153**(3):722-31.

5. Schoth D, Liossi C. Specificity and time-course of attentional bias in chronic headache: a visual-probe investigation. Clinical Journal of Pain 2013;**29**(7):583-90 doi: 10.1097/AJP.0b013e31826b4849.

6. Carleton R, Richter A, Asmundson G. Attention Modification in Persons with Fibromyalgia: A Double Blind, Randomized Clinical Trial. Cognitive Behaviour Therapy 2011;**40**(4):279-90.

7. Macleod C. Cognitive bias modification procedures in the management of mental disorders. Curr Opin Psychiatry 2012;**25**(2):114-20 doi: 10.1097/YCO.0b013e32834fda4a.

8. MacLeod C, Mathews A, Tata P. Attentional bias in emotional disorders. Journal of Abnormal Psychology 1986;**95**(1):15-20 doi: 10.1037/0021-843X.95.1.15.

9. Melzack R. The McGill Pain Questionnaire: major properties and scoring methods. Pain 1975;**1**(3):277-99.

10. Wilson MD. The MRC Psycholinguistic Database: Machine Readable Dictionary, Version 2. Behavioural Research Methods, Instruments and Computers 1988;**20**(1):6-11.

For peer review only

11. MRC Psycholinguistic Database: Machine Usable Dictionary. Version 2.00.: MRC Psycholinguistic Database, 1997.

12. Lane RD, Chua PM, Dolan RJ. Common effects of emotional valence, arousal and attention on neural activation during visual processing of pictures. Neuropsychologia 1999;**37**(9):989-97 doi: 10.1016/S0028-3932(99)00017-2.

13. Niedenthal PM, Kitayama S. *The heart's eye: Emotional influences in perception and attention*. London: Academic Press, 2013.

14. Bradley MM, Lang PJ. Measuring Emotion: The Self-Assessment Manikin and the Semantic Differential. J Behav Therap & Exp Psychiat 1994;**25**(1):49-59.

15. Liossi C, Schoth D. Attention toward interpersonal stimuli in individuals with and without chronic daily headache. Journal of Neurology and Neurosurgery 2016;**3**(3):1-7.

16. Schoth DE, Liossi C. Attentional bias toward pictorial representations of pain in individuals with chronic headache. Clin J Pain 2010;**26**(3):244-50 doi: 10.1097/AJP.0b013e3181bed0f9.

17. Craig KD. The facial expression of pain. Better than a thousand words? APS Journal 1992;**1**(3):153-62.

18. Prkachin KM, Solomon PE. The structure, reliability and validity of pain expression: evidence from patients with shoulder pain. Pain 2008;**139**(2):267-74 doi: 10.1016/j.pain.2008.04.010.

19. Schoth DE, Georgallis T, Liossi C. Attentional bias modification in people with chronic pain: a proof of concept study. Cognitive Behaviour Therapy 2013;**42**(3):233-43.

20. Bradley MM, Lang PJ. The International Affective Picture System (IAPS) in the study of emotion and attention. In: Allen CaJJB, ed. Handbook of Emotion Elicitation and Assessment: Oxford University Press, 2007.

21. Leeuw M, Goossens ME, van Breukelen GJ, et al. Measuring perceived harmfulness of physical activities in patients with chronic low back pain: the Photograph Series of Daily

For peer review only - http://bmjopen.bmj.com/site/about/guidelines.xhtml

Activities--short electronic version. J Pain 2007;**8**(11):840-9 doi: 10.1016/j.jpain.2007.05.013.

22. Tottenham N, Tanaka JW, Leon AC, et al. The NimStim set of facial expressions: judgments from untrained research participants. Psychiatry Res 2009;**168**(3):242-9 doi: 10.1016/j.psychres.2008.05.006.

23. Nummenmaa L, Hyönä J, Calvo MG, & Nummenmaa, L. Eye movement assessment of selective attentional capture by emotional pictures. Emotion 2006;**6**(2):257-68 doi: 10.1037/1528-3542.6.2.257.

24. Borji A, Sihite DN, Itti L. What stands out in a scene? A study of human explicit saliency judgment. Vision research 2013;**91**:62-77 doi: 10.1016/j.visres.2013.07.016.

25. Henderson JM. Human gaze control during real-world scene perception. Trends in Cognitive Sciences 2003;**7**(11):498-504 doi: 0.1016/j.tics.2003.09.006.

26. Torralba A, Oliva, A., Castelhano, M. S., & Henderson, J. M. Contextual guidance of eye movements and attention in real-world scenes: The role of global features in object search. Psychological Review 2006;**113**(4):766-86 doi: 10.1037/0033-295X.113.4.766.

27. Buodo G, Sarlo M, Palomba D. Attentional resources measured by reaction times highlight differences within pleasant and unpleasant, high arousing stimuli. Motivation and Emotion 2002;**26**(2):123-38 doi: 10.1023/A:1019886501965.

For peer review only - http://bmjopen.bmj.com/site/about/guidelines.xhtml

Supplementary Material 2 – Outcome Measures

A detailed description of each outcome measure used in this study is provided here. While the questionnaires will be administered online as part of this study, the psychometric properties reported are for paper and pencil versions of the questionnaires.

The BPI-SF[1] is a commonly used measure of pain intensity and pain interference. Participants indicate their worst, least, average and current pain on an 11-point numerical scale ranging from *no pain at all* to *pain as bad as you can imagine*. Additional questions include how much relief pain medication has provided, along with pain interference on general activity, mood, walking ability, normal work, relations with others, sleep, and enjoyment of life. A body diagram is also provided for participants to indicate where they feel pain. The psychometric properties of the BPI are well supported in patients with cancer pain and other pain populations[1] [2], including internal consistency of pain intensity and interference items as assessed in patients with chronic nonmalignant pain (Cronbach's alpha .85 and .88 respectively).[3]

The HADS[4] consists of 14 items measuring the severity of anxiety and depression symptoms (seven items each) over the past week. Each item is measured on a four point scale, with possible scores ranging from 0 – 21 for each subscale. Higher scores indicate higher levels of anxiety and depression. A review of studies using the HADS (the majority of which were conducted with patients with cancer or other physical health conditions) has supported the internal consistency of anxiety (.68 - .93, mean .82) and depression (.67 - .90, mean .82) subscales.[5]

The STAI[6] is a 40-item measure of state and trait anxiety (20 items each). Each item is measuerd on a four point scale , with possible scores for both state and trait levels ranging between 20 and 80. Higher scores represent more intense or more frequent feelings of anxiety. A review of 45 articles reporting psychometric properties from clinical and non-clinical samples of state and trait subscales found high levels of internal consistency (.91 and .89 respectively) and test-retest reliability (.70 and .88 respectively).[7]

For peer review only - http://bmjopen.bmj.com/site/about/guidelines.xhtml

The FOP-III[8] is a 30 item measure of pain-related fear. On a 5-point scale, respondents are asked to rate how fearful they are of pain associated with specific situations and events (e.g., being in an automobile accident). Possible scores range between 30 and 150, with higher scores representing a more intense fear of pain. In addition to a total score, the measure also provides subscales of minor, severe, and medical pain-related fear. The FOP-III is commonly used in clinical and non-clinical populations, and possesses high internal consistency as indexed in a non-clinical sample (total score = .93; severe = .88; minor = .86, medical = .88).[9]

The MOS-SS[10] is a twelve-item questionnaire developed for patients with chronic illness, measuring six dimensions of sleep over the past four weeks; sleep initiation (time to fall asleep), quantity (hours per night sleeping), maintenance, respiratory problems, perceived adequacy, and somnolence. A sleep problems index which summarises information from nine items may also be scored. Responses are recoded on a 0 – 100 scale, with higher scores indicating more of the concept being measured (e.g., greater difficulties falling asleep). Research has supported the internal consistency of the MOS-SS scales to be acceptable (.70) or higher in samples of patients with neuropathic pain.[11 12]

The SOT[13] assesses participants' attitudes towards their online administered treatment, and will be used at the study endline. Participants state how strongly they agree/disagree with each of the following items: (a) the use of a computer to access treatment, (b) level of easiness of the online intervention, (c) duration of the potential effects of the treatment, and (d) potential recommendation of online interventions to others. Responses range from "*Agree very strongly*" to "*Disagree very strongly*". An additional question asks participants how helpful they have found the online intervention, with responses ranging from "*Very helpful*" to "*Not at all helpful*". Two open questions are also included that require participants to state the aspects of the intervention they most liked and disliked.

The EOT, developed by the researchers, will be presented at the end of every online session. The EOT includes three questions. The first asks participants to indicate where they

For peer review only - http://bmjopen.bmj.com/site/about/guidelines.xhtml

completed the online training session (e.g., home or office). The second is related to whether or not they have been distracted during the training and, if distracted, to indicate the source of distraction (e.g., telephone ringing). The third question asks participants to rate the level of their concentration during the online task on an 11-point Visual Analogue Scale (VAS) numbered from 0 (i.e., *no concentration at all*) to 10 (i.e. *very concentrated*).

For peer review only

References

1. Cleeland CS, Ryan KM. Pain assessment: global use of the Brief Pain Inventory. Ann Acad Med Singapore 1994;**23**(2):129-38.

2. Jensen MP. The validity and reliability of pain measures in adults with cancer. The Journal of Pain 2003;**4**(1):2-21 doi: 10.1054/jpai.2003.1.

3. Tan G, Jensen MP, Thornby JI, et al. Validation of the Brief Pain Inventory for chronic nonmalignant pain. The Journal of Pain 2004;**5**(2):133-37 doi: 10.1016/j.jpain.2003.12.005.

4. Zigmond AS, Snaith RP. The hospital anxiety and depression scale. Acta Psychiatr Scand 1983;**67**(6):361-70.

5. Bjelland I, Dahl AA, Tangen T, et al. The validity of the Hospital Anxiety and Depression Scale: An updated literature review. Journal of Psychosomatic Research 2002;**52**:69-77 doi: 10.1016/S0022-3999(01)00296-3.

6. Spielberger CD, Gorsuch RL, Lushene RE. *State Trait Anxiety Inventory*. Palo Alto, California: Consulting Psychologists Press, 1970.

7. Barnes LLB, Harp D, Jung WS. Reliability generalization of scores on the Spielberger State-Trait Anxiety Inventory. Educational and Psychological Measurement 2002;**62**(4):603 - 18 doi: 10.1177/0013164402062004005.

8. McNeil DW, Rainwater A. Development of the Fear of Pain Questionnaire-III. Journal of Behavioral Medicine 1998;**21**(4):389-410 doi: 10.1023/A:1018782831217.

9. Roelofs J, Peters ML, Deutz J, et al. The Fear of Pain Questionnaire (FPQ): further psychometric examination in a non-clinical sample. PAIN 2005;**116**(3):339-46 doi: 10.1016/j.pain.2005.05.003.

10. Hays RD, Stewart AL. Sleep Measures. Measuring functioning and well-being. In: Steweart AL, JEJ W, eds. Measuring function and well-being; the medical outcome study approach. Duke: Duke University Press, 1992:235-59.

For peer review only - http://bmjopen.bmj.com/site/about/guidelines.xhtml

11. Hays RD, Martin SA, Sesti AM, et al. Psychometric properties of the medical outcomes study sleep measure. Sleep Medicine 2005;**6**(1):41-44 doi: 10.1016/j.sleep.2004.07.006.

12. Viala-Danten M, Martin S, Guillemin I, et al. Evaluation of the reliability and validity of the Medical Outcomes Study sleep scale in patients with painful diabetic peripheral neuropathy during an international clinical trial. Health and Quality of Life Outcomes 2008;**6**(113) doi: 10.1186/1477-7525-6-113.

13. Richards D, Timulak L. Satisfaction with therapist-delivered vs. self-administered online cognitive behavioural treatments for depression symptoms in college students. British Journal of Guidance & Counselling 2013;**41**(2):193-207 doi: 10.1080/03069885.2012.726347.

For peer review only

Supplementary Material 3 – Think Aloud Study

A qualitative think aloud study[1 2] was conducted following the development of the initial version of the ABMT intervention to address the question: *What are the thoughts and first impressions of individuals with chronic musculoskeletal pain towards an online ABMT programme?* Ethical approval was obtained from the University of Southampton Research Ethics Committee (ERGO ID: 26486). Thirteen participants (mean age = 37.54, *SD* = 16.31, range 21 – 60 years) with chronic musculoskeletal pain were recruited from the South of England from a chronic pain support group.

Each participant met with the researcher (FH) on a single occasion to take part in a think aloud interview followed by a short semi-structured interview. Participants navigated their way through the iABMT Lifeguide website and completed the first assessment visual-probe task, with the researcher seated next to them. The researcher used an interview topic guide featuring standardized instructions that were read at the beginning of the study, as well as a list of pre-determined neutral prompts used when the participant stopped talking or showed a reaction to the material through their facial expressions. On occasions where the participant deviated from the topic, the researcher attempted to steer the participant's focus back to the task at hand. The prompts used were similar to those in former research[3], such as '*what are you thinking right now?*' and '*tell me why you think that*'.

Following the think-aloud interview, a short semi-structured interview took place for the researcher to ask questions about any specific aspects of the intervention that had not yet been discussed. Questions were also asked regarding thoughts about the intervention as a whole. This method of triangulation is commonly used to complement a think-aloud interview because it can be difficult to interpret spoken thoughts alone.[4 5] Following transcription of the interviews, thematic analysis using inductive encoding was used to identify themes that clearly represent the raw data, following guidelines set out by Braun and

For peer review only - http://bmjopen.bmj.com/site/about/guidelines.xhtml

Clarke.[6] Three themes and 13 subthemes were identified, which are shown in a thematic map in Figure S1 and defined in Table S1 below. While it is beyond the scope of this article to present the results of this thematic analysis in full, the main points resulting in amendments to the iABMT intervention are presented here.

Regarding the iABMT website, participants liked the continuity of design between webpages, although pointed out inconsistencies in the formatting of text, which have been rectified. On some pages the volume of text was considered to be too great, and therefore where possible text has been reduced, language simplified, better use of spacing made, and longer paragraphs divided into multiple shorter paragraphs. While some participants were positive about the inclusion of images, others felt additional visual stimuli could be added throughout. It was therefore decided to only include images where these would help emphasize the points being made. Regarding the visual-probe task sessions, one important practicality raised was ability of participants to stay seated for a sufficient amount of time in order to complete the task in a single session. An additional inclusion criteria has therefore been added to the study, being that participants must be able to sit at a personal computer for forty minutes. It was also mentioned that participants needed sufficient computer skills to take part in the study. The inclusion criteria were therefore amended to state that participants need both access to and familiarity using a Window-based computer.

For peer review only - http://bmjopen.bmj.com/site/about/guidelines.xhtml

References

1. Yardley L, Ainsworth B, Arden-Close E, et al. The person-based approach to enhancing the acceptability and feasibility of interventions. Pilot and Feasibility Studies 2015;**1**(1):37.

2. Van den Haak MJ, De Jong MD, Schellens PJ. Evaluation of an informational web site: three variants of the think-aloud method compared. Technical Communication 2007;**54**(1):58-71.

3. Morrison L, Moss-Morris R, Michie S, et al. Optimizing engagement with internet-based health behaviour change interventions: Comparison of self-assessment with and without tailored feedback using a mixed methods approach. British Journal of Health Psychology 2014;**19**(4):839-55 doi: 10.1111/bjhp.12083.

4. Charters E. The use of think-aloud methods in qualitative research an introduction to think-aloud methods. Brock Education Journal 2003;**12**(2).

5. Anthierens S, Tonkin-Crine S, Douglas E, et al. General practitioners' views on the acceptability and applicability of a web-based intervention to reduce antibiotic prescribing for acute cough in multiple European countries: a qualitative study prior to a randomised trial. BMC Family Practice 2012;**13**(101) doi: 10.1186/1471-2296-13-101.

6. Braun V, Clarke V. Using thematic analysis in psychology. Qualitative Research in Psychology 2006;**3**(2):77-101 doi: 10.1191/1478088706qp063oa.

For peer review only - http://bmjopen.bmj.com/site/about/guidelines.xhtml

Table S1. Definitions of themes and subthemes identified in the think aloud study

Theme/Sub-theme	Definition
A. AMBT website	Comments relating to the ABMT website including the post-intervention questionnaires
A1. Accessibility and usability	Comments relating to the ease with which participants were able to use the website and the extent to which it was deemed to be accessible
A2. Visual design	Comments relating to the visual design and appearance of the website, including colour, orientation of elements and text
A3. Questionnaires	Comments regarding any aspect of the post-intervention questionnaires, including their purpose and presentation
A4. Specific menu pages	Comments pertaining to the six main menu pages, which are titled (i) How does ABMT work, (ii) Will ABMT cure my pain, (iii) The team behind ABMT, (iv) Evidence for ABMT, (v) Why try ABMT, and (iv) What is it like to use ABMT?
B. Visual-probe task	Comments relating to any and all aspects of the visual-probe tasks
B1. Experiences of testing the intervention	Comments provided prior to and during testing of the visual-probe task
B2. Attitudes towards task	Positive and negative attitudes expressed by participants regarding the visual-probe task
B3. Practicalities	Comments pertaining to the feasibility and practicalities of completing the visual-probe task, including commitment needed
C. Influencing factors	All personal and situational factors that may affect the participants' experiences of testing the intervention
C1. Interviewing situation	Comments relating to the style of qualitative research adopted for the think aloud study
C2. Personal barriers	Comments relating to personal factors that hindered participants' completion of the visual-probe task
C3. Personal facilitators	Comments relating to personal factors that increased participants' ability to complete the visual-probe task and improved their attitude regarding it

For peer review only - http://bmjopen.bmj.com/site/about/guidelines.xhtml

SPIRIT

STANDARD PROTOCOL ITEMS: RECOMMENDATIONS FOR INTERVENTIONAL TRIALS

SPIRIT 2013 Checklist: Recommended items to address in a clinical trial protocol and related documents*

Section/item	Item No	Description	Addressed on page number
**Administrative information**			
Title	1	Descriptive title identifying the study design, population, interventions, and, if applicable, trial acronym	1
Trial registration	2a	Trial identifier and registry name. If not yet registered, name of intended registry	1
	2b	All items from the World Health Organization Trial Registration Data Set	Registered with clinicaltrials.gov
Protocol version	3	Date and version identifier	1
Funding	4	Sources and types of financial, material, and other support	28
Roles and responsibilities	5a	Names, affiliations, and roles of protocol contributors	1, 28
	5b	Name and contact information for the trial sponsor	28
	5c	Role of study sponsor and funders, if any, in study design; collection, management, analysis, and interpretation of data; writing of the report; and the decision to submit the report for publication, including whether they will have ultimate authority over any of these activities	28
	5d	Composition, roles, and responsibilities of the coordinating centre, steering committee, endpoint adjudication committee, data management team, and other individuals or groups overseeing the trial, if applicable (see Item 21a for data monitoring committee)	______________

For peer review only

**Introduction**			
Background and rationale	6a	Description of research question and justification for undertaking the trial, including summary of relevant studies (published and unpublished) examining benefits and harms for each intervention	4-6
	6b	Explanation for choice of comparators	4
Objectives	7	Specific objectives or hypotheses	6
Trial design	8	Description of trial design including type of trial (eg, parallel group, crossover, factorial, single group), allocation ratio, and framework (eg, superiority, equivalence, noninferiority, exploratory)	7-8
**Methods: Participants, interventions, and outcomes**			
Study setting	9	Description of study settings (eg, community clinic, academic hospital) and list of countries where data will be collected. Reference to where list of study sites can be obtained	6-8
Eligibility criteria	10	Inclusion and exclusion criteria for participants. If applicable, eligibility criteria for study centres and individuals who will perform the interventions (eg, surgeons, psychotherapists)	7
Interventions	11a	Interventions for each group with sufficient detail to allow replication, including how and when they will be administered	8-14
	11b	Criteria for discontinuing or modifying allocated interventions for a given trial participant (eg, drug dose change in response to harms, participant request, or improving/worsening disease)	19
	11c	Strategies to improve adherence to intervention protocols, and any procedures for monitoring adherence (eg, drug tablet return, laboratory tests)	13
	11d	Relevant concomitant care and interventions that are permitted or prohibited during the trial	7
Outcomes	12	Primary, secondary, and other outcomes, including the specific measurement variable (eg, systolic blood pressure), analysis metric (eg, change from baseline, final value, time to event), method of aggregation (eg, median, proportion), and time point for each outcome. Explanation of the clinical relevance of chosen efficacy and harm outcomes is strongly recommended	14-15
Participant timeline	13	Time schedule of enrolment, interventions (including any run-ins and washouts), assessments, and visits for participants. A schematic diagram is highly recommended (see Figure)	Table 1

For peer review only - http://bmjopen.bmj.com/site/about/guidelines.xhtml

Sample size	14	Estimated number of participants needed to achieve study objectives and how it was determined, including clinical and statistical assumptions supporting any sample size calculations	9
Recruitment	15	Strategies for achieving adequate participant enrolment to reach target sample size	7
**Methods: Assignment of interventions (for controlled trials)**			
Allocation:			
Sequence generation	16a	Method of generating the allocation sequence (eg, computer-generated random numbers), and list of any factors for stratification. To reduce predictability of a random sequence, details of any planned restriction (eg, blocking) should be provided in a separate document that is unavailable to those who enrol participants or assign interventions	8-9
Allocation concealment mechanism	16b	Mechanism of implementing the allocation sequence (eg, central telephone; sequentially numbered, opaque, sealed envelopes), describing any steps to conceal the sequence until interventions are assigned	8-9
Implementation	16c	Who will generate the allocation sequence, who will enrol participants, and who will assign participants to interventions	8-9
Blinding (masking)	17a	Who will be blinded after assignment to interventions (eg, trial participants, care providers, outcome assessors, data analysts), and how	8-9
	17b	If blinded, circumstances under which unblinding is permissible, and procedure for revealing a participant's allocated intervention during the trial	8-9
**Methods: Data collection, management, and analysis**			
Data collection methods	18a	Plans for assessment and collection of outcome, baseline, and other trial data, including any related processes to promote data quality (eg, duplicate measurements, training of assessors) and a description of study instruments (eg, questionnaires, laboratory tests) along with their reliability and validity, if known. Reference to where data collection forms can be found, if not in the protocol	15-19, Supplementary material 2
	18b	Plans to promote participant retention and complete follow-up, including list of any outcome data to be collected for participants who discontinue or deviate from intervention protocols	11-12

For peer review only - http://bmjopen.bmj.com/site/about/guidelines.xhtml

Data management	19	Plans for data entry, coding, security, and storage, including any related processes to promote data quality (eg, double data entry; range checks for data values). Reference to where details of data management procedures can be found, if not in the protocol	19
Statistical methods	20a	Statistical methods for analysing primary and secondary outcomes. Reference to where other details of the statistical analysis plan can be found, if not in the protocol	15-19
	20b	Methods for any additional analyses (eg, subgroup and adjusted analyses)	17
	20c	Definition of analysis population relating to protocol non-adherence (eg, as randomised analysis), and any statistical methods to handle missing data (eg, multiple imputation)	17
**Methods: Monitoring**			
Data monitoring	21a	Composition of data monitoring committee (DMC); summary of its role and reporting structure; statement of whether it is independent from the sponsor and competing interests; and reference to where further details about its charter can be found, if not in the protocol. Alternatively, an explanation of why a DMC is not needed	________
	21b	Description of any interim analyses and stopping guidelines, including who will have access to these interim results and make the final decision to terminate the trial	________
Harms	22	Plans for collecting, assessing, reporting, and managing solicited and spontaneously reported adverse events and other unintended effects of trial interventions or trial conduct	19
Auditing	23	Frequency and procedures for auditing trial conduct, if any, and whether the process will be independent from investigators and the sponsor	________
**Ethics and dissemination**			
Research ethics approval	24	Plans for seeking research ethics committee/institutional review board (REC/IRB) approval	19
Protocol amendments	25	Plans for communicating important protocol modifications (eg, changes to eligibility criteria, outcomes, analyses) to relevant parties (eg, investigators, REC/IRBs, trial participants, trial registries, journals, regulators)	19

For peer review only - http://bmjopen.bmj.com/site/about/guidelines.xhtml

Consent or assent	26a	Who will obtain informed consent or assent from potential trial participants or authorised surrogates, and how (see Item 32)	9, 19
	26b	Additional consent provisions for collection and use of participant data and biological specimens in ancillary studies, if applicable	____________
Confidentiality	27	How personal information about potential and enrolled participants will be collected, shared, and maintained in order to protect confidentiality before, during, and after the trial	19
Declaration of interests	28	Financial and other competing interests for principal investigators for the overall trial and each study site	28
Access to data	29	Statement of who will have access to the final trial dataset, and disclosure of contractual agreements that limit such access for investigators	28
Ancillary and post-trial care	30	Provisions, if any, for ancillary and post-trial care, and for compensation to those who suffer harm from trial participation	____________
Dissemination policy	31a	Plans for investigators and sponsor to communicate trial results to participants, healthcare professionals, the public, and other relevant groups (eg, via publication, reporting in results databases, or other data sharing arrangements), including any publication restrictions	19
	31b	Authorship eligibility guidelines and any intended use of professional writers	28
	31c	Plans, if any, for granting public access to the full protocol, participant-level dataset, and statistical code	19
**Appendices**			
Informed consent materials	32	Model consent form and other related documentation given to participants and authorised surrogates	Supplementary materials
Biological specimens	33	Plans for collection, laboratory evaluation, and storage of biological specimens for genetic or molecular analysis in the current trial and for future use in ancillary studies, if applicable	N/A

*It is strongly recommended that this checklist be read in conjunction with the SPIRIT 2013 Explanation & Elaboration for important clarification on the items. Amendments to the protocol should be tracked and dated. The SPIRIT checklist is copyrighted by the SPIRIT Group under the Creative Commons "Attribution-NonCommercial-NoDerivs 3.0 Unported" license.

For peer review only

BMJ Open

Internet-delivered attentional bias modification training (iABMT) for the management of chronic musculoskeletal pain: a protocol for a randomised controlled trial

Journal:	*BMJ Open*
Manuscript ID	bmjopen-2019-030607.R3
Article Type:	Protocol
Date Submitted by the Author:	07-Jan-2020
Complete List of Authors:	Liossi, Christina; University of Southampton Faculty of Engineering and the Environment, Psychology;<br>Georgallis, Tsampikos; University of Southampton Faculty of Engineering and the Environment, School of Psychology<br>Zhang, Jin; University of Southampton Faculty of Engineering and the Environment, School of Psychology<br>Hamilton, Fiona; University of Southampton Faculty of Engineering and the Environment, School of Psychology<br>White, Paul; University of the West of England, Engineering, Design and Mathematics<br>Schoth, Daniel; University of Southampton Faculty of Engineering and the Environment, School of Psychology
**Primary Subject Heading**:	Neurology
Secondary Subject Heading:	Paediatrics, Qualitative research
Keywords:	Chronic pain, Musculoskeletal pain, Visual-probe task, Attention bias modification, Internet-delivered therapy, Randomised-controlled trial

SCHOLARONE™
Manuscripts

I, the Submitting Author has the right to grant and does grant on behalf of all authors of the Work (as defined in the below author licence), an exclusive licence and/or a non-exclusive licence for contributions from authors who are: i) UK Crown employees; ii) where BMJ has agreed a CC-BY licence shall apply, and/or iii) in accordance with the terms applicable for US Federal Government officers or employees acting as part of their official duties; on a worldwide, perpetual, irrevocable, royalty-free basis to BMJ Publishing Group Ltd ("BMJ") its licensees and where the relevant Journal is co-owned by BMJ to the co-owners of the Journal, to publish the Work in this journal and any other BMJ products and to exploit all rights, as set out in our licence.

The Submitting Author accepts and understands that any supply made under these terms is made by BMJ to the Submitting Author unless you are acting as an employee on behalf of your employer or a postgraduate student of an affiliated institution which is paying any applicable article publishing charge ("APC") for Open Access articles. Where the Submitting Author wishes to make the Work available on an Open Access basis (and intends to pay the relevant APC), the terms of reuse of such Open Access shall be governed by a Creative Commons licence – details of these licences and which Creative Commons licence will apply to this Work are set out in our licence referred to above.

Other than as permitted in any relevant BMJ Author's Self Archiving Policies, I confirm this Work has not been accepted for publication elsewhere, is not being considered for publication elsewhere and does not duplicate material already published. I confirm all authors consent to publication of this Work and authorise the granting of this licence.

For peer review only

Internet-delivered attentional bias modification training (iABMT) for the management of chronic musculoskeletal pain: a protocol for a randomised controlled trial

Christina Liossi[1*], Tsampikos Georgallis[1], Jin Zhang[1], Fiona Hamilton[1], Paul White[2], and Daniel E. Schoth[1,]

[1]Pain Research Laboratory, School of Psychology, University of Southampton, Highfield, Southampton, UK;

[2] Engineering Design and Mathematics, University of the West of England, Bristol, UK

Protocol version 4.0; 30/11/19

Corresponding author: Prof. Christina Liossi, Pain Research Laboratory, Department of Psychology, University of Southampton, Highfield, Southampton, SO17 1BJ, UK. E-mail: Cliossi@soton.ac.uk, Tel: 0044 (0)238059 4645.

Trial registration: This Study Protocol was registered in ClinicalTrials.gov (NCT02232100) on 1 September 2014

Word count: 4117

For peer review only

ATTENTIONAL BIAS MODIFICATION PROTOCOL

Abstract

Introduction: Chronic musculoskeletal pain is a complex medical condition that can significantly impact quality of life. Patients with chronic pain demonstrate attentional biases towards pain-related information. The therapeutic benefits of modifying attentional biases by implicitly training attention away from pain-related information towards neutral information have been supported in the small number of published studies. Limited research however has explored the efficacy of modifying pain-related biases via the internet. This protocol describes a randomised, double-blind internet-delivered attentional bias modification intervention, aimed to evaluate the efficacy of the intervention on reducing pain interference. Secondary outcomes are pain intensity, state and trait anxiety, depression, pain-related fear, and sleep impairment. This study will also explore the effects of training intensity on these outcomes, along with participants' perceptions about the therapy. **Methods and analysis**: The study is a double-blind randomised controlled trial with four arms exploring the efficacy of online attentional bias modification training versus placebo training theorised to offer no specific therapeutic benefit. Participants with chronic musculoskeletal pain will be randomised to one of four groups: (i) 10-session attentional modification group; (ii) 10-session placebo training group; (iii) 18-session attentional modification group; or (iv) 18-session placebo training group. In the attentional modification groups, the probe-classification version of the visual-probe task will be used to implicitly train attention away from threatening information towards neutral information. Following the intervention, participants will complete a short interview exploring their perceptions about the online training. In addition, a subgroup analysis for by participant age 16 – 24 and 25 – 60 will be undertaken. **Ethics and dissemination**: This study has been approved by the University of Southampton Research Ethics Committee. Results will be published in peer-reviewed journals, academic conferences, and in lay reports for pain charities and patient support groups. **Registration**: The study protocol has been registered with ClinicalTrial.gov (NCT02232100).

For peer review only

Strengths and limitations of this study

- This protocol describes an internet-delivered intervention to explore the efficacy of attentional bias modification training in patients with chronic musculoskeletal pain.
- Recruitment of a diverse sample in terms of age, socioeconomic status, and geographical region.
- Results will provide important information regarding dose effects, age effects and participants' experiences and attitudes using the online intervention.
- Limited control over the conditions under which training is completed.
- Impossibility of independently verifying participant diagnosis.

Keywords: chronic pain; musculoskeletal pain; visual-probe task; attention bias modification; internet-delivered therapy; randomised-controlled trial.

For peer review only

Introduction

Chronic musculoskeletal pain (CMSK) is pain arising from bones, muscles, ligaments, tendons and/or joints lasting for more than three months.[1] Prevalence estimates of CMSK vary considerably, which for adults is estimated to affect between 11.4 and 24% of the population[2] and for children and adolescents ranges between 4 and 40% of the population.[3] Negative effects on quality of life are commonly reported in adult[4-7] and paediatric[8-11] CMSK populations, and theory driven research[12-14] has shown that patients with chronic pain exhibit attentional biases (i.e., a selective attention) for salient pain-related words (e.g.,[15-18]) and images (e.g.,[19 20]) relative to neutral information. Three meta-analyses have supported the presence of attentional biases in chronic pain patients[21-23], although evidence for the time-course of such biases is mixed. Specifically, two meta-analyses reported larger effect sizes when stimuli are presented for longer than 1000 ms,[21 23] whereas a recent analysis found evidence of bias for presentation times of 500 – 1000ms but not when stimuli are presented for longer than 1000 ms.[22] Despite this inconsistency, attentional biases are commonly shown in chronic pain, and preliminary research has explored the effects of re-directing attention may have on pain and pain-related distress using attentional bias modification (ABM) techniques. ABM is a computer-based intervention which implicitly trains attention away from threat-related cues toward neutral information, and is frequently achieved through a modified version of the visual-probe task[24] (Figure 1).

Two small randomised controlled trials (RCTs)[25 26] and one pilot study[27] have used the modified probe-classification version of the visual-probe task to assess ABM in adults with chronic pain. Carleton and colleagues[25] found participants receiving ABM showed significant reductions pre- to post ABM in anxiety sensitivity and fear of pain, and marginally significant reduction in current pain severity. No significant reductions were found in illness/injury sensitivity or pain anxiety. Significantly more patients receiving ABM reported clinically significant change in current pain compared with those in the placebo group (44% vs. 17%, respectively). Attentional bias scores were not recorded, and therefore the researchers could not

For peer review only - http://bmjopen.bmj.com/site/about/guidelines.xhtml

comment on mechanisms of action. Sharpe and colleagues[26] found participants receiving ABM, compared to those in a placebo group, showed significant improvements in pain-related disability after the ABM. No significant improvements were found for pain, fear of pain, fear of re-injury, depression, anxiety, stress, anxiety sensitivity or pain self-efficacy. At 6-month follow-up (and after all participants had undergone 8 sessions of CBT) the ABM group, compared to the placebo group, showed significant improvements in disability and anxiety sensitivity, and marginally significant improvements in fear of re-injury. No other significant effects were found, and the mechanism of action could not be established as ABM was not found to change patterns of attentional bias across time. Schoth and colleagues[27] reported statistically and clinically significant change pre- to post-ABM in pain intensity, pain interference, anxiety, and depression. Assessed via the McGill Pain Questionnaire[28], significant changes pre- to post-ABM were shown for total pain, affective pain and miscellaneous pain, but not sensory-pain or evaluative pain. There was also no evidence that attentional biases changed across time.

One small RCT in adolescents with chronic pain randomised participants to either ABM, placebo training or waiting-list groups. No significant differences were found between the three groups in terms of pain, disability, pain catastrophising, anxiety, depression, or objective measures of physical functioning (sit-to-stand task and cardio wall tasks), and no evidence was found that ABM changed attentional bias or attentional control compared to placebo and waiting-list conditions.[29] Overall, while some studies have provided support for the benefits of ABM in adults, the evidence in regards to specific outcome variables is mixed and the mechanisms of action have yet to be established. Given the methodological shortcomings and small sample sizes of previous studies however, further research is needed to determine the therapeutic benefits of ABM for patients with chronic pain, along with the optimal form ABM should take and the impact of age on outcomes and intervention engagement. Considering the optimal form of ABM, a review of the broader cognitive bias modification (CBM) literature identified 12 published meta-analyses, 11 examining ABM specifically.[30] Three out of six analyses found the number of

For peer review only - http://bmjopen.bmj.com/site/about/guidelines.xhtml

sessions to be a significant moderator of CBM, and three out of nine analyses found the number of sessions to be a significant moderator of symptom outcomes. Although evidence is therefore mixed, it is nevertheless worth exploring whether number of sessions impacts patterns of attentional bias and pain-related outcomes in individuals with chronic pain.

This article presents a study protocol for a randomised, double-blind internet-delivered ABM intervention. The results of a pilot think aloud study informing the design of the intervention are also presented. The aim of this RCT is to assess the efficacy of internet-delivered ABM in improving pain and pain-related distress in people aged 16 –60 with CMSK, and will report a subgroup by treatment effects using the two age brackets of 16-24 and 25-60. While only limited evidence has been found for age as a moderator of CBM on bias change and symptoms,[30] it has been argued that the age of adolescence should be extended from 19 to 24 as the latter corresponds more closely to adolescent growth and popular understandings of this life phase.[31] Furthermore, although use of the internet has been growing among all age groups, there are nevertheless still some differences in internet use and activity according to age (e.g., in the UK 1% of those aged 16 to 24 do not use the internet, compared to 7% aged 45-54),[32] thus warranting a separate consideration of adolescent and adult participants. Overall, a diverse sample in terms of age, socioeconomic status, and geographical region will be recruited, thus increasing generalisability of results to the CMSK population. The primary outcome measure is pain interference. Secondary outcome measures are pain intensity, state and trait anxiety, depression, pain-related fear and sleep impairment, and engagement to and satisfaction with the online visual-probe training task. Dose effects will be explored via the inclusion of 10 versus 18 session conditions. It is hypothesised that participants in the intervention group, relative to those in the placebo group, will report significant reductions in pain interference, pain intensity, anxiety, depression, sleep impairment and pain-related fear at study endline. It is also hypothesised that dose will significantly moderate these findings, with greater effects in 18 compared to 10 sessions.

For peer review only - http://bmjopen.bmj.com/site/about/guidelines.xhtml

Methods and Analysis

Participants

Participants will be recruited via press announcements, social media, and patient organizations throughout the UK. Inclusion criteria will be: (a) aged between 16 to 60 years old, (b) experiencing any type of CMSK (i.e., any condition that involves pain lasting for more than three months and arises from bones, muscles and/or joints), (c) able to sit at a personal computer for forty minutes, (d) normal or corrected to normal vision, (e) access to the internet at least twice a week, (f) access to, and familiarity using, a Windows-based computer, (g) successful completion of primary school, and (h) living in the United Kingdom. Exclusion criteria will be (1) experiencing malignant CMSK (i.e., pain caused due to a tumour), (2) a diagnosis of other forms of comorbid chronic pain (e.g., chronic headache), and (3) a diagnosis of any psychiatric disorder, either currently or within the last 5 years. There are no restrictions placed on concomitant care and participants are not required to make any changes to current treatments they may be receiving.

Patient and Public Involvement

Participant burden of the intervention and outcome measures were assessed using individual interviews and informal feedback from patients participating in two pilot studies.[27 33] Patients will not be involved in recruitment of participants or conduct of the study. Results of this study will be disseminated to participants through presentation at client and community forums.

Study Design and Setting

The study is a double-blind randomised controlled trial (RCT) with four arms exploring the efficacy of online ABM training versus a placebo training condition theorised to offer no specific therapeutic benefits. The choice of comparator was selected as placebo training closely mimics attentional bias modification training, and is frequently used as a comparator in ABM studies (e.g.,[25 26 34 35]). The study will take place in a location of the participant's choice (which

For peer review only - http://bmjopen.bmj.com/site/about/guidelines.xhtml

we anticipate will typically be their home), which is accessed via personal computer connected to the internet.

The study design has a 2 x 2 x 3 experimental structure, with multiple non-commensurate dependent variables measuring pain intensity and interference, emotional functioning and pain-related disability. Treatment is a two-level factor (ABM, Placebo), number of Sessions is a two-level factor (10 sessions, 18 sessions), with measures recorded at three points in time (baseline, endline, and 6-month follow-up). Measures in the 18 session groups (18AMG and 18 PTG) will also be taken after session 10.

Randomisation and Sample Size

Block randomisation of size four, performed via a randomisation command in the Lifeguide platform[36], will be used to sequentially randomise eligible consecutive consenting participants to one of four groups: (i) 10-session attentional modification group (10AMG); (ii) 10-session placebo training group (10PTG); (iii) 18-session attentional modification group (18AMG); or (iv) 18-session placebo training group (18PTG). This blocked randomisation will be performed separately for those aged 16-24 and those aged 25-60. An allocation concealment mechanism will be used to ensure neither participants nor researchers know the study group to which the next participant will be assigned. This will be automatically performed within the Lifeguide platform (see below), with participants provided access to appropriate AMG or PTG sessions. Participants randomised to either 10-session condition will receive ten 35-minute online sessions across a four-week period, while those randomised to either 18-session condition will receive eighteen 35-minute online sessions across an eight-week period. All online sessions will be administered twice a week on a separate set of days (Monday-Thursday, Tuesday-Friday). Participants randomised to either AMG condition will receive attentional training via the modified training version of the probe-classification visual probe-task, while those randomised to either PTG condition will receive placebo training via the standard version of the probe-classification visual-probe task. To maintain the overall quality and legitimacy of the

For peer review only - http://bmjopen.bmj.com/site/about/guidelines.xhtml

intervention, code breaks (i.e., unblinding) will only occur in exceptional circumstances. Adherence will be monitored electronically via Lifeguide.

Our former lab-based proof-of-concept ABM intervention found reductions in pain intensity and pain interference pre- to post-ABM were associated with large effect sizes[27], although differences in methodology including delivery (online vs lab-based) and number of sessions warrants a more conservative estimate. On this basis, the study will recruit a minimum of N = 100 with 1:1:1:1 randomisation. This study is powered primarily to establish treatment efficacy for the primary outcome variable of pain interference by considering baseline and measures after session 10 between those randomised to an attentional modification arm and those randomised to a placebo arm. Randomisation should ensure baseline comparability on measures between these two groups, and a difference in beneficial outcomes between these groups will be modelled using a linear mixed model. For an assumed correlation of 0.7 between baseline and end of session 10 measures a total of $n = 40$ per group (i.e. 20 per randomized arm) would be needed to detect small to moderate standardized intervention differences (Cohen's $d = 0.25$) with at least 80% power (alpha = 0.05, two-sided). The recruitment strategy includes a 20% oversampling to mitigate a loss of power from loss to follow-up

Procedure

Interested individuals will contact the researchers on the details provided in study advertisements and will be assessed against the inclusion and exclusion criteria via telephone interview. If eligible, they will receive a copy of the participant information sheet (PIS) detailing the requirements and procedure of the study, along with a link to the study website which provides further details on ABM and the research team. If the individual consents to take part they will be required to create an online ABM account. During registration, participants will select their preferred days for training, which will be either (i) Monday/Thursday or (ii) Tuesday/Friday.

For peer review only - http://bmjopen.bmj.com/site/about/guidelines.xhtml

ATTENTIONAL BIAS MODIFICATION PROTOCOL

Session 1 is the baseline session, and includes the first assessment visual-probe task assessing attentional biases prior to beginning the intervention, along with the questionnaire battery assessing the primary and secondary outcome measures. Randomisation will take place following session 1, where participants will be informed of the length of their training (i.e., four or eight weeks). Participants randomised to either of the ten session conditions will complete four pictorial and four linguistic training sessions, while those randomised to either of the 18 session conditions will complete eight pictorial and eight linguistic training sessions (baseline and endline assessment sessions comprise the remainder of the 10/18 sessions). Participants' attentional biases will be re-assessed at endline (i.e., during the final session 10/18) using the same assessment version of the visual probe task (i.e., featuring the same stimuli) used at baseline and the same questionnaire battery. Participants randomised to either of the 18 session conditions will also have their attentional biases re-assessed in session 10 after their training session.

Participants' satisfaction with the online training will also be assessed. Six months after the last session, participants will be invited to again complete the online assessment visual-probe task (same as baseline and final session 10/18) and questionnaire battery. Within two weeks of the completion of the six-month follow-up session, participants will be invited to take part in a brief telephone semi-structured interview including 15 questions spread across five thematic sections: (a) motivation and expectations about the study, (b) experience of the online study – person, environmental and lifestyle parameters, (c) experience of the visual-probe task, (d) understanding of the process of change, and (e) the visual-probe task as a potential therapeutic tool in pain management. The study flow is presented in Table 1.

For peer review only - http://bmjopen.bmj.com/site/about/guidelines.xhtml

ATTENTIONAL BIAS MODIFICATION PROTOCOL

Table 1. Schedule of enrolment, intervention, and assessments.

	STUDY PERIOD					
	Enrolment	Post-allocation				
**TIMEPOINT****	$-t_1$	t_1 Baseline	t_2 Training	t_3 Evaluation	t_4 Follow-up	t_5 Study exit
**ENROLMENT:**						
**Eligibility screen**	X					
**Informed consent**	X					
**Allocation**		X				
**INTERVENTIONS:**						
Attentional bias modification training			X			
**ASSESSMENTS:**						
Demographic questionnaire		X				
EOT		X	X	X	X	
BPI-SF		X		X	X	
HADS		X		X	X	
STAI		X		X	X	
MOS-SS		X		X	X	
FOP-III		X		X	X	
SOT				X		
Interview						X

T1 = Baseline phase including standard assessment visual-probe task and questionnaires. Also includes randomization to groups.
T2 = Training phase including training visual-probe tasks.
T3 = Evaluation phase including standard assessment visual-probe task and questionnaires. Participants randomized to 18 session conditions also complete evaluation phase during session 10.
T4 = Six-month follow-up including standard assessment visual-probe task and questionnaires.
T5 = Telephone interview within two weeks of T4.

Note. EOT, Engagement with the Online Training questionnaire; BPI-SF, Brief Pain Inventory-Short Form; HADS, Hospital Anxiety and Depression Scale; STAI, State-Trait Anxiety Inventory; MOS-SS, Medical Outcome Study –Sleep Scale, FOP-III, Fear of pain questionnaire III; SOT, Satisfaction with the online treatment questionnaire

To retain as many participants as possible, reminder emails will be sent to participants 24 hours before each scheduled session becomes available, and also at 13:00 on the scheduled session date should the participant not have already logged into their iABMT account. An

For peer review only - http://bmjopen.bmj.com/site/about/guidelines.xhtml

additional reminder email will be sent one week before their 6-month follow-up session is ready. Participants will also be sent encouragement emails informing them when they have reach specific milestones (i.e., successfully completing 5 sessions for those randomised to complete 10 sessions; successfully completing 6 and 12 sessions for those randomised to 18 sessions). Participants will also have the option of receiving an SMS message on their mobile phone at 09:00 on the morning of each scheduled session.

The Intervention

The intervention will be hosted using LifeGuide and iSurvey platforms, both developed by the University of Southampton. LifeGuide is an open-source software which allows researchers to create online interventions.[36] iSurvey is a survey generation tool which allows researchers to create and disseminate questionnaires online. Participants will register for the intervention using LifeGuide, which will also host the majority of the questionnaires. iSurvey will be used to host the visual-probe tasks, along with the Engagement with Online Training questionnaire and the Brief Pain Inventory[37] that appear at the end of every visual-probe task session. The flow of sessions is provided in Figure 2, and examples of webpages are shown in Figure 3. On loading the intervention homepage, the individual is provided with a brief welcome to the website and rationale for the intervention. A recent review of cognitive bias meta-analyses concluded that effect sizes were smaller when biases were modified remotely compared to laboratory-based training.[30] We therefore decided to provide information on the rationale behind ABMT in order to increase motivation and reduce attrition as much as possible, which also mirrors clinical practice that involves educating patients into chronic pain and the psychological interventions used in its management. Individuals are able to either create an iABMT account or log into their existing account. Before any session participants are asked to indicate whether they have enough time and are ready to begin the session. Participants are only permitted to complete one session per day. Should a participant complete their missed session on a subsequently scheduled date (e.g., completing their scheduled session from Monday on Thursday instead), all

For peer review only - http://bmjopen.bmj.com/site/about/guidelines.xhtml

remaining sessions are shifted back accordingly. This process retains the structure of the intervention.

Guidance has been informed by research showing attention to be a limited capacity resource[38 39], sleepiness to negatively influence concentration and performance[40], alcohol to impair speed of information processing and cognition[41], energy drinks to be associated with restlessness and nervousness[42], caffeine to improve alertness[43], large meals to be associated with impairments in cognitive functioning[44], and hunger to influence patterns of attention.[45 46] Participants are provided with guidance to ensure concentration and comfort is maintained throughout each session: (1) allowing enough time for the session with no anticipated distractions, (2) adjusting screen brightness to match the brightness of the environment, (3) closing distracting applications such as Twitter and Facebook, (4) avoiding completing the session when tired, (5) not consuming alcohol at least 3 hours prior to the session, (6) caution in the use of energy drinks which may cause restlessness, (7) if the participant is a coffee or tea drinker, suggesting a cup 30 minutes prior to the session, and (8) avoiding completing the session when hungry or directly after a meal.

Visual-Probe Tasks. The flow of visual-probe sessions is illustrated in Figure 2. In total there will be eight linguistic and eight pictorial probe-classification visual-probe training tasks used in the study, each with different stimuli. A complete description of the stimuli including ratings of valence, arousal, pain intensity, written frequency, and low-level features, is provided in Supplementary Material 1. To briefly summarise, each linguistic visual-probe task includes 32 threatening/neutral and 16 neutral/neutral word pairs. Each task includes eight threatening words from each of the following categories: sensory-pain, affective-pain, health-threat and general-threat. Each pictorial task includes 32 threatening/neutral and 16 neutral/neutral image pairs. Each task includes eight threatening images from the following categories: musculoskeletal pain, facial expressions of pain, health-threat and general-threat. A total of 384 trials are included in each task, with each stimuli-pair presented four times for 500 ms and four times for 1250 ms.

For peer review only

ATTENTIONAL BIAS MODIFICATION PROTOCOL

Within each exposure duration, each stimulus appears twice in the upper location of the screen, and twice in the lower location.

A separate linguistic visual-probe task will be used in the three assessment sessions, featuring different stimuli to the linguistic training visual-probe tasks. The use of separate assessment and training stimuli is necessary to establish whether training effects generalise to novel stimuli not used in the actual training. The same assessment stimuli will be used in all assessment sessions. The ABM training version of the visual-probe task to be completed by AMG groups includes three main stages per trial (Figure 1). Following a 500 ms initial fixation point, a stimulus pair are presented in distinct locations (e.g., above and below the initial fixation point) for either 500 or 1250 ms. The stimulus pair may be either words or images, although critically includes one threat-related and one neutral stimulus. 500 and 1250 ms stimuli presentation times are used as these are the most common stimuli presentation times adopted in chronic pain visual-probe studies (e.g., [18 20 47]), and will allow us to closely compare our results to those of former research. Immediately following their presentation, the stimuli disappear and a probe appears (either *'p'* or *'q'*) in the location of the neutral stimulus. Participants use corresponding keys on their keyboard to indicate the probe letter as quickly and as accurately as possible. In the standard version of this paradigm completed by PTG groups and used in the assessment sessions, the probe replaces the neutral and threat-related stimulus an equal number of times. The probe-classification version of the visual-prove task is argued to encourage a more even monitoring of the display than the probe-position version (i.e., which requires participants to indicate the location of a dot, either left/right or up/down), as for the latter participants can adopt biased monitoring strategies favouring one particular region over the other.[48] As any such biases in monitoring many affect training, we will use the probe-classification version of the visual-probe task.

Outcome Measures

For peer review only - http://bmjopen.bmj.com/site/about/guidelines.xhtml

ATTENTIONAL BIAS MODIFICATION PROTOCOL

The primary outcome measure of pain interference will be assessed by the Brief Pain Inventory – Short Form (BPI-SF[37]). Pain interference will be scored as the mean of seven interfernce items (i.e., general activity, walking, work, mood, enjoyment of life, relations with others, and sleep). Following guidance in the Brief Pain Inventory User Guide[49] and also IMMPACT recommendations for assessing pain in clinical trials,[50] we will include individual assessments of 'worst', 'least', 'average' and 'now' (i.e., current) pain intensity as secondary outcome measures. Additional secondary outcome measures of state and trait anxiety will be assessed by the State Trait Anxiety Inventory (STAI [51]), depression by the Hospital Anxiety and Depression Scale (HADS[52]), pain-related fear by the Fear of Pain Questionnaire III (FOP-III[53]), sleeping impairment by the Medical Outcome Study – Sleep Scale (MOS-SS[54]), and engagement to and satisfaction with the online visual-probe training task by the Satisfaction with the Online Training questionnaire (SOT[55]) and the Engagement with the Online Training questionnaire (EOT). A demographic questionnaire developed by the researchers will be used at baseline to collect information on participants' age, gender, ethnicity, socio-economic status, place of residence, and pain characteristics. Aside from the SOT and EOT, questionnaires in the 10 session groups (10AMG and 10PTG) will be administered to participants at baseline, after 10 sessions, and at six-month follow-up. In the 18 session groups (18AMG and 18PTG) questionnaires will be administered to participants at baseline, after 10 sessions, after 18 sessions, and at six-month follow-up. At endline for each participant we will also assess whether the participant and researchers became aware at any point prior to endline which group they had been allocated to. Full details on each outcome measure are provided in Supplementary Material 2.

Think Aloud Study

A qualitative think aloud study[33] including a short semi-structured interview was conducted following the development of the initial version of the intervention with the aim to understand participants' first impressions and attitudes towards the intervention, and to collate

For peer review only - http://bmjopen.bmj.com/site/about/guidelines.xhtml

feedback that could be used to make improvements. Details and outcomes of this study are provided in Supplementary Material 3.

Analyses of the RCT Data

Quantitative Analyses

A full and detailed Statistical Analysis Plan will be written prior to trial closure. In brief, the data analysis will be performed on an intention-to-treat (ITT) basis to provide an unbiased estimate of the treatment effect.[56] Per protocol analyses will also be performed (comprising those correctly randomized, without protocol violations, and providing complete data on the primary outcome measure). Sweetman and Doig[57] have identified five types of protocol violations, including violations in (i) enrollment (i.e., failure to correctly apply eligibility criteria resulting in the enrolment of an inappropriate participant), (ii) randomization (i.e., violation of intended randomization sequence), (iii) study intervention (i.e., dosing, timing or delivery errors attributable to members of the research team), (iv) patient compliance (i.e., participants failing to comply with the trial protocol or other requirements of participation in the trial, such as skipping scheduled appointments or sessions), and (v) data collection (i.e., a failure by the research team to comply with pre-specific trial guidelines for data collection and/or outcome evaluation due to avoidable reasons). Any such violations will be clearly reported.

Analyses undertaken will include:

(a) The primary analysis on pain interference will utilize the initial 2 by 2 by 2 structure comprising Stage (baseline, 10 weeks), randomized Treatment (AMBT, Placebo), and randomized number of Sessions (10, 18). This comparison will be extended to compare 10AMG and 10PTG after 10 sessions, and to compare 18AMG and 18PTG after 10 sessions.

(b) An analysis of pain interference will utilize the 2 by 2 by 3 structure comprising randomised Treatment (AMBT, Placebo), randomized number of Sessions (10, 18), and Stage (baseline, endline, follow-up).

For peer review only - http://bmjopen.bmj.com/site/about/guidelines.xhtml

(c) An assessment of change in pain interference, both within and between groups, for 18AMG and 18PTG, between the end of session 10 and endline.

(d) The four randomized arms compared against one another on pain interference at each of baseline, after session 10, endline, and follow-up.

(e) An age (16 – 24; 25 – 60) subgroup analysis using an age subgroup by treatment effect interaction. These analyses will be an extension of (a), (b), and (c) to incorporate age as a subgroup factor.

The same analyses would apply to the secondary outcome measures. The data will be represented descriptively and graphically by arm, and by treatment and age, and standardized effect sizes reported.

Linear mixed model with random intercepts will be used to analyze the data with, as appropriate, Treatment (AMBT, Placebo), number of Sessions (10, 18) and assessment stage as fixed factors. This approach avoids listwise deletion which would apply in a repeated measures ANOVA approach. The linear mixed modelling approach uses all of the available data and adheres to ITT. As a secondary analysis we will used multiple imputation chained equations (MICE) to impute outcome data[58 59] and use these imputed data sets in the linear mixed model. Pairwise comparisons will follow the same procedure. An assessment of the sensitivity of findings to missing data mechanisms will be undertaken.[60 61] Specifically, we will use the strategy discussed by Morris and colleagues[61] whereby multiply imputed values are altered by a randomised group dependent value prior to analysis. We will run this sensitivity analysis over a factorial arrangement of group dependent values constraining any altered imputed values to be in the valid response range. The instance of the group dependent values both being equal to zero will correspond to multiple imputation under MAR and non-zero values would reflect deviations away from this assumption. The analysis plan would apply to the primary outcome measure and secondary outcome measures.

For peer review only - http://bmjopen.bmj.com/site/about/guidelines.xhtml

ATTENTIONAL BIAS MODIFICATION PROTOCOL

Similar to former studies[25 27] the Reliable Change Index (RCI)[62] will be used to assess primary and secondary outcome measures for clinically significant changes. Here, a clinically significant result is a post-treatment score falling outside two standard deviations of the mean population of interest (in this study, two standard deviations outside pre-treatment means). In contrast to tests of statistical significance comparing group means, tests of clinical significance explore effects of treatment on the individual.[63]

For visual-probe data, and as per former research[19 27], practice and incorrect trials will be removed prior to analyses. Box and whisker plots for overall data will be used to reveal overall outliers, which will be removed. Following this, mean response times will be computed for each participant, with any response >3SD from their individual mean also removed as outliers. This process ensures extremely quick or slow responses do not unduly bias the results, which are typically removed when cleaning and screening visual-probe data in pain-related research (e.g., [18 64 65 66 67]). An attentional bias index will then be computed for each stimulus and presentation time condition using the following equation: $(TuPl - TlPl)+(TlPu - TuPu))/2$. Here T is the threatening stimulus, P is the probe, u is the upper position, and l is the lower position. The attention capturing quality of threatening stimuli is measured by subtracting the mean probe classification time for congruent trials from the mean probe classification time of incongruent trials.[21]

Split-half reliability will be computed via a bootstrap procedure, by randomly splitting the total number of trials in two halves, such that each half has the same number of congruent and incongruent trials. Attentional bias scores will be computed for each half and the correlation between them calculated across participants. This procedure will be repeated 100 times, and an average split-half correlation computed.

Qualitative Analyses

Qualitative analysis will be used to detail participants' experiences completing the iABMT intervention. Specifically, thematic analysis using inductive coding will be used to

For peer review only - http://bmjopen.bmj.com/site/about/guidelines.xhtml

identify themes present in the data obtained from the semi-structured interviews and the two open-ended questions included in the Satisfaction with the Online Training questionnaire. Guidelines provided by Braun and Clarke[68] will be followed. Following transcription of all interviews verbatim, initial codes will be generated. Potential themes and sub-themes will then be identified from the initial coding, which subsequently will be reviewed and refined. The validity of the themes in relation to the data set will also be considered. Each theme and sub-theme will then be named and defined, and a thematic map of the data finalized. Examples from the data will be illustrated for each theme and sub-theme in the final report.

Ethics and Dissemination

This study has been approved by the University of Southampton Research Ethics Committee (ERGO ID: 26486), and the study protocol registered with ClinicalTrial.gov (NCT02232100) with a planned start date of September 2020 and planned end date of September 2022. Ethical approval will be sought before any protocol modifications are made and the study protocol will be updated at ClinicalTrial.gov. Participants will provide informed consent prior to taking part in the study. All data will remain confidential and stored on password-protected systems and databases. Lifeguide and iSurvey servers both use HTTPS connections for security purposes. All data will be anonymised prior to dissemination, and personally identifiable information not known to anyone other than the researchers. Participants may withdraw from the intervention at any point by contacting the researchers via email or telephone, or by simply opting not to log into their iABMT account for their scheduled sessions. Participants are also encouraged to contact the researchers should they experience any distress or discomfort completing the intervention. As per University policy, any adverse events, harms or complaints arising from participation in the intervention will be reported to the University of Southampton Research Governance Office. Findings will be published in the most relevant, high-impact peer-reviewed journals and presented at relevant conferences. Lay reports will be written for interested pain charities and patient support groups.

For peer review only - http://bmjopen.bmj.com/site/about/guidelines.xhtml

List of figures and supplementary files

Table 1: Schedule of enrolment, intervention, and assessments.

Figure 1. A graphical representation of a typical visual-probe task training trial with linguistic stimuli.

Figure 2. Flow of the visual-probe task sessions for the 10-week and 18-week groups in the study

Figure 3. Examples of Lifeguide webpages included in the iABMT intervention.

Supplementary Material 1 – Stimuli development and properties

Supplementary Material 2 – Outcome Measures

Supplementary Material 3 – Think Aloud Study

For peer review only

References

1. Sambrook P, Taylor T, Schrieber L, et al. *The Musculoskeletal System: Basic Science and Clinical Conditions*. Second ed: Churchill Livingstone, Elsevier, 2010.

2. Cimmino MA, Ferrone C, Cutolo M. Epidemiology of chronic musculoskeletal pain. Best Practice & Research Clinical Rheumatology 2011;**25**(2):173-83 doi: 10.1016/j.berh.2010.01.012.

3. King S, Chambers CT, Huguet A, et al. The epidemiology of chronic pain in children and adolescents revisited: a systematic review. Pain 2011;**152**(12):2729-38 doi: 10.1016/j.pain.2011.07.016.

4. Tüzün EH. Quality of life in chronic musculoskeletal pain. Best Practice & Research Clinical Rheumatology 2007;**21**(3):567-79 doi: http://dx.doi.org/10.1016/j.berh.2007.03.001.

5. Breivik H, Collett B, Ventafridda V, et al. Survey of chronic pain in Europe: prevalence, impact on daily life, and treatment. Eur J Pain 2006;**10**(4):287-333 doi: 10.1016/j.ejpain.2005.06.009.

6. Bair MJ, Wu J, Damush TM, et al. Association of depression and anxiety alone and in combination with chronic musculoskeletal pain in primary care patients. Psychosom Med 2008;**70**(8):890-7 doi: 10.1097/PSY.0b013e318185c510.

7. Blyth FM, Noguchi N. Chronic musculoskeletal pain and its impact on older people. Best Practice & Research Clinical Rheumatology 2017;**31**(2):160-68 doi: 10.1016/j.berh.2017.10.004.

8. Harrison L, Wilson S, Munafò MR. Exploring the associations between sleep problems and chronic musculoskeletal pain in adolescents: a prospective cohort study. Pain research and management 2014;**19**(5):e139-e45 doi: 10.1155/2014/615203.

9. Flatø B, Aasland A, Vandvik I, et al. Outcome and predictive factors in children with chronic idiopathic musculoskeletal pain. Clinical and Experimental Rheumatology 1997;**15**(5):569-77.

For peer review only

For peer review only - http://bmjopen.bmj.com/site/about/guidelines.xhtml

10. Gold JI, Mahrer NE, Yee J, et al. Pain, fatigue and health-related quality of life in children and adolescents with chronic pain. Clinical Journal of Pain 2009;**25**(5):407 doi: 10.1097/AJP.0b013e318192bfb1.

11. Vinall J, Pavlova M, Asmundson GJ, et al. Mental health comorbidities in pediatric chronic pain: A narrative review of epidemiology, models, neurobiological mechanisms and treatment. Children 2016;**3**(4):40 doi: 10.3390/children3040040.

12. Pincus T, Morley S. Cognitive-processing bias in chronic pain: a review and integration. Psychol Bull 2001;**127**(5):599 - 617 doi: 10.1037/0033-2909.127.5.599.

13. Todd J, Sharpe L, Johnson A, et al. Towards a new model of attentional biases in the development, maintenance, and management of pain. Pain 2015;**156**(9):5189-1600 doi: 10.1097/j.pain.0000000000000214.

14. Vlaeyen JWS, & Linton, S. J. Fear-avoidance and its consequences in chronic musculoskeletal pain: a state of the art. Pain 2000;**85**(3):317 - 32.

15. Liossi C, Schoth DE, Bradley BP, et al. Time-course of attentional bias for pain-related cues in chronic daily headache sufferers. European Journal of Pain 2009;**13**(9):963-69 doi: http://dx.doi.org/10.1016/j.ejpain.2008.11.007.

16. Haggman SP, Sharpe, L. A., Nicholas, M. K., & Refshauge, K. M. Attentional biases toward sensory pain words in acute and chronic pain patients. The Journal of Pain 2010;**11**(11):1136 - 45.

17. Sharpe L, Dear BF, Schrieber L. Attentional biases in chronic pain associated with rhumatoid arthritis: Hypervigilance or difficulties disengaging? J Pain 2009;**10**(3):329 - 35 doi: 10.1016/j.jpain.2008.10.005.

18. Schoth DE, Beaney R, Broadbent P, et al. Attentional, interpretation and memory biases for sensory-pain words in individuals with chronic headache. British Journal of Pain 2019;**13**(1):22 - 31 doi: 10.1177/2049463718789445.

For peer review only

19. Schoth D, Liossi C. Specificity and time-course of attentional bias in chronic headache: a visual-probe investigation. Clinical Journal of Pain 2013;**29**(7):583-90 doi: 10.1097/AJP.0b013e31826b4849.

20. Schoth DE, Liossi C. Attentional bias towards pictorial representations of pain in individuals with chronic headache. Clin J Pain 2010;**26**(3):244 - 50 doi: 10.1097/AJP.0b013e3181bed0f9.

21. Schoth DE, Delgado Nunes V, Liossi C. Attentional bias towards pain-related information in chronic pain; a meta-analysis of visual-probe investigations. Clin Psychol Rev 2012;**32**(1):13 - 25 doi: 10.1016/j.cpr.2011.09.004.

22. Todd J, van Ryckeghem DM, Sharpe L, et al. Attentional bias to pain-related information: A meta-analysis of dot-probe studies. Health Psychol Rev 2018;**12**(4):419-36.

23. Crombez G, Van Ryckeghem D, Eccleston C, et al. Attentional bias to pain-related information: a meta-analysis. Pain 2013;**154**(4):497-510 doi: 10.1016/j.pain.2012.11.013.

24. MacLeod C, Mathews, A., & Tata, P. Attentional Bias in Emotional Disorders. Journal of Abnormal Psychology 1986;**95**(1):15 - 20.

25. Carleton RN, Richter AA, Asmundson GJG. Attention Modification in Persons with Fibromyalgia: A Double Blind, Randomized Clinical Trial. Cogn Behav Ther 2011;**40**(4):279-90 doi: 10.1080/16506073.2011.616218.

26. Sharpe L, Ianiello M, Dear BF, et al. Is there a potential role for attention bias modification in pain patients? Results of 2 randomised, controlled trials. Pain 2012;**153**(3):722-31 doi: 10.1016/j.pain.2011.12.014.

27. Schoth DE, Georgallis T, Liossi C. Attentional bias modification in people with chronic pain: a proof of concept study. Cognitive Behaviour Therapy 2013;**42**(3):233-43.

28. Curriculum for a CCT in Anaesthetics (2010). Secondary Curriculum for a CCT in Anaesthetics (2010). https://www.rcoa.ac.uk/document-store/curriculum-cct-anaesthetics-2010.

For peer review only - http://bmjopen.bmj.com/site/about/guidelines.xhtml

29. Heathcote LC, Jacobs K, Van Ryckeghem DML, et al. Attention bias modification training for adolescents with chronic pain: a randomized placebo-controlled trial. Pain 2018;**159**(2):239 - 51 doi: 10.1097/j.pain.0000000000001084.

30. Jones EB, Sharpe L. Cognitive bias modification: A review of meta-analyses. J Affect Disord 2017;**223**:175-83 doi: 10.1016/j.jad.2017.07.034.

31. Sawyer SM, Azzopardi PS, Wickremarathne D, et al. The age of adolescence. The Lancet Child & Adolescent Health 2018;**2**(3):223-28 doi: 10.1016/s2352-4642(18)30022-1.

32. Ofcom. Adults: Media use and attitudes report 2019, 2019.

33. Hamilton F. Perspectives of individuals with chronic musculoskeletal pain towards an online attentional bias modification training programme. University of Southampton, 2015.

34. Hakamata Y, Lissek, S., Bar-Haim, Y., Britton, J.C., Fox, N.A., Leibenluft, E., Ernst, M., & Pine, D. S. Attention bias modification treatment: A meta-analysis towards the establishment of novel treatment for anxiety. Biol Psychiatry 2010;**68**:982-90 doi: 10.1016/j.biopsych.2010.07.021.

35. Mogoaşe C, David D, Koster EH. Clinical efficacy of attentional bias modification procedures: An updated meta-analysis. J Clin Psychol 2014;**70**(12):1133-57 doi: 10.1002/jclp.2208.

36. Williams S, Yardley L, Wills GB. A qualitative case study of LifeGuide: users' experiences of software for developing Internet-based behaviour change interventions. Health Informatics J 2013;**19**(1):61-75 doi: 10.1177/1460458212458915.

37. Cleeland CS, Ryan KM. Pain assessment: global use of the Brief Pain Inventory. Ann Acad Med Singapore 1994;**23**(2):129-38.

38. Kahneman D. *Attention and effort*. New Jersey: Prentice Hall, 1973.

39. Broadbent D. *Perception and communication*. Oxford: Pergamon, 1958.

40. Thomas M, Sing H, Belenky G, et al. Neural basis of alertness and cognitive performance impairments during sleepiness. I. Effects of 24 h of sleep deprivation on waking human

For peer review only

regional brain activity. Journal of Sleep Research 2000;**9**(4):335-52 doi: 10.1046/j.1365-2869.2000.00225.x.

41. Tzambazis K, Stough C. Alcohol impairs speed of information processing and simple and choice reaction time and differentially impairs higher-order cognitive abilities. Alcohol and Alcoholism 2000;**35**(2):197-201 doi: 10.1093/alcalc/35.2.197.

42. Reissig CJ, Strain EC, Griffiths RR. Caffeinated energy drinks—a growing problem. Drug and Alcohol Dependence 2009;**99**(1):1-10 doi: 10.1093/alcalc/35.2.197.

43. Zwyghuizen-Doorenbos A, Roehrs TA, Lipschutz L, et al. Effects of caffeine on alertness. Psychopharmacology 1990;**100**(1):36-39 doi: 10.1007/BF02245786.

44. Müller K, Libuda L, Terschlüsen AM, et al. A review of the effects of lunch: On adults' short-term cognitive functioning. Canadian Journal of Dietetic Practice and Research 2013;**74**(4):181-88 doi: 10.3148/74.4.2013.181.

45. Piech RM, Pastorino MT, Zald DH. All I saw was the cake. Hunger effects on attentional capture by visual food cues. Appetite 2010;**54**(3):579-82 doi: 10.1016/j.appet.2009.11.003.

46. Al-Shawaf L. The evolutionary psychology of hunger. Appetite 2016;**105**:591-95 doi: 10.1016/j.appet.2016.06.021.

47. Schoth DE, Liossi C. Specificity and time-course of attentional bias in chronic headache: a visual-probe investigation. Clin J Pain 2013;**29**(7):583-90 doi: 10.1097/AJP.0b013e31826b4849.

48. Mogg K, & Bradley, B. P. Some methodological issues in assessing attentional biases for threatening faces in anxiety: a replication study using a modified version of the probe detection task. Behav Res Ther 1999;**37**(6):595 - 604.

49. Cleeland CS. The brief pain inventory: user guide. Houston, TX: The University of Texas MD Anderson Cancer Center 2009:1-11.

For peer review only

For peer review only - http://bmjopen.bmj.com/site/about/guidelines.xhtml

50. Dworkin RH, Turk DC, Wyrwich KW, et al. Interpreting the clinical importance of treatment outcomes in chronic pain clinical trials: IMMPACT recommendations. J Pain 2008;**9**(2):105-21.

51. Spielberger CD, Gorsuch RL, Lushene RE. *State Trait Anxiety Inventory*. Palo Alto, California: Consulting Psychologists Press, 1970.

52. Zigmond AS, Snaith RP. The hospital anxiety and depression scale. Acta Psychiatr Scand 1983;**67**(6):361-70.

53. McNeil DW, Rainwater A. Development of the Fear of Pain Questionnaire-III. Journal of Behavioral Medicine 1998;**21**(4):389-410 doi: 10.1023/A:1018782831217.

54. Hays RD, Stewart AL. Sleep Measures. Measuring functioning and well-being. In: Steweart AL, JEJ W, eds. Measuring function and well-being; the medical outcome study approach. Duke: Duke University Press, 1992:235-59.

55. Richards D, Timulak L. Satisfaction with therapist-delivered vs. self-administered online cognitive behavioural treatments for depression symptoms in college students. British Journal of Guidance & Counselling 2013;**41**(2):193-207 doi: 10.1080/03069885.2012.726347.

56. Gupta SK. Intention-to-treat concept: a review. Perspectives in Clinical Research 2011;**2**(3):109 doi: 10.4103/2229-3485.83221.

57. Sweetman EA, Doig GS. Failure to report protocol violations in clinical trials: a threat to internal validity? Trials 2011;**12**(1):214.

58. Chan A-W, Tetzlaff JM, Gøtzsche PC, et al. SPIRIT 2013 explanation and elaboration: guidance for protocols of clinical trials. BMJ 2013;**346**:e7586 doi: 10.1136/bmj.e7586.

59. Groenwold RH, Donders ART, Roes KC, et al. Dealing with missing outcome data in randomized trials and observational studies. American Journal of Epidemiology 2012;**175**(3):210-17 doi: 10.1093/aje/kwr302.

For peer review only

For peer review only - http://bmjopen.bmj.com/site/about/guidelines.xhtml

60. White IR, Horton NJ, Carpenter J, et al. Strategy for intention to treat analysis in randomised trials with missing outcome data. BMJ 2011;**342**:d40.

61. Morris TP, Kahan BC, White IR. Choosing sensitivity analyses for randomised trials: principles. BMC Med Res Methodol 2014;**14**(1):11.

62. Jacobson NS, Truax P. Clinical significance: A statistical approach to defining meaningful change. Psychotherapy Research 1991;**59**:12-19 doi: 10.1037/0022-006X.59.1.12.

63. Zahra D, Hedge C. The reliable change index: Why isn't it more popular in academic psychology? Psychology Postgraduate Affairs Group Quarterly 2010;**76**(76):14-19.

64. Schoth DE, Parry L, Liossi C. Combined cognitive biases for sensory-pain and disability information in individuals with chronic headache. J Health Psychol 2018;**23**(12):1610-21 doi: 10.1177/1359105316664136.

65. Dear BF, Sharpe L, Nicholas M, et al. Pain-related attentional biases: The importance of the personal relevance and ecological validity of stimuli. J Pain 2011a;**12**(6):625-32.

66. Schoth DE, Williams S, Liossi C. Attentional bias for pain- and period-related symptom words in healthy women who experienced a recent painful period. Eur J Pain 2015;**19**(6):745-51 doi: 10.1002/ejp.597.

67. McDermott MJ, Peck KR, Walters AB, et al. Do episodic migraineurs selectively attend to headache-related visual stimuli? Headache: The Journal of Head and Face Pain 2013;**53**(2):356-64 doi: 10.1111/head.12011.

68. Braun V, Clarke V. Using thematic analysis in psychology. Qualitative Research in Psychology 2006;**3**(2):77-101 doi: 10.1191/1478088706qp063oa.

For peer review only

Authors' contributions: All authors have contributed to the development of the study protocol. CL, TG and DES conceived the project. CL and DES registered the trial and are responsible for recruitment and implementation of the protocol. TG was primary responsible for the development of the online materials, including development and validation of linguistic and pictorial stimuli used in the visual-probe tasks. TG also assisted in programming the Lifeguide components. JZ was responsible for programming the visual-probe tasks to be used in the intervention, and assisted in programming the Lifeguide components. FH conducted the think aloud study. PW was responsible for development of the statistical analysis plan. All authors contributed to the writing of this manuscript, and approve its final form. These same authors will be included on future publications presenting intervention results, and will have access to the cleaned study datasets. **Funding**: This work was supported by the Economic and Social Research Council (grant number: RES-000-22-4128). This funding source had no role in the design of this study and will not have any role during its execution, analyses, interpretation of the data, or decision to submit results. **Competing interests**: The authors declare that they have no competing interest.

For peer review only

For peer review only - http://bmjopen.bmj.com/site/about/guidelines.xhtml

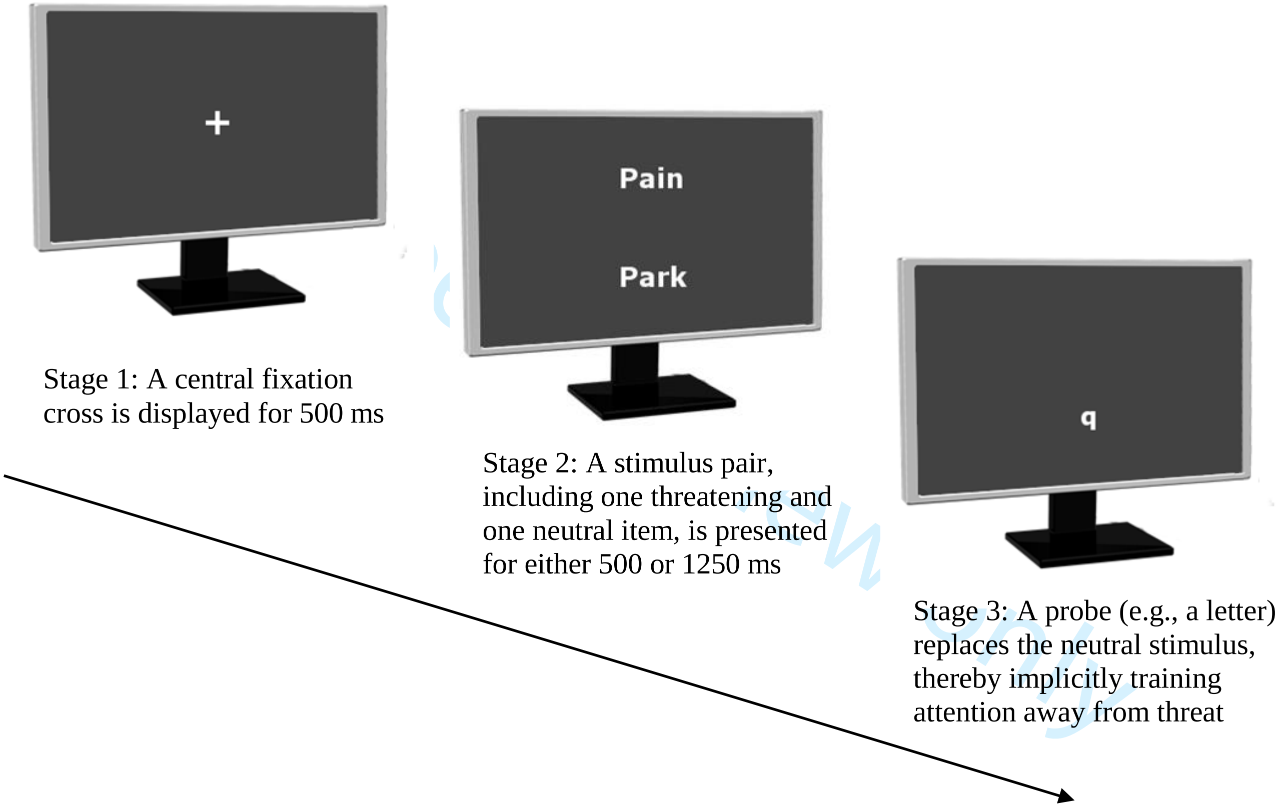

Figure 1. A graphical representation of a typical visual-probe task training trial with linguistic stimuli.

For peer review only - http://bmjopen.bmj.com/site/about/guidelines.xhtml

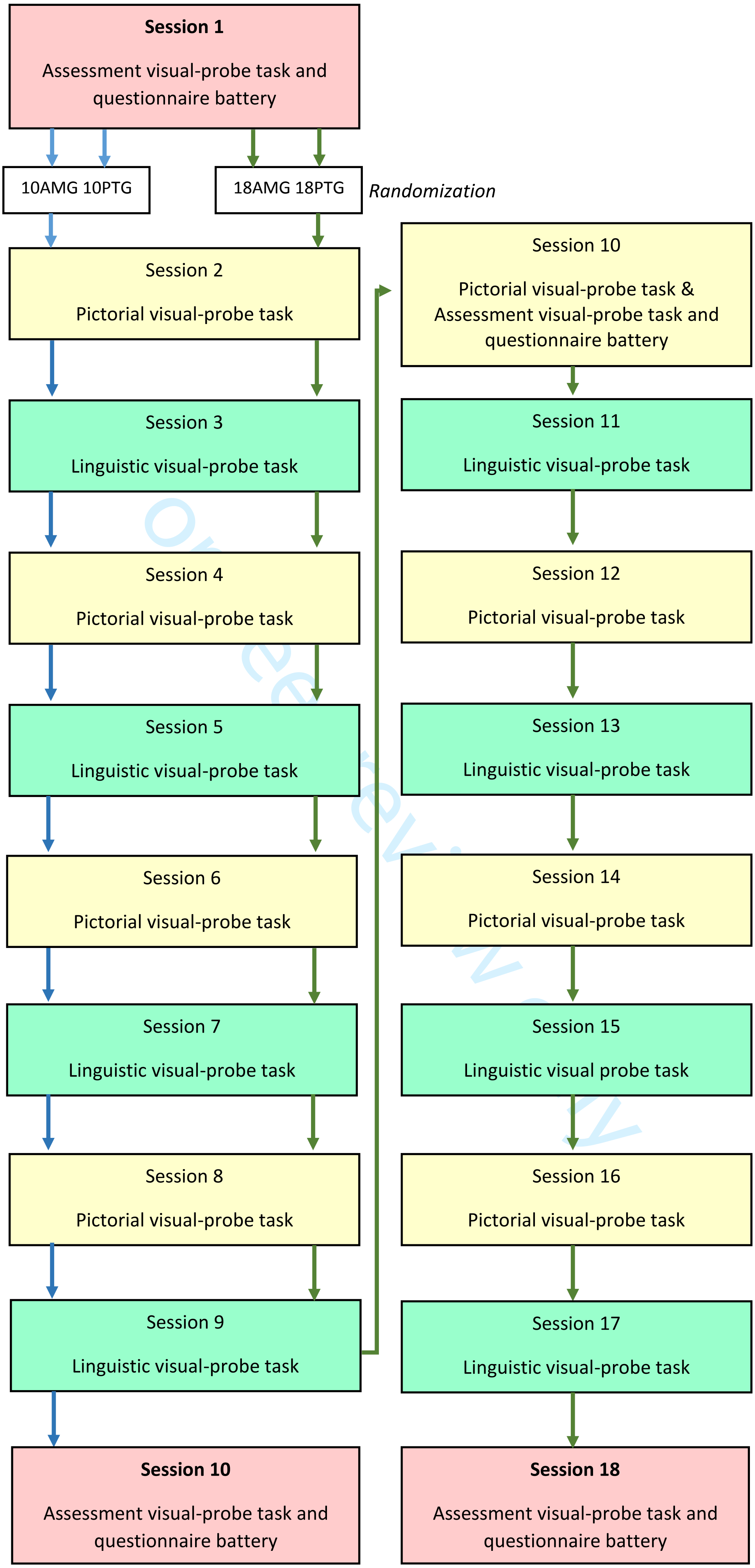

Figure 2. Flow of the visual-probe task sessions for the 10-week and 18-week groups in the study.

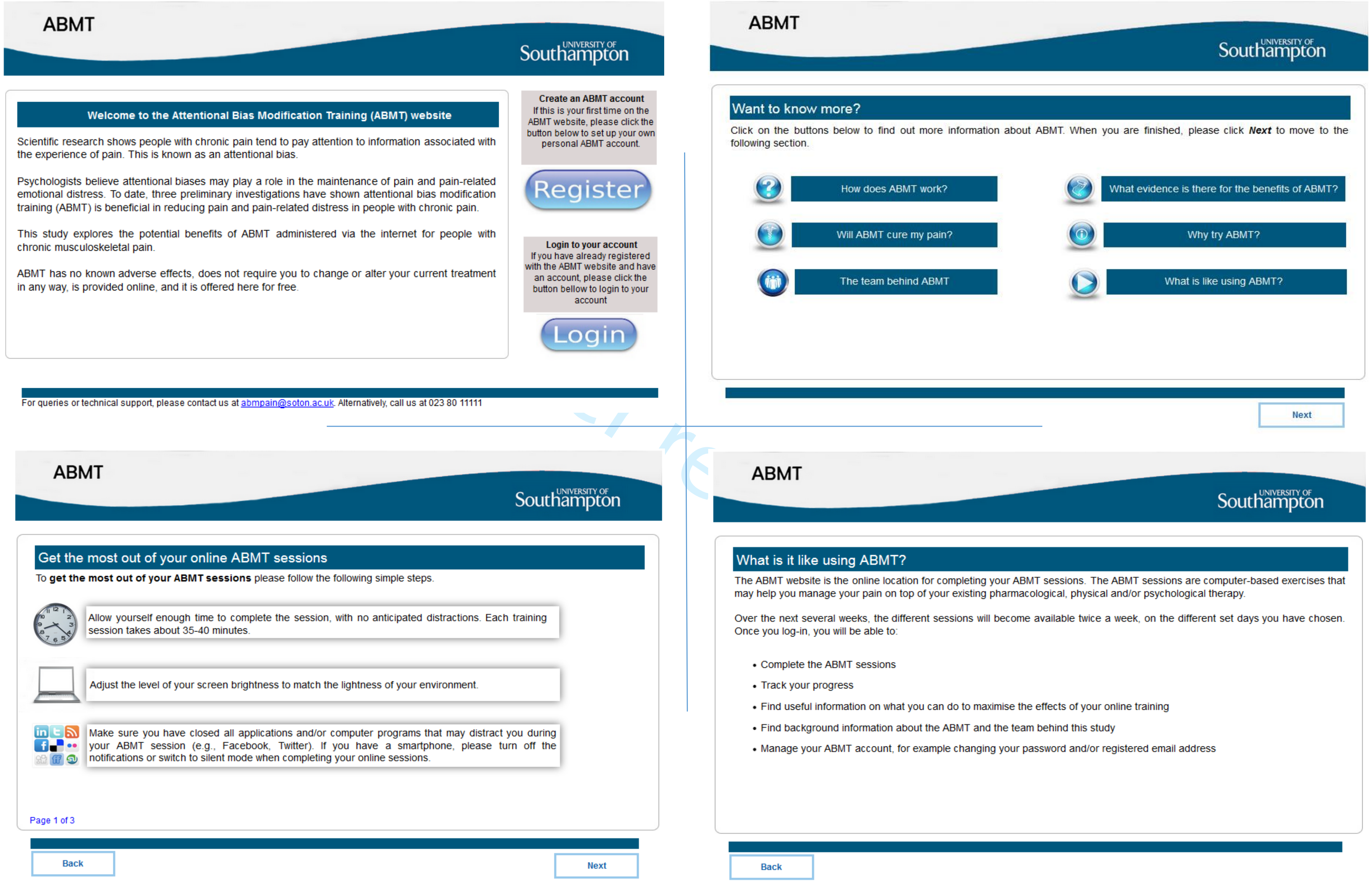

Figure 3. Examples of Lifeguide webpages included in the iABMT intervention.

Supplementary Material 1

Stimuli Development and Properties

A detailed description of the stimuli to be used in the iAMBT intervention is presented in this document. Linguistic stimulus properties and analyses on written frequency, valence, and arousal are first presented, followed by a description of pictorial stimuli properties and analyses of low-level features, valence, arousal and pain intensity. Following this, comparisons of stimuli properties between visual-probe sessions are then presented for linguistic and pictorial stimuli.

Linguistic stimuli

Properties. Linguistic and pictorial versions of the visual-probe task will be used. Each linguistic visual-probe task will include 32 threatening/neutral and 16 neutral/neutral word pairs. Threatening words stem from the following categories, with 8 words from each included in each visual-probe task: (i) sensory-pain words reflecting the sensory dimension of pain (e.g., *shooting*; *burning*; *aching*); (ii) affective-pain words reflecting the unpleasantness of pain and negative emotions associated with pain (e.g., *hopeless, punishing, tormenting*); (iii) health-threat words reflecting threat to health and well-being (e.g., *hospital, doctor, stress*); and (iv) general-threat words reflecting physical and emotional sources of threat, including manmade threat (e.g., *gun*), natural threat (e.g., *fire*), social threat (e.g., *humiliation*), and emotional threat (e.g., *sad*). Neutral words (including the neutral counterparts of the threatening words) include non-threatening words unrelated to pain or suffering (e.g., *version*; *rector*; *monument*), which were matched with their threatening counterparts on Kucera-Francis written frequency of use in the English language[1] and word length. Matching on written frequency minimise the chances of participants preferentially processing one stimulus due to its high frequency of use (e.g., faster recognition) when displayed simultaneously with another stimulus[2 3], while matching word length also minimise the chances of participants attending towards a stimulus due to its size.

In total, 864 words have been collected from previous studies exploring and modifying biases in pain and/or anxiety (i.e.,[4-8]), including words from the McGill Pain Questionnaire.[9]

For peer review only - http://bmjopen.bmj.com/site/about/guidelines.xhtml

Matching of threatening and neutral words was achieved via the MRC Psycholinguistic database[10], an online psycholinguistic tool that generates words with specific lexical characteristics. The database includes a dictionary of 150,837 English words providing syntactic, semantic and orthographical properties of each word.[11] Valence and arousal can influence attentional allocation to external stimuli.[12 13] A computerised version of the SAM task[14] was therefore used to collect ratings on valence and arousal for each word in a sample of sixteen healthy, pain-free participants (M_{age} = 25.9, SD = 4.08, range: 20 – 33 years; 8 female). All words were randomly presented to participants for 3 seconds each. Following each word, two 9-point SAM scales were presented, one for valence (1 = low pleasure to 9 = high pleasure) and one for arousal (1 = low arousal to 9 = high arousal). As per former chronic pain attentional bias research (e.g.,[5 15 16]), stimuli categories were assessed but not matched on valence and arousal, as differences are expected to exist between categories.

Analyses and results: written frequency, valence, and arousal. Analyses comparing properties between the five word categories are presented here, while analyses comparing word properties between the nine linguistic visual-probe tasks are presented below. One-way ANOVA was conducted comparing Kucera-Francis written frequency (Table 1) across words in the five word categories, revealing no significant main effect, $F(4, 853) = 1.97$, $p = .10$.

A repeated-measures ANOVA was conducted comparing arousal ratings (Table 2) across the five word categories, revealing a significant main effect, $F(2, 25) = 18.78$, $p < .001$, $\eta p^2 = .56$. Pairwise comparisons revealed the neutral category had significantly lower arousal scores than sensory-pain (*Md* [mean difference] = 1.82, *SE* = 0.45, *p* = .001), affective-pain (*Md* = 2.36, *SE*= 0.46, *p* = .001), general-threat (*Md* = 2.20, *SE* = 0.47, *p* = .003) and health-threat (*Md* = 2.05, *SE* = 0.35, *p* < .001) categories. Statistically significant differences were not found between the arousal scores of the words included in the four threat-related categories.

For peer review only - http://bmjopen.bmj.com/site/about/guidelines.xhtml

ATTENTIONAL BIAS MODIFICATION PROTOCOL

Table 1

Mean Kucera-Francis Written Frequency per Word Category

Word category	*M*	*SD*
Sensory-pain	19.07	27.01
Affective-pain	17.28	20.63
General-threat	25.00	32.10
Health-threat	21.36	36.39
All neutral words	22.60	27.97

Table 2

Mean Arousal and Valence Ratings per Word Category

	Arousal		Valence	
Word Category	*M*	*SD*	*M*	*SD*
Sensory-pain	3.80	1.85	3.95	0.83
Affective-pain	4.34	1.64	3.29	0.73
General-threat	4.19	1.78	3.35	0.79
Health-threat	4.03	1.32	3.48	0.68
Sensory-pain neutral counterparts	2.18	1.28	5.33	0.41
Affective-pain neutral counterparts	2.19	1.32	5.29	0.29
General-threat neutral counterparts	2.06	1.28	5.24	0.33
Health-threat neutral counterparts	2.05	1.19	5.19	0.36
Neutral/Neutral	1.95	1.32	5.29	0.33

A repeated-measures ANOVA was conducted comparing valence ratings (Table 2) across the five word categories, revealing a significant main effect, $F(2, 29) = 52.00$, $p < .001$, $\eta p^2 = .78$. Pairwise comparisons showed the neutral category had significantly higher pleasantness scores than sensory-pain (*Md* = 1.33, *SE* = 0.23, *p* <. 001) affective-pain (*Md* = 1.99, *SE* = 0.22, *p* < .001), general-threat (*Md* = 1.93, *SE* = 0.23, *p* <. 001) and health-threat (*Md* = 1.80, *SE* = 0.32, *p* < .001) categories. The words in the sensory-pain category were rated as more pleasant than

For peer review only - http://bmjopen.bmj.com/site/about/guidelines.xhtml

For peer review only

words included in the affective-pain (Md = 0.66, SE = 0.11, $p < .001$), general-threat (Md = 0.60, SE = 0.11, $p < .001$) and health-threat (Md = 0.47, SE = 0.11, $p = .01$) categories.

Pictorial Stimuli

Properties. Pictorial visual-probe tasks each include 32 threatening/neutral and 16 neutral/neutral image pairs. Threatening images stem from the following categories: (i) musculoskeletal pain images depicting people experiencing pain in muscles and/or joints, including images of people performing daily activities that can be considered painful and/or harmful for individuals with CMP (e.g., a person running; a person lifting a heavy weight with poor posture); (ii) facial expressions of pain, featuring a single model depicting a prototypical pain expression – closing the eyes or narrowing the eyelids, lowering the eyebrows, wrinkling the nose or raising the cheek, and partly opening the mouth or extending the lips[17], including horizontal lip stretching and/or oblique lip raising[18]; (iii) health-threat images reflecting threat to health and well-being (e.g., medication, wheelchair); and (iv) general-threat images reflecting physical and emotional sources of threat, including manmade threat (e.g., gun), natural threat (e.g., flood), and social threat (e.g., social isolation). Neutral images (including the neutral pairs of the threatening images) depict non-threatening objects (e.g., a car) and people performing every-day activities (e.g., a person reading a newspaper). In total, 768 images have been collected from previous studies exploring and modifying attentional biases in chronic pain (i.e.,[5] [19]), including online databases providing batteries of pictorial stimuli for research (i.e., International Affective Picture System database[20]; The Photograph Series of Daily Activities Short Version[21]; NimStim Set of Facial Expressions[22]).

Each threatening image depicting a person was paired with a neutral image featuring the same person, with the same clothes and background. Each threatening image depicting an object was matched with a neutral image depicting a similar, non-threatening object. Image pairs in the neutral/neutral condition were matched in the same manner. An equal number of images depicting male and female models were used within each image category. All images were full-

For peer review only - http://bmjopen.bmj.com/site/about/guidelines.xhtml

colour and the same size (i.e., 265 pixels wide x 250 pixels high). Each threatening image was matched with its neutral counterpart on complexity, luminance, contrast level, and colour saturation in blue, red and green channels via visual inspection[23]. Visual attention and gaze fixations are influenced by bottom-up variables, including the saliency of objects[24] and low-level features of visual scenes.[25 26] The additional matching rules for pictorial stimuli in the present study were therefore used to minimise the chances of participants preferentially processing one stimulus due to unnecessary variations in content. Using Adobe Photoshop CS6 Extended, the means and standard deviations for all parameters were extracted for evaluation. Luminance values range from 0 to 255, where 0 indicates no light intensity (i.e., no light emission; black) and 255 indicates the highest light intensity (i.e., absolute light emission; white). Contrast levels values range from -100 to +100, with values closer to +100 indicating more contrasted image. The intensity values for red, green and blue colours range from 0 to 255, where 0 indicates black (i.e., darkest extreme) and 255 white (i.e., lightest extreme) for each colour dimension.

Visual complexity was examined as the size of each compressed image file (.jpeg) in kilobytes under the assumption that more complex images would have higher values in kilobytes.[27] As with linguistic stimuli, a computerised version of the SAM task[14] was used to collect ratings on valence and arousal for each image. Musculoskeletal and facial expressions of pain categories were also evaluated on pain intensity using a 10 cm Visual Analogue Scale (VAS). Participants were required to rate the pain that the person in the image experienced. The scale ranged from 0 to 10, where 0 indicated '*no pain at all*' and 10 indicated '*excruciating pain*'. This rating aimed to assess the degree to which images included in the pain-related categories were perceived as depicting pain. Sixteen healthy, pain-free participants (M_{age} = 24.8, SD = 4.56, range: 20 – 33 years; 8 female) were recruited to provide ratings on valence, arousal, and pain intensity (this was a separate sample to that recruited to provide linguistic stimuli ratings).

Analyses and results: Low-level features. Analyses comparing properties between the five image categories on low-level features (Table 3) are presented here, while analyses

For peer review only - http://bmjopen.bmj.com/site/about/guidelines.xhtml

comparing word properties between the eight pictorial visual-probe tasks are provided below. Considering low-level features, one-way ANOVAs revealed no significant main effects for complexity, $F(4,763) = 2.18$, $p = .70$, or luminance, $F(4,763) = 2.31$, $p = .06$. A significant main effect was found for red saturation, $F(4,763) = 5.19$, $p < .001$, $\eta p^2 = .03$. Pairwise comparisons revealed images in the musculoskeletal-pain category had significantly less red saturation than images in the facial expressions of pain (*Md* = 24.24, *SE* = 7.89, $p = .022$) and neutral categories (*Md* = 19.04, *SE* = 5.91, $p = .013$). Images in the general-threat category had significantly less red saturation than facial expressions of pain (*Md* = 23.96, *SE* = 7.89, $p = .025$) and neutral categories (*Md* = 18.76, *SE* = 5.91, $p = .016$). A significant main effect was found for blue saturation, $F(4,763) = 2.74$, $p = .03$, $\eta p^2 = .01$. Images in the general-threat categories had significantly less blue saturation than images in the neutral category (*Md* = 29.64, *SE* = 9.96, $p = .030$). A significant main effect was found for green saturation, $F(4, 763) = 4.19$, $p = .002$, $\eta p^2 = .02$. Images in the general-threat expressions category had significantly less green saturation than images in the neutral category (*Md* = 22.03, *SE* = 6.23, $p = .004$). A significant main effect was found for RMS contrast levels, $F(4,763) = 5.98$, $p < .001$, $\eta p^2 = .03$. Images in the facial expressions of pain category had significantly higher RMS contrast than the images included in musculoskeletal-pain (*Md* = 9.57, *SE* = 3.32, $p = .040$), health-threat (*Md* =13.77, *SE* = 3.32, $p < .001$) and neutral categories (*Md* = 10.89, *SE* = 2.49, $p < .001$).

For peer review only

Table 3

Mean Low-Level values per Image Category

	Musculoskeletal-pain		Facial Expressions of Pain		General-threat		Health-threat		All neutral stimuli	
	M	*SD*	*M*	*SD*	*M*	*SD*	*M*	*SD*	*M*	*SD*
Complexity	20.03	7.17	15.69	3.10	20.14	6.36	19.30	10.78	18.62	10.92
Red Saturation	148.72	41.35	172.95	22.79	148.99	43.43	164.37	57.07	167.76	45.41
Green Saturation	140.39	41.39	153.81	27.56	132.80	41.82	154.92	58.08	154.84	48.60
Blue Saturation	132.53	43.55	144.86	31.64	119.90	48.00	149.09	61.87	149.54	85.49
Luminance	142.01	41.10	158.57	25.50	136.21	41.39	157.37	57.10	162.79	92.44
RMS contrast level	61.25	15.3	70.82	18.75	64.22	18.95	57.00	20.91	59.93	18.86

Analyses and results: Valence, arousal and pain intensity. A repeated-measures ANOVA was conducted comparing arousal ratings (Table 4) across the five image categories, revealing a significant main effect, $F(2,23) = 32.79$, $p < .001$, $\eta p^2 = .69$. Pairwise comparisons revealed that the neutral category had significantly lower arousal scores than facial expressions of pain (*Md* = 2.24, *SE* = 0.45, *p* = .002), musculoskeletal-pain (*Md* = 1.28, *SE* = 0.33, *p* = .014), general-threat (*Md* = 2.86, *SE* = 0.38, *p* < .001), and health-threat (*Md* = 1.91, *SE* = 0.34, *p* = .001) categories. The general-threat category was significantly more arousing than facial expressions of pain (*Md* = 0.62, *SE* = 0.15, *p* = .010), musculoskeletal-pain (*Md* = 1.59, *SE* = 0.14, *p* < .001), and health-threat categories (*Md* = 0.95, SE =0.15, *p* < .001). The musculoskeletal-pain category was also significantly less arousing than the facial expressions of pain (*Md* = 0.96, *SE* = 0.15, *p* < .001) and the health-threat (*Md* = 0.64, *SE* = 0.14, *p* = .005) categories.

For peer review only - http://bmjopen.bmj.com/site/about/guidelines.xhtml

A repeated-measures ANOVA conduced on valence scores (Table 4) across the five image conditions revealed a significant main effect, $F(2,29) = 64.52$, $p < .001$, $\eta p^2 = .82$. Pairwise comparisons showed the neutral category had significantly higher pleasantness scores than facial expressions of pain (*Md* = 1.56, *SE* = 0.15, *p* <. 001), musculoskeletal-pain (*Md* = 0.86, *SE* = 0.13, *p* < .001), general-threat (*Md* = 1.69, *SE* = 0.16, *p*<. 001) and health-threat (*Md* = 1.27, *SE* = 0.18, *p*< .001) categories. Images in the musculoskeletal-pain category were rated significantly more pleasant than the images included in the facial expressions of pain (*Md* = 0.69, *SE* = 0.07, *p* < .001), general-threat (*Md* = 0.83, *SE* = 0.08, *p* < .001) and health-threat (*Md* = 0.41, *SE* = 0.09, *p*= .003) categories. Images in the health-threat category had significantly higher pleasantness scores than images included in the general-threat category (*Md* = 0.42, *SE* = 0.07, *p* < .001).

An independent samples *t*-test was conducted comparing pain intensity scores (Table 4) between the images in the musculoskeletal pain and facial expressions of pain categories. There was a significant difference in the pain intensity scores of musculoskeletal-pain (*M* = 3.35, *SD* = 2.27) and facial expressions of pain (*M* = 5.15, *SD* = 2.13) categories; $t(245) = 6.520$, $p < .001$, $r = 0.38$. This result suggests images included in the facial expressions of pain category had significantly higher pain intensity ratings than those included in the musculoskeletal-pain category.

For peer review only - http://bmjopen.bmj.com/site/about/guidelines.xhtml

Table 4

Mean Arousal, Valence and Pain Intensity Ratings per Image Category

	Arousal		Valence		Pain Intensity	
Image Category	*M*	*SD*	*M*	*SD*	*M*	*SD*
Musculoskeletal-pain	2.85	1.45	4.20	0.57	3.35	2.09
Facial expressions of pain	3.81	1.92	3.50	0.66	5.15	2.02
General-threat	4.43	1.69	3.37	0.62		
Health-threat	3.48	1.53	3.79	0.71		
Musculoskeletal-pain neutral counterparts	1.82	0.68	5.11	0.22		
Facial expressions of pain neutral counterparts	1.74	0.69	5.00	0.14		
General-threat neutral counterparts	1.82	0.68	5.08	0.17		
Health-threat neutral counterparts	1.75	0.54	5.04	0.24		
Neutral/Neutral	1.57	0.49	5.10	0.15		

For peer review only

Comparison between Visual-Probe Sessions – Linguistic Stimuli

Differences in Arousal Scores between Threatening Word Categories across the Nine Linguistic Visual Probe Sessions

A 4 (word category; sensory-pain, affective-pain, general-threat, health-threat) x 9 (linguistic visual probe session; session 1, 3, 5, 7, 9, 11, 13, 15, 17) ANOVA was conducted to compare the arousal scores of the threatening word categories across the 9 linguistic visual probe task sessions. The results showed a significant main effect of session, $F(8, 120) = 8.22$, $p < .001$, $\eta_p^2 = .35$, and a significant word category x session interaction, $F(24, 360) = 3.05$, $p < .001$, $\eta_p^2 = .17$.

Post-hoc analyses across sessions. To explore the significant interaction, a series of one-way ANOVAs with 9 levels (Session: 1, 3, 5, 7, 9, 11, 13, 15, 17) were run for each threatening word category independently. These explored whether the arousal ratings of each threatening word category differed across the sessions. For the sensory-pain words, a main effect of session was found, $F(5,67) = 6.02$, $p < .001$, $\eta_p^2 = .29$. Pairwise comparisons revealed sensory words to be significantly more arousing in session 1 than session 9 (*Md* = 0.92, *SE* = 0.22, *p* = .03), and significantly more arousing in session 17 than session 9 (*Md* = 0.75, *SE* = 0.17, *p* = .02) and session 15 (*Md* = 0.59, *SE* = 0.11, *p* = .003).

For the affective-pain words, a main effect of session was found, $F(8,120) = 4.26$, $p < .001$, $\eta_p^2 = .22$. Pairwise comparisons revealed these words to be significantly more arousing in session 1 than in session 5 (*Md* = 0.57, *SE* = 0.12, *p* = .01) and session 13 (*Md* = 1.04, *SE* = 0.26, *p* = .05).

For the general-threat words, a main effect of session was found, $F(8, 120) = 4.91$, $p < .001$, $\eta_p^2 = .25$. Pairwise comparisons revealed these words to be significantly more arousing in session 1 than session 5 (*Md* = 0.65, *SE* = 0.13, *p* = .01), session 7 (*Md* = 0.88, *SE* = 0.17, *p* = .004) and session 15 (*Md* = 0.84, *SE* = 0.19, *p* = .02).

For peer review only - http://bmjopen.bmj.com/site/about/guidelines.xhtml

For the health-threat words, a main effect of session was found, $F(8,120) = 4.08$, $p < .001$, $\eta_p^2 = .21$. Pairwise comparisons revealed these words to be significantly more arousing in session 1 than session 11 ($Md = 1.05$, $SE = 0.18$, $p = .001$).

Post-hoc analyses within sessions. To further explore the significant interaction, a series of one-way ANOVAs with 4 levels (word category; sensory-pain, affective-pain, general-threat and health-threat) were conducted for each session independently. These explored whether the arousal ratings of each threatening word category differed within each linguistic visual-probe session. No significant main effects of word category were found for session 1, $F(3,45) = 1.16$, $p =.20$, $\eta_p^2 = .10$, session 5, $F(3,45) = 2.34$, $p = .86$, $\eta_p^2 = .14$, session 13, $F(3,45) = 0.70$, $p = .06$, $\eta_p^2 = .05$, and session 17, $F(3,45) = 2.27$, $p = .09$, $\eta_p^2 = .13$.

A significant main effect of word category was found in session 3, $F(3,45) = 4.31$, $p = .01$, $\eta_p^2 = .22$. Pairwise comparisons revealed no significant differences between the word categories of this session, however.

A significant main effect of word category was found in session 7, $F(3,45) = 3.75$, $p = .02$, $\eta_p^2 = .20$. Pairwise comparisons revealed no significant differences between the four threat-related categories.

A significant main effect of word category was found in session 9, $F(3,45) = 5.22$, $p = .004$, $\eta_p^2 = .26$. Pairwise comparisons revealed that sensory-pain words were significantly less arousing than affective-pain ($Md = 0.84$, $SE = 0.21$, $p = .01$), general-threat ($Md = 0.77$, $SE = 0.23$, $p = .03$) and health-threat categories ($Md = 0.92$, $SE = 0.30$, $p = .05$).

A significant main effect of word category was found in session 11, $F(3,45) = 4.45$, $p = .01$, $\eta_p^2 = .23$. Pairwise comparisons showed that the general-threat words were significantly more arousing than the health-threat words ($Md = 0.61$, $SE = 0.19$, $p = .04$).

A significant main effect of word category was found in session 15, $F(3,45) = 2.86$, $p =.05$, $\eta_p^2 = .16$. Pairwise comparisons revealed no significant differences between the four threat-related categories.

For peer review only - http://bmjopen.bmj.com/site/about/guidelines.xhtml

Differences in Valence Scores between Threatening Word Categories across the nine Linguistic Visual Probe Sessions

A 4 (word category; sensory-pain, affective-pain, general-threat and health-threat) x 9 (linguistic visual probe sessions; 1, 3, 5, 7, 9, 11, 13, 15, and 17) ANOVA was conducted to compare the pleasantness scores of the threatening categories across the 9 linguistic visual probe task sessions. The results showed significant main effects of word category, $F(3,45) = 15.86$, $p < .001$, $\eta_p^2 = .51$, and session, $F(8,120) = 11.90$, $p < .001$, $\eta_p^2 = .44$, and a significant word category x session interaction, $F(24,360) = 2.57$, $p < .001$, $\eta_p^2 = .15$.

Post-hoc analyses across sessions. To explore the significant interaction, a series of one-way ANOVAS with 9 levels (Session; 1, 3, 5, 7, 9, 11, 13, 15, 17) were run for each word category independently. These explored whether the pleasantness ratings for each word category differed across the sessions. For the sensory-pain words, a main effect of session was found, $F(5,75) = 6.88$, $p< .001$, $\eta_p^2 = .31$. Pairwise comparisons revealed sensory words to be significantly less pleasant in session 1 than session 3 (*Md* = 0.49, *SE* = 0.12, *p* =.04), session 7 (*Md* = 0.59, *SE* = 0.11, *p* = .003) and session 9 (*Md* = 0.73, *SE* = 0.09, *p* < .001). Sensory-pain words were also significantly less pleasant in session 17 than session 5 (*Md* = 0.26, *SE* = 0.05, *p* = .01) and session 9 (*Md* = 0.49, *SE* = 0.09, *p* = .003).

For the affective-pain words, a main effect of session was found, $F(8,120) = 4.86$, $p< .001$, $\eta p^2 = .25$. Pairwise comparisons revealed these words to be significantly less pleasant in session 1 than session 5 (*Md* = 0.51, *SE* = 0.11, *p* = .01), session 7 (*Md* = 0.47, *SE* = 0.09, *p* = .01), session 13 (*Md* = 0.59, *SE* = 0.11, *p* = .004) and session 15 (*Md* = 0.44, *SE* = 0.11, *p* = .04).

For the general-threat words, a main effect of session was found, $F(8,120) = 9.64$, $p< .001$, $\eta p^2 = .39$. Pairwise comparisons revealed these words to be significantly less pleasant in session 1 than session 5 (*Md* = 0.63, *SE* = 0.09, *p* < .001), session 7 (*Md* = 0.65, *SE* = 0.11, *p* = .001), session 9 (*Md* = 0.44, *SE* = 0.07, *p* < .001), session 13 (*Md* = 0.33, *SE* = 0.06, *p* = .001) and session 15 (*Md* = 0.65, *SE* = 0.12, *p* = .002). Significantly less pleasant words were also found in

For peer review only - http://bmjopen.bmj.com/site/about/guidelines.xhtml

session 3 than session 5 (*Md* = 0.37, *SE* = 0.09, *p* = .03) and session 7 (*Md* = 0.39, *SE* = 0.08, *p* = .01). Session 17 included significantly less pleasant words than session 7 (*Md* = 0.41, *SE* = 0.10, *p* = .03).

For the health-threat words, a main effect of session was found, $F(4,58)= 2.90$, $p= .03$, $\eta_p^2 = .16$. Pairwise comparisons revealed these words to be significantly less pleasant in session 1 than session 11 (*Md* = 0.57, *SE* = 0.14, *p* = .03).

Post-hoc analyses within sessions. To further explore the significant interaction, one-way ANOVAs with 4 levels (word category; sensory-pain, affective-pain, general-threat and health-threat) were conducted for each session independently. These explored whether pleasantness ratings for each threatening word category differed within each session. For session 1, a significant main effect was found, $F(3,45) = 4.84$, $p = .01$, $\eta_p^2 = .24$. Pairwise comparisons revealed that sensory-pain words were significantly more pleasant than affective words (*Md* = 0.63, *SE* = 0.16, *p* = .01).

For session 3, a significant main effect was found, $F(3,45) = 12.71$, $p < .001$, $\eta_p^2 = .46$. Pairwise comparisons showed that sensory-pain words were significantly more pleasant than affective-pain (*Md* = 0.73, *SE* = .15, *p* < .001), general-threat (*Md* = 0.79, *SE* = 0.13, *p* < .001) and health-threat words (*Md* = 0.56, *SE* = 0.15, *p* = .01).

For session 5, a significant main effect was found, $F(3,45) = 6.45$, $p <.001$, $\eta_p^2 = .30$. Pairwise comparisons revealed that sensory-pain words were significantly more pleasant than the affective-pain words (*Md* = 0.62, *SE* = 0.15, *p* = .01).

For session 7, a significant main effect was found, $F(3,45) = 10.19$, $p < .001$, $\eta_p^2 = .41$. Pairwise comparisons revealed that sensory-pain words were significantly more pleasant than the affective-pain words (*Md* = 0.17, *SE* = 0.13, *p* < .001), the general-threat words (*Md* = 0.51, *SE* = 0.09, *p* < .001) and the health-threat words (*Md* = 0.55, *SE* = 0.16, *p* = .02).

For session 9, a significant main effect was found, $F(2,31) = 13.67$, $p < .001$, $\eta_p^2 = .48$. Pairwise comparisons revealed that the sensory-pain words were significantly more pleasant than

For peer review only - http://bmjopen.bmj.com/site/about/guidelines.xhtml

the affective-pain words (*Md* = 0.98, *SE*= 0.16, $p < .001$), the general-threat words (*Md* = 0.87, *SE* = 0.15, $p < .001$) and the health-threat words (*Md* = 0.92, *SE* = 0.21, $p < .001$).

For session 11, a significant main effect was found, $F(3,45) = 11.63$, $p < .001$, $\eta_p^2 = .44$. Pairwise comparisons revealed that the sensory-pain words were significantly more pleasant than the affective-pain (*Md* = 0.69, *SE* = 0.13, $p < .001$) and the general-threat words (*Md* = 0.70, *SE* = 0.17, $p < .001$). Also, the affective-pain words were significantly less pleasant than the health-threat words (*Md* = 9.39, *SE* = 0.13, $p = .04$).

For session 13, a significant main effect was found, $F(3,45) = 4.43$, $p = .01$, $\eta_p^2 = .23$. Pairwise comparisons revealed that the sensory-pain words were significantly more pleasant than the general-threat words (*Md* = 0.56, *SE* = 0.12, $p < .001$).

For session 15, a significant main effect was found, $F(3,45) = 8.40$, $p < .001$, $\eta_p^2 = .36$. Pairwise comparisons showed that the sensory-pain words were significantly more pleasant than the words included in the affective (*Md* = 0.61, *SE* = 0.12, $p < .001$) and the health-threat words (*Md* = 0.71, *SE* = 0.19, $p = .01$).

For session 17, a significant main effect was found, $F(3,45) = 6.78$, $p<.001$, $\eta_p^2 = 0.36$. ${}_p^2$ = 0.31. Pairwise comparisons revealed that the sensory-pain words were significantly more pleasant than affective-pain (*Md* = 0.56, *SE* = 0.15, $p = .01$) and the general-threat words (*Md* = 0.57, *SE* = 0.15, $p = .01$).

Comparison between Visual-Probe Sessions – Pictorial Stimuli

Differences in Arousal Scores between Threatening Image Categories across the eight Pictorial Visual Probe Sessions

A 4 (image category; musculoskeletal-pain, facial expressions of pain, general-threat, health-threat) x 8 (pictorial visual probe sessions; 2, 4, 6, 8, 10, 12, 14, 16) ANOVA was conducted to compare the arousal scores of the threatening image categories across the 8 pictorial visual probe task sessions. The results showed a significant main effect of image category, $F(3,45) = 33.52$, *p*

For peer review only - http://bmjopen.bmj.com/site/about/guidelines.xhtml

< .001, η_p^2 = .69, and a significant image category x session interaction, $F(7,98) = 9.84$, $p < .001$, η_p^2 = .40.

Post-hoc analyses within image categories. To explore the significant interaction, a series of one-way ANOVAs with 8 levels (Session; 2, 4, 6, 8, 10, 12, 14, 16) were run for each threatening image category independently. These explored whether the arousal ratings of each threat-related image category differed across the sessions. For the images in the musculoskeletal-pain category, no main effect of session was found, $F(2,31) = 2.49$, $p = .10$, $\eta_p^2 = .14$. For the images in the facial expressions of pain category, no main effect of session was found, $F(3,46) = 0.77$, $p = .50$, $\eta_p^2 = .05$.

For the images in the general-threat category, a main effect of session was found, $F(7, 105) = 7.40$, $p < .001$, $\eta_p^2 = .33$. Pairwise comparisons revealed these images to be significantly less arousing in session 4 than session 6 (*Md* = 0.70, *SE* = 0.17, *p* = .023), session 12 (*Md* = 0.73, *SE* = 0.19, *p* = .04) and session 16 (*Md* = 0.82, *SE* = 0.21, *p* = .04). These images were also found to be significantly less arousing in session 14 than session 6 (*Md* = 0.93, *SE* = 0.19, *p* = .006), session 12 (*Md* = 0.96, *SE* = 0.17, *p* = .001) and session 16 (*Md* = 1,05, *SE* = 0.17, *p* = .001).

For the images in the health-threat category, a main effect of session was found, $F(7, 105) = 13.10$, $p < .001$, $\eta_p^2 = .47$. Pairwise comparisons revealed these images to be significantly more arousing in session 10 than session 8 (*Md* = 1.15, *SE* = 0.20, *p* = .001) and session 12 (*Md* = 0.88, *SE* = 0.20, *p* = .02). Health-threat images were also found to be significantly less arousing in session 16 than session 2 (*Md* = 0.97, *SE* = 0.17, *p* = .001), session 4 (*Md* = 1.34, *SE* = 0.26, *p* = .004), session 6 (*Md* = 1.32, *SE* = 0.29, *p* = .009), session 10 (*Md* = 1.91, *SE* = 0.28, *p*< .001), session 12 (*Md* = 1.03, *SE* = 0.26, *p* = .04) and session 14 (*Md* = 1.45, *SE* = 0.9, *p*< .001).

Post-hoc analyses within sessions. To further explore the significant interaction, a series of one-way ANOVAs with 4 levels (image category; musculoskeletal-pain, facial expressions of pain, general-threat and health-threat) were conducted for each session independently. These

For peer review only - http://bmjopen.bmj.com/site/about/guidelines.xhtml

explored whether the arousal ratings of each threatening image category differed within each pictorial visual-probe session.

A significant main effect of image category was found in session 2, $F(3,45) = 23.27$, $p < .001$, $\eta_p^2 = .61$. Pairwise comparisons revealed that the images in the facial expressions of pain category were more arousing than those included in the musculoskeletal-pain category (Md = 0.98, SE = 0.27, $p = .014$). The images in the general-threat category were also found to be significantly more arousing than those included in the musculoskeletal-pain (Md = 2.05, SE = 0.32, $p < .001$), facial expressions of pain (Md = 1.07, SE = 0.19, $p < .001$) and health-threat categories (Md = 1.34, SE = 0.24, $p < .001$).

A significant main effect of image category was found in session 4, $F(3,45) = 8.27$, $p < .001$, $\eta_p^2 = .36$. Pairwise comparisons revealed that the images in the musculoskeletal-pain category were significantly less arousing than the images in the facial expressions of pain (Md = 0.76, SE = 0.16, $p = .001$), health-threat (Md = 1.10, SE = 0.23, $p = .002$) and general-threat categories (Md = 0.70, SE = 0.22, $p = .04$).

A significant main effect of image category was found in session 6, $F(3,45) = 26.77$, $p < .001$, $\eta_p^2 = .64$. Pairwise comparisons revealed that the images in the musculoskeletal-pain category were significantly less arousing than the images included in the facial expressions of pain (Md = 1.17, SE = 0.25, $p = .002$), general-threat (Md = 2.06, SE = 0.23, $p < .001$) and health-threat categories (Md = 0.97, SE = 0.25, $p = .009$). The images in the general-threat category were significantly more arousing than those in the facial expressions of pain (Md = 0.89, SE = 0.21, $p = .003$) and health-threat categories (Md = 1.09, SE = 0.21, $p = .001$).

A significant main effect of image category was also found in session 8, $F(2,32) = 10.56$, $p < .001$, $\eta_p^2 = .41$. Pairwise comparisons revealed that the images in the musculoskeletal-pain category were significantly less arousing than those included in the facial expressions of pain (Md = 0.89, SE = 0.22, $p = .006$) and general-threat categories (Md = 1.13, SE = 0.25, $p = .003$).

For peer review only - http://bmjopen.bmj.com/site/about/guidelines.xhtml

The images in general-threat categories were significantly more arousing that those included in the health-threat category (*Md* = 1.00, *SE* = 0.20, p = .001).

A significant main effect of image category was found in session 10, $F(3,45) = 17.37$, $p < .001$, $\eta_p^2 = .54$. Pairwise comparisons revealed that the images in the musculoskeletal-pain category were significantly less arousing than those included in the general-threat (*Md* = 0.92, *SE* = 0.17, $p < .001$) and health-threat categories (*Md* = 1.23, *SE* = 0.20, $p < .001$). The images in the facial expressions of pain category were also significantly less arousing than those included in the health-threat category (*Md* = 0.71, *SE* = 0.18, $p = .009$).

A significant main effect of image category was found in session 12, $F(3,45) = 18.40$, $p < .001$, $\eta_p^2 = .55$. Pairwise comparisons revealed that the images in the musculoskeletal-pain category were significantly less arousing that those included in the facial expressions of pain (*Md* = 1.61, *SE* = 0.18, $p = .022$) and general-threat categories (*Md* = 1.64, *SE* = 0.19, $p < .001$). The images included in the general-threat category were significantly more arousing than the images included in the facial expressions of pain (*Md* = 1.03, *SE* = 0.24, $p = .001$) and health-threat categories (*Md* = 1.03, *SE* = 0.26, $p < .001$).

A significant main effect of image category was found in session 14, $F(3,45) = 14.72$, $p < .001$, $\eta_p^2 = .50$. Pairwise comparisons revealed than the images in the musculoskeletal-pain category were significantly less arousing than those included in facial expressions of pain (*Md* = 1.39, *SE* = 0.30, $p = .002$), general-threat (*Md* = 1.41, *SE* = 0.25, $p < .001$) and health-threat categories (*Md* = 1.35, *SE* = 0.20, $p < .001$).

A significant main effect of image category was found in session 16, $F(2,28) = 38.74$, $p < .001$, $\eta_p^2 = .72$. Pairwise comparisons revealed that the images in the musculoskeletal-pain category were significantly less arousing than the images included in the facial expressions of pain (*Md* = 1.38, *SE* = 0.29, $p = .001$) and general-threat categories (*Md* = 2.37, *SE* = 0.27, $p = .002$). Images included in the facial expression of pain category were also found to be significantly less arousing than those included in the general-threat category (*Md* = 0.98, *SE* =

For peer review only - http://bmjopen.bmj.com/site/about/guidelines.xhtml

0.21, p = .002). Images in the health-threat category were significantly less arousing than the images included in the facial expression of pain (Md = 1.57, SE = 0.36, p = .004) and general-threat categories (Md = 2.56, SE = 0.29, p < .001).

Differences in Valence Scores between Threatening Pictorial Categories across the eight Pictorial Visual Probe Sessions

A 4 (image category; musculoskeletal-pain, facial expressions of pain, general-threat and health-threat) x 8 (pictorial visual probe sessions; 2, 4, 6, 8, 10, 12, 14, 16) ANOVA was conducted to compare the pleasantness scores of the threatening categories across the 8 pictorial visual probe task sessions. The results showed a significant main effect of image category, $F(3,45) = 41.09$, $p < .001$, $\eta_p^2 = .73$, and a significant image category x session interaction, $F(7,102) = 9.33$, $p < .001$, $\eta_p^2 = .38$.

Post-hoc analyses within image categories. To explore the significant interaction, a series of one-way ANOVAS with 8 levels (Session; 2, 4, 6, 8, 10, 12, 14, 16) were run for each image category independently. These explored whether the pleasantness ratings for each image category differed across the sessions.

For the images included in the musculoskeletal-pain category, a main effect of session was found, $F(2,32) = 3.70$, $p = .033$, $\eta_p^2 = .20$. Pairwise comparisons revealed these images to be significantly more pleasant in session 2 than session 4 (Md = 0.54, SE = 0.11, p = .007).

For the images included in the facial expressions of pain category, a main effect of session was found, $F(7,105) = 3.44$, $p = .002$, $\eta_p^2 = .19$. Pairwise comparisons revealed these images to be significantly more pleasant in session 10 than session 6 (Md = 0.51, SE = 0.13, p = .03) and session 16 (Md = 0.43, SE = 0.09, p = .005).

For the images included in the general-threat category, a main effect of session was found, $F(7,105) = 6.84$, $p < .001$, $\eta_p^2 = .31$. Pairwise comparisons revealed these images to be significantly more pleasant in session 10 than session 6 (Md = 0.61, SE = 0.13, p = .009), session 12 (Md = 0.56, SE = 0.14, p = .05) and session 16 (Md = 0.68, SE = 0.14, p = .007). General-

For peer review only - http://bmjopen.bmj.com/site/about/guidelines.xhtml

threat images were significantly more pleasant in session 14 than session 6 (*Md* = 0.60, *SE* = 0.14, p = .019), session 12 (*Md* = 0.55, *SE* = 0.12, p = .013) and session 16 (*Md* = 0.67, *SE* = 0.11, p = .001). Images in this category were also significantly more pleasant in session 4 than session 6 (*Md* = 0.45, *SE* = 0.11, p = .04) and session 16 (*Md* = 0.52, *SE* = 0.11, p = .007).

For the images included in the health-threat category, a main effect of session was found, $F(7,105) = 9.87$, $p < .001$, $\eta_p^2 = .40$. Pairwise comparisons revealed these images to be significantly more pleasant in session 8 than session 10 (*Md* = 0.73, *SE* = 0.10, p = .005). Images in this category were significantly more pleasant in session 16 than session 2 (*Md* = 0.54, *SE* = 0.11, p = .004), session 4 (*Md* = 0.62, *SE* = 0.12, p = .003), session 6 (*Md* = 0.52, *SE* = 0.12, p = .02), session 10 (*Md* = 1.00, *SE* = 0.10, $p < .001$), session 12 (*Md* = 0.56, *SE* = 0.12, p = .008) and session 14 (*Md* = 0.80, *SE* = 0.10, $p < .001$).

Post-hoc analyses within sessions. To further explore the significant interaction, one-way ANOVAs with 4 levels (image category; musculoskeletal-pain, facial expressions of pain, general-threat and health-threat) were conducted for each session independently. These explored whether pleasantness ratings for each image category differed within each session.

For session 2, a significant main effect was found, $F(3,45) = 13.36$, $p < .001$, $\eta_p^2 = .47$. Pairwise comparisons revealed that images in the musculoskeletal-pain category were significantly more pleasant than images included in the facial expressions of pain (*Md* = 1.00, *SE* = 0.18, $p < .001$), general-threat (*Md* = 0.98, *SE* = 0.20, p = .001) and health-threat categories (*Md* = 0.72, *SE* = 0.21, p = .024).

For session 4, a significant main effect was found, $F(3,45) = 7.09$, $p = .001$, $\eta_p^2 = .32$. Pairwise comparisons showed that the images in the musculoskeletal-pain category were significantly more pleasant than the facial expressions of pain (*Md* = 0.52, *SE* = 0.10, p = .001) and general-threat categories (*Md* = 0.48, *SE* = 0.12, p = .005).

For session 6, a significant main effect was found, $F(3,45) = 32.12$, $p <.001$, $\eta_p^2 = .68$. Pairwise comparisons revealed that images in the musculoskeletal-pain category were

For peer review only - http://bmjopen.bmj.com/site/about/guidelines.xhtml

significantly more pleasant than the images included in the facial expressions of pain (*Md* = 1.05, *SE* = 0.14, $p < .001$) and general-threat categories (*Md* = 1.27, *SE* = 0.13, $p < .001$). The images in the health-threat category were also significantly more pleasant than the images included in the facial expressions of pain (*Md* = 0.56, *SE* = 0.14, $p = .01$) and general-threat categories (*Md* = 0.77, *SE* = 0.13, $p < .001$).

For session 8, a significant main effect was found, $F(3,45) = 11.73$, $p < .001$, $\eta_p^2 = .44$. Pairwise comparisons revealed that images in the musculoskeletal-pain category were significantly more pleasant than those included in the facial expressions of pain (*Md* = 0.62, *SE* = 0.07, $p < .001$) and general-threat categories (*Md* = 0.57, *SE* = 0.14, $p = .004$). The images included in the health-threat category were also significantly more pleasant than those included in the facial expressions of pain (*Md* = 0.54, *SE* = 0.15, $p = .019$) and general-threat categories (*Md* = 0.49, *SE* = 0.14, $p = .016$).

For session 10, a significant main effect was found, $F(3,45) = 12.59$, $p < .001$, $\eta_p^2 = .46$. Pairwise comparisons revealed that images in the health-threat category were significantly less pleasant than those included in the musculoskeletal-pain (*Md* = 0.74, *SE* = 0.12, $p < .001$) and facial expressions of pain categories (*Md* = 0.44, *SE* = 0.12, $p = .017$).

For session 12, a significant main effect was found, $F(3,45) = 14.90$, $p < .001$, $\eta_p^2 = .50$. Pairwise comparisons revealed that images in the musculoskeletal-pain category were significantly more pleasant than images included in the facial expressions of pain (*Md* = 0.45, *SE* = 0.08, $p < .001$) and general-threat categories (*Md* = 0.88, *SE* = 0.16, $p < .001$). Images in the health-threat category were also significantly more pleasant than images included in the general health category (*Md* = 0.68, *SE* = 0.16, $p = .004$).

For session 14, a significant main effect was found, $F(3,45) = 13.30$, $p < .001$, $\eta_p^2 = .47$. Pairwise comparisons revealed that images in the musculoskeletal-pain category were significantly more pleasant than images included in the facial expressions of pain (*Md* = 0.71, *SE*

For peer review only - http://bmjopen.bmj.com/site/about/guidelines.xhtml

= 0.16, *p* = .003), general-threat (*Md* = 0.71, *SE* = 0.15, *p* = .001) and health-threat categories (*Md* = 0.82, *SE* = 0.12, *p* < .001).

For session 16, a significant main effect was found, $F(3,45) = 83.05$, $p < .001$, $\eta_p^2 = .85$. Pairwise comparisons revealed that images in the facial expressions of pain category were significantly less pleasant than musculoskeletal-pain (*Md* = 0.97, *SE* = 0.10, *p* < .001) and health-threat categories (*Md* = 0.99, *SE* = 0.12, *p* < .001). Images in the general-threat category were also significantly less pleasant than those included in the musculoskeletal-pain (*Md* = 1.34, *SE* = 0.11, *p* < .001), facial expressions of pain (*Md* = 0.37, *SE* = 0.09, *p* = .007) and health-threat categories (*Md* = 1.36, *SE* = 0.09, *p* < .001).

Differences in Pain Intensity Scores of Threatening Pain Categories across the eight Pictorial Visual Probe Sessions

A 2 (image category; musculoskeletal-pain, facial expressions of pain) x 8 (pictorial visual probe sessions; session 2, 4, 6, 8, 10, 12, 14, 16) ANOVA was conducted to compare the pain intensity scores of the threatening pain categories across the 8 pictorial visual probe task sessions. The results showed significant main effects of image category, $F(1,15) = 45.40$, $p < .001$, $\eta_p^2 = .75$, and session, $F(2,29) = 3.81$, $p = .04$, $\eta_p^2 = .20$, and a significant image category x session interaction, $F(3,44) = 7.44$, $p < .001$, $\eta_p^2 = .33$.

Post-hoc analyses within image categories. To explore the significant interaction, two one-way ANOVAs with 8 levels (Session; 2, 4, 6, 8, 10, 12, 14, 16) were run for each pain-related image category independently. These explored whether the pain intensity ratings of each pain-related image category differed across the sessions. For the musculoskeletal-pain category, a main effect of session was found, $F(2,25) = 4.70$, $p = .023$, $\eta p^2 = .24$. Pairwise comparisons revealed that the images included in session 14 had significantly less pain intensity scores than the images included in session 8 (*Md* = 1.06, *SE* = 0.24, *p* = .014), session 10 (*Md* = 0.95, *SE* = 0.19, *p* = .004) and session 12 (*Md* = 1.19, *SE* = 0.15, *p* < .001). Images in session 4 had significantly higher pain intensity scores than the images included in session 6 (*Md* = 0.82, *SE* =

For peer review only - http://bmjopen.bmj.com/site/about/guidelines.xhtml

0.21, p = .05). The images included in session 12 had also significantly higher pain intensity scores than the images included in session 16 (*Md* = 1.03, *SE* = 0.17, p = .001).

For the facial expression of pain category, a main effect of session was found, $F(3,39)$ = 4.65, p = .01, ηp^2 = .24. Pairwise comparisons revealed that the images included in session 10 had significantly less pain intensity scores that the images included in session 1 (*Md* = 1.07, *SE* = 0.25, p = .02) and session 6 (*Md* = 1.38, *SE* = 0.31, p = .013).

Differences in low-level features of pictorial categories across the eight visual probe sessions

A series of one-way ANOVAs were conducted independently to compare the scores of complexity, red, green and blue saturation, luminance and RMS contrast level of the threat related categories (i.e., musculoskeletal-pain, facial expression of pain, general-threat and health-threat) across the 8 pictorial visual probe task sessions (i.e., Session 2, 4, 6, 8, 10, 12, 14, 16).

For complexity, the analysis revealed a significant main effect of image category, $F(3, 224)$ = 5.15, p = .002, η_p^2 = .06. There was no significant main effect of session, $F(7, 224)$ = 1.22, p = .30, and no significant image category x session interaction, $F(21, 224)$ = 0.82, p =.70.

For red saturation, the analysis revealed a significant main effect of image category, $F(3, 224)$ = 5.05, p = .002, η_p^2 = .06. There was no significant main effect of session , $F(7,224)$ = 1.03, p = .41, and no significant image category x session interaction, $F(21,224)$ = 1.196, p =.26.

For green saturation, the analysis revealed a significant main effect of image category, $F(3, 224)$ = 4.08, p = .008, η_p^2 = .08. There was no significant main effect of session, $F(7, 224)$ = 1.65, p = .12, and no and no significant image category x session interaction, $F(21,224)$ = 1.41, p =.12.

For blue saturation, the analysis revealed a significant main effect of image category, $F(3, 224)$ = 5.288, p = .002, η_p^2 = 0.07, and a significant image category x session interaction, $F(21, 224)$ = 1.672, p = .04, η_p^2 = 0.14. There was no significant main effect of session, $F(7, 224)$ = 1.750, p = .10.

For peer review only - http://bmjopen.bmj.com/site/about/guidelines.xhtml

For luminance, the analysis revealed a significant main effect of image category, $F(3, 224) = 4.548$, $p = .004$, $\eta_p^2 = 0.06$. The analysis revealed no significant main effect of session, $F(7,224) = 1.504$, $p = .17$, and no significant image category x session interaction, $F(21, 224) = 1.361$, $p = .14$.

For RMS contrast level, the analysis revealed a significant main effect of image category, $F(3, 224) = 6.84$, $p < .001$, $\eta_p^2 = .08$, and a significant image category x session interaction, $F(21,224) = 1.98$, $p = .008$. There was no significant main effect of session, $F(7,224) = 1.68$, $p = .12$,

Post-hoc analyses within image categories. To explore the significant interaction for blue saturation, a series of one-way ANOVAS with 8 levels (Session; 2, 4, 6, 8, 10, 12, 14, 16) were run for each image category independently. These explored whether the blue saturation scores for each image category differed across the sessions. No significant main effect of session was found for the musculoskeletal-pain category, $F(7,56) = 0.82$, $p = .57$, or the health-threat category, $F(7,56) = 1.59$, $p = .16$.

For the facial expressions of pain category, a significant interaction for session number was found, $F(7,56) = 2.94$, $p = .01$, $\eta_p^2 = .27$. Pairwise comparisons did not reveal any significant differences between the factors.

For the general-threat category, a significant main effect of session number, $F(7,56) = 2.20$, $p = .05$, $\eta_p^2 = .22$. Pairwise comparisons did not reveal any significant differences between the factors.

To explore the significant interaction for RMS contrast level, a series of one-way ANOVAS with 8 levels (Session; 2, 4, 6, 8, 10, 12, 14, 16) were run for each image category independently. These explored whether the RMS contrast level scores for each image category differed across the sessions. No significant main effect of session was found for the musculoskeletal-pain category, $F(7,56) = 0.59$, $p = .76$, or the health-threat category, $F(7,56) = 0.16$, $p = .99$

For peer review only - http://bmjopen.bmj.com/site/about/guidelines.xhtml

For the facial expressions of pain category, a significant interaction for session number was found, $F(7,56) = 5.127$, $p < .001$, $\eta_p^2 = 0.39$. Pairwise comparisons revealed that the images in session 6 had significantly less RMS contrast than those included in session 4 (*Md* = 36.02, *SE* = 7.76, *p* = .001), session 10 (*Md* = 30.81, *SE* = 7.76, *p* = .006), session 14 (*Md* = 25.96, *SE* = 7.76, *p* = .04) and session 16 (*Md* = 37.09, *SE* = 776, *p*< .001).

For the general-threat category, a significant interaction for session number was found, $F(7,56) = 3.252$, $p = .006$, $\eta_p^2 = 0.29$. Pairwise comparisons did not reveal any significant interaction between the sessions.

Post-hoc analyses within sessions. To further explore the significant interaction for blue saturation, eight one-way ANOVAs with 4 levels (image category; musculoskeletal-pain, facial expressions of pain, general-threat and health-threat) were conducted for each session independently. These explored whether blue saturation scores between the five image categories differed within each session (i.e., session 2, 4, 6, 8, 10, 12, 14, 16).

No significant main effects were found for session 2, $F(3,28) = 1.49$, $p = .24$, session 4, $F(3,28) = 0.18$, $p = .91$, session 6, $F(3,28) = 1.37$, $p = .27$, session 8, $F(3,28) = 1.127$, $p = .36$, and session 14, $F(3,28) = 0.508$, $p = .68$.

For session 10, there was a significant main effect of image category, $F(3,28) = 5.56$, $p = .004$, $\eta_p^2 = .37$. Pairwise comparisons revealed that the images included in the facial expression of pain category had significantly higher blue saturation than the images included in the general-threat category (*Md* = 70.64, *SE* = 18.03, *p* = .003).

For session 12, there was a significant main effect of image category, $F(3,28) = 3.55$, $p = .027$, $\eta_p^2 = .28$. Pairwise comparisons revealed that the images included in the musculoskeletal-pain category had significantly higher blue saturation than the images included in the general-threat category (*Md* = 62.46, *SE* = 21.76, *p* = .046).

For session 16, there was a significant main effect of image category, $F(3,28) = 6.99$, $p = .001$, $\eta_p^2 = .43$. Pairwise comparisons revealed that the images included in the health-threat

For peer review only - http://bmjopen.bmj.com/site/about/guidelines.xhtml

categories had significantly higher blue saturation than the images included in the musculoskeletal-pain (*Md* = 74.57, *SE* = 20.09, *p* = .005) and general-threat categories (*Md* = 83.89, *SE* = 20.09, *p* = .002)

To further explore the significant interaction for RMS contrast level, eight one-way ANOVAs with 4 levels (image category; musculoskeletal-pain, facial expressions of pain, general-threat and health-threat) were conducted for each session independently. These explored whether the scores of RMS contrast level between the five image categories differed within each session (i.e., session 2, 4, 6, 8, 10, 12, 14, 16).

No significant main effects were found for session 2, $F(3,28) = 0.720$, $p = .55$, session 8, $F(3,28) = 1.56$, $p = .22$, session 12, $F(3,28) = 0.62$, $p = .61$, and session 14, $F(3,28) = 1.76$, $p = .18$.

For session 4, there was a significant main effect of image category, $F(3,28) = 5.69$, $p = .004$, $\eta_p^2 = .38$. Pairwise comparisons revealed that the images included in the facial expression of pain category had significantly higher RMS contrast than the images included in the health-threat category (*Md* = 33.44, *SE* = 8.27, *p* = .002).

For session 6, there was a significant main effect of image category, $F(3,28) = 4.00$, $p = .02$, $\eta_p^2 = .30$. Pairwise comparisons revealed that the images included in the musculoskeletal-pain category had significantly higher RMS contrast than those included in the facial expressions of pain category (*Md* = 17.69, *SE* = 5.95, *p* = .04).

For session 10, there was a significant main effect of image category, $F(3,28) = 6.02$, $p = .003$, $\eta_p^2 = .39$. Pairwise comparisons revealed that the images included in the facial expressions of pain category had significantly higher RMS contrast than those included in the general-threat (*Md* = 30.37, *SE* = 7.35, *p* = .002) and health-threat categories (*Md* = 21.42, *SE* = 7.35, *p* = .04).

For session 16, there was a significant main effect of image category, $F(3,28) = 3.18$, $p = .04$, $\eta_p^2 = .25$. Pairwise comparisons revealed that the images included in the facial expressions

For peer review only - http://bmjopen.bmj.com/site/about/guidelines.xhtml

of pain category had significantly higher RMS contrast level than those included in the health-threat category (*Md* = 29.32, *SE* = 10.24, *p* = .05).

For peer review only

References

1. Kučera H, Francis N. *Computational analysis of present-day American English*. Providence: Brown University Press, 1967.

2. AP Y. The Nature of Recollection and Familiarity: A Review of 30 Years of Research. Journal of Memory and Language 2002;**46**(3):441-517 doi: 10.1006/jmla.2002.2864.

3. Balota DA, Chumbley JI. Are lexical decisions a good measure of lexical access? The role of word frequency in the neglected decision stage. Journal of Experimental Psychology: Human perception and performance 1984;**10**(3):340-57.

4. Sharpe L, Ianiello, M., Dear, B. F, Nicholson Perry, K., Refshauge, K., Nicholas, M. K. Is there a potential role for attention bias modification in pain patients? Results of 2 randomised, controlled trials. Pain 2012;**153**(3):722-31.

5. Schoth D, Liossi C. Specificity and time-course of attentional bias in chronic headache: a visual-probe investigation. Clinical Journal of Pain 2013;**29**(7):583-90 doi: 10.1097/AJP.0b013e31826b4849.

6. Carleton R, Richter A, Asmundson G. Attention Modification in Persons with Fibromyalgia: A Double Blind, Randomized Clinical Trial. Cognitive Behaviour Therapy 2011;**40**(4):279-90.

7. Macleod C. Cognitive bias modification procedures in the management of mental disorders. Curr Opin Psychiatry 2012;**25**(2):114-20 doi: 10.1097/YCO.0b013e32834fda4a.

8. MacLeod C, Mathews A, Tata P. Attentional bias in emotional disorders. Journal of Abnormal Psychology 1986;**95**(1):15-20 doi: 10.1037/0021-843X.95.1.15.

9. Melzack R. The McGill Pain Questionnaire: major properties and scoring methods. Pain 1975;**1**(3):277-99.

10. Wilson MD. The MRC Psycholinguistic Database: Machine Readable Dictionary, Version 2. Behavioural Research Methods, Instruments and Computers 1988;**20**(1):6-11.

For peer review only - http://bmjopen.bmj.com/site/about/guidelines.xhtml

11. MRC Psycholinguistic Database: Machine Usable Dictionary. Version 2.00.: MRC Psycholinguistic Database, 1997.

12. Lane RD, Chua PM, Dolan RJ. Common effects of emotional valence, arousal and attention on neural activation during visual processing of pictures. Neuropsychologia 1999;**37**(9):989-97 doi: 10.1016/S0028-3932(99)00017-2.

13. Niedenthal PM, Kitayama S. *The heart's eye: Emotional influences in perception and attention*. London: Academic Press, 2013.

14. Bradley MM, Lang PJ. Measuring Emotion: The Self-Assessment Manikin and the Semantic Differential. J Behav Therap & Exp Psychiat 1994;**25**(1):49-59.

15. Liossi C, Schoth D. Attention toward interpersonal stimuli in individuals with and without chronic daily headache. Journal of Neurology and Neurosurgery 2016;**3**(3):1-7.

16. Schoth DE, Liossi C. Attentional bias toward pictorial representations of pain in individuals with chronic headache. Clin J Pain 2010;**26**(3):244-50 doi: 10.1097/AJP.0b013e3181bed0f9.

17. Craig KD. The facial expression of pain. Better than a thousand words? APS Journal 1992;**1**(3):153-62.

18. Prkachin KM, Solomon PE. The structure, reliability and validity of pain expression: evidence from patients with shoulder pain. Pain 2008;**139**(2):267-74 doi: 10.1016/j.pain.2008.04.010.

19. Schoth DE, Georgallis T, Liossi C. Attentional bias modification in people with chronic pain: a proof of concept study. Cognitive Behaviour Therapy 2013;**42**(3):233-43.

20. Bradley MM, Lang PJ. The International Affective Picture System (IAPS) in the study of emotion and attention. In: Allen CaJJB, ed. Handbook of Emotion Elicitation and Assessment: Oxford University Press, 2007.

21. Leeuw M, Goossens ME, van Breukelen GJ, et al. Measuring perceived harmfulness of physical activities in patients with chronic low back pain: the Photograph Series of Daily

For peer review only - http://bmjopen.bmj.com/site/about/guidelines.xhtml

Activities--short electronic version. J Pain 2007;**8**(11):840-9 doi: 10.1016/j.jpain.2007.05.013.

22. Tottenham N, Tanaka JW, Leon AC, et al. The NimStim set of facial expressions: judgments from untrained research participants. Psychiatry Res 2009;**168**(3):242-9 doi: 10.1016/j.psychres.2008.05.006.

23. Nummenmaa L, Hyönä J, Calvo MG, & Nummenmaa, L. Eye movement assessment of selective attentional capture by emotional pictures. Emotion 2006;**6**(2):257-68 doi: 10.1037/1528-3542.6.2.257.

24. Borji A, Sihite DN, Itti L. What stands out in a scene? A study of human explicit saliency judgment. Vision research 2013;**91**:62-77 doi: 10.1016/j.visres.2013.07.016.

25. Henderson JM. Human gaze control during real-world scene perception. Trends in Cognitive Sciences 2003;**7**(11):498-504 doi: 0.1016/j.tics.2003.09.006.

26. Torralba A, Oliva, A., Castelhano, M. S., & Henderson, J. M. Contextual guidance of eye movements and attention in real-world scenes: The role of global features in object search. Psychological Review 2006;**113**(4):766-86 doi: 10.1037/0033-295X.113.4.766.

27. Buodo G, Sarlo M, Palomba D. Attentional resources measured by reaction times highlight differences within pleasant and unpleasant, high arousing stimuli. Motivation and Emotion 2002;**26**(2):123-38 doi: 10.1023/A:1019886501965.

For peer review only

Supplementary Material 2 – Outcome Measures

A detailed description of each outcome measure used in this study is provided here. While the questionnaires will be administered online as part of this study, the psychometric properties reported are for paper and pencil versions of the questionnaires.

The BPI-SF[1] is a commonly used measure of pain intensity and pain interference. Participants indicate their worst, least, average and current pain on an 11-point numerical scale ranging from *no pain at all* to *pain as bad as you can imagine*. Additional questions include how much relief pain medication has provided, along with pain interference on general activity, mood, walking ability, normal work, relations with others, sleep, and enjoyment of life. A body diagram is also provided for participants to indicate where they feel pain. The psychometric properties of the BPI are well supported in patients with cancer pain and other pain populations[1] [2], including internal consistency of pain intensity and interference items as assessed in patients with chronic nonmalignant pain (Cronbach's alpha .85 and .88 respectively).[3]

The HADS[4] consists of 14 items measuring the severity of anxiety and depression symptoms (seven items each) over the past week. Each item is measured on a four point scale, with possible scores ranging from 0 – 21 for each subscale. Higher scores indicate higher levels of anxiety and depression. A review of studies using the HADS (the majority of which were conducted with patients with cancer or other physical health conditions) has supported the internal consistency of anxiety (.68 - .93, mean .82) and depression (.67 - .90, mean .82) subscales.[5]

The STAI[6] is a 40-item measure of state and trait anxiety (20 items each). Each item is measuerd on a four point scale , with possible scores for both state and trait levels ranging between 20 and 80. Higher scores represent more intense or more frequent feelings of anxiety. A review of 45 articles reporting psychometric properties from clinical and non-clinical samples of state and trait subscales found high levels of internal consistency (.91 and .89 respectively) and test-retest reliability (.70 and .88 respectively).[7]

For peer review only - http://bmjopen.bmj.com/site/about/guidelines.xhtml

The FOP-III[8] is a 30 item measure of pain-related fear. On a 5-point scale, respondents are asked to rate how fearful they are of pain associated with specific situations and events (e.g., being in an automobile accident). Possible scores range between 30 and 150, with higher scores representing a more intense fear of pain. In addition to a total score, the measure also provides subscales of minor, severe, and medical pain-related fear. The FOP-III is commonly used in clinical and non-clinical populations, and possesses high internal consistency as indexed in a non-clinical sample (total score = .93; severe = .88; minor = .86, medical = .88).[9]

The MOS-SS[10] is a twelve-item questionnaire developed for patients with chronic illness, measuring six dimensions of sleep over the past four weeks; sleep initiation (time to fall asleep), quantity (hours per night sleeping), maintenance, respiratory problems, perceived adequacy, and somnolence. A sleep problems index which summarises information from nine items may also be scored. Responses are recoded on a 0 – 100 scale, with higher scores indicating more of the concept being measured (e.g., greater difficulties falling asleep). Research has supported the internal consistency of the MOS-SS scales to be acceptable (.70) or higher in samples of patients with neuropathic pain.[11 12]

The SOT[13] assesses participants' attitudes towards their online administered treatment, and will be used at the study endline. Participants state how strongly they agree/disagree with each of the following items: (a) the use of a computer to access treatment, (b) level of easiness of the online intervention, (c) duration of the potential effects of the treatment, and (d) potential recommendation of online interventions to others. Responses range from "*Agree very strongly*" to "*Disagree very strongly*". An additional question asks participants how helpful they have found the online intervention, with responses ranging from "*Very helpful*" to "*Not at all helpful*". Two open questions are also included that require participants to state the aspects of the intervention they most liked and disliked.

The EOT, developed by the researchers, will be presented at the end of every online session. The EOT includes three questions. The first asks participants to indicate where they

For peer review only - http://bmjopen.bmj.com/site/about/guidelines.xhtml

completed the online training session (e.g., home or office). The second is related to whether or not they have been distracted during the training and, if distracted, to indicate the source of distraction (e.g., telephone ringing). The third question asks participants to rate the level of their concentration during the online task on an 11-point Visual Analogue Scale (VAS) numbered from 0 (i.e., *no concentration at all*) to 10 (i.e. *very concentrated*).

For peer review only

References

1. Cleeland CS, Ryan KM. Pain assessment: global use of the Brief Pain Inventory. Ann Acad Med Singapore 1994;**23**(2):129-38.
2. Jensen MP. The validity and reliability of pain measures in adults with cancer. The Journal of Pain 2003;**4**(1):2-21 doi: 10.1054/jpai.2003.1.
3. Tan G, Jensen MP, Thornby JI, et al. Validation of the Brief Pain Inventory for chronic nonmalignant pain. The Journal of Pain 2004;**5**(2):133-37 doi: 10.1016/j.jpain.2003.12.005.
4. Zigmond AS, Snaith RP. The hospital anxiety and depression scale. Acta Psychiatr Scand 1983;**67**(6):361-70.
5. Bjelland I, Dahl AA, Tangen T, et al. The validity of the Hospital Anxiety and Depression Scale: An updated literature review. Journal of Psychosomatic Research 2002;**52**:69-77 doi: 10.1016/S0022-3999(01)00296-3.
6. Spielberger CD, Gorsuch RL, Lushene RE. *State Trait Anxiety Inventory*. Palo Alto, California: Consulting Psychologists Press, 1970.
7. Barnes LLB, Harp D, Jung WS. Reliability generalization of scores on the Spielberger State-Trait Anxiety Inventory. Educational and Psychological Measurement 2002;**62**(4):603 - 18 doi: 10.1177/0013164402062004005.
8. McNeil DW, Rainwater A. Development of the Fear of Pain Questionnaire-III. Journal of Behavioral Medicine 1998;**21**(4):389-410 doi: 10.1023/A:1018782831217.
9. Roelofs J, Peters ML, Deutz J, et al. The Fear of Pain Questionnaire (FPQ): further psychometric examination in a non-clinical sample. PAIN 2005;**116**(3):339-46 doi: 10.1016/j.pain.2005.05.003.
10. Hays RD, Stewart AL. Sleep Measures. Measuring functioning and well-being. In: Steweart AL, JEJ W, eds. Measuring function and well-being; the medical outcome study approach. Duke: Duke University Press, 1992:235-59.

For peer review only

11. Hays RD, Martin SA, Sesti AM, et al. Psychometric properties of the medical outcomes study sleep measure. Sleep Medicine 2005;**6**(1):41-44 doi: 10.1016/j.sleep.2004.07.006.

12. Viala-Danten M, Martin S, Guillemin I, et al. Evaluation of the reliability and validity of the Medical Outcomes Study sleep scale in patients with painful diabetic peripheral neuropathy during an international clinical trial. Health and Quality of Life Outcomes 2008;**6**(113) doi: 10.1186/1477-7525-6-113.

13. Richards D, Timulak L. Satisfaction with therapist-delivered vs. self-administered online cognitive behavioural treatments for depression symptoms in college students. British Journal of Guidance & Counselling 2013;**41**(2):193-207 doi: 10.1080/03069885.2012.726347.

For peer review only

Supplementary Material 3 – Think Aloud Study

A qualitative think aloud study[1 2] was conducted following the development of the initial version of the ABMT intervention to address the question: *What are the thoughts and first impressions of individuals with chronic musculoskeletal pain towards an online ABMT programme?* Ethical approval was obtained from the University of Southampton Research Ethics Committee (ERGO ID: 26486). Thirteen participants (mean age = 37.54, *SD* = 16.31, range 21 – 60 years) with chronic musculoskeletal pain were recruited from the South of England from a chronic pain support group.

Each participant met with the researcher (FH) on a single occasion to take part in a think aloud interview followed by a short semi-structured interview. Participants navigated their way through the iABMT Lifeguide website and completed the first assessment visual-probe task, with the researcher seated next to them. The researcher used an interview topic guide featuring standardized instructions that were read at the beginning of the study, as well as a list of pre-determined neutral prompts used when the participant stopped talking or showed a reaction to the material through their facial expressions. On occasions where the participant deviated from the topic, the researcher attempted to steer the participant's focus back to the task at hand. The prompts used were similar to those in former research[3], such as '*what are you thinking right now?*' and '*tell me why you think that*'.

Following the think-aloud interview, a short semi-structured interview took place for the researcher to ask questions about any specific aspects of the intervention that had not yet been discussed. Questions were also asked regarding thoughts about the intervention as a whole. This method of triangulation is commonly used to complement a think-aloud interview because it can be difficult to interpret spoken thoughts alone.[4 5] Following transcription of the interviews, thematic analysis using inductive encoding was used to identify themes that clearly represent the raw data, following guidelines set out by Braun and

For peer review only - http://bmjopen.bmj.com/site/about/guidelines.xhtml

Clarke.[6] Three themes and 13 subthemes were identified, which are shown in a thematic map in Figure S1 and defined in Table S1 below. While it is beyond the scope of this article to present the results of this thematic analysis in full, the main points resulting in amendments to the iABMT intervention are presented here.

Regarding the iABMT website, participants liked the continuity of design between webpages, although pointed out inconsistencies in the formatting of text, which have been rectified. On some pages the volume of text was considered to be too great, and therefore where possible text has been reduced, language simplified, better use of spacing made, and longer paragraphs divided into multiple shorter paragraphs. While some participants were positive about the inclusion of images, others felt additional visual stimuli could be added throughout. It was therefore decided to only include images where these would help emphasize the points being made. Regarding the visual-probe task sessions, one important practicality raised was ability of participants to stay seated for a sufficient amount of time in order to complete the task in a single session. An additional inclusion criteria has therefore been added to the study, being that participants must be able to sit at a personal computer for forty minutes. It was also mentioned that participants needed sufficient computer skills to take part in the study. The inclusion criteria were therefore amended to state that participants need both access to and familiarity using a Window-based computer.

For peer review only

References

1. Yardley L, Ainsworth B, Arden-Close E, et al. The person-based approach to enhancing the acceptability and feasibility of interventions. Pilot and Feasibility Studies 2015;**1**(1):37.

2. Van den Haak MJ, De Jong MD, Schellens PJ. Evaluation of an informational web site: three variants of the think-aloud method compared. Technical Communication 2007;**54**(1):58-71.

3. Morrison L, Moss-Morris R, Michie S, et al. Optimizing engagement with internet-based health behaviour change interventions: Comparison of self-assessment with and without tailored feedback using a mixed methods approach. British Journal of Health Psychology 2014;**19**(4):839-55 doi: 10.1111/bjhp.12083.

4. Charters E. The use of think-aloud methods in qualitative research an introduction to think-aloud methods. Brock Education Journal 2003;**12**(2).

5. Anthierens S, Tonkin-Crine S, Douglas E, et al. General practitioners' views on the acceptability and applicability of a web-based intervention to reduce antibiotic prescribing for acute cough in multiple European countries: a qualitative study prior to a randomised trial. BMC Family Practice 2012;**13**(101) doi: 10.1186/1471-2296-13-101.

6. Braun V, Clarke V. Using thematic analysis in psychology. Qualitative Research in Psychology 2006;**3**(2):77-101 doi: 10.1191/1478088706qp063oa.

For peer review only - http://bmjopen.bmj.com/site/about/guidelines.xhtml

Table S1. Definitions of themes and subthemes identified in the think aloud study

Theme/Sub-theme	Definition
A. AMBT website	Comments relating to the ABMT website including the post-intervention questionnaires
A1. Accessibility and usability	Comments relating to the ease with which participants were able to use the website and the extent to which it was deemed to be accessible
A2. Visual design	Comments relating to the visual design and appearance of the website, including colour, orientation of elements and text
A3. Questionnaires	Comments regarding any aspect of the post-intervention questionnaires, including their purpose and presentation
A4. Specific menu pages	Comments pertaining to the six main menu pages, which are titled (i) How does ABMT work, (ii) Will ABMT cure my pain, (iii) The team behind ABMT, (iv) Evidence for ABMT, (v) Why try ABMT, and (iv) What is it like to use ABMT?
B. Visual-probe task	Comments relating to any and all aspects of the visual-probe tasks
B1. Experiences of testing the intervention	Comments provided prior to and during testing of the visual-probe task
B2. Attitudes towards task	Positive and negative attitudes expressed by participants regarding the visual-probe task
B3. Practicalities	Comments pertaining to the feasibility and practicalities of completing the visual-probe task, including commitment needed
C. Influencing factors	All personal and situational factors that may affect the participants' experiences of testing the intervention
C1. Interviewing situation	Comments relating to the style of qualitative research adopted for the think aloud study
C2. Personal barriers	Comments relating to personal factors that hindered participants' completion of the visual-probe task
C3. Personal facilitators	Comments relating to personal factors that increased participants' ability to complete the visual-probe task and improved their attitude regarding it

For peer review only - http://bmjopen.bmj.com/site/about/guidelines.xhtml

STANDARD PROTOCOL ITEMS: RECOMMENDATIONS FOR INTERVENTIONAL TRIALS

SPIRIT 2013 Checklist: Recommended items to address in a clinical trial protocol and related documents*

Section/item	Item No	Description	Addressed on page number
**Administrative information**			
Title	1	Descriptive title identifying the study design, population, interventions, and, if applicable, trial acronym	1
Trial registration	2a	Trial identifier and registry name. If not yet registered, name of intended registry	1
	2b	All items from the World Health Organization Trial Registration Data Set	Registered with clinicaltrials.gov
Protocol version	3	Date and version identifier	1
Funding	4	Sources and types of financial, material, and other support	28
Roles and responsibilities	5a	Names, affiliations, and roles of protocol contributors	1, 28
	5b	Name and contact information for the trial sponsor	28
	5c	Role of study sponsor and funders, if any, in study design; collection, management, analysis, and interpretation of data; writing of the report; and the decision to submit the report for publication, including whether they will have ultimate authority over any of these activities	28
	5d	Composition, roles, and responsibilities of the coordinating centre, steering committee, endpoint adjudication committee, data management team, and other individuals or groups overseeing the trial, if applicable (see Item 21a for data monitoring committee)	________

For peer review only

**Introduction**			
Background and rationale	6a	Description of research question and justification for undertaking the trial, including summary of relevant studies (published and unpublished) examining benefits and harms for each intervention	4-6
	6b	Explanation for choice of comparators	4
Objectives	7	Specific objectives or hypotheses	6
Trial design	8	Description of trial design including type of trial (eg, parallel group, crossover, factorial, single group), allocation ratio, and framework (eg, superiority, equivalence, noninferiority, exploratory)	7-8
**Methods: Participants, interventions, and outcomes**			
Study setting	9	Description of study settings (eg, community clinic, academic hospital) and list of countries where data will be collected. Reference to where list of study sites can be obtained	6-8
Eligibility criteria	10	Inclusion and exclusion criteria for participants. If applicable, eligibility criteria for study centres and individuals who will perform the interventions (eg, surgeons, psychotherapists)	7
Interventions	11a	Interventions for each group with sufficient detail to allow replication, including how and when they will be administered	8-14
	11b	Criteria for discontinuing or modifying allocated interventions for a given trial participant (eg, drug dose change in response to harms, participant request, or improving/worsening disease)	19
	11c	Strategies to improve adherence to intervention protocols, and any procedures for monitoring adherence (eg, drug tablet return, laboratory tests)	13
	11d	Relevant concomitant care and interventions that are permitted or prohibited during the trial	7
Outcomes	12	Primary, secondary, and other outcomes, including the specific measurement variable (eg, systolic blood pressure), analysis metric (eg, change from baseline, final value, time to event), method of aggregation (eg, median, proportion), and time point for each outcome. Explanation of the clinical relevance of chosen efficacy and harm outcomes is strongly recommended	14-15
Participant timeline	13	Time schedule of enrolment, interventions (including any run-ins and washouts), assessments, and visits for participants. A schematic diagram is highly recommended (see Figure)	Table 1

For peer review only - http://bmjopen.bmj.com/site/about/guidelines.xhtml

Sample size	14	Estimated number of participants needed to achieve study objectives and how it was determined, including clinical and statistical assumptions supporting any sample size calculations	9
Recruitment	15	Strategies for achieving adequate participant enrolment to reach target sample size	7

Methods: Assignment of interventions (for controlled trials)

Allocation:

Sequence generation	16a	Method of generating the allocation sequence (eg, computer-generated random numbers), and list of any factors for stratification. To reduce predictability of a random sequence, details of any planned restriction (eg, blocking) should be provided in a separate document that is unavailable to those who enrol participants or assign interventions	8-9
Allocation concealment mechanism	16b	Mechanism of implementing the allocation sequence (eg, central telephone; sequentially numbered, opaque, sealed envelopes), describing any steps to conceal the sequence until interventions are assigned	8-9
Implementation	16c	Who will generate the allocation sequence, who will enrol participants, and who will assign participants to interventions	8-9
Blinding (masking)	17a	Who will be blinded after assignment to interventions (eg, trial participants, care providers, outcome assessors, data analysts), and how	8-9
	17b	If blinded, circumstances under which unblinding is permissible, and procedure for revealing a participant's allocated intervention during the trial	8-9

Methods: Data collection, management, and analysis

Data collection methods	18a	Plans for assessment and collection of outcome, baseline, and other trial data, including any related processes to promote data quality (eg, duplicate measurements, training of assessors) and a description of study instruments (eg, questionnaires, laboratory tests) along with their reliability and validity, if known. Reference to where data collection forms can be found, if not in the protocol	15-19, Supplementary material 2
	18b	Plans to promote participant retention and complete follow-up, including list of any outcome data to be collected for participants who discontinue or deviate from intervention protocols	11-12

Data management	19	Plans for data entry, coding, security, and storage, including any related processes to promote data quality (eg, double data entry; range checks for data values). Reference to where details of data management procedures can be found, if not in the protocol	19
Statistical methods	20a	Statistical methods for analysing primary and secondary outcomes. Reference to where other details of the statistical analysis plan can be found, if not in the protocol	15-19
	20b	Methods for any additional analyses (eg, subgroup and adjusted analyses)	17
	20c	Definition of analysis population relating to protocol non-adherence (eg, as randomised analysis), and any statistical methods to handle missing data (eg, multiple imputation)	17
**Methods: Monitoring**			
Data monitoring	21a	Composition of data monitoring committee (DMC); summary of its role and reporting structure; statement of whether it is independent from the sponsor and competing interests; and reference to where further details about its charter can be found, if not in the protocol. Alternatively, an explanation of why a DMC is not needed	________
	21b	Description of any interim analyses and stopping guidelines, including who will have access to these interim results and make the final decision to terminate the trial	________
Harms	22	Plans for collecting, assessing, reporting, and managing solicited and spontaneously reported adverse events and other unintended effects of trial interventions or trial conduct	19
Auditing	23	Frequency and procedures for auditing trial conduct, if any, and whether the process will be independent from investigators and the sponsor	________
**Ethics and dissemination**			
Research ethics approval	24	Plans for seeking research ethics committee/institutional review board (REC/IRB) approval	19
Protocol amendments	25	Plans for communicating important protocol modifications (eg, changes to eligibility criteria, outcomes, analyses) to relevant parties (eg, investigators, REC/IRBs, trial participants, trial registries, journals, regulators)	19

For peer review only - http://bmjopen.bmj.com/site/about/guidelines.xhtml

Consent or assent	26a	Who will obtain informed consent or assent from potential trial participants or authorised surrogates, and how (see Item 32)	9, 19
	26b	Additional consent provisions for collection and use of participant data and biological specimens in ancillary studies, if applicable	________
Confidentiality	27	How personal information about potential and enrolled participants will be collected, shared, and maintained in order to protect confidentiality before, during, and after the trial	19
Declaration of interests	28	Financial and other competing interests for principal investigators for the overall trial and each study site	28
Access to data	29	Statement of who will have access to the final trial dataset, and disclosure of contractual agreements that limit such access for investigators	28
Ancillary and post-trial care	30	Provisions, if any, for ancillary and post-trial care, and for compensation to those who suffer harm from trial participation	________
Dissemination policy	31a	Plans for investigators and sponsor to communicate trial results to participants, healthcare professionals, the public, and other relevant groups (eg, via publication, reporting in results databases, or other data sharing arrangements), including any publication restrictions	19
	31b	Authorship eligibility guidelines and any intended use of professional writers	28
	31c	Plans, if any, for granting public access to the full protocol, participant-level dataset, and statistical code	19
**Appendices**			
Informed consent materials	32	Model consent form and other related documentation given to participants and authorised surrogates	Supplementary materials
Biological specimens	33	Plans for collection, laboratory evaluation, and storage of biological specimens for genetic or molecular analysis in the current trial and for future use in ancillary studies, if applicable	N/A

*It is strongly recommended that this checklist be read in conjunction with the SPIRIT 2013 Explanation & Elaboration for important clarification on the items. Amendments to the protocol should be tracked and dated. The SPIRIT checklist is copyrighted by the SPIRIT Group under the Creative Commons "Attribution-NonCommercial-NoDerivs 3.0 Unported" license.

For peer review only
